# Imidazole[1,5-a]pyridine derivatives as EGFR tyrosine kinase inhibitors unraveled by umbrella sampling and steered molecular dynamics simulations

**DOI:** 10.1038/s41598-024-62743-3

**Published:** 2024-05-28

**Authors:** Duc Toan Truong, Kiet Ho, Huynh Thi Yen Nhi, Van Ha Nguyen, Tuan Thanh Dang, Minh Tho Nguyen

**Affiliations:** 1 Laboratory for Chemical Computation and Modeling, Institute for Computational Science and Artificial Intelligence, Van Lang University, Ho Chi Minh City, 70000 Vietnam; 2 Faculty of Applied Technology, School of Technology, Van Lang University, Ho Chi Minh City, 70000 Vietnam; 3 Institute for Computational Science and Technology (ICST), Quang Trung Software City, Ho Chi Minh City, 70000 Vietnam; 4 Faculty of Chemistry, VNU University of Science, Vietnam National University, Hanoi, 19 Le Thanh Tong, Hoan Kiem, Hanoi, 11021 Vietnam

**Keywords:** Imidazole[1,5-a]pyridines, Epidermal growth factor receptor (EGFR), Tyrosine kinase inhibitors (TKI), Absolute protein binding free energy, Umbrella sampling molecular dynamics simulation, Steered molecular dynamics simulation, Biochemistry, Biophysics, Cancer, Chemistry

## Abstract

Although the use of the tyrosine kinase inhibitors (TKIs) has been proved that it can save live in a cancer treatment, the currently used drugs bring in many undesirable side-effects. Therefore, the search for new drugs and an evaluation of their efficiency are intensively carried out. Recently, a series of eighteen imidazole[1,5-a]pyridine derivatives were synthetized by us, and preliminary analyses pointed out their potential to be an important platform for pharmaceutical development owing to their promising actions as anticancer agents and enzyme (kinase, HIV-protease,…) inhibitors. In the present theoretical study, we further analyzed their efficiency in using a realistic scenario of computational drug design. Our protocol has been developed to not only observe the atomistic interaction between the EGFR protein and our 18 novel compounds using both umbrella sampling and steered molecular dynamics simulations, but also determine their absolute binding free energies. Calculated properties of the 18 novel compounds were in detail compared with those of two known drugs, erlotinib and osimertinib, currently used in cancer treatment. Inspiringly the simulation results promote three imidazole[1,5-a]pyridine derivatives as promising inhibitors into a further step of clinical trials.

## Introduction

The epidermal growth factor receptor (EGFR), which is a well-known transmembrane protein, plays an important role in the cell proliferation. This protein was first introduced in 1980 by the biochemist Stanley Cohen et al.^1^ and their work was subsequently awarded by the Nobel Prize in Physiology or Medicine for 1986, jointly with the biologist Rita Levi-Montalcini for their discovery of *epidermal growth factor* (EGF) and *nerve growth factor* (NGF), respectively, who could show how the growth and differentiation of a cell is regulated. Accordingly, both the NGF and EGF were the first of several growth-regulating signal substances to be discovered and characterized. Cohen and coworkers focused on the relationship between an epidermal growth factor (EGF) and an epidermal growth factor receptor (EGFR)^2^.

A single EGFR contains three chains, namely, an extracellular domain, a helix-transmembrane domain and an intracellular domain. During a normal cell development, the interaction between an EGF and an extracellular domain of the EGFR protein tends to stabilize a dimerization of two EGFR molecules and subsequently signal the beginning of cell growth and proliferation. Later, the EGFR was at the first time linked to cancer via the 1984 reports of Downward, Ullrich and Schlessinger^3,4^. Subsequent research work has identified the EGFR as responsible for expression^5^ and generation of various cancer types including carcinomas, non-cell lung cancer, liver cancer, malignant gliomas, etc.^6–8^. It has been established that the cancer caused in 2020 the disparition of about 10 million people and has become an emergency healthcare issue all over the world^9^.

Inspiringly, there is still high hope for an effective cancer treatment since we have known, among other things, that the inhibition of an intracellular domain, the so-called tyrosine kinase, can block an over-expressive activity of EGFR protein^10^. In this context, many inhibitors identified as EGFR Tyrosine Kinase Inhibitors (TKIs) have been synthesized with the aim to target the tyrosine domain of EGFR protein. For example, one of the most well known inhibitor, the getifinib (or Iressa), was promoted in 2001^11^ by Astra Zeneca, and then quickly proceeded to trial clinical in 2002^12,13^ and successfully passed the FDA approval only a few years later^14^. Drugs can save more life but it is never easy when a cancer treatment faces difficulty with regard to the efficacy of TKIs due to several issues including the undesirable side effect, engagement of efflux pumps, competition of ATP^15,16^. Various genomic mutations in the structure of tyrosine kinase domain have been reported in the context that these variants could escape the effects of some drugs used, and more seriously, they appeared only few years after using these drugs^17,18^. In the viewpoint of evolutionary biological science, an EGFR attempts to alter the spatial organization of the functional area in order to: (i) prevent the TKI action, and (ii) enhance the ATP activity^19–21^. Drug resistance turns out to be only a matter of time, and seems to be getting worse when it usually takes longer time for approval of a new drug than it does for the appearance of a new mutation^19–21^. Therefore the search for new efficient drug for inhibiting the EGFR, either a wide type or mutants, is a key important issue in the cancer treatment. In this intensive search, computational approaches at the atomistic level, could significantly help us owing to their relatively lower costs, faster speed, and their wider accessibility, particularly in developing countries^22^.

Owing to the available of TKI-EGFR crystal complexes that are uploaded in the PDB bank, and the assistance of high performance computers, relevant computations are actually able to generate plenty of fresh information. In fact, the tyrosine kinase domain, a valuable target for computational studies, has greatly attracted the attention of researchers whose subjects as listed in Table S1 point out a rich variety of the issues considered. The molecular structure of a tyrosine kinase domain is known to be formed by 542 amino acids that are separated into three regions including a β-sheet N lobe, an ATP binding region and an α-helix C lobe. Computational approaches have much contributedto the knowledge and understanding of EGFR spatial structure, the protein–TKIs interaction and especially into the screening of new inhibitors. Due to the complexity of the EGFR kinetics, molecular dynamics (MD) simulations emerge as an effective tool for providing us with atomistic observation. In addition, the use of these computational approaches also allow the binding free energies between TKIs and EGFR protein, both wide type and mutants, to be quantitatively determined^23–25^.

Let us briefly summarize the previous determinations of the binding free energies. In 2012, Park et al.^23^ aimed to evaluate the binding free energy of the erlotinib drug to EGFR proteins in both active and inactive types. These authors obtained two disparate ΔG values of − 9.3 and − 28.2 kcal/mol via two different tools including the docking glide score (DGS) and the molecular mechanics Poisson-Boltzmann Surface Area (MM-PBSA) method, respectively. Tadsaporn^24^ computed the binding free energies between most of the well known and currently used drugs such as erlotinib, gefitinib, afatinib, dacomitinib, and Osimertinib with the EGFR kinase domain. This study also revealed the MM-PBSA values of binding free energies of − 14.1 and − 6.2 kcal/mol for erlotinib and osimertinb, respectively. In a latest study, Zhou et al.^25^ reported an absolute binding free energy − 10.5 kcal/mol for the of erlotinib-EGFR interaction by applying the free energy perturbation (FEP) method^25^. Based on the IC50 values, a subsequent study of Tadsaporn et al.^24^ came up with the experimental values − 10.7 and − 8.7 kcal/mol for the binding free energies of the latter drugs.

Another technique of enzyme kinetic study, as implemented by Zhai et al.^26^, contributed more insights into the selectivity of some FDA approved drugs. For both erlotinib and osimertinib drugs, Zhai’s bioassays showed the experimental inhibitory constants of *K*_i_ to the EGFR wide type amounting to 0.29 (nm) and 930 (nm), being equal to the binding free energy values of − 13.1 and − 8.3 kcal/mol, respectively. These findings, although again somewhat disparate due to the differences of techniques and initial binding structures, were consensual to predict that the erlotinib drug binds more strongly to an EGFR wide type than the osimertinib. These observations are also consistent with data from a previous trial clinical^27^. In this context, with the purpose to establish a reference value for ranking those of new compounds, we first attempt to determine the absolute binding free energies of both erlotinib and Osimertinib drugs by using the umbrella sampling method whose values are not known yet.

Of the various biologically relevant organic scaffold, the imidazol[1,5-a]pyridine (ImPy) core^28^ has recently emerged as an promising platform for drug discovery due to their many appropriate characteristics, such as the water solubility, biocompatibility and easy uptake by living cells. A number of ImPy derivatives have been demonstrated to be good anticancer and cardiotonic agents^29^. In addition, several Impy-based compounds also display inhibiting activities toward various enzymes, such as HIV-1 protease^30^, phosphodiesterase, kinase and 5-HT4 receptor^31^.

In a recent experimental study, we successfully developed an efficient iodine-promoted route for the synthesis of a series of symmetrical bis(1-imidazo[1,5-a]pyridyl)arylmethanes that bear two ImPy units connected by a arylmethylene (cf. Table 1)^29^. Interestingly, the preliminary study showed that all the synthesized bis(1-imidazo[1,5-a]pyridyl)arylmethanes compounds display a high cytotoxicity toward 3 different human cancer cell lines, including lung adenocarcinoma (SK-LU-1), malignant liver cancer (HepG2), and human breast adenocarcinoma (MCF-7). As part of our continuing effort to gain insights into the mode of action for the compounds and to employ computational studies to provide us with a judicious choice of compounds for the next step in vitro testing on proteins and enzymes, we set out to carry out the present theoretical study. Our main purpose is to determine the abilities of these novel compounds in their binding with the EGFR wide type protein and to further understand their bioactivities at an atomistic level making use of molecular dynamics simulations. In this scheme, a key goal is to figure out some lead compounds to be considered in a further step of clinical testing. To achieve this goal, the umbrella sampling (US) method is implemented to verify the new compound binding affinities with protein because this method has been known as one of the most suitable approaches for generating the potential of mean force (PMF) of the ligand-receptor, ion- protein, etc.^32–35^. Although the US method can allow us to extract the absolute binding free energy of a ligand–protein system, its cost is too high in terms of computing time^36,37^, which goes beyond our actual computing resources. In the situation that we do not have appropriate computing power, computations using the US method for all 18 compounds considered (Table 1) is simply not possible. Therefore, we propose an alternative strategy including four successive steps including: (1) a docking, (2) molecule dynamics (MD) simulation, (3) steered molecule dynamics (SMD) simulation and finally (4) an umbrella sampling (US) in the last step for the most relevant compounds. The details of our protocol are described in the following section. As for a reference, the present study also evaluates the binding free energy between two known drugs, namely the erlotinib and Osimertinib, with the EGFR wide type. Based on this computational protocol we can successfully rank the binding affinities of 18 compounds considered. Our present work consumes a total of 10 ms simulation time and it is applicable for screening compounds from a large database containing a dozen or up to hundreds of ligands. From the 18 compounds considered (Table 1), simulation results allow us to select three prospective candidates for further studies that are also based on their specificity, selectivity, efficacy and safety. In addition, this examination not only represents a characterization of each novel compound by quantum chemical calculation but also reveals the interaction features between their chemical sub-groups and nine key residues of the EGFR tyrosine domain.

**Table 1:**
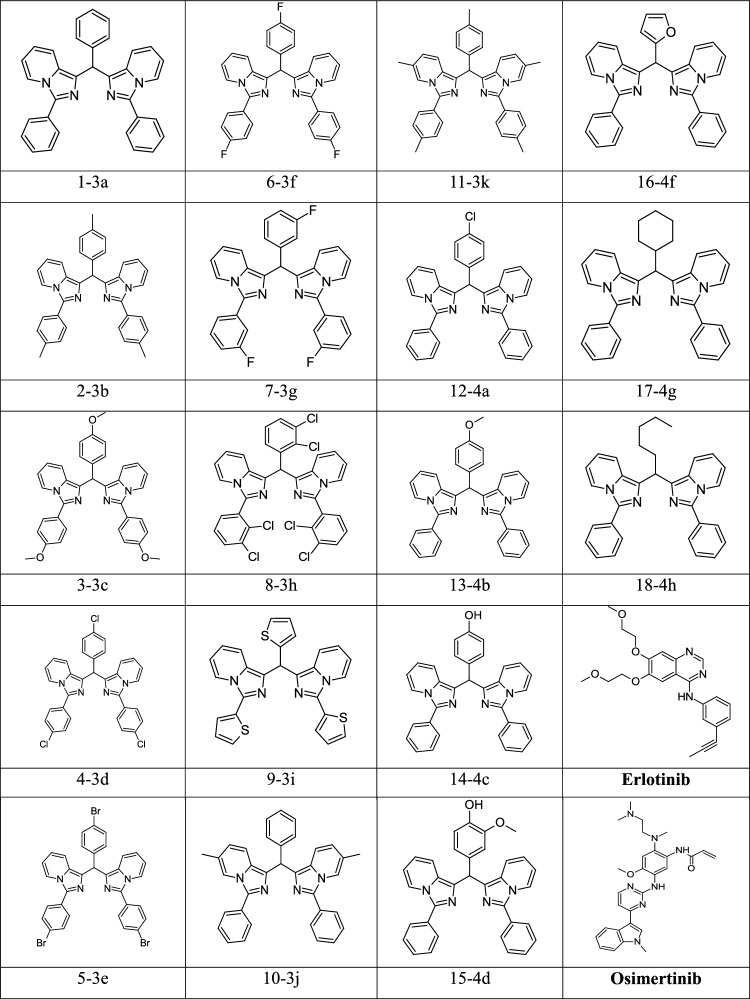
Chemical structures of 18 novel compounds and two FDA-approved drugs.

## Materials and methods

### EGFR: ligand system’s preparation

**Figure 1:**
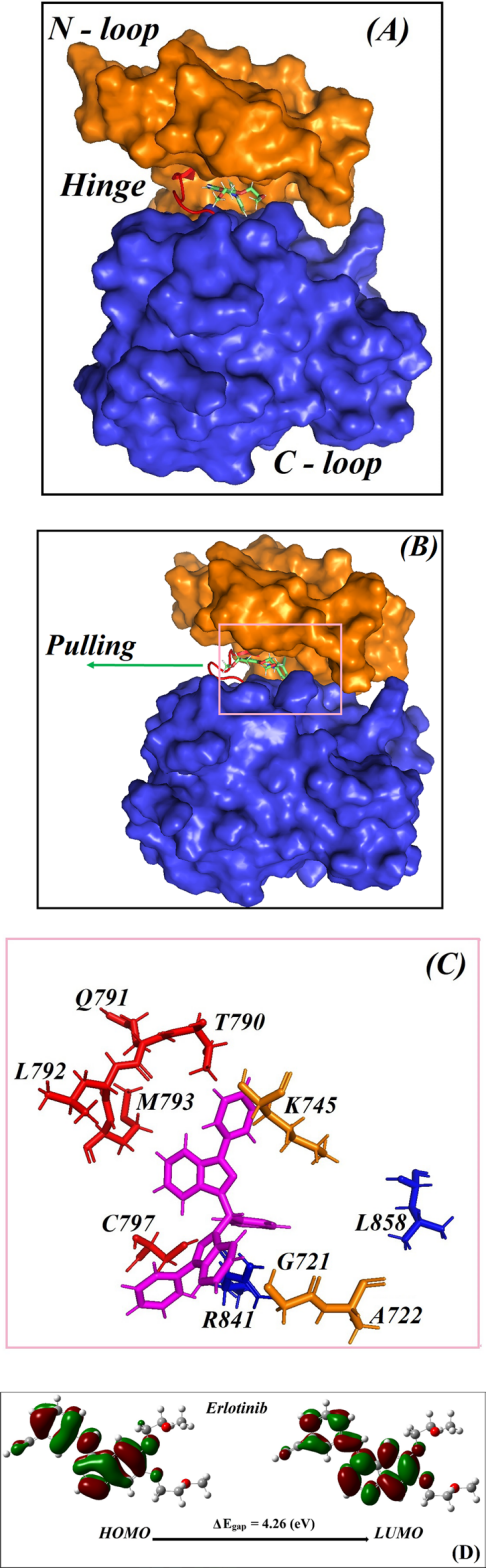
Structure of EGFR tyrosine kinase domain from PDB-ID 1M17 is shown. (**A**) Front view: N-loop, C-loop and hinge are also noted, (**B**) a side view, (**C**) zoom of 10 amino acids surrounding erlotinib (in magenta stick) are named, and (**D**) the shape of frontier orbitals HOMO and LUMO of the erlotinib generated by DFT method. Their energy gaps are relatively similar, being 3.5 to 4.0 eV, and larger than the energy of a semiconductor (being below 3.2 eV).

The crystal structures of the two known drugs, erlotinib and Osimertinib, located in an active conformation within the binding site of EGFR tyrosine kinase domain, are downloaded from the PDB bank, 1M17^38^ and 4ZAU^39^ (PDB-ID). A tyrosine kinase domain consists of N-lobe and Clobe, connected with each other by a hinge sequence. Adjacent to the ATP binding region, the interface between two lobes is also occupied by an A-loop and an α-loop. The salt bridge (K745—G762) and DFG-motif (D855, F856, G857 in A—loop) play an important role in stabilizing the α-loop connection and the protein substrate’s function^40^. The majority of ATP competitive inhibitors works by entering into the deep hydrophobic pocket of the kinase domain which gives them their efficacy. According to the binding modes when a ligand interacts with a tyrosine kinase domain, Zhao^41^ classified all available PDB structures into six groups. Most of ligands were found binding to the ATP region where 39 residues are discovered to be in β-sheets, hinge regions, and the α-helix. The identity of amino acids that surround the ATP binding site helps us to explore how an inhibitor represents its interactive picture with EGFR tyrosine kinase domain. In this study, ten amino acids including the G721, A722, K745, T790, Q791, L792, M793, C797, R841 and L858, can be viewed in Fig. 1 below, are used to calculate the averaging distance from the 18 novel compounds, as we do in the simulation part described below, mainly for one purpose, determination as to whether or not the compound left the ATP active site. These amino acids are called locating residues.

The crystal structures of both erlotinib and Osimertinib drugs located in the binding site of the EGFR tyrosine kinase domain, in an active conformation, are downloaded from the PDB bank, 1M17^38^ and 4ZAU^39^ (PDB-ID). The 2D conformations of 18 novel compounds are taken from our related previous study^29^. In summary, twenty structures of small ligands are examined including 18 novel compounds and two known ones (erlotinib and Osimertinib). Their conformations are optimized and their electrostatic potential maps are calculated using density functional method with the aid of the Gaussian 16^42^ program. Atomic net charges of the ligands are derived using the RESP^43^ method. The Antechamber module of AMBER Tools is used to calculate additional parameters for the compounds using the General Amber Force Field (GAFF)^44,45^. Structure of the EGFR protein, taken from PDB-ID 1M17 after removing that of erlotinib, is used as the receptor in the docking step.

### Docking method

The ligands considered are docked into receptors by using the AutoDock Vina^46^ program. During the docking process, each ligand moves within a box of size 80 × 80 × 80 Å (center − 56.673, − 1.725, − 23.122) which is large enough to cover the ATP binding region. Spacing grid and exhaustiveness are, 0.375 and 400, respectively. The MGLTools version 1.5.7^47^ is used to visualize and generate PDBQT file format for receptor and ligand which is the subsequent input for AutoDock Vina script. When done, the scoring function returns several models with its corresponding binding energy. Accordingly, the best model is taken, often the first one which has the lowest binding energy as the initial conformation for the next step.

### Molecular dynamics simulations

With the support of the pdb2gmx package from Gromacs software^48^ for proteins and ions we apply the amber ff99SB-ILDN force fields^49^ while the TIP3P^50^ force field is used for water molecules. The system is neutralized by addition of Cl^−1^ anions. The 90-90-90 (angstroms^3^) periodic box containing more than 30,000 water molecules is made to guarantee that the separation between the EGFR protein and the boundary is greater than 16 angstroms. This condition ensures that the protein does not interact with itself. With positional restrictions on ligand atoms and protein heavy atoms, the energy of solvated systems is then minimized. The system is equilibrated for 1 ns under constant volume (NVT) then run for 1 ns at constant pressure and temperature (NPT). Electrostatic interactions are calculated using the particle mesh Ewald method^51^. A cutoff radius of 10 Å is applied for both the electrostatic interactions and the Van der Waals interactions. The P-LINCS algorithm^52^ is employed to constrain all covalent hydrogen bonds. Pressure is kept at 1 bar using the Parrinello-Rahman^53^ pressure coupling and temperature is kept at 298 K using velocity rescaling with a stochastic term. The time constants for the temperature and pressure couplings to the bath were 0.1 and 2 ps, respectively. All molecular dynamics (MD) simulations carried out in this study are performed in periodic boundary conditions using the GROMACS program (version 2020)^48^. Time step is set at 2 fs and snapshots are saved every 5000 steps during each of MD run.

### Steered molecular dynamics (SMD) simulation

In a typical SMD simulation, an external force is used to drive the ligand out of the protein’s binding position. This non-equilibrium method has been known to be less computing time consuming, but the obtained results are still as accurate enough to classify the relative ligand–protein affinities based on the principle that the larger the rupture force obtained, the higher the binding affinity system contains^54^. In this study, the pulling direction is navigated on the basis of the guiding of Caver^55^ web server. At first the complex is aligned in such a way that the ligand’s center of mass (COM) is placed at the original position (0,0,0). The pulling direction is aligned to the z-direction of the simulation box. To ignore the drifting of protein, a harmonic potential is used to restrain the motion of protein’s Ca atoms (k = 1000 kJ/mol/nm). For each protein–ligand complex, 100 independent trajectories are performed with a pulling velocity v = 5 (m/s) to enhance the sampling conformation. Pulling time is set to 800 (ps). Force–time and displacement–time profiles are recorded every 10 fs. Other related parameters can be found in a previous publication^56^.

### Umbrella sampling (US) method

Although the umbrella sampling (US) is one of the highly reliable approaches for computing the equilibrium free energy, it is not able to generate by itself the configurations of protein–ligand dissociation process. For this task, preliminary SMD simulations must be carried out. For each of the ligand-EGFR representative conformations collected from a MD step, a very slow SMD running with a small loading rate of v = 0.1 nm/ns, is run. Snapshots are saved at every 1 ps. For each saving snapshot, the distance between ligand’s center of mass and its initial position is used as variables. More than 150 protein–ligand configurations are chosen with the requirement that the position of the ligand moves every 0.02 nm. All these configurations are prepared as input structures to especially enhance the sampling in transition state. At each window, a 10 ns MD simulation is run after three common equilibrium steps (EM, 1 ns NVT, 1 ns NPT same as described above in the MD step) and the ligand’s center of mass is recorded. The Gmx WHAM package is then used to construct the free energy curve in the potential mean force (PMF) step. A 10 ns of MD running from each window appears long enough to obtain the overlap between adjacent windows. Due to the high cost of these computation, only a few of the hit ligand-EGFR conformations from SMD step could be performed in the PMF calculations.

### Strategy for screening important compounds

**Figure 2:**
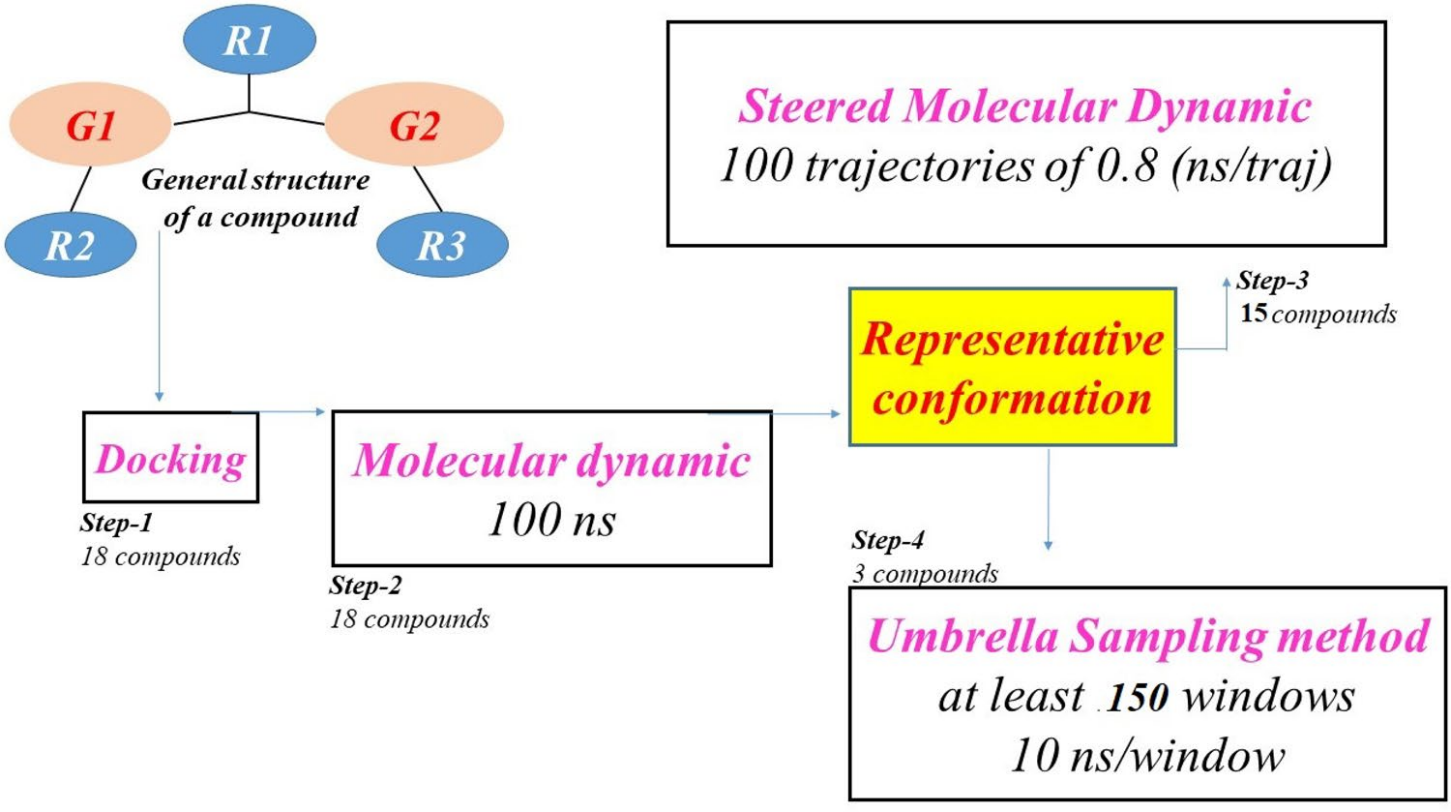
A schematic description of our computational protocol.

Due to the limitation of our computing resources, development of an appropriate strategy which can effectively classify the binding affinities of new compounds in a realistic drug design scenario is of crucial importance. We first apply the docking method to insert small ligands into their initial binding mode. After docking ligands into the active site of the EGFR, the resulting ligand-EGFR structures are run for 100 ns MD simulation. This step aims to establish the stability of the ligand–protein interaction. In other words, the system is given a chance for finding a better binding conformation. To analyze the outcome of MD simulations, several quantities are computed including the root mean square deviation of protein or ligand (RMSD), the number of contact between ligand and EGFR protein (numcount), the number of hydrogen bond (hbond) being formed between ligand and protein, the solvent accessible surface areas (SASA) of ligand, the nonbonding interaction energy (IE energy) and the minimal distance (mindist) from the ligand to key residues of EGFR. Quantitative data are collected into a matrix. On the basis of these numerical data, we evaluate one by one the stabilization of ligand–protein interaction. The goal of this MD step is to select as many as possible the number of compounds into the next SMD and US steps. Compounds that do not have any significant expectation will be dismissed. In the case of a compound which can pass the MD step, a representative snapshot from MD simulation is extracted for use as the input of SMD simulation. The last 50 ns of the MD trajectory of each system is then analyzed to take in account the value of solvent accessible surface areas and the value of nonbonding interaction energy. The free energy landscape is plotted to extract the representative structure corresponding to each global minimum. Ligand–receptor representative conformations owning the highest probability from MD step are chosen as initial structure of the following SMD performance. As a result, 15 of 18 novel compounds considered could be selected into the SMD simulation step. Indeed, when applying an external force to rupture the ligand–protein dissociation, SMD simulations have been proved that it can be an effective tool to rank the ligand–receptor binding affinities. From the data obtained in the SMD step, three compounds which receive the best results for rupture force or external work will be applied to the PMF calculation. In following the whole protocol summarized in Fig. 2, we believe that a compound which appears in the final step will have a high percentage to be considered as a strong candidate for further testing toward a medical use.

## Results and discussion

### Conformation of compounds obtained from quantum chemical calculations

The geometries of the 18 compounds studied are optimized by using Gaussian 16^42^ software with density functional theory (DFT) at level B3LYP/6-31 + G(d,p), which is appropriate enough to calculate for compounds contained C, H, O, N, S atoms. Additionally, we also calculate the harmonic vibrational frequencies by using normal mode method to characterize the optimized structures as local energy minima.

Several chemical characteristics are evaluated including the energy of highest occupied molecular orbital ${\text{E}}_{\text{HOMO}}$, energy of lowest unoccupied molecular orbital ${\text{E}}_{\text{LUMO}}$, energy gap ${\text{E}}_{\text{gap}}=\left|{\text{E}}_{\text{HOMO}}\right|-\left|{\text{E}}_{\text{LUMO}}\right|$, vertical ionization energy $\text{IE}=-{\text{E}}_{\text{HOMO}}$, vertical electron affinity $\text{EA}=-{\text{E}}_{\text{LUMO}}$, electronegativity $\upchi =\frac{1}{2}\left(\text{IP}+\text{EA}\right)$, chemical hardness $\upeta =\frac{1}{2}\left(\text{IP}-\text{EA}\right)$, chemical softness $\text{S}=\frac{1}{\upeta }$, chemical potential $\upmu =-\upchi$, electrophilicity $\upomega =\frac{{\upmu }^{2}}{2\upeta }$.

Table S1 lists some molecular quantities for the 18 compounds plus 2 reference drugs, erlotinib and Osimertinib. The energy gap of erlotinib amounts tp 4.3 eV, slightly higher than the others. The energy gap of 18 novel compounds (index from 1 to 18) are quite similar with that of Osimertinib, being approximate 3.5 to 4.0 eV, of which the lowest one belongs compound 9 with the existence of a thiophene ring. The frontier orbitals (HOMO and LUMO) of all compounds generated by DFT method can be viewed in the Supporting Information file (SI file, Fig. S60). All gap values are in phase transition range of insulator and semiconductor, therefore these compounds are able to interact with biomolecules as protein or enzyme. Additionally, 18 compounds considered are stable because of their negative chemical potential, and they behave like an electron-withdrawing compound with electron affinities ranged from 3.0 to 3.465 eV, that are relatively large. The chemical hardness, chemical softness and electrophilicity are also approximately similar to those of the 2 reference compounds. As compared to the two reference compounds, all DFT results imply that the new synthetic compounds can be further tested for their biological properties.

### Initial ligand–protein binding mode from docking approach

Docking is the only tool that can help us to quickly insert a ligand into a region of a receptor. Related results are listed in Table 1. However, since the docking method basically ignores not only the interaction of protein’s side chain but also the vibration of protein backbone, this leads to a necessary molecular dynamics simulation in a next step.

### 15/18 novel compounds pass the examination of 100 ns molecular dynamics simulation

To explore the ligand–protein interaction, eighteen ligand-EGFR docking structures are now examined by 100 ns MD simulations. We quantify the number of hydrogen bonds, the number of contacts, the solvent accessible surface areas of ligand, the non-bounding interaction energy and the minimal distance connecting from ligand to 10 key residues. These quantities are computed to build a library of numerical information. In view of the fact that the number of 18 novel compounds considered in this study is relatively small, we could compare them one by one and manually screen the compounds that interact with the EGFR active sites. The effort devoted in this process aims to introduce as many as possible the number of compounds into the following step. At first, large distances presented in Table 2 and Table S2 (SI file) show that three compounds (**3-3c, 10-3j** and **15-4d**) are completely left so far from the ATP binding region of EGFR and their interaction with key amino acids is cut. In other words, these compounds do not tend to be associated with an EGFR active site. Consequently, all other numerical quantities analyzed from the **3-3c, 10-3j** and **15-4d** MD simulations are misleading. Passing the MD step, 15 remaining compounds can be selected to go into a further step, and three of them are removed for further consideration.

**Table 2:** Mean values averaged from the last 20 ns of 100 ns MD simulation of nonbonding interaction energy; combined from Coulomb and Van der Waals potential energies.

N	Ref.	Docking result (kcal/mol)	Distance to T790 (nm)	RMSD (nm)	Numcount	SASA (nm^2^)	Ligand–protein interaction energy (kcal/mol)	Hydrogen bond (number)
1	3a	− 10.2	0.25	0.21	2608	7.83	− 54.2	1.73
2	3b	− 11.0	0.81	0.27	1714	8.76	− 41.6	0.0
3	**3c**	− 9.2	**2.36**	**0.35**	**1049**	**9.24**	− **61.2**	**1.94**
4	3d	− 10.7	0.28	0.12	2220	8.33	− 48.4	0.0
5	3e	− 10.6	0.82	0.14	1499	8.43	− 49.3	0.01
6	3f.	− 10.6	0.35	0.09	2094	7.92	− 61.5	1.46
7	3 g	− 10.9	0.29	0.12	1918	8.02	− 65.0	1.32
8	3 h	− 10.6	0.25	0.05	2677	7.81	− 79.9	1.54
9	3i	− 8.5	0.82	0.16	1260	7.22	− 40.0	0.07
10	**3j**	− 9.4	**1.03**	**0.3**	**1230**	**8.54**	− **31.1**	**0.44**
11	3 k	− 9.4	0.84	0.31	1894	9.44	− 41.0	1.26
12	4a	− 9.8	0.65	0.24	1697	8.1	− 51.3	0.87
13	4b	− 10.1	0.86	0.3	1655	8.14	− 59.4	0.36
14	4c	− 10.1	0.56	0.26	1804	7.8	− 53.1	1.25
15	**4d**	− 10.3	**1.77**	**0.35**	**1804**	**8.42**	− **43.9**	**1.09**
16	4f.	− 10.0	1.01	0.24	1168	7.59	− 49.4	0.03
17	4 g	− 10.2	0.69	0.27	2034	8.08	− 66.6	1.09
18	4 h	− 8.3	0.38	0.19	2145	8.17	− 53.8	0.66

A contact is counted when the distance from one ligand’s atom and one protein’s atom becomes smaller than 0.6 nm. Minimal distance from ligand to one of the most important residues T790 is shown. Averaging minimal distances to 10 locating residues are listed in Table S2 (SI file).

Significant values are in bold.

In Table 2 and Table S2 (SI file), the averaged value of various numerical information from the last 20 ns of all 100 ns MD simulation are listed. Figure 3 illustrates the data obtained from a typical case, namely MD simulation of compound **1-3a**. The number of contacts (denoted by numcount) and the nonbonding interaction energy (denoted by energy) of **1-3a** with respect to the time dependence are shown. According to these results, compound **1-3a** is recognized after MD step to move into a better new position. Similar data from other 17 compounds can be viewed in Figs. S1–S17 (SI file).

**Figure 3:**
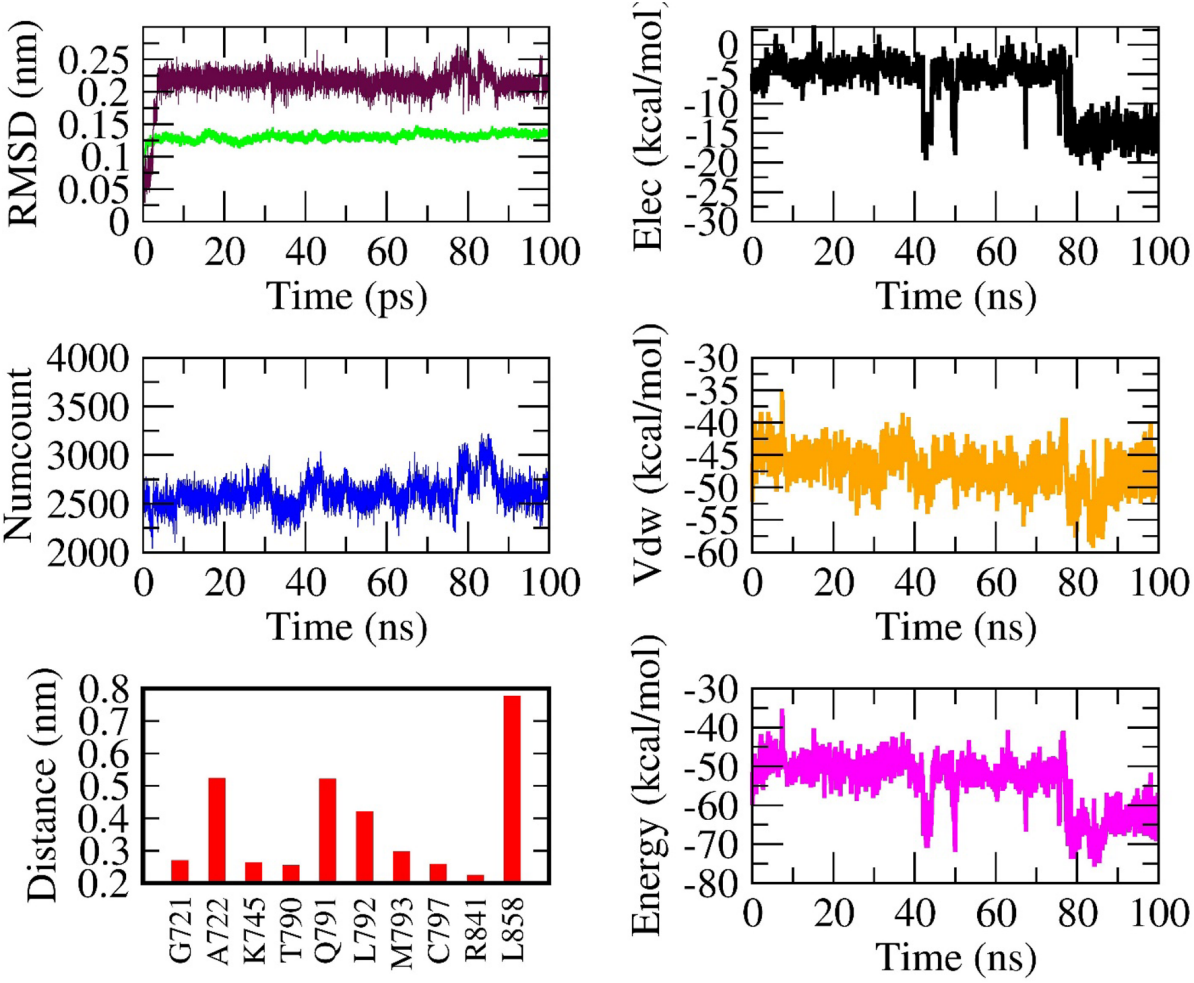
Results obtained from 100 ns MD simulations for compound** 1-3a** and EGFR complex. Time dependent root-mean-square deviation of protein and ligand are plotted in green and maroon, respectively. Number of contacts between **1-3a** with respect the time is shown in blue. Nonbonding interaction energy (in magenta) is combined from both Coulomb (in black) and Van de Waals potential energies (in orange). The mean of minimal distances (averaging in the last 20 ns of simulation) from compound **1-3a** to ten chosen residues located in the ATP region are also shown in red bar.

Because the residue T790 is located deeply insight in the ATP binding region of EGFR tyrosine domain, a compound containing a distance to T790 smaller than 0.6 nm, is expected to become a good inhibitor against EGFR. In searching in Table 2, we can find that the compounds denoted as **1, 4, 6, 7, 8, 14** and **18** are placed in short distances of 0.25, 0.28, 0.35, 0.29, 0.25, 0.56 and 0.38 nm, respectively, to the to T790. Many of these distances are actually smaller than 0.35 nm which is a value ranged within the ligand and receptor distance to easily form a hydrogen bond. All other compounds, except for **3-3c**, **10-3j** and **15-4d**, can thus be selected for the next examination, which is the determination of their binding affinity performed by the steered molecular dynamics simulation (SMD). Before carrying out these SMD computations, we extract a representative as one of the most important results that MD simulations can generate for the ligand-EGFR complexes.

#### Representative structure reveals a diversity of ligand-receptor bonding

**Figure 4:**
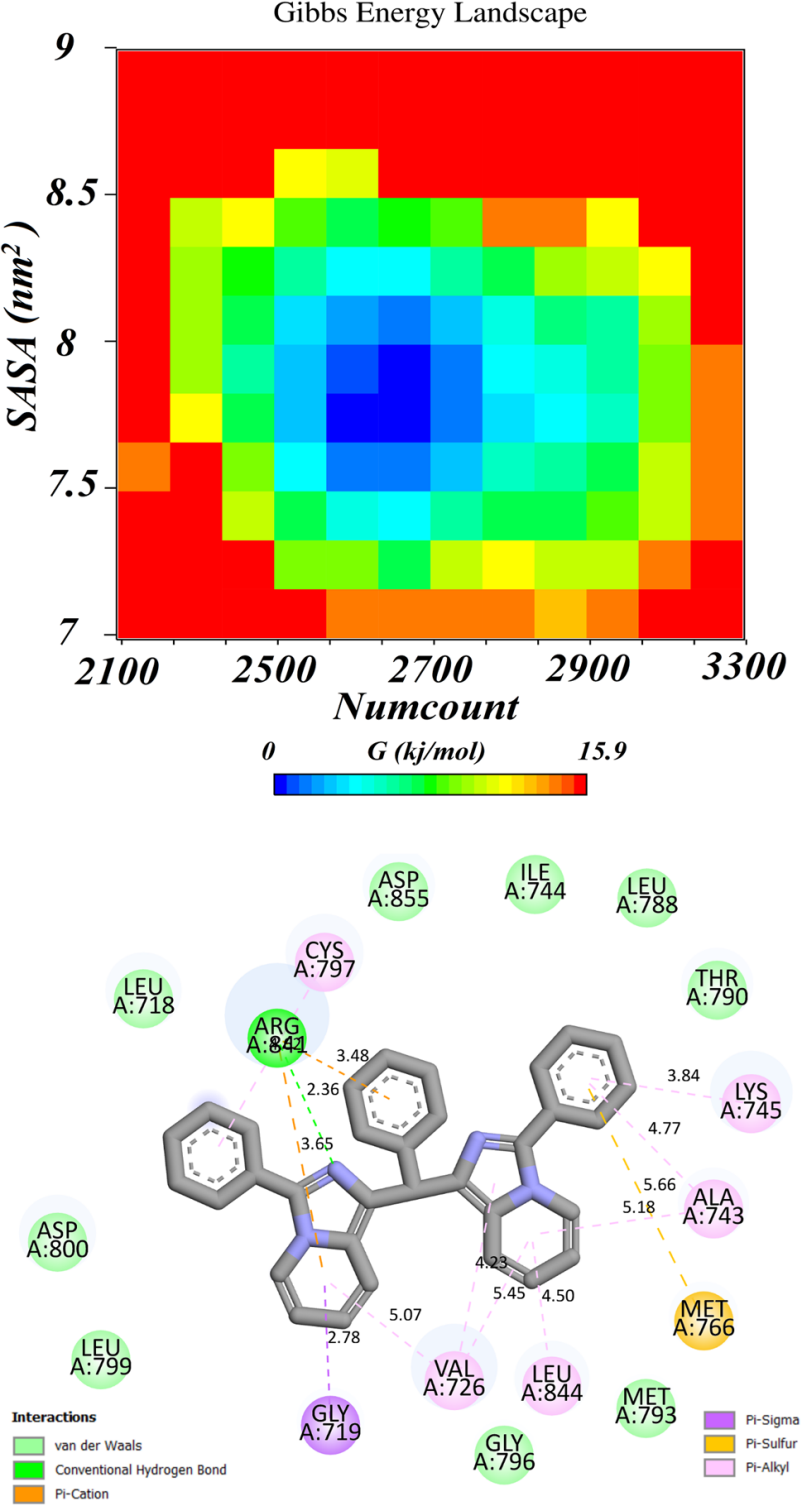
Free energy landscape (FES) obtained from the last 50 ns of 100 ns MD simulation of **3a**-EGFR complex (upper panel). Number of contacts and solvent accessible surface area are used as the PC1 and PC2 dimension, respectively. Interactive picture between **1-3a** and residues surrounding in the ATP binding region (lower panel).

As discussed above, the compound**s** denoted as **3-3c 10-3j** and **15-4d** are now ejected for further computation. We now consider 15 complexes of our synthetic ligands (Table 1) and the EGFR protein. In each system, ~ 5000 snapshots are generated from the last 50 ns of each 100 ns MD simulation. The upper panel of Fig. 4 presents a typical example of the free energy landscape for compound **1-3a** which is constructed from the number of contact (PC1) and the solvent accessible surface area (PC2). A representative ligand-EGFR structure is selected from the global minima. All other compounds are plotted and given in the SI file. As for information, these figures are plotted by the Discovery Studio Visualizer^57^ (version 3.0) which can reveal various types of chemical bonding the complex formed by **1-3a** with EGFR residues. There is one hydrogen bond with **ARG 841.** Besides that, thirteen π tacking bonds are recognized in Fig. 4—lower.

In compound **8-3 h** (Fig. S39 in SI file), two hydrogen bonds with **ARG 841**–**LYS 745**, four halogen bonds with **ALA 743–LEU 788–MET 793** and eleven π tacking bonds are identified. Table 3 inserts into account the total number of the bonds and the number of EGFR amino acids bound to our 15 novel ligands. Interactive pictures can be viewed in the SI file, Figs. S33–S49 (SI file).

**Table 3:** Bond type and number of bonding: π-tacking, hydrogen and halogen bonds.

STT	Ref	Number of pi tacking	Number of hydrogen bond	Amino acid in forming hydrogen bond	Number of halogen bond	Amino acid in forming halogen bond
1	**3a**	13	1	ARG841		
2	**3b**	7				
4	**3d**	14				
5	**3e**	7				
6	**3f**	8			1	ASP800
7	**3g**	11	1	THR79O	1	ASP800
8	**3h**	14	2	ARG841– LYS745	4	ALA747–LEU788– MET793
9	**3i**	7	1	ARG842		
11	**3k**	9	1	*ARG 841*		
12	**4a**	4				
13	**4b**	9				
14	**4c**	6	1	GLY721		
16	**4f**	6				
17	**4g**	10				
18	**4h**	9	1	LYS745		

Data are obtained from representative conformations of 15 compounds considered (**3c, 3j** and **4d** are neglected).

### SMD method measuring the strength of ligand–EGFR interaction

The conventional steered molecular dynamics simulation (SMD) is an effective tool to evaluate the binding affinities of small compounds to different receptors. A larger rupture force and/or a higher pulling work and/or a larger value of activation barrier are all expected to reveal a complex which is characterized by a strong binding affinity between the ligand and receptor. Table 4 lists the average values and corresponding error margins of the quantities obtained after 100 independent SMD simulation orbits. The rupture force, the external work and especially the high of Hummer-Szabo unbinding barrier are computed following a protocol described in our previous publication^56^. Figures S50–S52 in the Supporting Information illustrates the evolutions of the rupture force, pulling work and unbinding barrier obtained from independent SMD trajectories.

**Table 4:** Numerical results achieved from 100 independent trajectories of SMD simulation: mean of rupture force, mean of external pulling work and free energy barrier.

N	Ref	Rupture force (pN)	Pulling work (kcal/mol)	Unbinding barrier (kcal/mol)
1	3a	966.6 ± 12	147.5 ± 1.9	64.3 ± 1.5
2	3b	758.5 ± 11.8	102.1 ± 1.4	36.5 ± 1.2
4	3d	611.5 ± 7.7	78.9 ± 1	25.3 ± 0.7
5	3e	466.2 ± 4.6	64.9 ± 0.8	13.4 ± 0.4
6	3f	737.6 ± 8.1	109.9 ± 1.3	35.2 ± 0.9
7	3g	803.1 ± 10.3	123.7 ± 1.4	37.2 ± 1.3
8	3h	862.3 ± 9.4	117.8 ± 1.4	50.4 ± 1.2
9	3i	509.5 ± 6.8	50.7 ± 0.7	14.2 ± 0.4
11	3k	530.8 ± 6.7	64.8 ± 0.9	17.9 ± 0.5
12	4a	597 ± 7	81.1 ± 1.1	21.7 ± 0.6
13	4b	495.2 ± 6.1	71 ± 1.1	14.1 ± 0.4
14	4c	599.2 ± 7.6	83 ± 1.2	19.1 ± 0.6
16	4f	512.2 ± 8.7	64.4 ± 0.9	18.3 ± 0.7
17	4g	766.8 ± 8.1	106 ± 1.2	33.7 ± 1.1
18	4h	638.2 ± 7.6	102.1 ± 1.3	23.6 ± 0.7
19	1m17	717.7 ± 13.4	100.1 ± 3.9	31.1 ± 1.2
20	4zau	445.4 ± 10.1	56.1 ± 1.5	12.7 ± 0.7

The 1M17 and 4ZAU are also tested.

It is straightforward to recognize that there is a group containing six compounds **1, 2, 6, 7, 8** and **17** having higher values of rupture force, work and barrier than the corresponding values of the known erlotinib and Osimertinib drugs. We thus classify these compounds into a high inhibitory group (I). In comparison to the two used drugs, the group (I) compounds are characterized by higher binding affinities. According to obtained data, the remaining nine compounds belong to the low inhibitory group (II). Compound **1-3a** possesses the highest averaged force, being 966 pN, while **7-3g** and **8-3h** also give inspired results of 803 and 862 pN, respectively. Overall, the three compounds **1, 7** and** 8** share the three top rank positions not only in the rupture force but also in the external work and unbinding barrier. In terms of absolute values, the highest value of work of 147.5 kcal/mol also belongs to **1-3a** and two values of 123.7 and 117.8 kcal/mol are the external work obtained for both **7-3g** and **8-3h**, respectively. Both the rupture force and pulling work present the strength of ligand–receptor interaction. A larger value indicates a harder breaking of the ligand–receptor complex.

We can also extracted the Hummer–Szabo unbinding barrier which characterizes the translation of a macro molecular system^58^. Recently more evidence proved that the unbinding rate correlates better with the drug’s efficacy than the binding free energy^59–61^. When a ligand moves in or out of the receptor binding active site, it has to cross an energy barrier. Thus, a higher value of an unbinding barrier tends to prevent the system from translating from a bound to an unbound state. Compounds **1-3a, 7-3g** and **8-3h** are calculated to have unbinding barriers of 64.3, 37.2 and 50.4 kcal/mol, respectively, that are significantly higher than those of the rest of compounds. Here let us recognize that the three compounds **1, 7** and** 8** emerge to have strong binding affinities with the EGFR protein, even better than the erlotinib and Osimertinib drugs.

**Figure 5:**
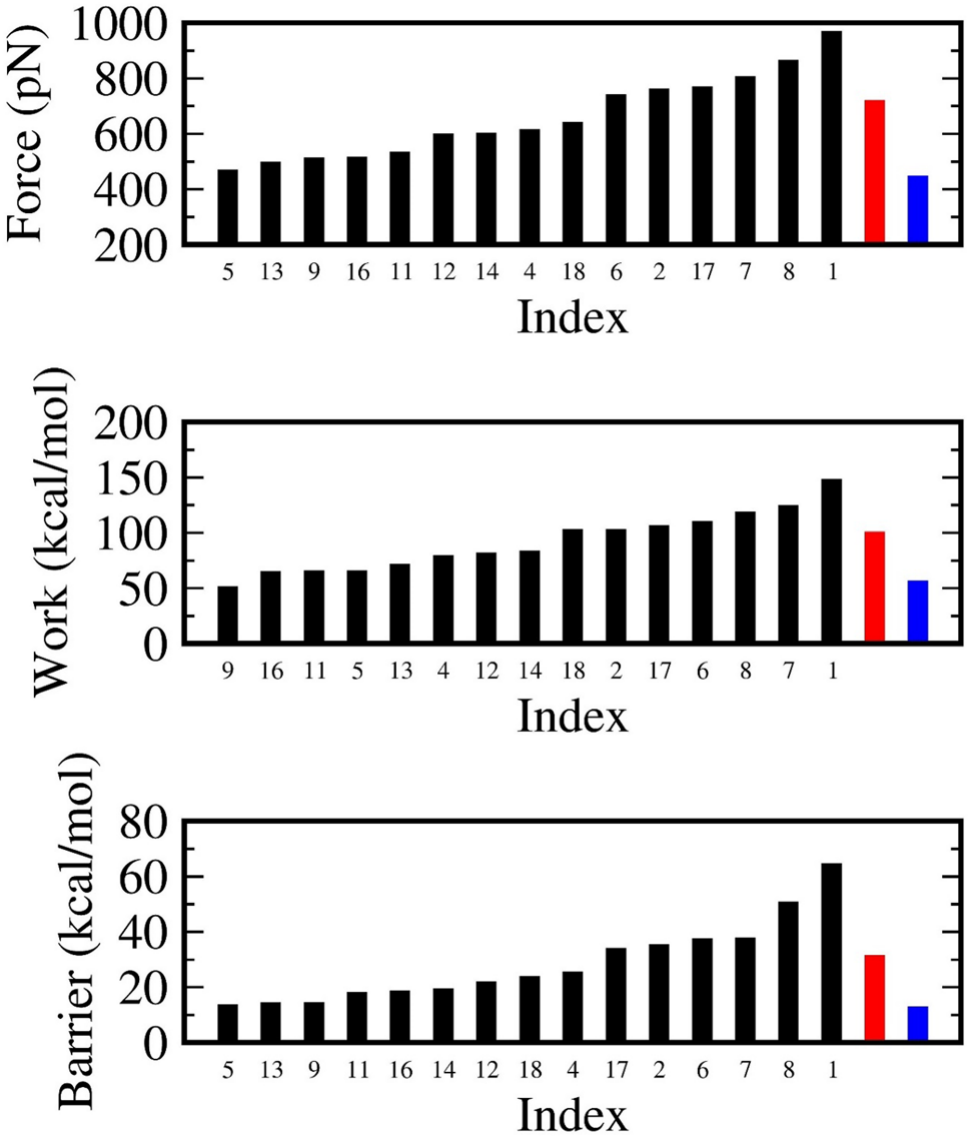
Measurement of rupture force (upper panel), pulling work (center) and barrier barrier (lower) can effectively rank the ligand–protein binding affinity. Mean values (shown in black bar) are computed from 100 independent trajectories of pulling for 15 ligands considered (in black), along with erlotinib 1M17 (in red) and osimertinib 4ZAU (in blue) out of the EGFR binding region.

Other compounds in group (I) including **2, 6** and **17** have lesser prospect when they in all cases reach the lowest values as seen in Fig. 5 below. On this basis, the three compounds **1, 7** and** 8** could reasonably be selected into the next step of PMF treatment.

### Absolute binding free energy from umbrella sampling method verifies the inhibitor-EGFR binding affinity

**Figure 6:**
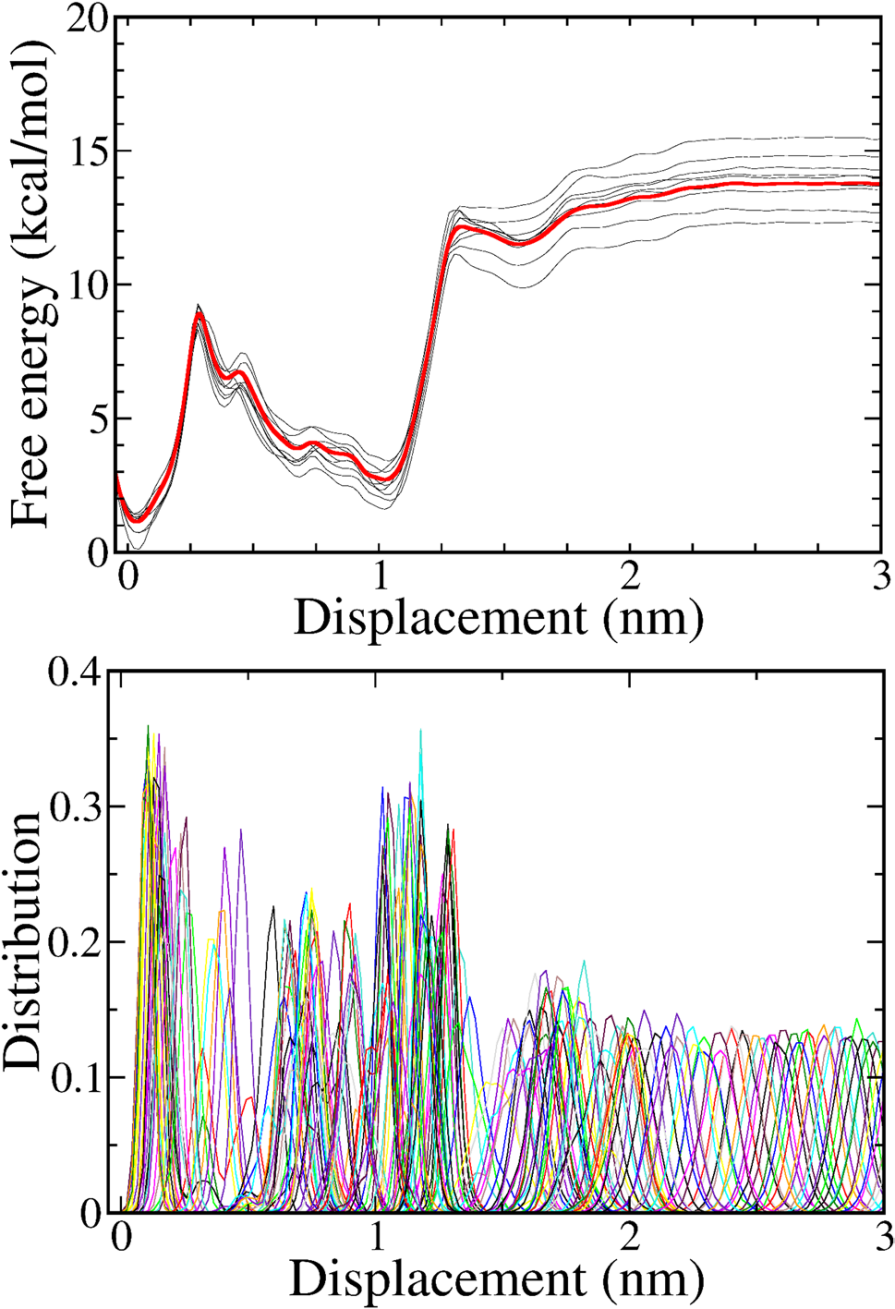
Potential of mean force (upper) and diagram (lower) are computed from 150 windows of umbrella sampling method. Data was collected in case of complex: compound **1-3a** and **EGFR**.

Briefly, the SMD simulation results discussed above allow us to identify a group of three promising compounds **1**, **7** and **8**. Notably, in this final ranking step, it appears necessary to evaluate the absolute binding free energy for two well-known drugs, the erlotinib and Osimertinib. Calculated results for these two drugs are used as a calibration to verify the potentiality of the three novel compounds **1**, **7** and **8**. For this purpose, the *gmx wham* from Gromacs package helps us to reconstruct the PMF curve of data generated by the US method. Figure 6 illustrates the PMF curve of the system containing compound **1-3a** and EGFR tyrosine kinase domain. Results for other systems (compound 3g, 4h, erlotinib, Osimertinib) are given in Figs. S53–S56 (SI file), respectively. With the computed values of − 9.1 kcal/mol for erlotinib and − 8.7 kcal/mol for Osimertinib, the binding free energies extracted from simulations are in good agreement with available experimental. In fact, experimental study pointed out that Osimertinib was proved to have a weaker binding to the EGFR WT than erlotinib.

From such a good agreement on the absolute free binding energy, we now have a reliable reference to evaluate the binding affinities of three novel compounds. Indeed, **1-3a** emerges as the compound bearing the highest free binding energy of − 12.8 kcal/mol, significantly larger than those of erlotinib and Osimertinib. This result encourages us to consider compound **1-3a** as the best promising candidate for an EGFR inhibition. Other compounds **7-3g** and **8-3h** have values of − 11.7 and − 10.6 kcal/mol, respectively, thus also possess comparable results to erlotinib and Osimertinib. The results listed in Table 5 also confirm that three compounds in this final round exhibit a high potential to become the prospective anti-cancer drug. Of course, the bioactivities of these candidate molecules need to be extensively explored in further studies to clarify their effectiveness.

**Table 5:** Binding free energy extracted by the umbrella sampling method for three compounds **1**, **7** and **8** and two known drugs erlotinib (1M17) and Osimertinib (4ZAU) in bound state with EGFR protein.

Compound	ΔG^cal^ (PMF) (kcal/mol)
1–3a	− 12.8
7–3g	− 11.7
8–3h	− 10.6
Erlotinib	− 9.1
Osimertinib	− 8.7

### Distinguished interaction between chemical sub-groups and key residues of EGFR

**Figure 7:**
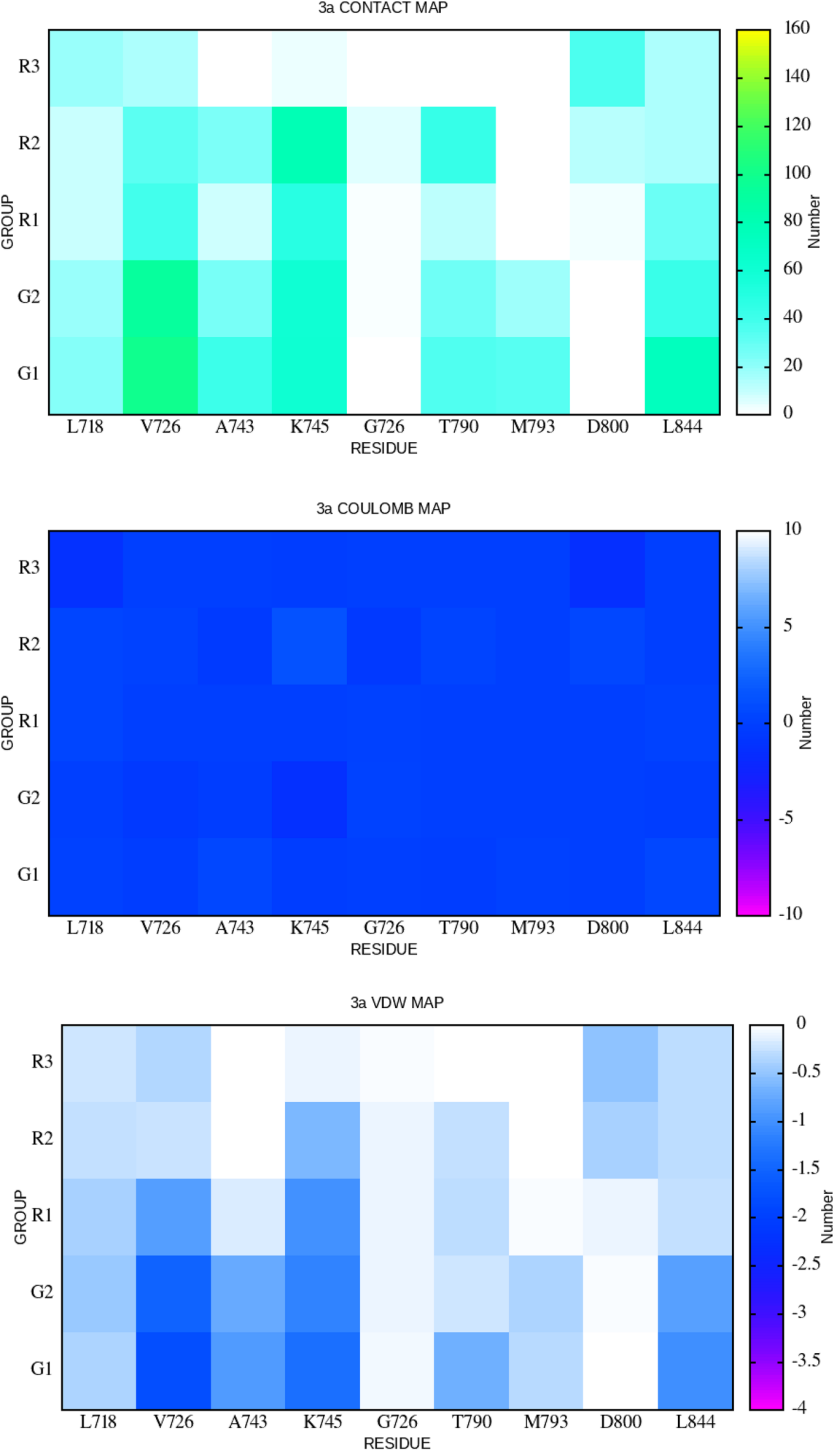
The contact map (upper), Coulomb interaction map (mediate) and Vdw interaction map (lower) between 5 sub-groups of compound **1-3a** and 9 key residues of EGFR tyrosine domain.

It has been shown^62^ that the seven residues L718, V726, A743, , T790, M793, D800 and L844 that are located in the EGFR active site, would keep an important role in the protein–ligand interaction. By adding more K745 and G762 residues into the cavity, which form the protein salt-bridge, we can generate an analysis of the contact map, the Coulomb and the Vdw interaction map between the nine key residues and five chemical sub-groups. Figure 7 shows the analysis of **1-3a**, whereas those for the other compounds can be viewed in Figs. S57–S59 (SI file).

In compound **1-3a**, both groups **G1** and **G2** represent an attraction from V726 when averaging numbers 99.6 and 92.6 of contacts per frame are collected, respectively (data are shown in Table S3, SI file). These contacts could be caused in part by a high Vdw potential. According to Table S4 (SI file), these amount to − 1.8 and − 1.5 kcal/mol between **G1** and **G2** and the residue **V726** (plot in Fig. 7), respectively. A smaller value of 79.4 for the number of contacts per frame is also formed by the **R2** subgroup of compound **1-3a** and the residue **K745**. Surprisingly, these contacts are related to a high value of Coulomb positive potential (+ 1.4 kcal/mol) between **R2** of compound **1-3a** and **K745** as shown in Fig. 7 and list in Table S5 (SI file). Full data of contacts, Coulomb and Vdw potential are listed in Tables S3–S5 (SI file). Overall, in looking at all values of Coulomb potential, we notice some significant results created in case of **K745–R3f** **2-3b** (− 9.0 kcal/mol) and **K745–R2** of **6-4f** (− 7.8 kcal/mol). Aside from these, the lowest values of Vdw potential belongs to **K745–R1** of compound **5-3e** (− 2.4 kcal/mol), **L718–G2** of **7-3g** (− 3.3 kcal/mol), **L718–R2** of **8-3h** (− 2.2 kcal/mol) and **K745–R3** of **18-4h** (− 2.4 kcal/mol). Since **K745** usually contributes to many significant results, it raises a necessary study to deeply understand the role of **K745** (beside others residues) in the association of EGFR protein-ImPy derivates. A thorough knowledge of this lacking should be covered in an upcoming study, when more data will be generated, and other common EGFR mutants will also be taken into consideration.

## Concluding remarks

In this theoretical study, we demonstrated how a realistic protocol of computations allows us to detect the potential inhibitors. The strategy developed in this study aims to discover some promising compounds to be considered in the further steps of both in vivo and trial clinical testing. We have combined computational techniques as follows: (i) a docking method for creating an initial configuration; (ii) MD simulations for exploring the best binding mode, and (iii) two methods, a fast-growth SMD method and a high accuracy umbrella sampling method, applied to evaluate the ligand–protein binding affinity.

Although this is an approach that has been used in the screening of novel compounds, the number of simulations we have sampled in this study is large enough to yield calculated results that can be trusted. Since the number of compounds in this study is not too large, we have considered each of them and carefully compared them one by one. In the case where there is a much larger number of chemicals to be screened, development of directives for selection is required in such research.

When considering a series of 18 compounds that we have recently prepared experimentally, our present theoretical study allowed us to neglect some compounds on the basis of docking results, in particular when a compound could not generate a suitable initial structure. The compound which can pass our computational protocol emerges as a promising candidate.

Based on theoretical evidence such as the high rupture force or the large binding free energy, three compounds **1-3a, 7-3g** and **8-3h** were found to be inspired as efficient inhibitors in a treatment against the EGFR.

In addition, two significantly interesting points have emerged. The first one concerns the the very small averaging distances between the compound**s 8-3h** and the three residues M793, C797 and R841 of the EGFR protein (being 0.18, 0.16, 0.15 nm, respectively). These distances are short enough for a covalent bond to appear. The second point is the similar curves of free energy profiles when the compound **1-3a** and erlotinib (in 1M17) translate from a bound state to an unbound counterpart. A double transition state is identified in either the non-equilibrium or the equilibrium free energy profile. In other words, the compound **1-3a** can also perform bioactivities similar to those of erlotinib. This information is valuable for following studies in the field of bioinformatics.

Our computational results suggested a concrete direction for carrying out further experimental investigations on the biochemical activities of three new and promising imidazole[1,5-a]pyridine derivatives toward inhibition of EGFR enzyme for the development of EGFR targeting agents. Although interaction with the active site of EGFR enzyme is not strong, the remaining compounds can still be screened for activities toward the popular targets in targeted cancer therapy such as the PI3K/AKT/mTOR, VEGFR. Research toward this direction is being pursued in our laboratories.

### Supplementary Information

Supplementary Information.

## Data Availability

Supplementary Information (ESI): Computed results are given in the Electronic Supplementary Information file (SI, 100 pages). Tables and Figures contain the results of molecular dynamics simulations. As the Input files are too big to be given in the ESI file, they are available from the corresponding author on request.
