# Supplementary material for: Imidazole[1,5-a]pyridine derivatives as EGFR tyrosine kinase inhibitors unraveled by umbrella sampling and steered molecular dynamics simulations
Source: Sci Rep. 2024 May 28;14:12218. doi: 10.1038/s41598-024-62743-3 (PMC11133355; doi:10.1038/s41598-024-62743-3)
Supplement: Supplementary file 1 — Supplementary Information. [file 41598_2024_62743_MOESM1_ESM.pdf]

Supplementary Information (SI)

Imidazole[1,5-a]pyridine Derivatives as EGFR Tyrosine Kinase Inhibitors Unraveled by Umbrella Sampling and Steered Molecular Dynamics Simulations

Duc Toan Truong,[1,2] Kiet Ho,[3] Huynh Thi Yen Nhi,[1] Van Ha Nguyen,[4]
Tuan Thanh Dang,[4] Minh Tho Nguyen[1,2,*]

[1] Laboratory for Chemical Computation and Modeling, Institute for Computational Science and Artificial Intelligence, Van Lang University, Ho Chi Minh City, 70000 Vietnam
Email: minhtho.nguyen@vlu.edu.vn
[2] Faculty of Applied Technology, School of Technology, Van Lang University, Ho Chi Minh City, 70000 Vietnam
[3] Institute for Computational Science and Technology (ICST), Quang Trung software city, Ho Chi Minh City, 70000 Vietnam
[4] Faculty of Chemistry, VNU-Hanoi University of Science, 19 Le Thanh Tong, Hanoi, Vietnam

Table S1: Parameters of 20 compounds considered from DFT calculations. (B3LYP/6-31+G(d,p)).

No.	Compound	E_{HOMO}	E_{LUMO}	E_{gap}	IP	EA	χ	η	μ	S	ω
1	3a	-5.09	-1.35	3.74	5.09	1.35	3.22	1.87	-3.22	0.54	2.78
2	3b	-4.98	-1.27	3.71	4.98	1.27	3.12	1.85	-3.12	0.54	2.63
3	3c	-4.87	-1.22	3.65	4.87	1.22	3.04	1.82	-3.04	0.55	2.54
4	3d	-5.4	-1.5	3.9	5.4	1.5	3.45	1.95	-3.45	0.51	3.05
5	3e	-5.4	-1.53	3.87	5.4	1.53	3.46	1.94	-3.46	0.52	3.09
6	3f	-5.29	-1.55	3.74	5.29	1.55	3.42	1.87	-3.42	0.54	3.12
7	3g	-5.36	-1.44	3.92	5.36	1.44	3.4	1.96	-3.4	0.51	2.95
8	3h	-5.35	-1.52	3.83	5.35	1.52	3.43	1.92	-3.43	0.52	3.08
9	3i	-5.05	-1.46	3.59	5.05	1.46	3.25	1.8	-3.25	0.56	2.95
10	3j	-5.05	-1.13	3.93	5.05	1.13	3.09	1.96	-3.09	0.51	2.43
11	3k	-4.94	-1.03	3.91	4.94	1.03	2.99	1.96	-2.99	0.51	2.28
12	4a	-5.18	-1.45	3.73	5.18	1.45	3.32	1.87	-3.32	0.54	2.95

13	4b	-5.05	-1.37	3.68	5.05	1.37	3.21	1.84	-3.21	0.54	2.81
14	4c	-5.07	-1.35	3.72	5.07	1.35	3.21	1.86	-3.21	0.54	2.77
15	4d	-5.08	-1.4	3.68	5.08	1.4	3.24	1.84	-3.24	0.54	2.85
16	4f	-5.13	-1.29	3.84	5.13	1.29	3.21	1.92	-3.21	0.52	2.68
17	4g	-5.07	-1.38	3.69	5.07	1.38	3.22	1.85	-3.22	0.54	2.81
18	4h	-5.07	-1.33	3.74	5.07	1.33	3.2	1.87	-3.2	0.54	2.73
19	Erlotinib	-5.74	-1.48	4.26	5.74	1.48	3.61	2.13	-3.61	0.47	3.06
20	Osimertinib	-5.11	-1.26	3.85	5.11	1.26	3.19	1.93	-3.19	0.52	2.64

Table S2: The mean of min distance (nm) obtained from the last 20ns of 100ns MD simulation.

Index	Ref	**721GLY**	**722ALA**	**745LYS**	**790THR**	**791GLN**	**792LEU**	**793MET**	**797CYS**	**841ARG**	**858LEU**
1	3a	0.268 ± 0.001	0.521 ± 0.001	0.26 ± 0.001	0.252 ± 0.001	0.519 ± 0.001	0.419 ± 0.001	0.294 ± 0.001	0.256 ± 0.001	0.222 ± 0	0.774 ± 0.001
2	3b	0.498 ± 0.001	0.569 ± 0.002	0.227 ± 0.003	0.806 ± 0.002	0.947 ± 0.002	0.757 ± 0.002	0.75 ± 0.002	0.283 ± 0.001	0.234 ± 0.001	0.549 ± 0.001
3	***3c***	***1.074 ± 0.004***	***0.69 ± 0.004***	***1.549 ± 0.005***	***2.364 ± 0.005***	***2.643 ± 0.004***	***2.571 ± 0.004***	***2.42 ± 0.003***	***1.614 ± 0.002***	***0.995 ± 0.003***	***0.919 ± 0.002***
4	3d	0.227 ± 0.001	0.453 ± 0.001	0.274 ± 0.001	0.278 ± 0.001	0.369 ± 0.001	0.256 ± 0.001	0.191 ± 0.001	0.221 ± 0.001	0.359 ± 0.001	0.979 ± 0.001
5	3e	0.241 ± 0.001	0.189 ± 0.001	0.271 ± 0.001	0.817 ± 0.002	0.985 ± 0.002	0.784 ± 0.002	0.817 ± 0.002	0.551 ± 0.001	0.272 ± 0.001	0.552 ± 0.002
6	3f	0.599 ± 0.001	0.515 ± 0.002	0.267 ± 0.001	0.353 ± 0.002	0.691 ± 0.002	0.557 ± 0.002	0.562 ± 0.002	0.282 ± 0.002	0.21 ± 0	0.643 ± 0.002
7	3g	0.276 ± 0.001	0.259 ± 0.001	0.366 ± 0.001	0.293 ± 0.001	0.527 ± 0.002	0.482 ± 0.001	0.401 ± 0.002	0.286 ± 0.001	0.264 ± 0.001	0.808 ± 0.002
8	3h	0.591 ± 0.001	0.796 ± 0.001	0.201 ± 0.001	0.253 ± 0.001	0.306 ± 0.001	0.241 ± 0.001	0.183 ± 0.001	0.166 ± 0.001	0.154 ± 0	0.95 ± 0.001
9	3i	0.313 ± 0.003	0.262 ± 0.003	0.196 ± 0.001	0.824 ± 0.002	1.024 ± 0.002	0.772 ± 0.002	0.813 ± 0.002	0.39 ± 0.004	0.256 ± 0.001	0.732 ± 0.002
10	***3j***	***0.229 ± 0.001***	***0.359 ± 0.003***	***0.565 ± 0.005***	***1.026 ± 0.003***	***1.07 ± 0.003***	***0.639 ± 0.004***	***0.619 ± 0.004***	***0.29 ± 0.002***	***0.562 ± 0.002***	***1.168 ± 0.002***
11	3k	0.269 ± 0.001	0.27 ± 0.002	0.547 ± 0.003	0.838 ± 0.001	0.879 ± 0.001	0.611 ± 0.001	0.546 ± 0.001	0.233 ± 0.001	0.225 ± 0.001	1.004 ± 0.002
12	4a	0.268 ± 0.001	0.449 ± 0.002	0.305 ± 0.001	0.65 ± 0.003	0.654 ± 0.002	0.519 ± 0.002	0.445 ± 0.002	0.348 ± 0.001	0.2 ± 0.001	0.9 ± 0.002
13	4b	0.236 ± 0.001	0.24 ± 0.001	0.384 ± 0.003	0.858 ± 0.002	1.104 ± 0.002	0.957 ± 0.002	0.956 ± 0.002	0.601 ± 0.002	0.165 ± 0.001	0.425 ± 0.003
14	4c	0.258 ± 0.001	0.388 ± 0.002	0.299 ± 0.002	0.564 ± 0.002	0.609 ± 0.002	0.431 ± 0.002	0.357 ± 0.001	0.298 ± 0.001	0.272 ± 0.001	0.756 ± 0.002
15	***4d***	***0.627 ± 0.002***	***0.378 ± 0.002***	***1.123 ± 0***	***1.767 ± 0.001***	***2.035 ± 0.002***	***1.97 ± 0.002***	***1.793 ± 0.001***	***1.088 ± 0.001***	***0.406 ± 0.001***	***0.712 ± 0.002***
16	4f	0.332 ± 0.003	0.361 ± 0.004	0.557 ± 0.004	1.033 ± 0.004	1.267 ± 0.004	1.096 ± 0.004	1.05 ± 0.003	0.586 ± 0.002	0.254 ± 0.001	0.495 ± 0.002
17	4g	0.433 ± 0.003	0.549 ± 0.002	0.241 ± 0.001	0.69 ± 0.004	0.791 ± 0.004	0.634 ± 0.003	0.513 ± 0.004	0.286 ± 0.002	0.199 ± 0	0.786 ± 0.001
18	4h	0.304 ± 0.002	0.462 ± 0.002	0.232 ± 0.001	0.378 ± 0.004	0.565 ± 0.002	0.507 ± 0.003	0.435 ± 0.002	0.283 ± 0.001	0.222 ± 0.001	0.786 ± 0.001

Table S3: The data of contact map formed by 9 key residues and 5 functional groups of a compound. Averaging number of contact per frame are obtained from 5.000 frames of last 50ns MD simulation.

**1**	**Compound 3a**	**L718**	**V726**	**A743**	**K745**	**G762**	**T790**	**M793**	**D800**	**L844**
	**G1**	22.7	99.7	41.1	61.7	0.3	35.1	33.3	0	74.2
	**G2**	18.5	92.6	25.7	60.9	1.4	27.4	17.2	0	42.4
	**R1**	9.4	39.5	7.9	48.2	0.9	11.3	0	2.4	28.7
	**R2**	8.9	32.6	23.9	79.4	5.6	43.2	0	12.6	14.1
	**R3**	18.4	14.4	0	3.5	0	0	0	36.2	13.8
**2**	**Compound 3b**	**L718**	**V726**	**A743**	**K745**	**G762**	**T790**	**M793**	**D800**	**L844**
	**G1**	0	1.5	0	16	0.8	0	0	0.9	3.9
	**G2**	0	0	0	0	0	0	0	17.4	3.2
	**R1**	55.7	82	0.8	5.2	0	0	0	0.5	6
	**R2**	0	0	0	0	0	0	0	0	0
	**R3**	0	8.6	0	70.2	13.6	0	0	0	0.1
**4**	**Compound 3d**	**L718**	**V726**	**A743**	**K745**	**G762**	**T790**	**M793**	**D800**	**L844**
	**G1**	93.8	55	69.9	2.1	0	21.3	92.5	0	132.5
	**G2**	29.7	64.5	0	0.1	0	0	0	0	16.5
	**R1**	85.4	12.6	0.1	0	0	0	0.2	37.4	23.8
	**R2**	21.6	23.6	0	0	0	0	0	0.1	0
	**R3**	10.4	72.7	53.4	122.5	8.9	63.1	0.1	0	39.7
**5**	**Compound 3e**	**L718**	**V726**	**A743**	**K745**	**G762**	**T790**	**M793**	**D800**	**L844**
	**G1**	35.8	99.9	2.1	56.2	0.6	0.3	0	0.3	13.4
	**G2**	0	1.7	0	0.6	0	0	0	0	0
	**R1**	0	48.8	0	122.3	5.7	0	0	0	0
	**R2**	3.1	3.7	0	0	0	0	0	15.9	2.7
	**R3**	0	0	0	0	0	0	0	0	0
**6**	**Compound 3f**	**L718**	**V726**	**A743**	**K745**	**G762**	**T790**	**M793**	**D800**	**L844**
	**G1**	37.5	73.4	4.3	12.9	0	0.4	6.6	7.1	77
	**G2**	0	2.8	0	12.6	0	0	0	0	7.5
	**R1**	2.7	3.8	0	0	0	0	0	11.8	1.7
	**R2**	7.9	70.3	25.3	125.4	5.5	32.5	1.6	0	70.7
	**R3**	0	0	0	14.8	1.2	0	0	0	0
**7**	**Compound 3g**	**L718**	**V726**	**A743**	**K745**	**G762**	**T790**	**M793**	**D800**	**L844**
	**G1**	1.3	23.4	0	4.1	0	0	0	0.1	16.2
	**G2**	159.1	72.5	12.3	3.4	0	0.1	33.9	1.2	90.2
	**R1**	22.6	8.8	0	0	0	0	0	29	3.7
	**R2**	27.2	72.8	40.7	95.2	1.2	45.6	7.2	0	89.2
	**R3**	0	12.5	0	3.4	0	0	0	0	0
**8**	**Compound 3h**	**L718**	**V726**	**A743**	**K745**	**G762**	**T790**	**M793**	**D800**	**L844**
	**G1**	74.3	15.5	1.5	0	0	0	2.5	48.4	40.5

	**G2**	61.5	62.6	82.7	12.8	0	48.2	114.8	0	129.9
	**R1**	35.9	90.5	0	3	0	0	0	0	22
	**R2**	140.7	0.1	1.9	0	0	0	18.5	0.2	2.7
	**R3**	2.6	53.4	39.3	169.9	12.6	74.4	0.9	0	27.7
**9**	**Compound 3i**	**L718**	**V726**	**A743**	**K745**	**G762**	**T790**	**M793**	**D800**	**L844**
	**G1**	0	0	0	0	0	0	0	0	0
	**G2**	49.6	76.5	0.3	4.4	0	0	0	0.8	2.9
	**R1**	0	0	0	0	0	0	0	7.6	0.1
	**R2**	0	0	0	0	0	0	0	0	0
	**R3**	0	25.3	0	70.9	6.1	0	0	0	0.1
**11**	**Compound 3k**	**L718**	**V726**	**A743**	**K745**	**G762**	**T790**	**M793**	**D800**	**L844**
	**G1**	49	4.1	0	0	0	0	0	1.1	0
	**G2**	112.6	73.2	2	0.5	0	0	5.3	10.8	56.5
	**R1**	2.3	41.7	0	10.9	0	0	0	0	0
	**R2**	2.7	0.1	0	0	0	0	0	0	0
	**R3**	0.2	0	0	0	0	0	0	106	1.7
**12**	**Compound 4a**	**L718**	**V726**	**A743**	**K745**	**G762**	**T790**	**M793**	**D800**	**L844**
	**G1**	0.1	0.5	0	0	0	0	0	0	0
	**G2**	52	36.9	0	0.2	0	0	0	14.4	0.8
	**R1**	1.3	79.1	0	52.6	0.1	0	0	0	0
	**R2**	0	0	0	0	0	0	0	0.7	0
	**R3**	92.4	91.1	13.4	2.6	0	0.6	11.7	0.5	71.1
**13**	**Compound 4b**	**L718**	**V726**	**A743**	**K745**	**G762**	**T790**	**M793**	**D800**	**L844**
	**G1**	12.2	7.4	0	0	0	0	0	14.3	1
	**G2**	3.7	27.9	0	8.5	0	0	0	0	30.1
	**R1**	0	4.7	0	6.5	0	0	0	0	0
	**R2**	12.5	0.3	0	0	0	0	0	0	0
	**R3**	53.2	86	26.8	43.4	0.5	9.9	12.6	0.1	58.1
**14**	**Compound 4c**	**L718**	**V726**	**A743**	**K745**	**G762**	**T790**	**M793**	**D800**	**L844**
	**G1**	8.8	13.3	0	0	0	0	0	13.2	0.4
	**G2**	9.7	51.2	0	10.8	0	0	0.1	0	51
	**R1**	0	27.4	0	47	0.6	0	0	0	0
	**R2**	8.4	0.2	0	0	0	0	0	0	0
	**R3**	114.9	67.9	26.4	5	0	3.8	34.1	1.9	91.9
**16**	**Compound 4f**	**L718**	**V726**	**A743**	**K745**	**G762**	**T790**	**M793**	**D800**	**L844**
	**G1**	30.3	40.8	0.6	0.9	0	0	0.1	1.3	7.1
	**G2**	0	0.2	0	2.8	0.1	0	0	0	0
	**R1**	0.9	0.1	0	0	0	0	0	3.4	0
	**R2**	4.2	47.2	0	52.9	4	0	0	1.1	6.7
	**R3**	0	0	0	0	0	0	0	0	0
**17**	**Compound 4g**	**L718**	**V726**	**A743**	**K745**	**G762**	**T790**	**M793**	**D800**	**L844**
	**G1**	0.1	22.8	0	36.6	0.9	0	0	0	4.9

	**G2**	4.6	7.2	0	0	0	0	0	4	0.1
	**R1**	93.2	98.4	7.2	5.8	0	0.7	11.4	7.5	83
	**R2**	0	50.5	0	140.2	1.2	0	0	0	0
	**R3**	0	0	0	0	0	0	0	51.6	0.1
**18**	**Compound 4h**	**L718**	**V726**	**A743**	**K745**	**G762**	**T790**	**M793**	**D800**	**L844**
	**G1**	0.7	36.6	0	22.3	0	0	0	2.1	19.3
	**G2**	25.8	53.1	54.2	29.4	0	44.6	62.9	5.5	108.9
	**R1**	118.6	69.3	4.8	2.4	0	0	3.3	6	14.3
	**R2**	1.6	76.8	0	97	3.5	0	0	0	0
	**R3**	9.3	48.1	34.2	164.9	18.7	64.5	2.5	0	33.2

Table S4: The data of Coulomb interaction map formed by 9 key residues and 5 functional groups of a compound. Averaging value of Coulomb potential per frame are obtained from 5.000 frames of last 50ns MD simulation

**1**	**Compound 3a**	**L718**	**V726**	**A743**	**K745**	**G762**	**T790**	**M793**	**D800**	**L844**
	**G1**	0.11	-0.19	0.61	-0.23	0.04	-0.12	0.11	0	0.55
	**G2**	-0.06	-0.57	-0.12	-1.28	0.27	-0.07	0	0	-0.24
	**R1**	0.37	-0.06	0.03	-0.03	0.06	0.01	0	0.06	0.15
	**R2**	0.37	0.2	-0.39	1.37	-0.55	0.35	0	0.62	-0.04
	**R3**	-1.27	0.04	0	-0.11	0.01	0	0	-1.33	-0.06
**2**	**Compound 3b**	**L718**	**V726**	**A743**	**K745**	**G762**	**T790**	**M793**	**D800**	**L844**
	**G1**	0	0.01	0	0.43	-0.1	0	0	0.01	0.01
	**G2**	0	0	0	0	0	0	0	-0.45	0
	**R1**	-0.14	-0.12	0	-0.21	0	0	0	0	-0.01
	**R2**	0	0	0	0	0	0	0	0	0
	**R3**	0	-0.03	0	-9.03	0.93	0	0	0	0
**4**	**Compound 3d**	**L718**	**V726**	**A743**	**K745**	**G762**	**T790**	**M793**	**D800**	**L844**
	**G1**	0.06	-0.02	0.09	0	0	0.01	0.01	0	0.13
	**G2**	-0.46	-0.03	0	-0.02	0	0	0	0.03	0.02
	**R1**	0.03	0.02	0	0	0	0	0	-0.75	0.04
	**R2**	1.29	0.05	0	0	0	0	0	0.04	0
	**R3**	-0.01	0.07	-0.35	0.85	-0.68	0.23	0	0	0.2
**5**	**Compound 3e**	**L718**	**V726**	**A743**	**K745**	**G762**	**T790**	**M793**	**D800**	**L844**
	**G1**	-0.08	-0.02	0	-1.4	0.05	0	0	-0.01	0
	**G2**	0	0	0	0	0	0	0	0	0
	**R1**	0	-0.01	0	0.58	-0.33	0	0	0	0
	**R2**	-0.06	0.01	0	0	0	0	0	-0.25	0
	**R3**	0	0	0	0	0	0	0	0	0
**6**	**Compound 3f**	**L718**	**V726**	**A743**	**K745**	**G762**	**T790**	**M793**	**D800**	**L844**
	**G1**	-0.09	-0.17	0.01	0.02	0	0	-0.01	-0.04	-0.01
	**G2**	0	-0.01	0	0.07	-0.04	0	0	0	0.03
	**R1**	-0.01	-0.01	0	0	0	0	0	-0.7	-0.01
	**R2**	0.03	0.28	0.07	-0.12	0.07	-0.11	0	0	0.03
	**R3**	0	0	0	0.6	-0.13	0	0	0	0
**7**	**Compound 3g**	**L718**	**V726**	**A743**	**K745**	**G762**	**T790**	**M793**	**D800**	**L844**
	**G1**	0.02	0	0	0.09	0	0	0	-0.02	0.08
	**G2**	-1.09	-0.22	-0.05	-0.04	0	0.01	-0.29	-0.06	-0.25
	**R1**	-2.69	0.03	0	0	0	0	0	-2.87	0.02
	**R2**	0.1	0.24	0.36	-1.09	0.29	-0.54	0.03	0	0.18
	**R3**	0	0.02	0	0.03	0	0	0	0	0

8	Compound 3h	L718	V726	A743	K745	G762	T790	M793	D800	L844
	G1	-0.02	0	0	0	0	0	-0.03	-1.52	-0.06
	G2	-0.15	-0.01	0.04	0.06	0	0.05	-1.61	0	-0.16
	R1	0.08	-0.06	0	0.02	0	0	0	0	-0.14
	R2	0.02	0	0.01	0	0	0	-0.75	-0.04	0.02
	R3	-0.02	-0.08	-0.97	-2.94	1.56	0.04	0	0	0.05
**9**	**Compound 3i**	**L718**	**V726**	**A743**	**K745**	**G762**	**T790**	**M793**	**D800**	**L844**
	G1	0	0	0	0	0	0	0	0	0
	G2	-0.04	-0.15	0	-0.01	0	0	0	-0.02	-0.01
	R1	0.01	0	0	0	0	0	0	-0.31	0
	R2	0	0	0	0	0	0	0	0	0
	R3	0	0.03	0	-2.82	0.28	0	0	0	0
**11**	**Compound 3k**	**L718**	**V726**	**A743**	**K745**	**G762**	**T790**	**M793**	**D800**	**L844**
	G1	-4.84	-0.01	0	0	0	0	0	-0.07	0
	G2	0.24	-0.12	-0.01	0	0	0	0	-0.04	0.01
	R1	-0.02	-0.09	0	-0.02	0	0	0	0	0
	R2	-0.01	0	0	0	0	0	0	0	0
	R3	-0.01	0	0	0	0	0	0	-1.97	0
**12**	**Compound 4a**	**L718**	**V726**	**A743**	**K745**	**G762**	**T790**	**M793**	**D800**	**L844**
	G1	0.01	0	0	0	0	0	0	0	0
	G2	-2.48	-0.07	0	0	0	0	0	-1.64	0
	R1	0.02	-0.05	0	-0.87	0.02	0	0	0	0
	R2	0	0	0	0	0	0	0	-0.04	0
	R3	-0.23	0.01	0	0.05	0	0	0	-0.02	0.18
**13**	**Compound 4b**	**L718**	**V726**	**A743**	**K745**	**G762**	**T790**	**M793**	**D800**	**L844**
	G1	0.01	-0.03	0	0	0	0	0	-0.33	0
	G2	0.01	-0.08	0	0.01	-0.02	0	0	0	0.17
	R1	0	0	0	0.06	0	0	0	0	0
	R2	-0.02	0	0	0	0	0	0	0	0
	R3	-0.02	0.06	0.03	0.31	-0.03	-0.04	-0.03	-0.01	0.04
**14**	**Compound 4c**	**L718**	**V726**	**A743**	**K745**	**G762**	**T790**	**M793**	**D800**	**L844**
	G1	0.05	-0.06	0	0	0	0	0	-0.35	0
	G2	0.03	-0.23	0	0.13	-0.02	0	0	0	0.4
	R1	0	-0.01	0	0.65	-0.03	0	0	0	0
	R2	-0.06	0	0	0	0	0	0	0	0
	R3	-0.57	0.09	0.03	0.04	0	0	-0.17	-0.17	0.02
**16**	**Compound 4f**	**L718**	**V726**	**A743**	**K745**	**G762**	**T790**	**M793**	**D800**	**L844**
	G1	-0.1	-0.06	0	-0.01	0	0	0	-0.01	-0.01
	G2	0	0	0	0.16	-0.03	0	0	0	0
	R1	-0.05	0	0	0	0	0	0	-0.3	0

	**R2**	0.18	-0.15	0	-7.75	0.64	0	0	0.16	0
	**R3**	0	0	0	0	0	0	0	0	0
**17**	**Compound 4g**	**L718**	**V726**	**A743**	**K745**	**G762**	**T790**	**M793**	**D800**	**L844**
	**G1**	0	-0.04	0	-0.24	-0.05	0	0	0	0
	**G2**	-0.22	0.01	0	0	0	0	0	0.28	0
	**R1**	-0.19	0.12	0	-0.01	0	0	0	-0.12	0.06
	**R2**	0	-0.03	0	-3.11	0.05	0	0	0	0
	**R3**	0	0	0	0	0	0	0	-0.5	0
**18**	**Compound 4h**	**L718**	**V726**	**A743**	**K745**	**G762**	**T790**	**M793**	**D800**	**L844**
	**G1**	0.01	-0.12	0	-0.94	0	0	0	0	0.01
	**G2**	0.03	-0.29	0.17	-0.55	0.01	-2.25	0.3	-0.54	0.18
	**R1**	-0.34	0.09	0.02	0.01	0	0	-0.01	-0.14	0.02
	**R2**	0.01	-0.1	0	-1.83	-0.04	0	0	0	0
	**R3**	0	-0.06	-0.02	-0.14	-1.04	0.03	0	0	0

Table S5: The data of Vdw interaction map formed by 9 key residues and 5 functional groups of a compound. Averaging value of Vdw potential per frame are obtained from 5.000 frames of last 50ns MD simulation

**1**	**Compound 3a**	**L718**	**V726**	**A743**	**K745**	**G762**	**T790**	**M793**	**D800**	**L844**
	**G1**	-0.36	-1.8	-0.89	-1.37	-0.05	-0.68	-0.29	-0.01	-1.02
	**G2**	-0.46	-1.51	-0.73	-1.13	-0.08	-0.21	-0.36	-0.02	-0.83
	**R1**	-0.37	-0.86	-0.15	-0.98	-0.06	-0.28	-0.01	-0.08	-0.25
	**R2**	-0.26	-0.23	0.08	-0.61	-0.06	-0.25	-0.01	-0.38	-0.29
	**R3**	-0.2	-0.32	0	-0.08	-0.01	0	0	-0.5	-0.28
**2**	**Compound 3b**	**L718**	**V726**	**A743**	**K745**	**G762**	**T790**	**M793**	**D800**	**L844**
	**G1**	-0.01	-0.09	0	-0.21	-0.06	0	0	-0.02	-0.08
	**G2**	-0.01	-0.01	0	0	0	0	0	-0.42	-0.04
	**R1**	-0.96	-1.04	-0.02	-0.11	0	0	-0.01	-0.04	-0.09
	**R2**	0	0	0	0	0	0	0	-0.01	0
	**R3**	0	-0.12	0	0.14	-0.32	0	0	0	-0.01
**4**	**Compound 3d**	**L718**	**V726**	**A743**	**K745**	**G762**	**T790**	**M793**	**D800**	**L844**
	**G1**	-1.61	-1.23	-1.27	-0.15	0	-0.35	-1.5	-0.01	-2.66
	**G2**	-1.09	-1.31	-0.01	-0.07	0	0	0	-0.09	-0.22
	**R1**	-1.1	-0.2	-0.02	0	0	0	-0.1	-0.95	-0.35
	**R2**	-0.8	-0.4	0	0	0	0	0	-0.04	0
	**R3**	-0.15	-1.22	-0.99	-1.89	-0.41	-1.21	-0.07	0	-0.54

**5**	**Compound 3e**	**L718**	**V726**	**A743**	**K745**	**G762**	**T790**	**M793**	**D800**	**L844**
	**G1**	-0.64	-1.56	-0.03	-0.76	-0.03	-0.01	-0.01	-0.03	-0.17
	**G2**	0	-0.05	0	-0.05	-0.01	0	0	0	0
	**R1**	-0.01	-0.8	0	-2.43	-0.26	0	0	0	0
	**R2**	-0.17	-0.11	0	-0.01	0	0	0	-0.52	-0.04
	**R3**	0	0	0	0	0	0	0	0	0
**6**	**Compound 3f**	**L718**	**V726**	**A743**	**K745**	**G762**	**T790**	**M793**	**D800**	**L844**
	**G1**	-0.72	-1.41	-0.06	-0.29	-0.01	-0.01	-0.1	-0.17	-1.11
	**G2**	0	-0.1	0	-0.2	-0.04	0	0	0	-0.1
	**R1**	-0.07	-0.07	0	-0.01	0	0	0	-0.28	-0.04
	**R2**	-0.11	-0.92	-0.34	-1.73	-0.16	-0.43	-0.09	0	-1.03
	**R3**	0	-0.01	0	-0.14	-0.05	0	0	0	0
**7**	**Compound 3g**	**L718**	**V726**	**A743**	**K745**	**G762**	**T790**	**M793**	**D800**	**L844**
	**G1**	-0.13	-0.54	0	-0.17	-0.01	0	0	-0.03	-0.23
	**G2**	-3.28	-1.56	-0.18	-0.17	0	-0.03	-0.56	-0.08	-1.65
	**R1**	-0.38	-0.17	0	0	0	0	0	-0.12	-0.07
	**R2**	-0.45	-0.94	-0.49	-1.22	-0.06	-0.6	-0.21	0	-1.4
	**R3**	-0.01	-0.17	0	-0.08	0	0	0	0	0
**8**	**Compound 3h**	**L718**	**V726**	**A743**	**K745**	**G762**	**T790**	**M793**	**D800**	**L844**
	**G1**	-1.68	-0.37	-0.06	-0.01	0	0	-0.2	-0.89	-0.62
	**G2**	-0.92	-1.15	-1.51	-0.37	-0.01	-0.8	-1.77	0	-2.27
	**R1**	-0.77	-1.59	-0.02	-0.18	0	0	-0.01	-0.01	-0.32
	**R2**	-2.2	-0.06	-0.06	0	0	0	-0.62	-0.04	-0.09
	**R3**	-0.08	-0.86	-0.56	-2.83	-0.32	-1.34	-0.08	0	-0.36
**9**	**Compound 3i**	**L718**	**V726**	**A743**	**K745**	**G762**	**T790**	**M793**	**D800**	**L844**
	**G1**	0	-0.01	0	0	0	0	0	0	0
	**G2**	-1.01	-1.11	-0.01	-0.12	0	0	0	-0.05	-0.09
	**R1**	-0.01	0	0	0	0	0	0	-0.17	-0.01
	**R2**	0	0	0	0	0	0	0	0	0
	**R3**	-0.03	-0.6	-0.01	-0.83	-0.14	0	0	0	-0.04
**11**	**Compound 3k**	**L718**	**V726**	**A743**	**K745**	**G762**	**T790**	**M793**	**D800**	**L844**
	**G1**	-1.05	-0.2	0	0	0	0	0	-0.06	0
	**G2**	-1.96	-1.2	-0.04	-0.05	0	0	-0.09	-0.22	-0.59
	**R1**	-0.09	-0.55	0	-0.14	0	0	0	0	0
	**R2**	-0.08	-0.02	0	0	0	0	0	0	0
	**R3**	-0.04	-0.01	0	0	0	0	0	-2.05	-0.05
**12**	**Compound 4a**	**L718**	**V726**	**A743**	**K745**	**G762**	**T790**	**M793**	**D800**	**L844**
	**G1**	-0.04	-0.05	0	0	0	0	0	-0.02	0
	**G2**	-1.45	-0.87	0	-0.03	0	0	0	-0.41	-0.05

	**R1**	-0.08	-1.23	0	-0.44	-0.05	0	0	0	0
	**R2**	0	0	0	0	0	0	0	-0.04	0
	**R3**	-1.37	-1.45	-0.16	-0.1	0	-0.03	-0.17	-0.05	-0.92
**13**	**Compound 4b**	**L718**	**V726**	**A743**	**K745**	**G762**	**T790**	**M793**	**D800**	**L844**
	**G1**	-0.52	-0.2	0	-0.01	0	0	0	-0.34	-0.02
	**G2**	-0.16	-0.62	-0.01	-0.21	-0.03	0	-0.01	-0.01	-0.4
	**R1**	-0.01	-0.07	0	-0.06	0	0	0	0	0
	**R2**	-0.37	-0.05	0	0	0	0	0	-0.01	0
	**R3**	-0.77	-1.31	-0.36	-0.52	-0.02	-0.14	-0.17	-0.01	-0.82
**14**	**Compound 4c**	**L718**	**V726**	**A743**	**K745**	**G762**	**T790**	**M793**	**D800**	**L844**
	**G1**	-0.32	-0.3	0	-0.01	0	0	0	-0.38	-0.02
	**G2**	-0.36	-1.16	-0.02	-0.26	-0.03	0	-0.02	-0.05	-0.62
	**R1**	-0.02	-0.4	0	-0.49	-0.03	0	0	0	0
	**R2**	-0.22	-0.06	0	0	0	0	0	-0.01	0
	**R3**	-1.94	-0.99	-0.32	-0.09	0	-0.05	-0.47	-0.08	-1.36
**16**	**Compound 4f**	**L718**	**V726**	**A743**	**K745**	**G762**	**T790**	**M793**	**D800**	**L844**
	**G1**	-0.51	-0.73	-0.01	-0.06	0	0	-0.01	-0.06	-0.09
	**G2**	0	-0.02	0	-0.04	-0.01	0	0	0	0
	**R1**	-0.04	-0.01	0	0	0	0	0	-0.06	0
	**R2**	-0.11	-0.62	0	-0.02	-0.09	-0.01	0	-0.05	-0.08
	**R3**	0	0	0	0	0	0	0	0	0
**17**	**Compound 4g**	**L718**	**V726**	**A743**	**K745**	**G762**	**T790**	**M793**	**D800**	**L844**
	**G1**	-0.04	-0.58	0	-0.54	-0.06	0	0	-0.01	-0.12
	**G2**	-0.17	-0.14	0	0	0	0	0	-0.18	-0.02
	**R1**	-1.11	-1.23	-0.07	-0.11	0	-0.02	-0.19	-0.15	-0.9
	**R2**	-0.01	-0.45	0	-1.76	-0.1	0	0	0	0
	**R3**	0	0	0	0	0	0	0	-0.98	-0.01
**18**	**Compound 4h**	**L718**	**V726**	**A743**	**K745**	**G762**	**T790**	**M793**	**D800**	**L844**
	**G1**	-0.1	-0.73	-0.01	-0.5	-0.01	0	-0.01	-0.06	-0.34
	**G2**	-0.53	-1.14	-0.98	-0.67	-0.02	-0.2	-1.03	-0.11	-1.65
	**R1**	-1.45	-0.62	-0.05	-0.05	0	0	-0.08	-0.15	-0.17
	**R2**	-0.07	-1.06	0	-1.2	-0.1	0	0	0	0
	**R3**	-0.12	-0.57	-0.55	-2.42	-0.38	-1.12	-0.07	0	-0.49

Table S6: Studies reported in the last 5 years applying a computational protocol to screen binding affinities between new drug compounds and EGFR tyrosine kinase domain. Screening novel inhibitors for both wide type and EGFR variants.

No.	Author, Year	EGFR-TK	Compound	Reference compound	Computational method		
					Force field, water model	Simulation time per trajectory	Method and Binding free energy of erlotinib or osimertinib to WT (kcal/mol)
1	Ashiru et al., 2023[1]	L858R/T790M/C797S (6LUD)	Mining from Pubchem database	Osimertinib	CHARMM36, TIP3P water model	100 ns	none
2	Pawara et al., 2021[2]	T790M (2JIU) and WT (4ZAU)	Mining from Zinc database	Osimertinib and AEE788	OPLS_2005, TIP3P water model	200 ns	MM-PBSA None computing
3	Kiriwan et al., 2021[3]	WT (1M17)	Tripeptide structures were iteratively generated	Erlotinib	AMBER99 SB-ILDN, TIP3P water model	100 ns	MM-PBSA
4	Yang et al., 2020[4]	T790R/L858R and WT	Small compounds M1 and T1	Osimertinib	AMBER 99SB, GAFF, TIP3P water model	40 ns and 60 ns	MM-PBSA

5	Karnik et al., 2020[5]	C797S (5D41, 4I23)	Mining from a database	EAI045	AMBER99, OPLS_2005, TIP3P	10 ns	none
6	Ahmad et al., 2020[6]	L858R/T790M/C797S (5XGN)	Mining from p38-alpha inhibitors	Osimertinib	OPLS_2005, TIP3P water model	100 ns	MM-PBSA
7	Maiti et al., 2020[7]	L858R (4LQM)	45 phyto-chemicals extracted from Vernonia cinerea	PD168393	CHARMM36, SPC/E water model	60 ns	Non- bond interaction energy
8	Singh et al., 2019[8]	L858R/T790M	Taken from Singh et al., 2017	Osimertinib	OPLS_2005, TIP3P water model	30 ns	MM-PBSA
9	Tinivella et al., 2018[9]	T790M and WT	EAI001 and EAI045	EAI005 (5D41)	AMBER, TIP3P water model	500 ns	MM-PBSA
10	Uchibori et al., 2017[10]	C797S/T790M/L858R	Brigatinib	Osimertinib and WZ4002	AMBER FF99SB-ILDN, TIP3P water model	500 ns	none

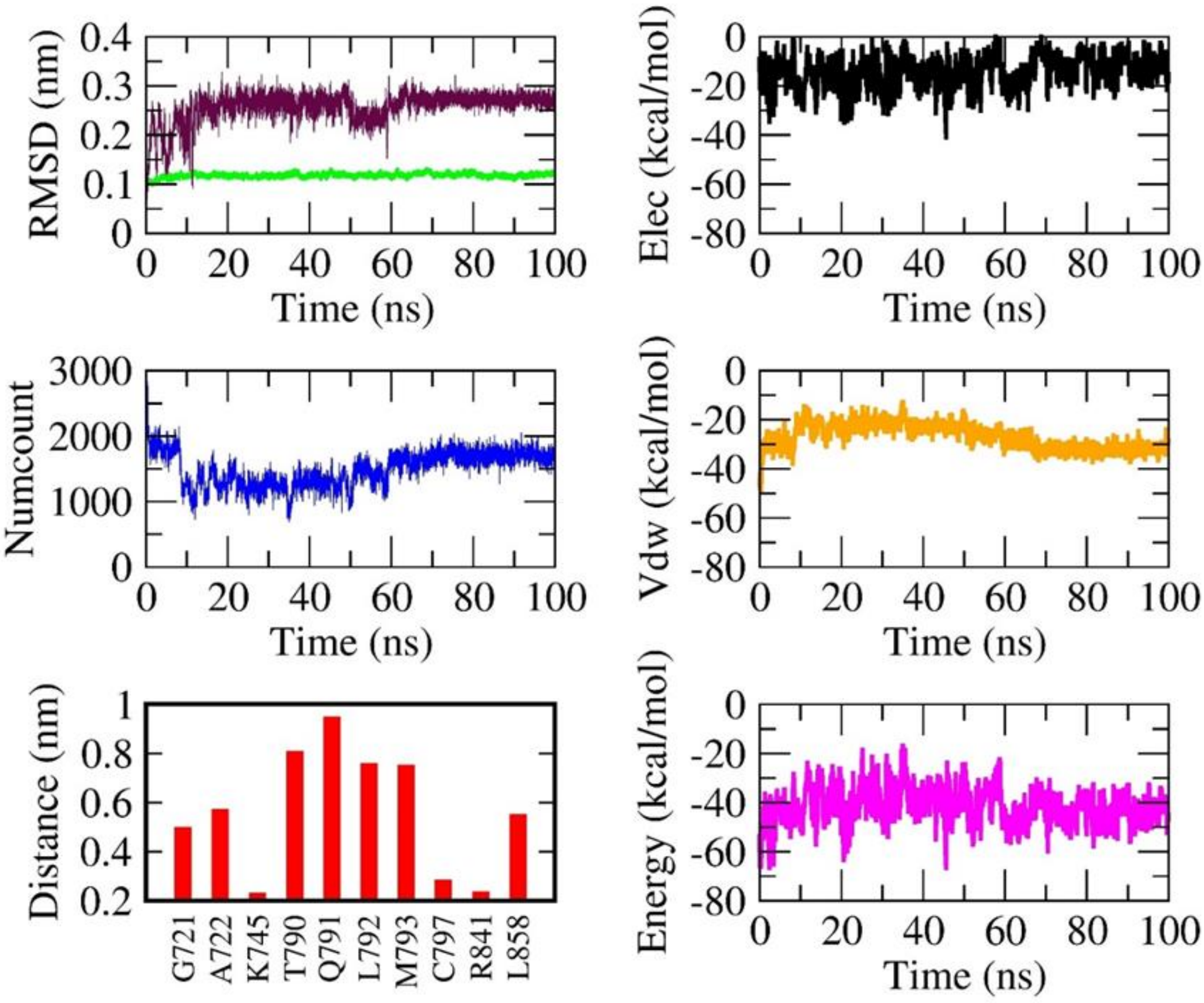

***Figure S1**: Data is obtained from 100ns MD simulation in case of **compound – 2** and EGFR complex. The time – dependent root mean square deviation of protein and ligand are plotted in green and maroon, respectively. The number of contact between **compound – 2** in the dependence of time is shown in blue. Non – bonded interaction energy (in magenta) is combined from the Coulomb potential energy (in black) and the Van de Waals potential energy (in orange). The mean of min distances from **compound – 2** to 10 key residues located in the ATP region are also shown in red bar.*

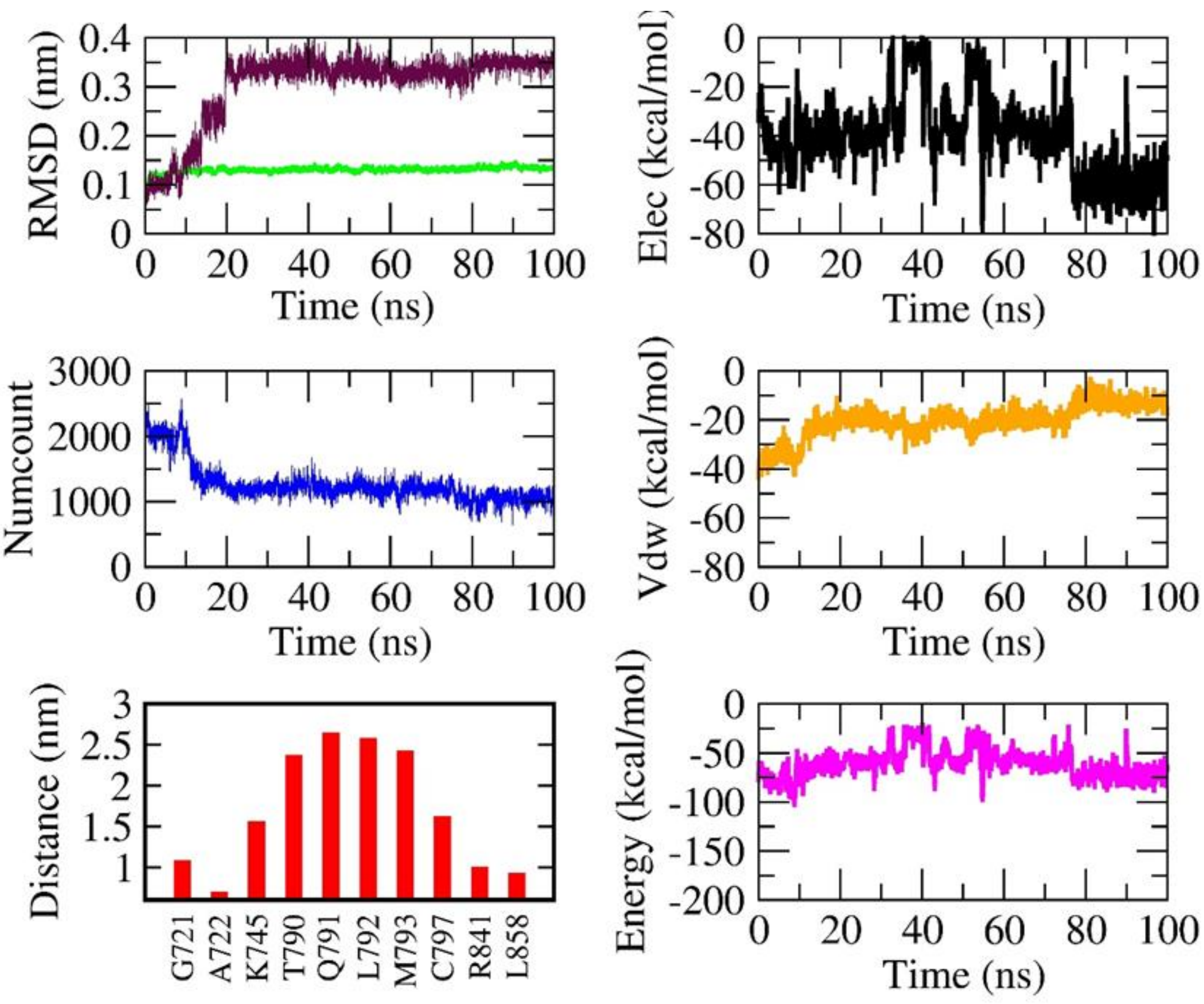

Figure S2*: Data is obtained from 100ns MD simulation in case of* ***compound – 3*** *and EGFR complex. The time – dependent root mean square deviation of protein and ligand are plotted in green and maroon, respectively. The number of contact between* ***compound – 3*** *in the dependence of time is shown in blue. Non – bonded interaction energy (in magenta) is combined from the Coulomb potential energy (in black) and the Van de Waals potential energy (in orange). The mean of min distances from* ***compound – 3*** *to 10 key residues located in the ATP region are also shown in red bar.*

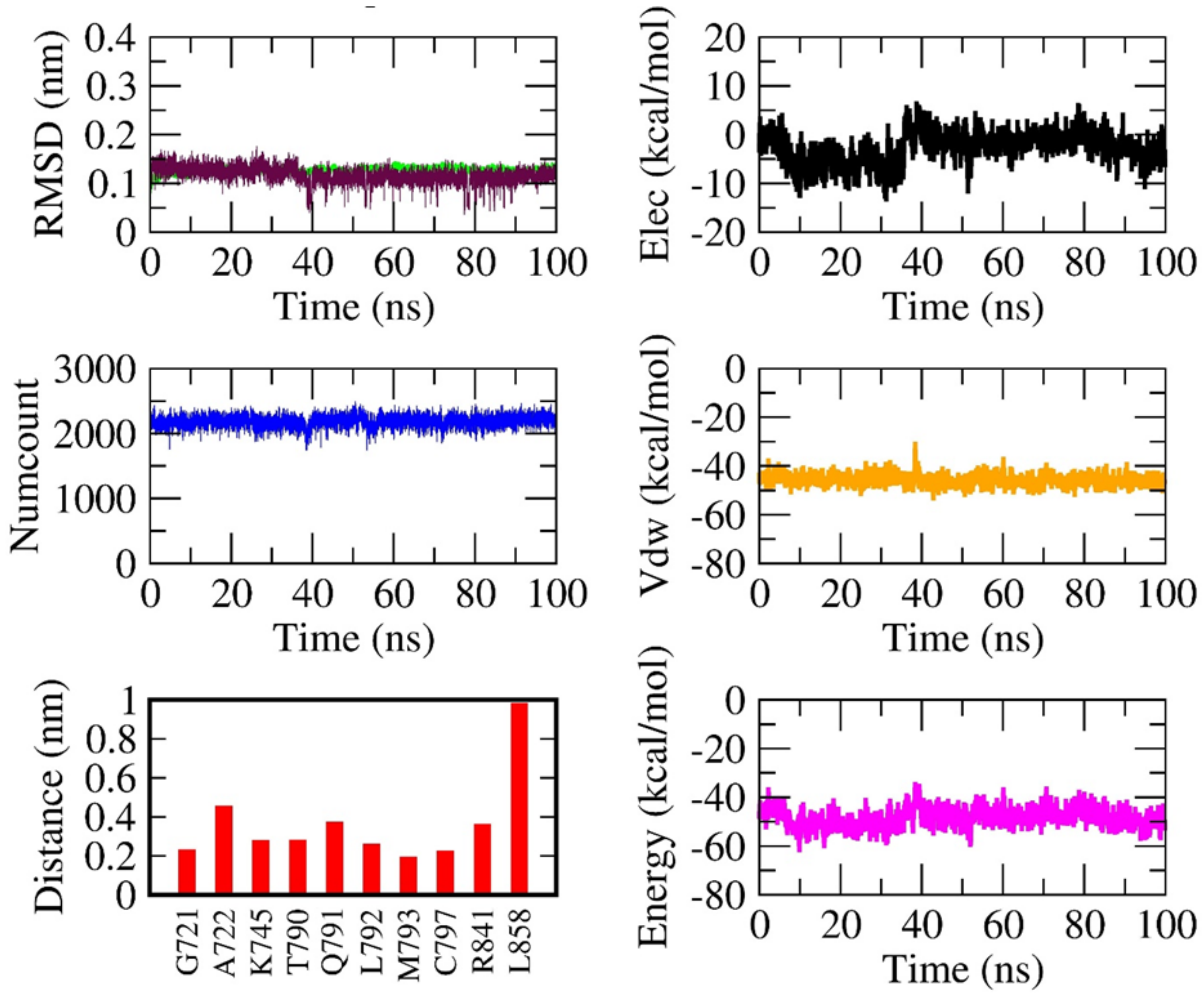

Figure S3: *Data is obtained from 100ns MD simulation in case of* ***compound – 4*** *and EGFR complex. The time – dependent root mean square deviation of protein and ligand are plotted in green and maroon, respectively. The number of contact between* ***compound – 4*** *in the dependence of time is shown in blue. Non – bonded interaction energy (in magenta) is combined from the Coulomb potential energy (in black) and the Van de Waals potential energy (in orange). The mean of min distances from* ***compound – 4*** *to 10 key residues located in the ATP region are also shown in red bar.*

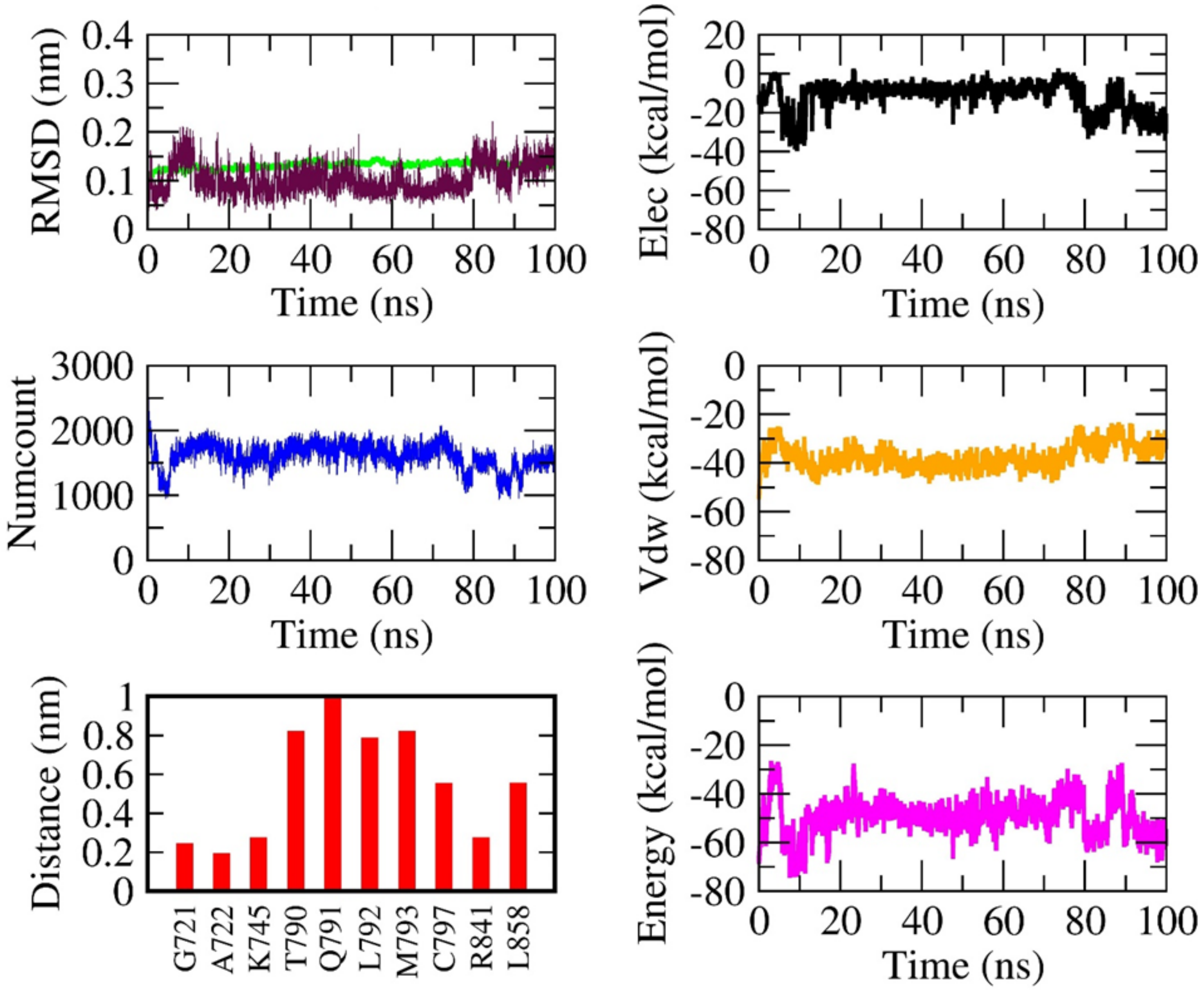

Figure S4: *Data is obtained from 100ns MD simulation in case of* ***compound – 5*** *and EGFR complex. The time – dependent root mean square deviation of protein and ligand are plotted in green and maroon, respectively. The number of contact between* ***compound – 5*** *in the dependence of time is shown in blue. Non – bonded interaction energy (in magenta) is combined from the Coulomb potential energy (in black) and the Van de Waals potential energy (in orange). The mean of min distances from* ***compound – 5*** *to 10 key residues located in the ATP region are also shown in red bar.*

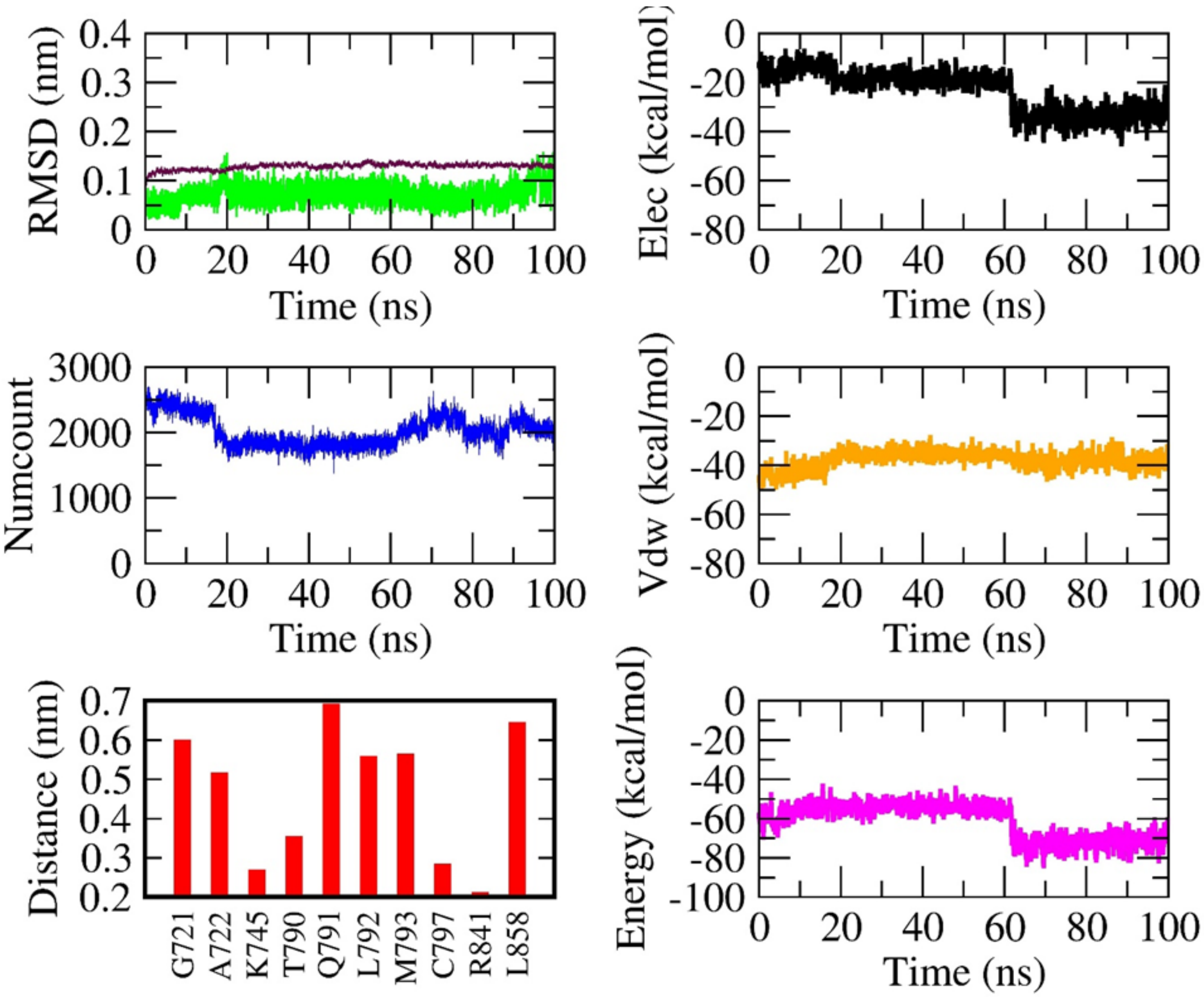

Figure S5: *Data is obtained from 100ns MD simulation in case of* ***compound – 6*** *and EGFR complex. The time – dependent root mean square deviation of protein and ligand are plotted in green and maroon, respectively. The number of contact between* ***compound – 6*** *in the dependence of time is shown in blue. Non – bonded interaction energy (in magenta) is combined from the Coulomb potential energy (in black) and the Van de Waals potential energy (in orange). The mean of min distances from* ***compound – 6*** *to 10 key residues located in the ATP region are also shown in red bar.*

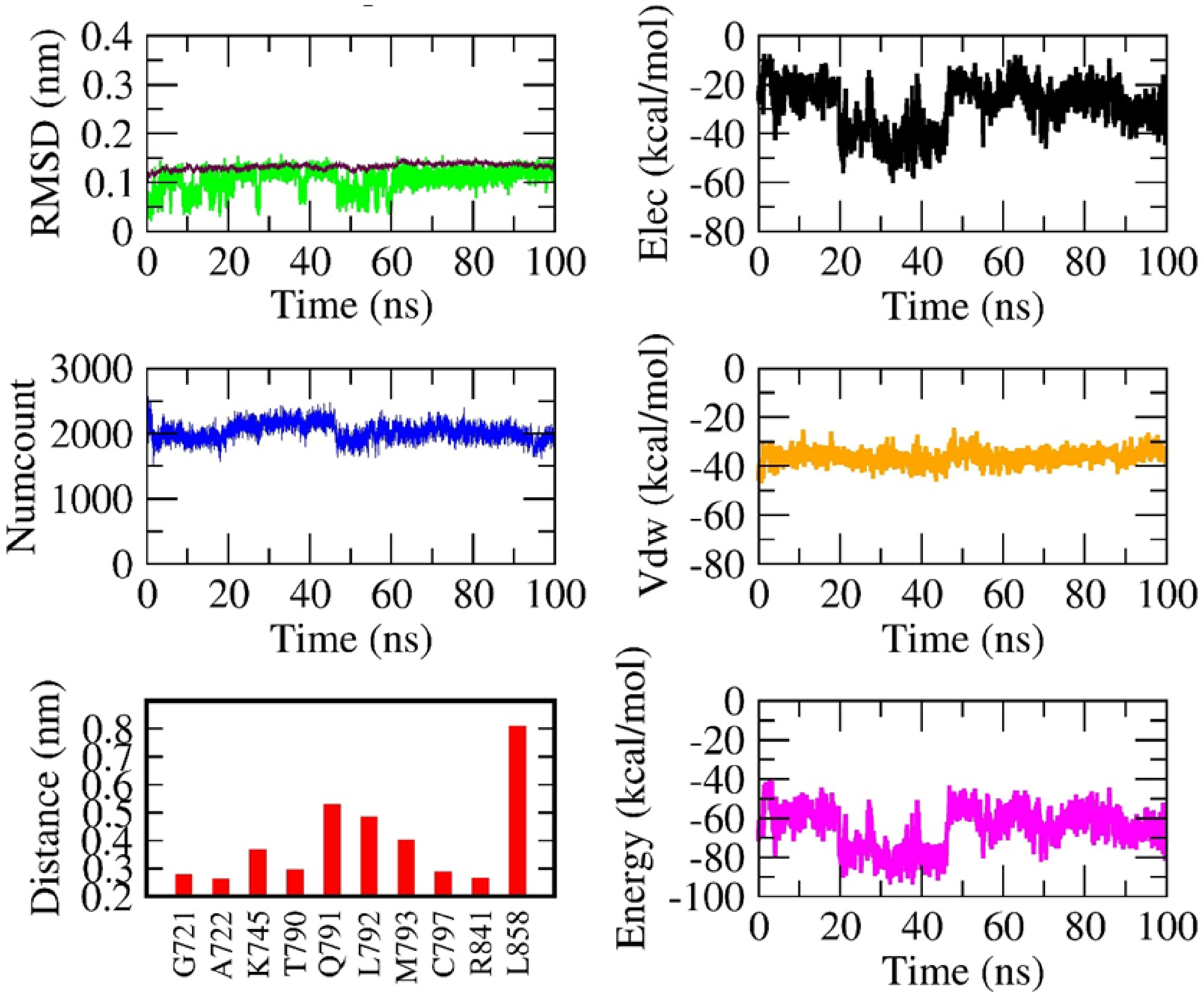

Figure S6: *Data is obtained from 100ns MD simulation in case of* ***compound – 7*** *and EGFR complex. The time – dependent root mean square deviation of protein and ligand are plotted in green and maroon, respectively. The number of contact between* ***compound – 7*** *in the dependence of time is shown in blue. Non – bonded interaction energy (in magenta) is combined from the Coulomb potential energy (in black) and the Van de Waals potential energy (in orange). The mean of min distances from* ***compound – 7*** *to 10 key residues located in the ATP region are also shown in red bar.*

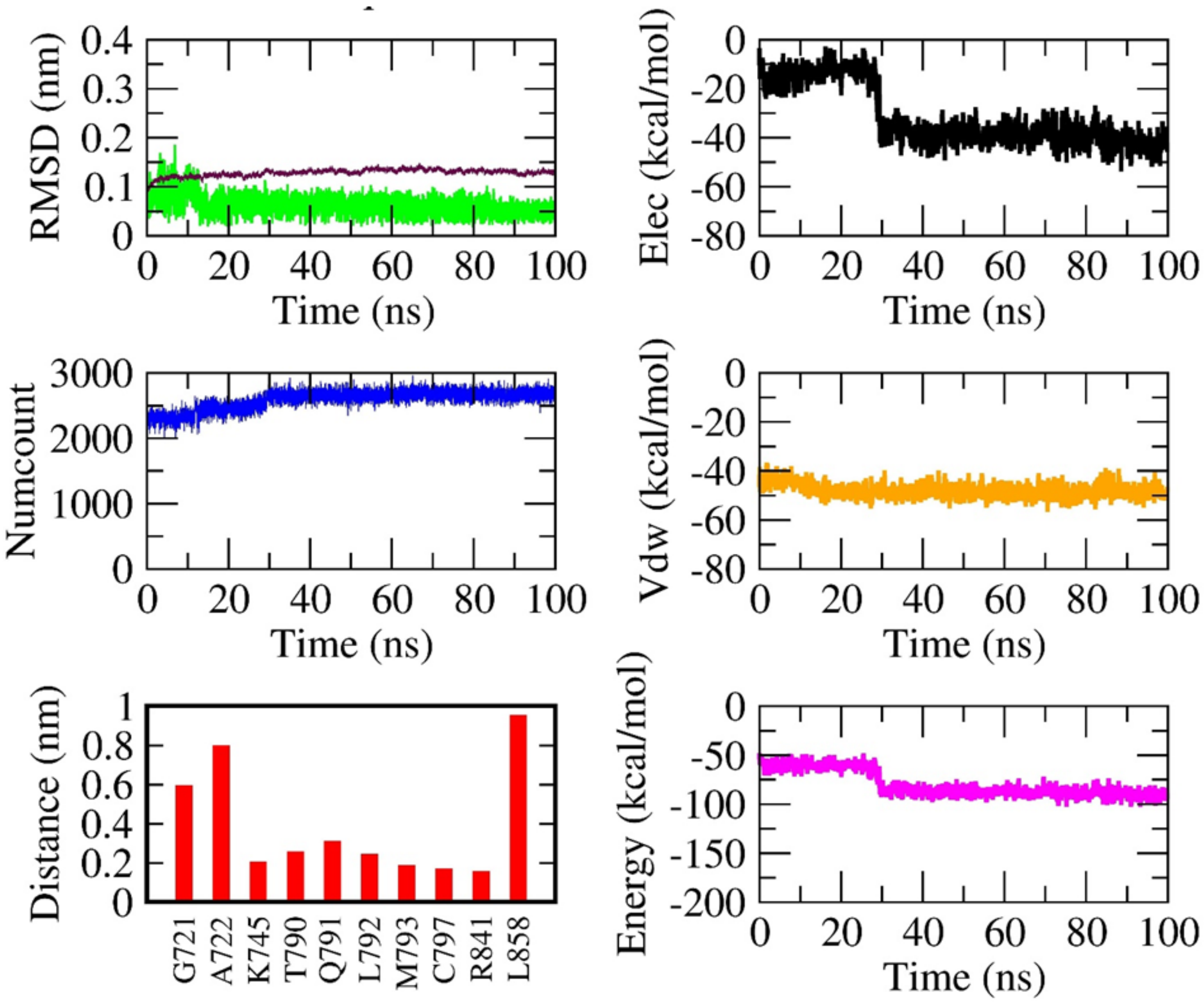

Figure S7: *Data is obtained from 100ns MD simulation in case of* ***compound – 8*** *and EGFR complex. The time – dependent root mean square deviation of protein and ligand are plotted in green and maroon, respectively. The number of contact between* ***compound – 8*** *in the dependence of time is shown in blue. Non – bonded interaction energy (in magenta) is combined from the Coulomb potential energy (in black) and the Van de Waals potential energy (in orange). The mean of min distances from* ***compound – 8*** *to 10 key residues located in the ATP region are also shown in red bar.*

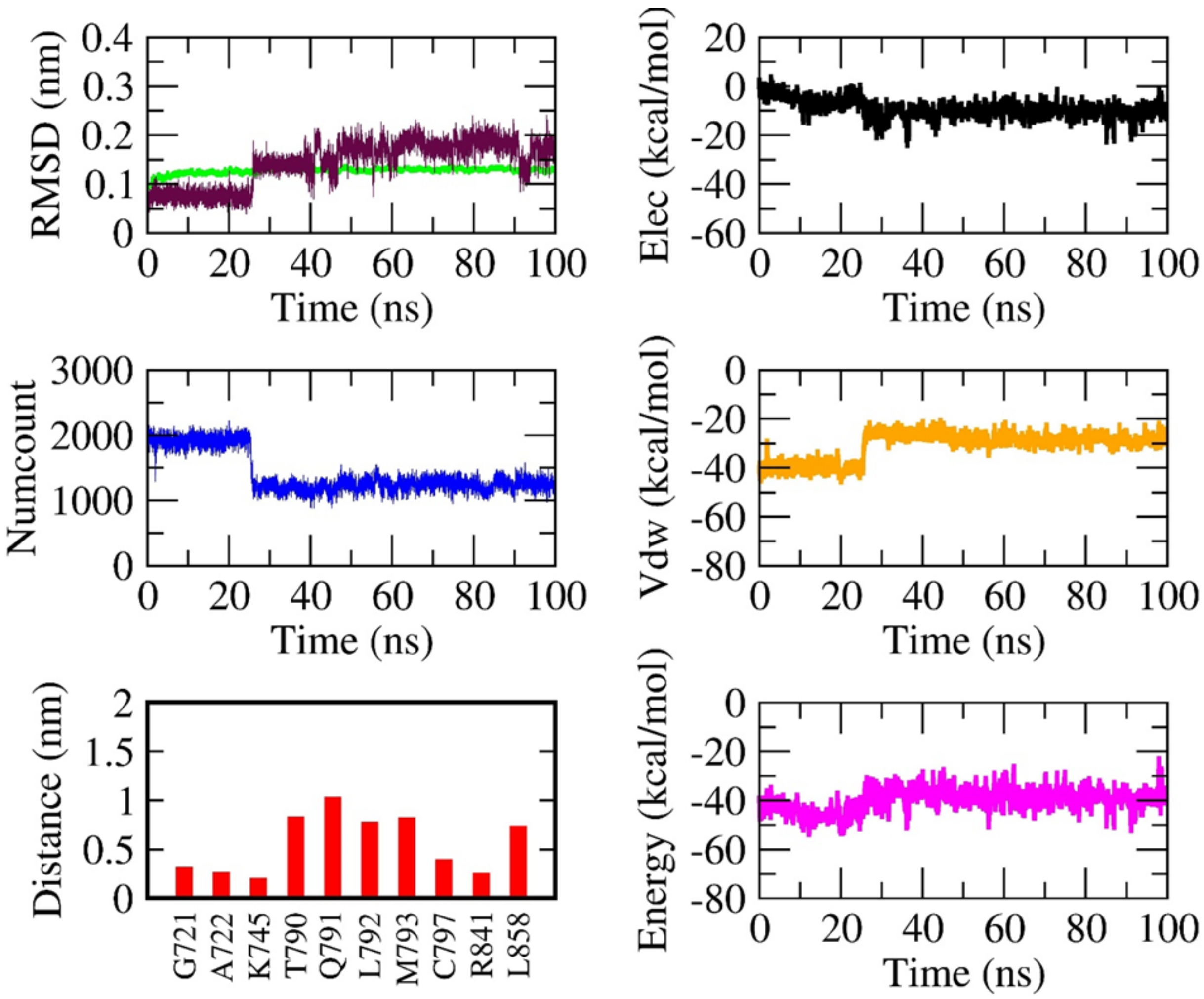

Figure S8: *Data is obtained from 100ns MD simulation in case of* ***compound – 9*** *and EGFR complex. The time – dependent root mean square deviation of protein and ligand are plotted in green and maroon, respectively. The number of contact between* ***compound – 9*** *in the dependence of time is shown in blue. Non – bonded interaction energy (in magenta) is combined from the Coulomb potential energy (in black) and the Van de Waals potential energy (in orange). The mean of min distances from* ***compound – 9*** *to 10 key residues located in the ATP region are also shown in red bar.*

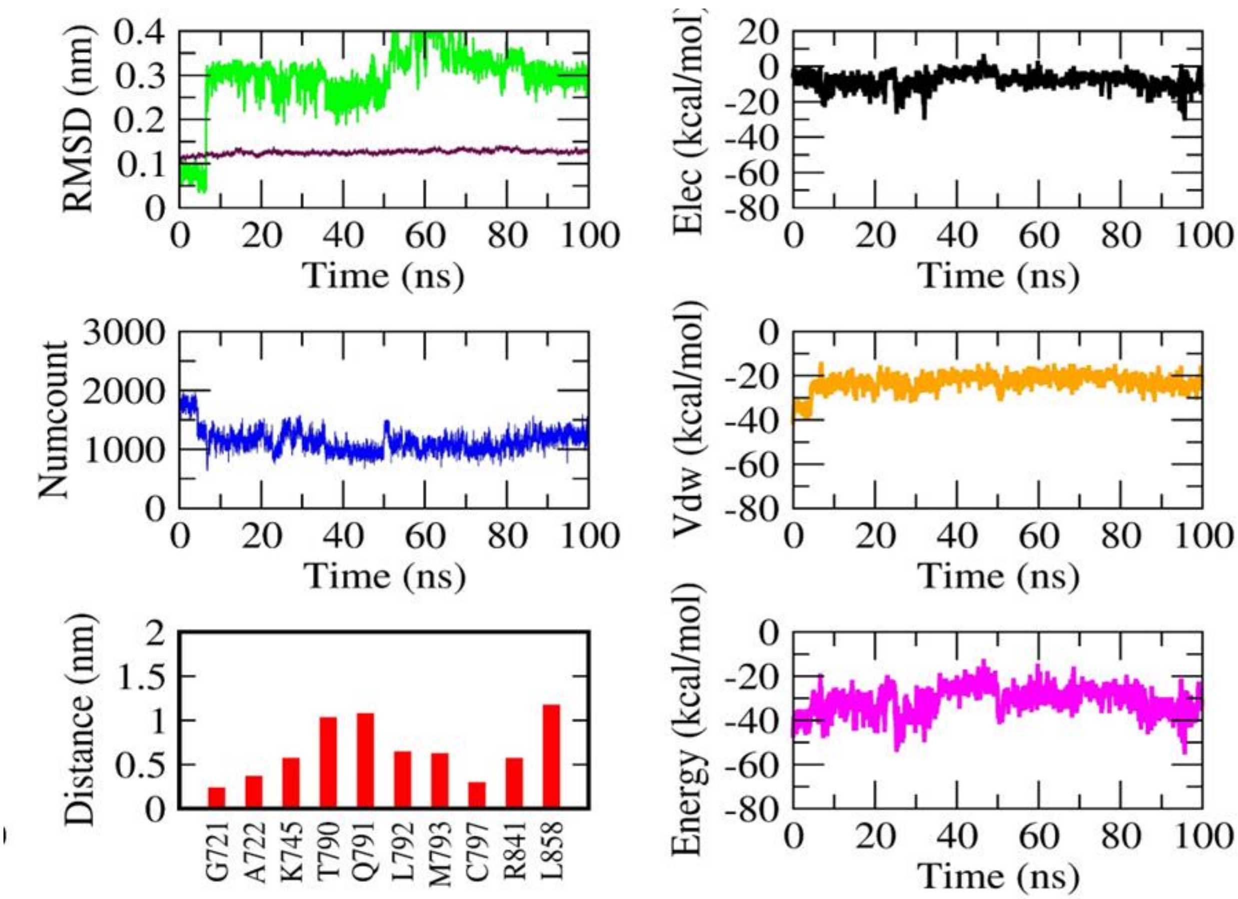

Figure S9: *Data is obtained from 100ns MD simulation in case of **compound – 10** and EGFR complex. The time – dependent root mean square deviation of protein and ligand are plotted in green and maroon, respectively. The number of contact between **compound – 10** in the dependence of time is shown in blue. Non – bonded interaction energy (in magenta) is combined from the Coulomb potential energy (in black) and the Van de Waals potential energy (in orange). The mean of min distances from **compound – 10** to 10 key residues located in the ATP region are also shown in red bar.*

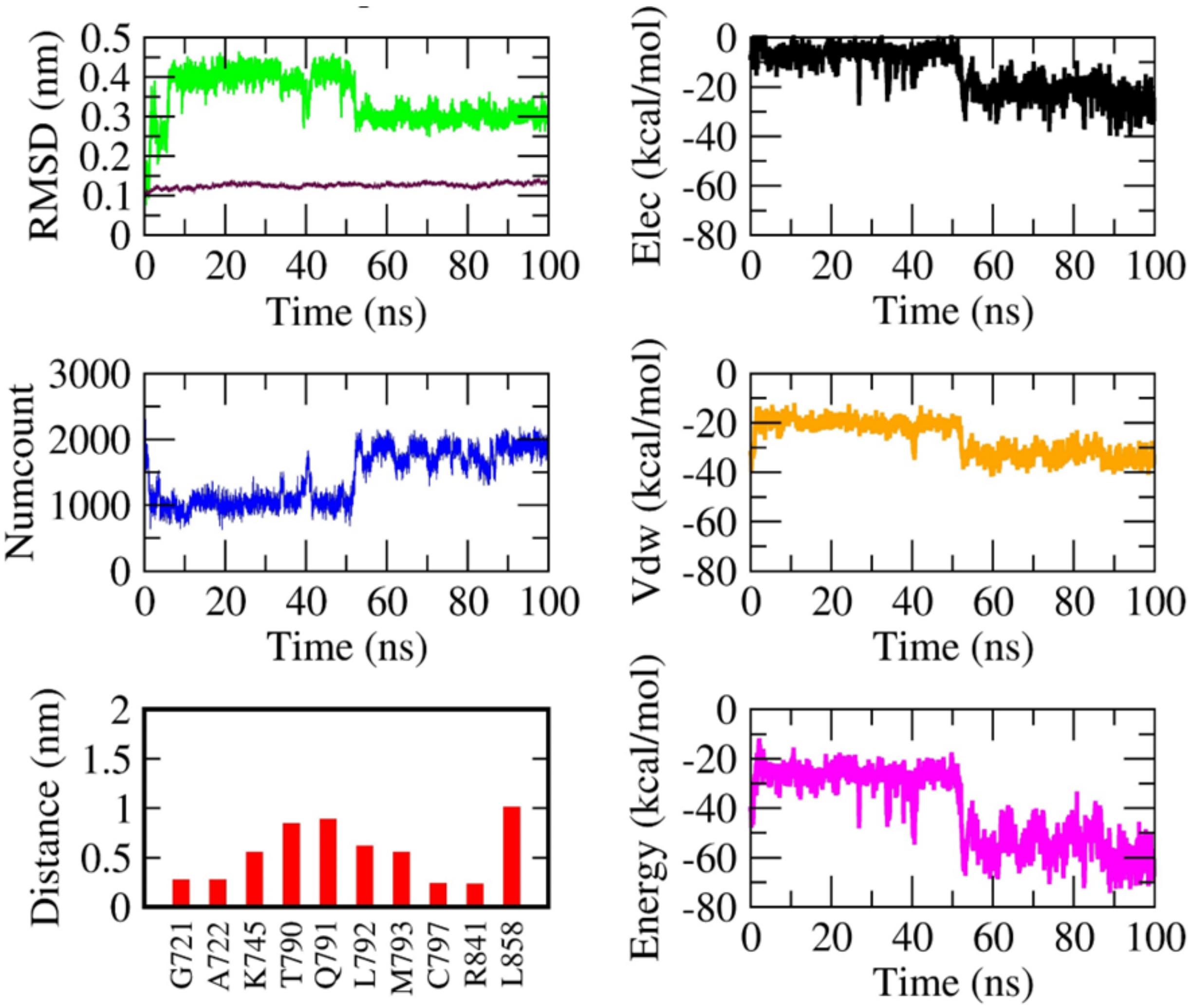

Figure S10: *Data is obtained from 100ns MD simulation in case of* ***compound – 11*** *and EGFR complex. The time – dependent root mean square deviation of protein and ligand are plotted in green and maroon, respectively. The number of contact between* ***compound – 11*** *in the dependence of time is shown in blue. Non – bonded interaction energy (in magenta) is combined from the Coulomb potential energy (in black) and the Van de Waals potential energy (in orange). The mean of min distances from* ***compound – 11*** *to 10 key residues located in the ATP region are also shown in red bar.*

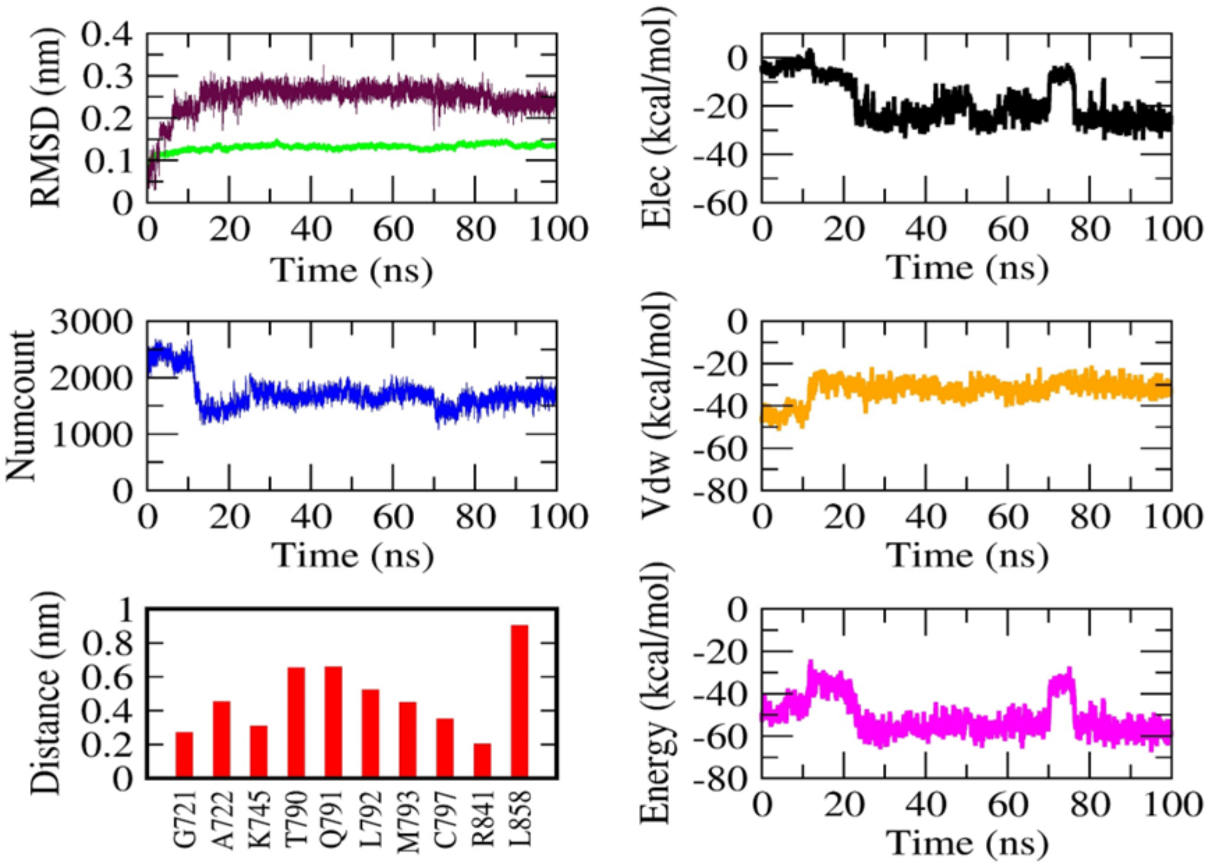

Figure S11: *Data is obtained from 100ns MD simulation in case of* ***compound – 12*** *and EGFR complex. The time – dependent root mean square deviation of protein and ligand are plotted in green and maroon, respectively. The number of contact between* ***compound – 12*** *in the dependence of time is shown in blue. Non – bonded interaction energy (in magenta) is combined from the Coulomb potential energy (in black) and the Van de Waals potential energy (in orange). The mean of min distances from* ***compound – 12*** *to 10 key residues located in the ATP region are also shown in red bar.*

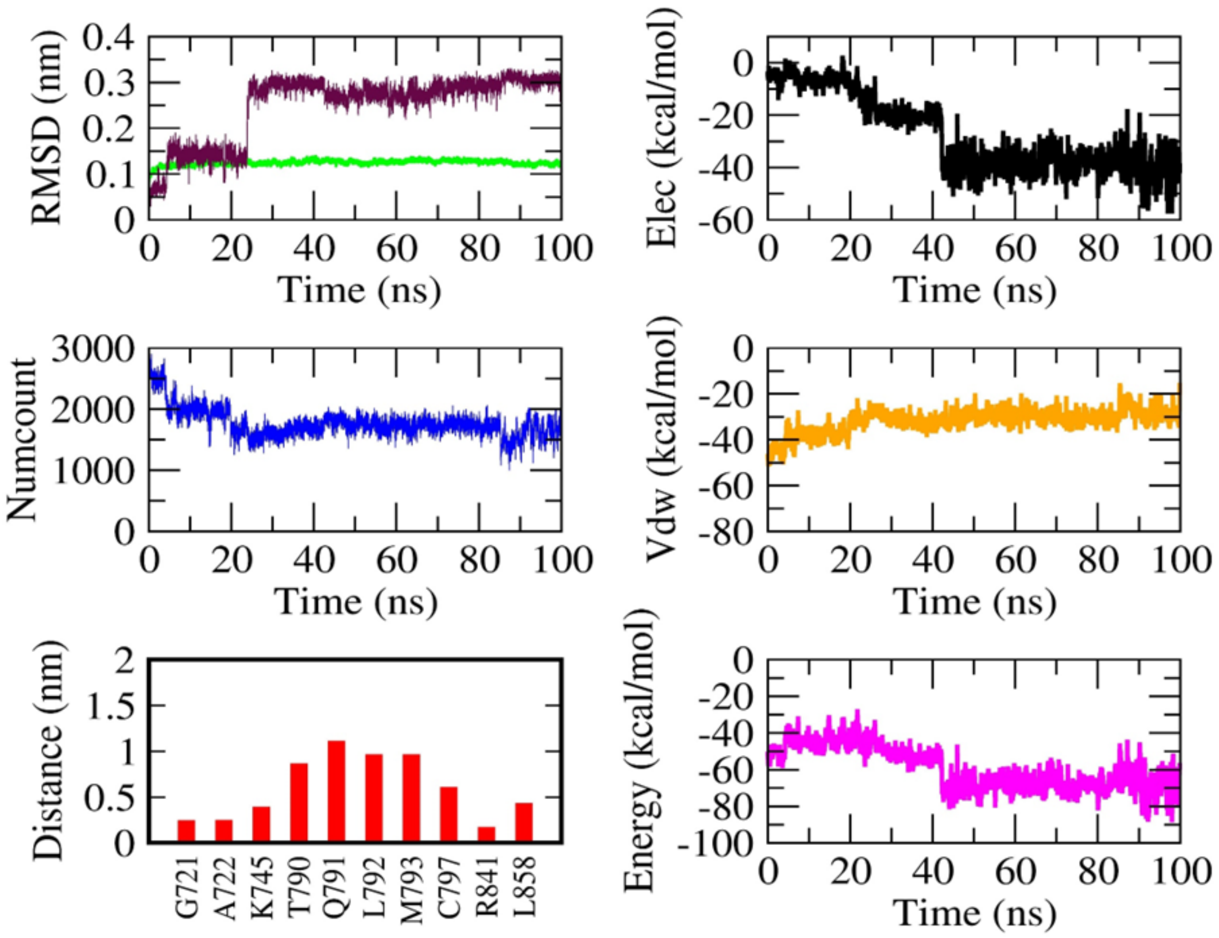

Figure S12: *Data is obtained from 100ns MD simulation in case of* ***compound – 13*** *and EGFR complex. The time – dependent root mean square deviation of protein and ligand are plotted in green and maroon, respectively. The number of contact between* ***compound – 13*** *in the dependence of time is shown in blue. Non – bonded interaction energy (in magenta) is combined from the Coulomb potential energy (in black) and the Van de Waals potential energy (in orange). The mean of min distances from* ***compound – 13*** *to 10 key residues located in the ATP region are also shown in red bar.*

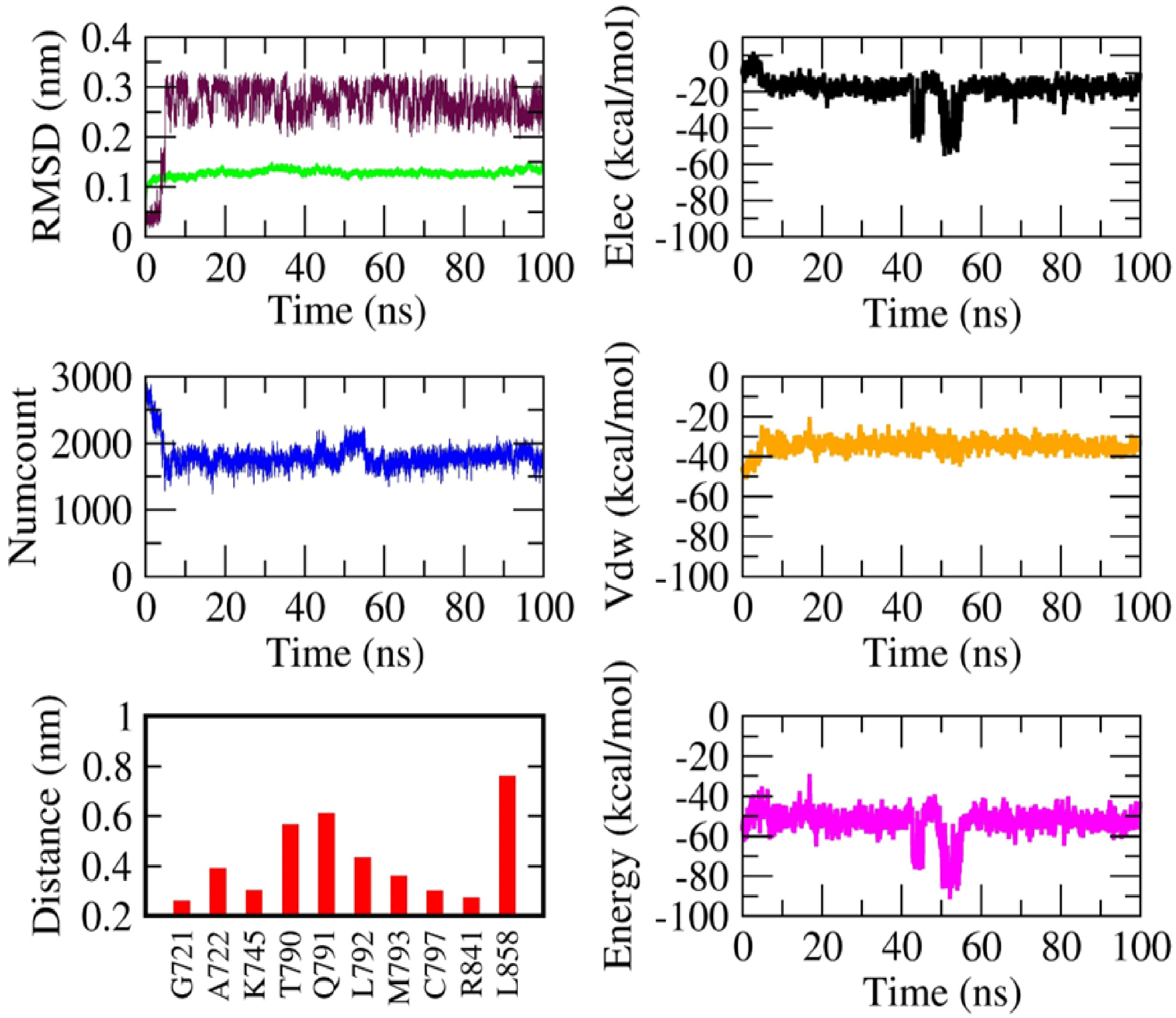

Figure S13: *Data is obtained from 100ns MD simulation in case of* ***compound – 14*** *and EGFR complex. The time – dependent root mean square deviation of protein and ligand are plotted in green and maroon, respectively. The number of contact between* ***compound – 14*** *in the dependence of time is shown in blue. Non – bonded interaction energy (in magenta) is combined from the Coulomb potential energy (in black) and the Van de Waals potential energy (in orange). The mean of min distances from* ***compound – 14*** *to 10 key residues located in the ATP region are also shown in red bar.*

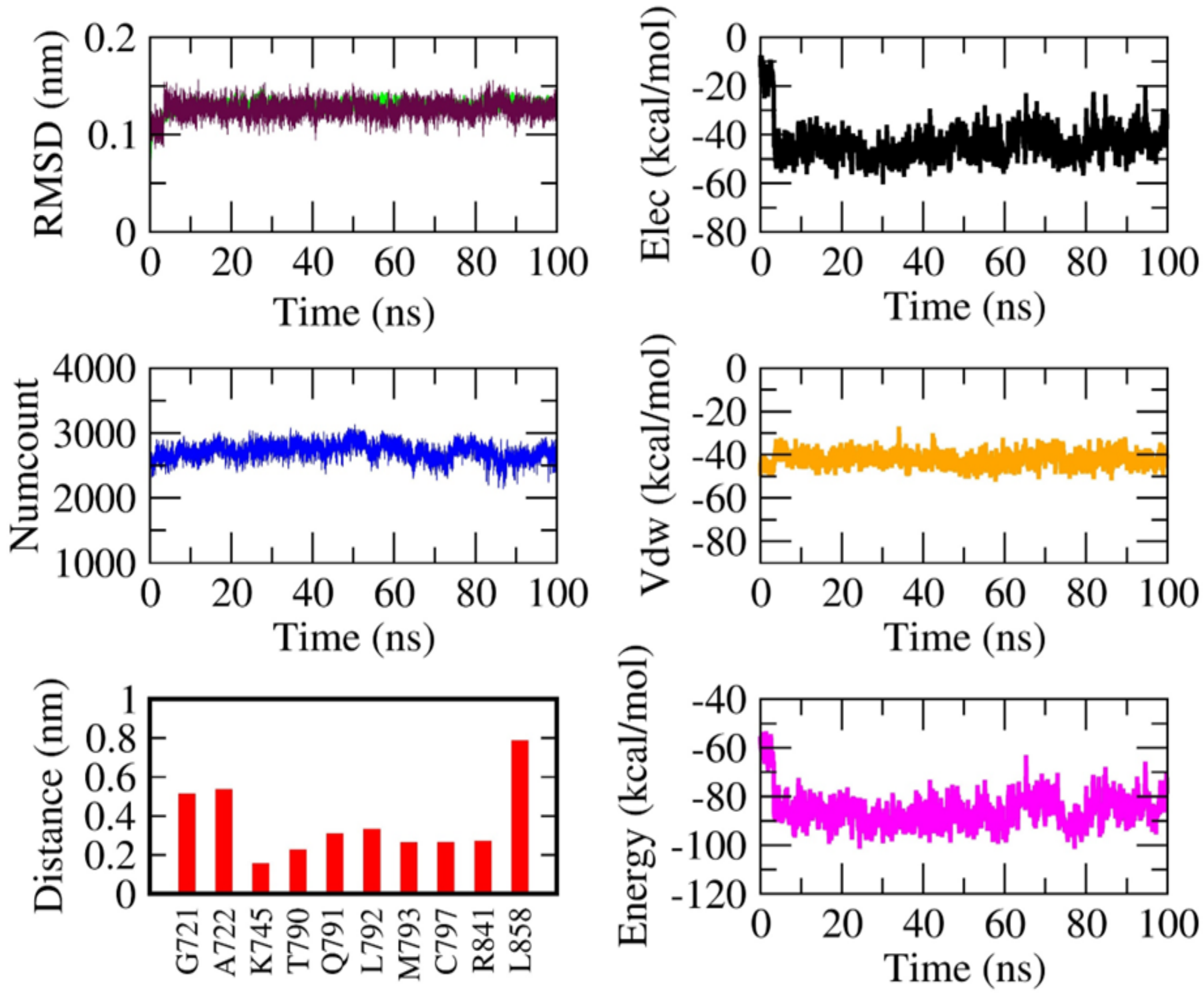

Figure S14: *Data is obtained from 100ns MD simulation in case of* ***compound – 15*** *and EGFR complex. The time – dependent root mean square deviation of protein and ligand are plotted in green and maroon, respectively. The number of contact between* ***compound – 15*** *in the dependence of time is shown in blue. Non – bonded interaction energy (in magenta) is combined from the Coulomb potential energy (in black) and the Van de Waals potential energy (in orange). The mean of min distances from* ***compound – 15*** *to 10 key residues located in the ATP region are also shown in red bar.*

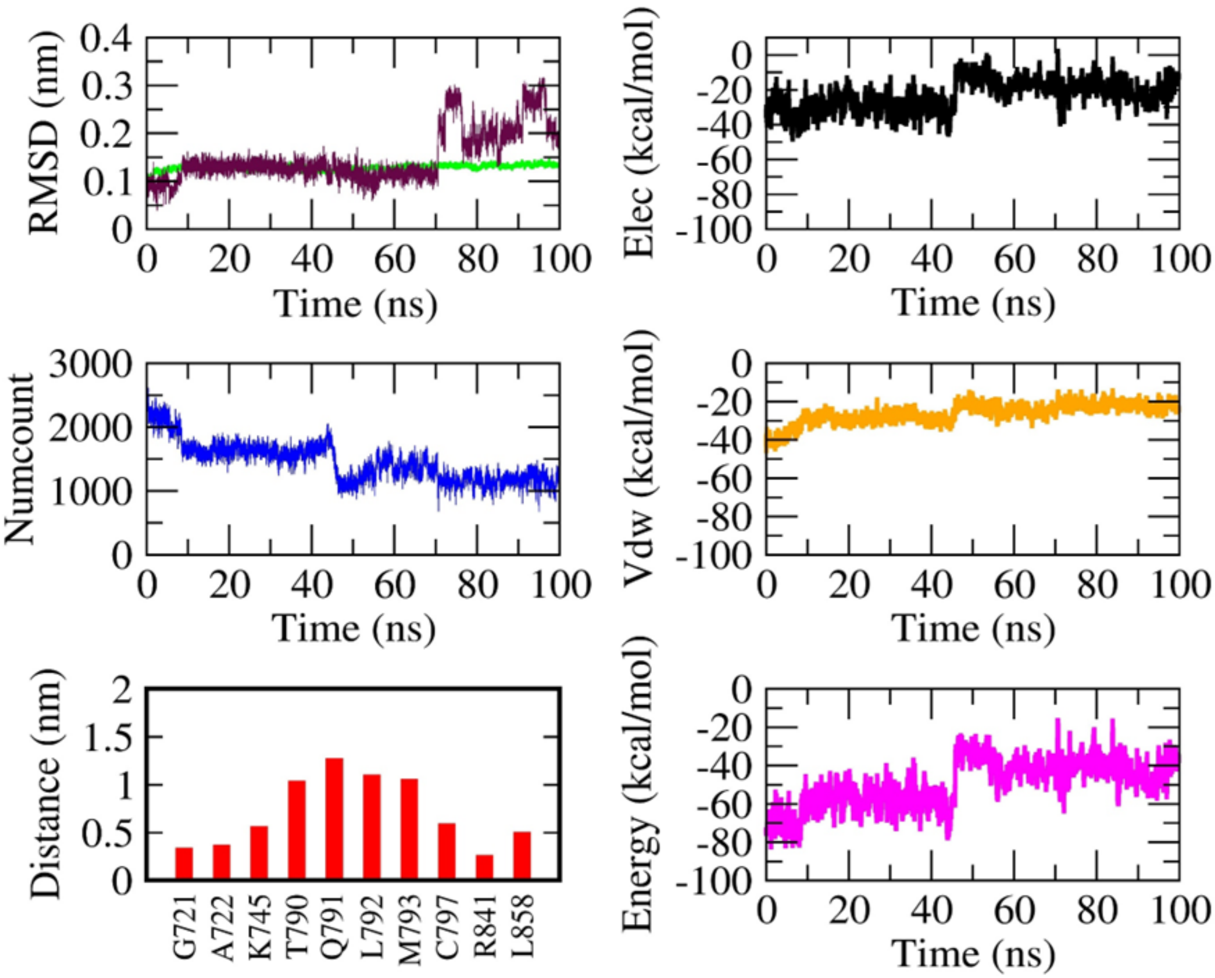

Figure S15: *Data is obtained from 100ns MD simulation in case of* ***compound – 16*** *and EGFR complex. The time – dependent root mean square deviation of protein and ligand are plotted in green and maroon, respectively. The number of contact between* ***compound – 16*** *in the dependence of time is shown in blue. Non – bonded interaction energy (in magenta) is combined from the Coulomb potential energy (in black) and the Van de Waals potential energy (in orange). The mean of min distances from* ***compound – 16*** *to 10 key residues located in the ATP region are also shown in red bar.*

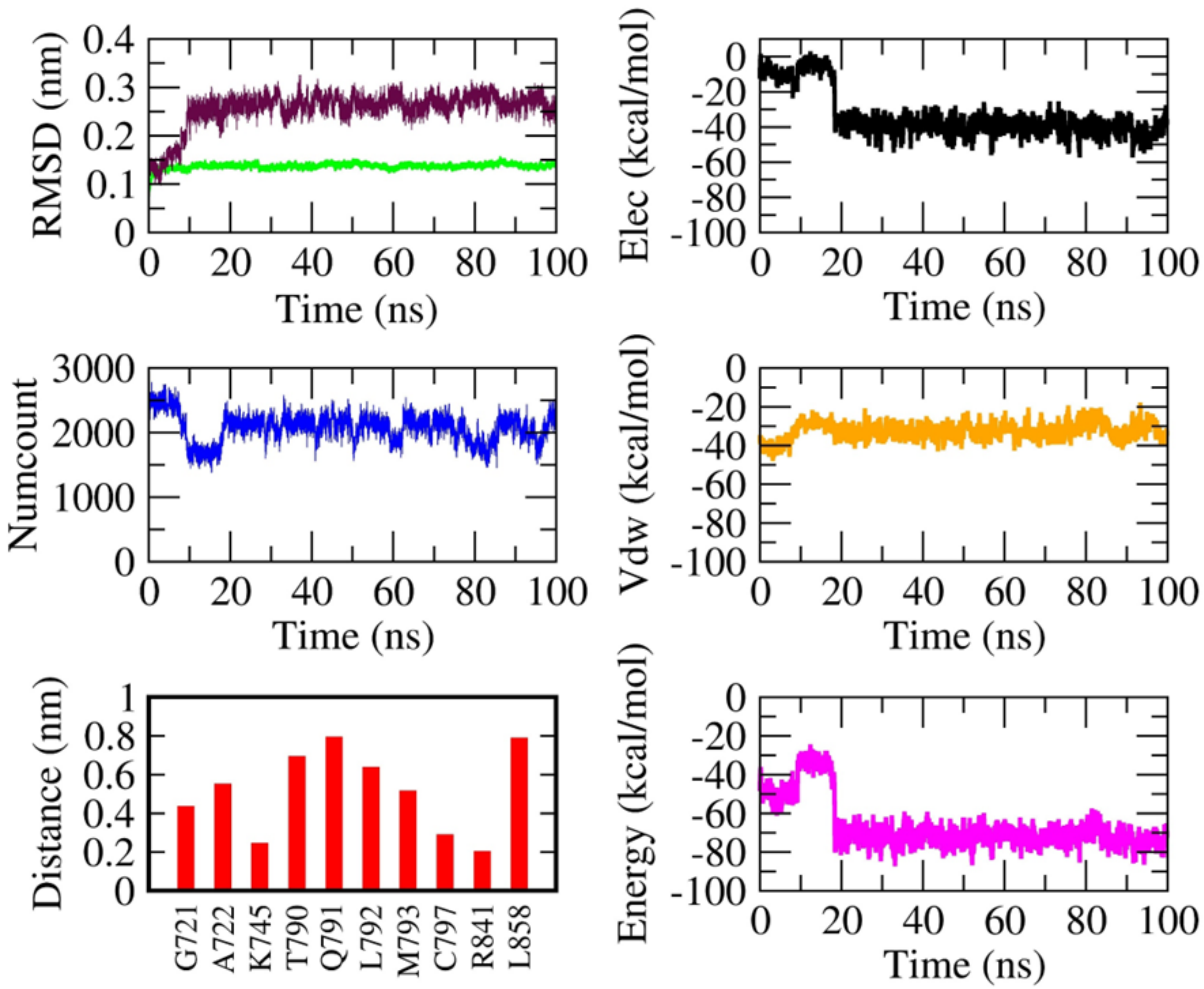

Figure S16: *Data is obtained from 100ns MD simulation in case of* ***compound – 17*** *and EGFR complex. The time – dependent root mean square deviation of protein and ligand are plotted in green and maroon, respectively. The number of contact between* ***compound – 17*** *in the dependence of time is shown in blue. Non – bonded interaction energy (in magenta) is combined from the Coulomb potential energy (in black) and the Van de Waals potential energy (in orange). The mean of min distances from* ***compound – 17*** *to 10 key residues located in the ATP region are also shown in red bar.*

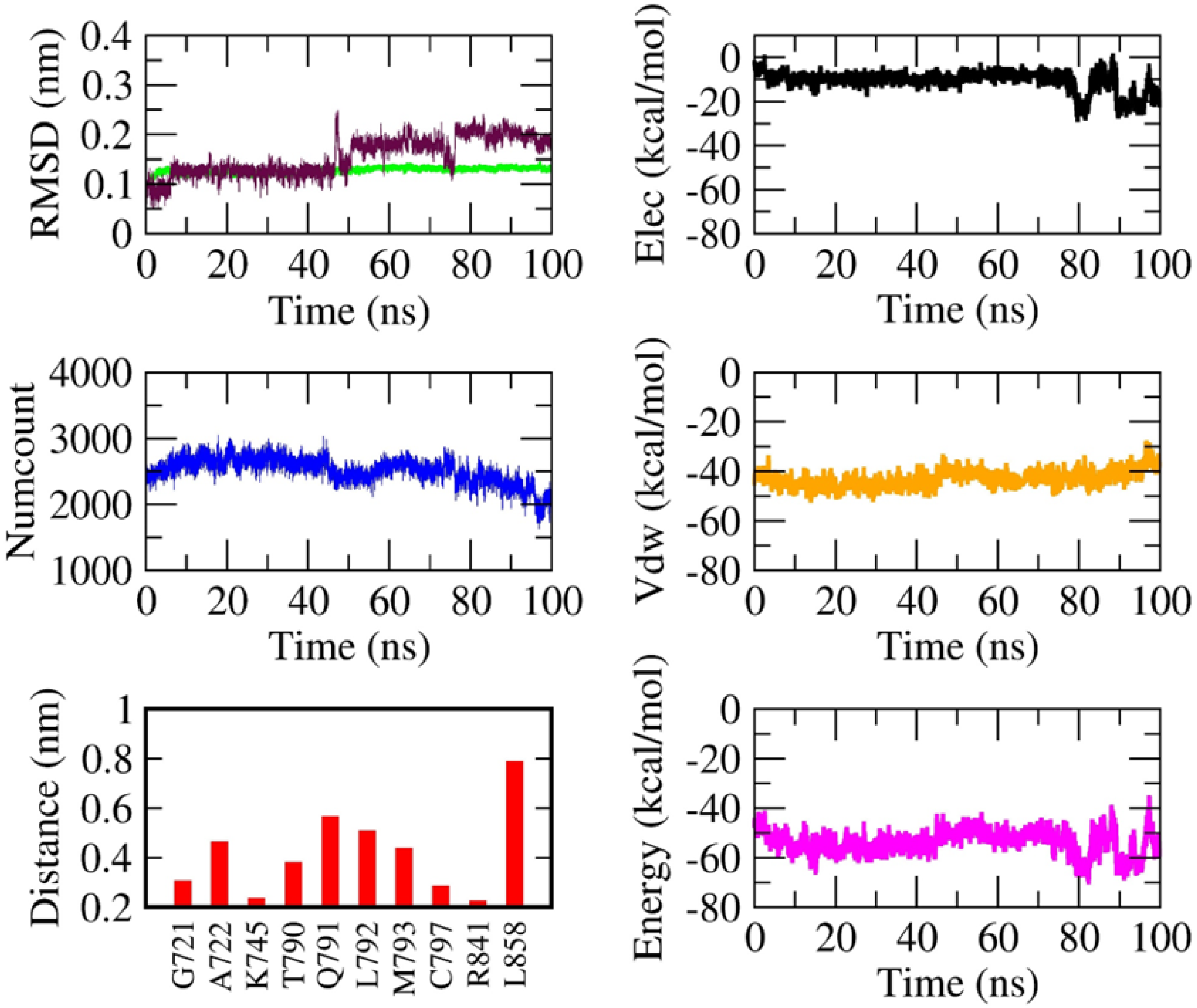

Figure S17: *Data is obtained from 100ns MD simulation in case of* ***compound – 18*** *and EGFR complex. The time – dependent root mean square deviation of protein and ligand are plotted in green and maroon, respectively. The number of contact between* ***compound – 18*** *in the dependence of time is shown in blue. Non – bonded interaction energy (in magenta) is combined from the Coulomb potential energy (in black) and the Van de Waals potential energy (in orange). The mean of min distances from* ***compound – 18*** *to 10 key residues located in the ATP region are also shown in red bar.*

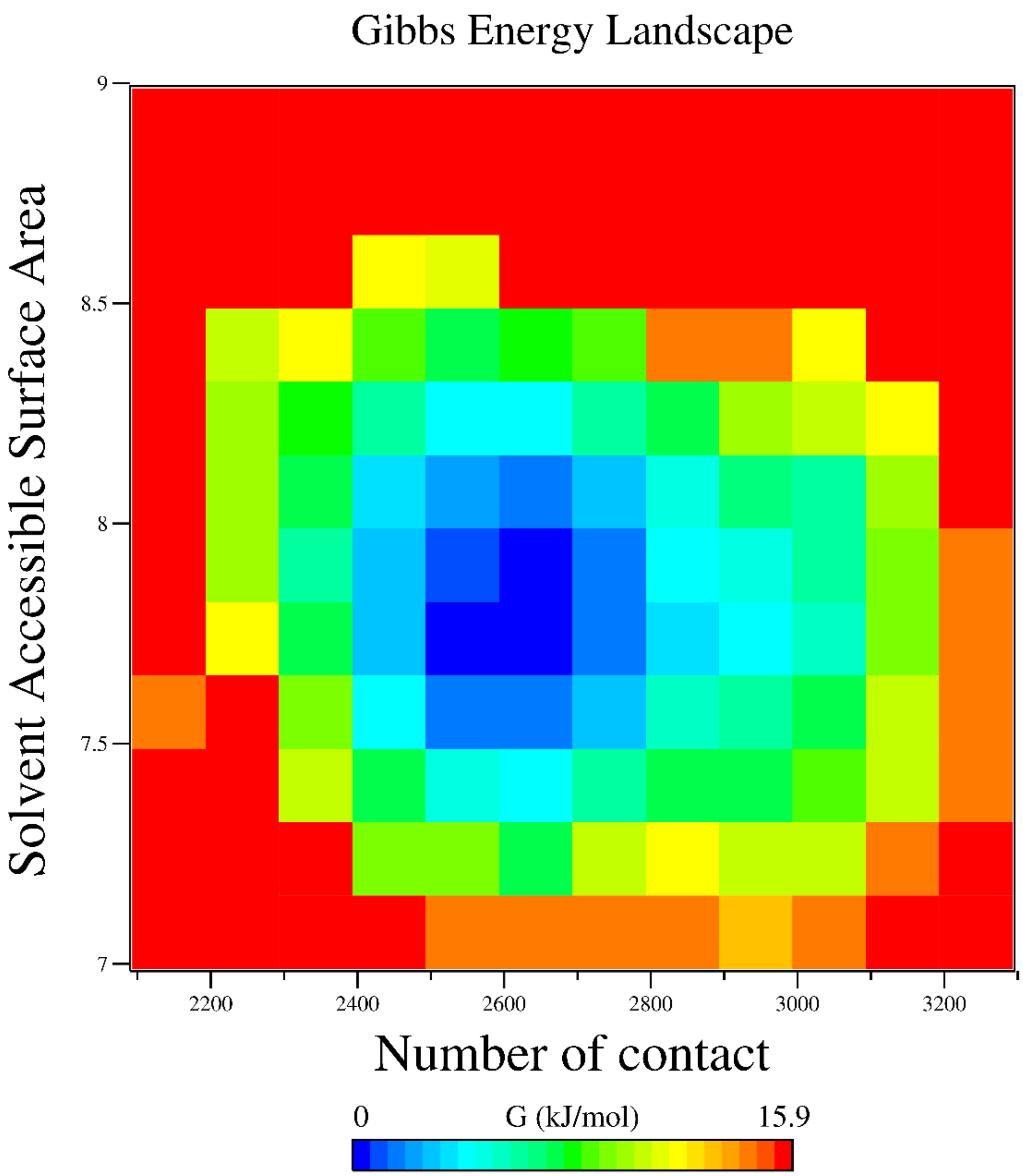

Figure S18: *The free energy landscape (FES) obtained from the last 50 ns of 100 ns MD simulation of compound* ***3a****-EGFR complex*

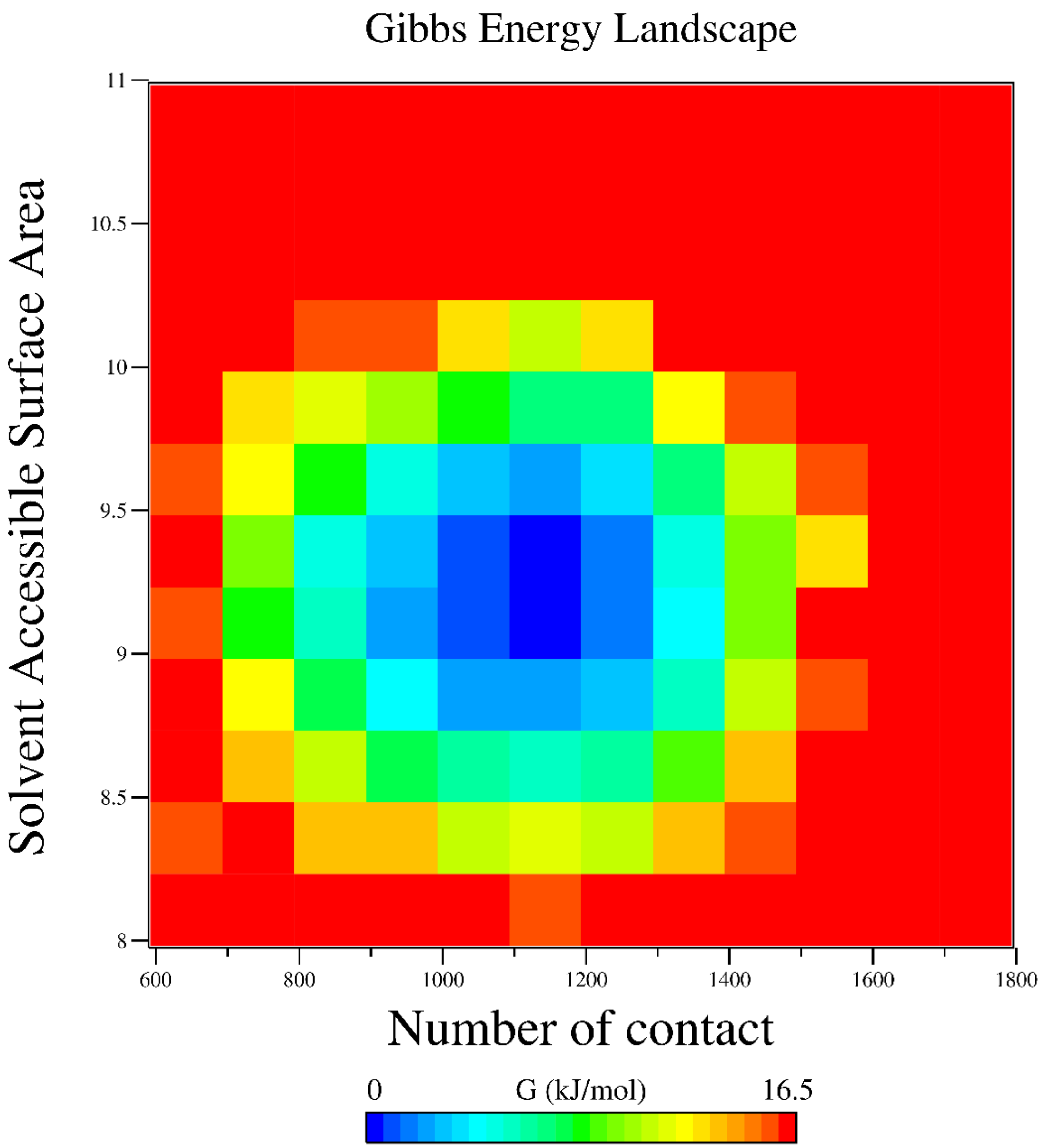

Figure S19: *The free energy landscape (FES) obtained from the last 50 ns of 100 ns MD simulation of compound* ***3b****-EGFR complex*

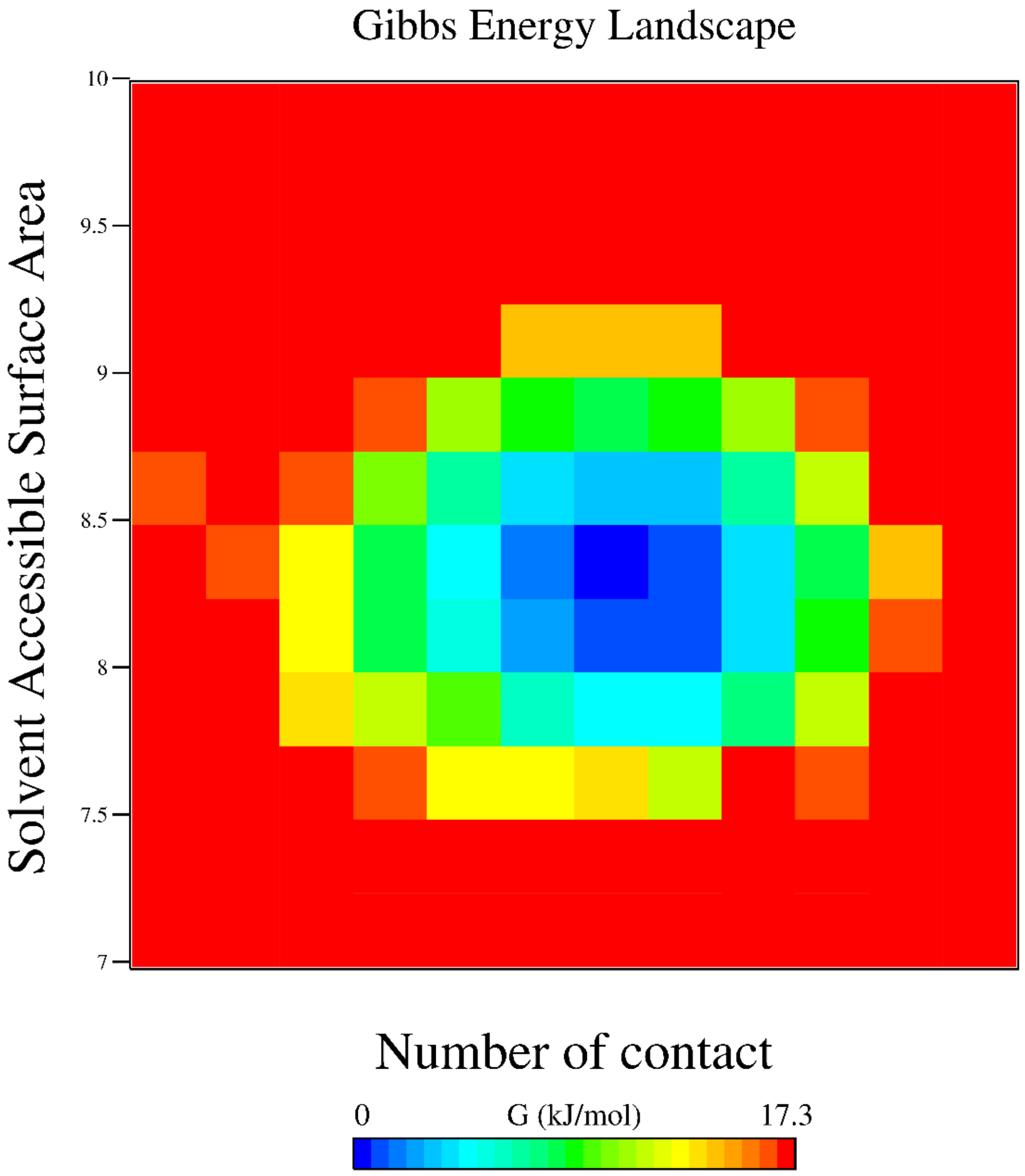

Figure S20: *The free energy landscape (FES) obtained from the last 50 ns of 100 ns MD simulation of compound* ***3d****-EGFR complex*

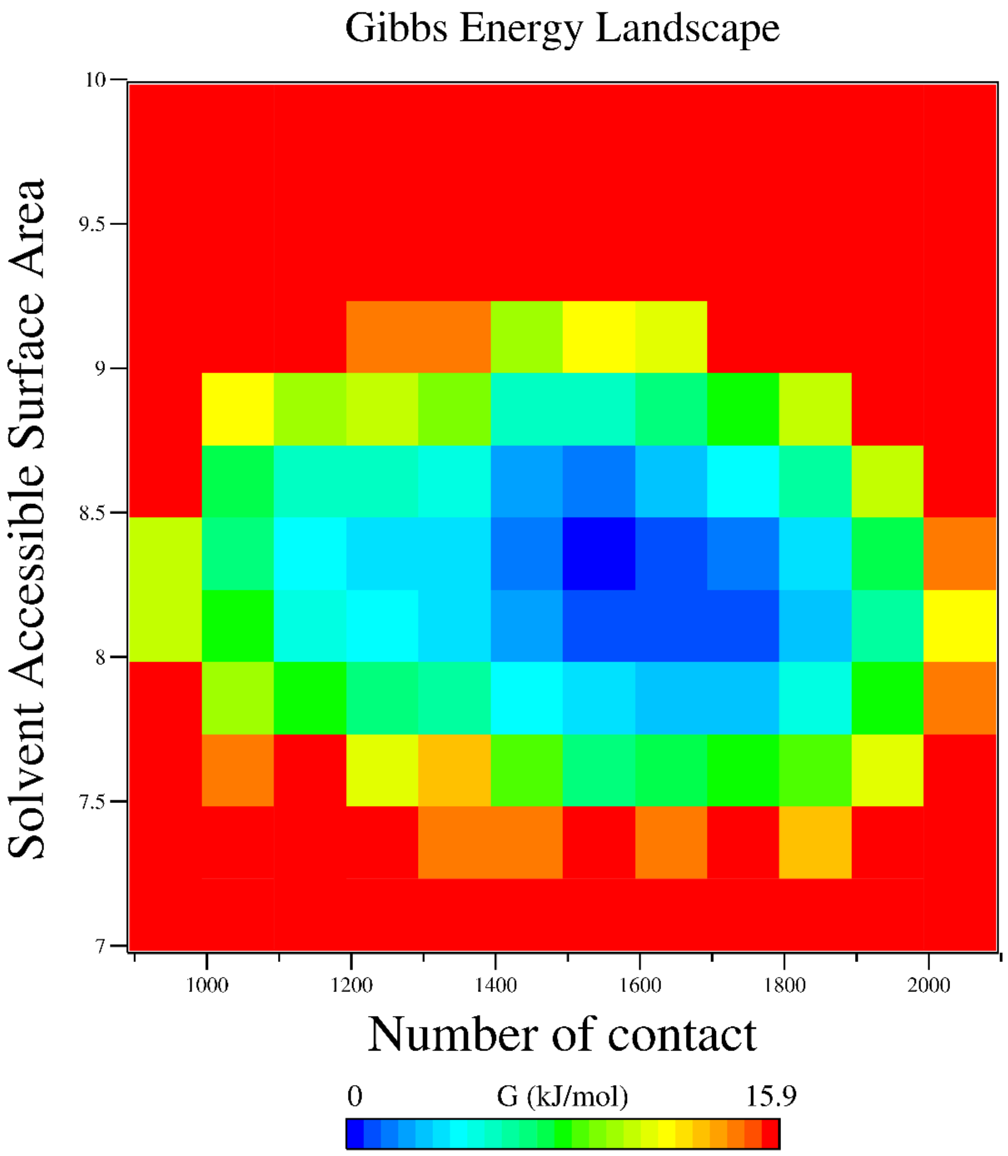

Figure S21: *The free energy landscape (FES) obtained from the last 50 ns of 100 ns MD simulation of compound **3e**-EGFR complex*

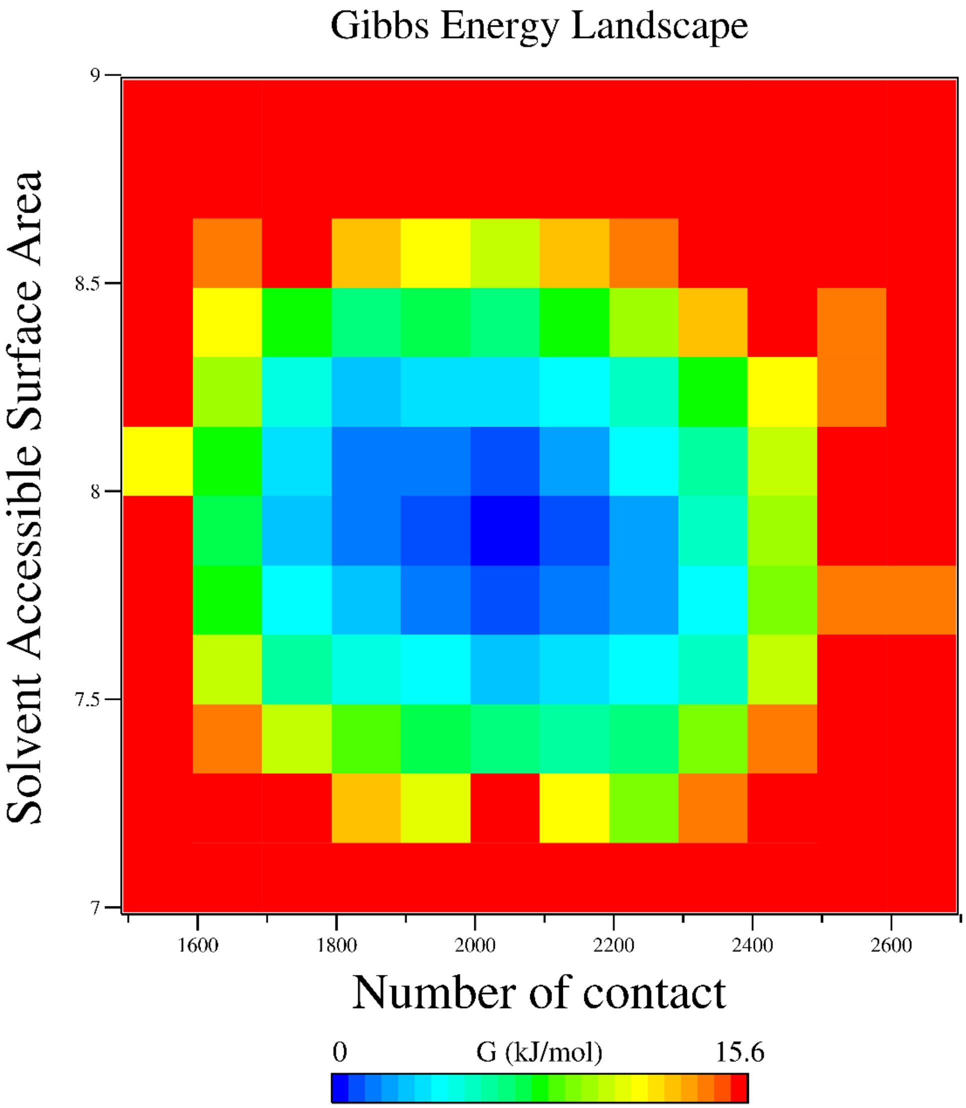

Figure S22: *The free energy landscape (FES) obtained from the last 50 ns of 100 ns MD simulation of compound **3f**-EGFR complex*

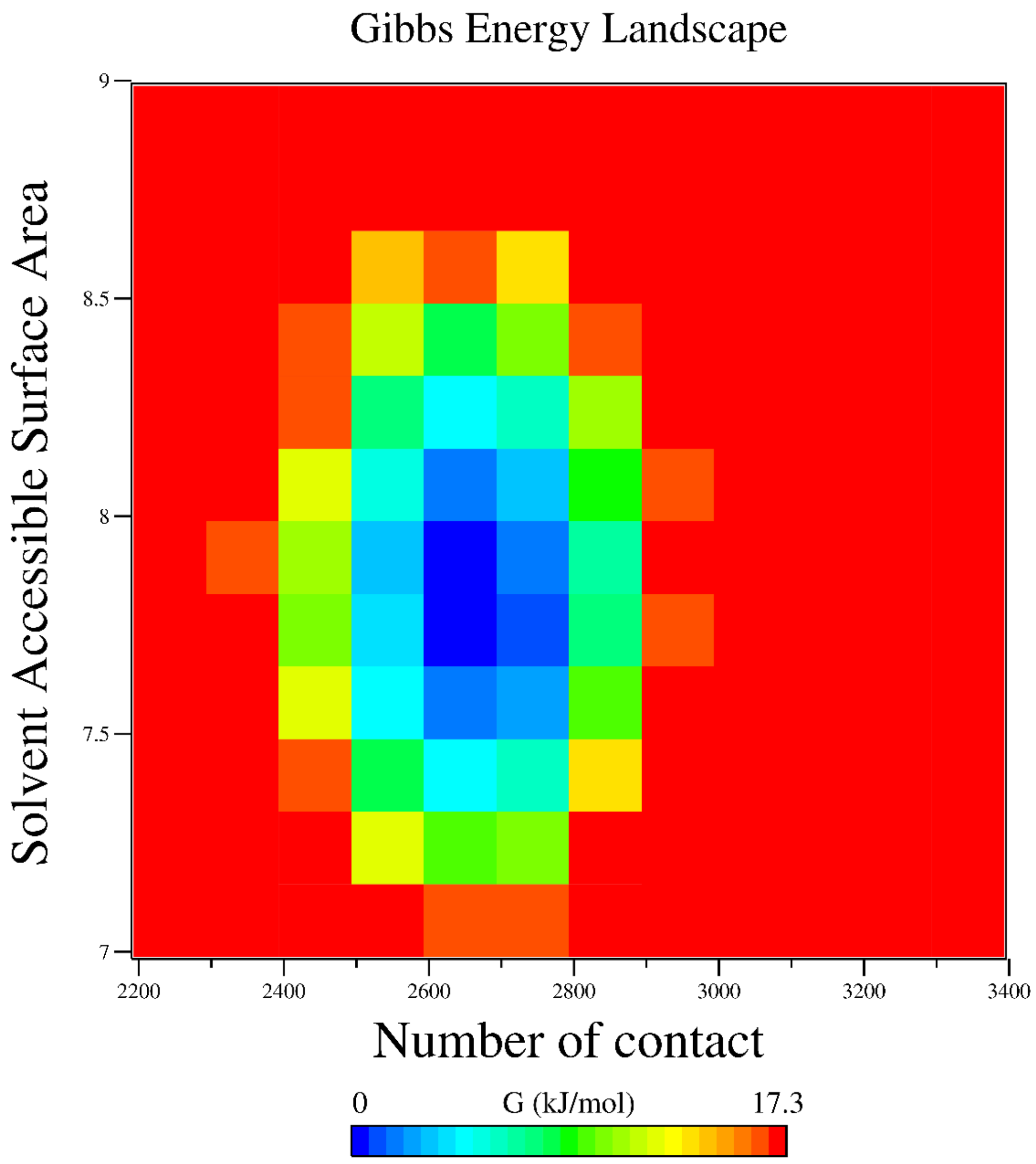

Figure S23: *The free energy landscape (FES) obtained from the last 50 ns of 100 ns MD simulation of compound* ***3g****-EGFR complex*

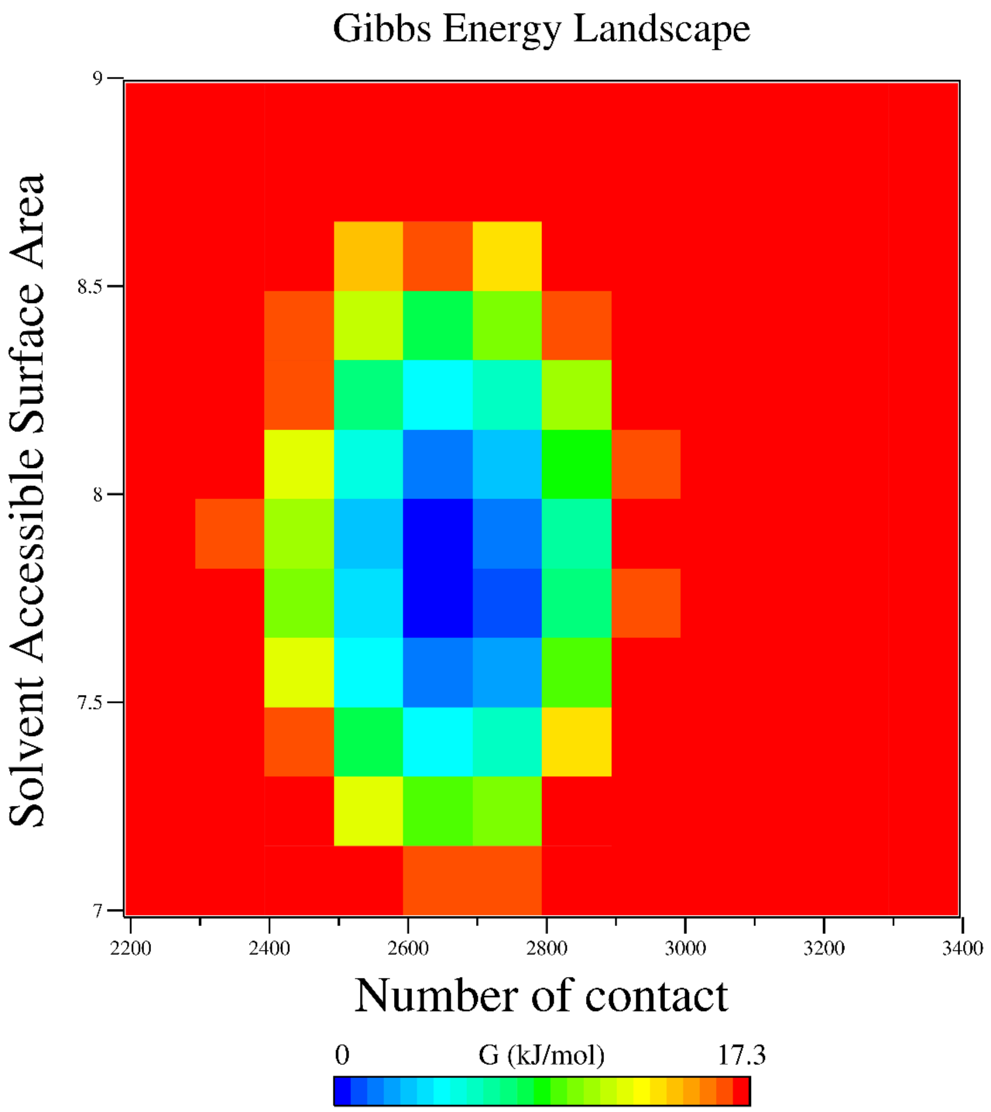

Figure S24: *The free energy landscape (FES) obtained from the last 50 ns of 100 ns MD simulation of compound **3h**-EGFR complex*

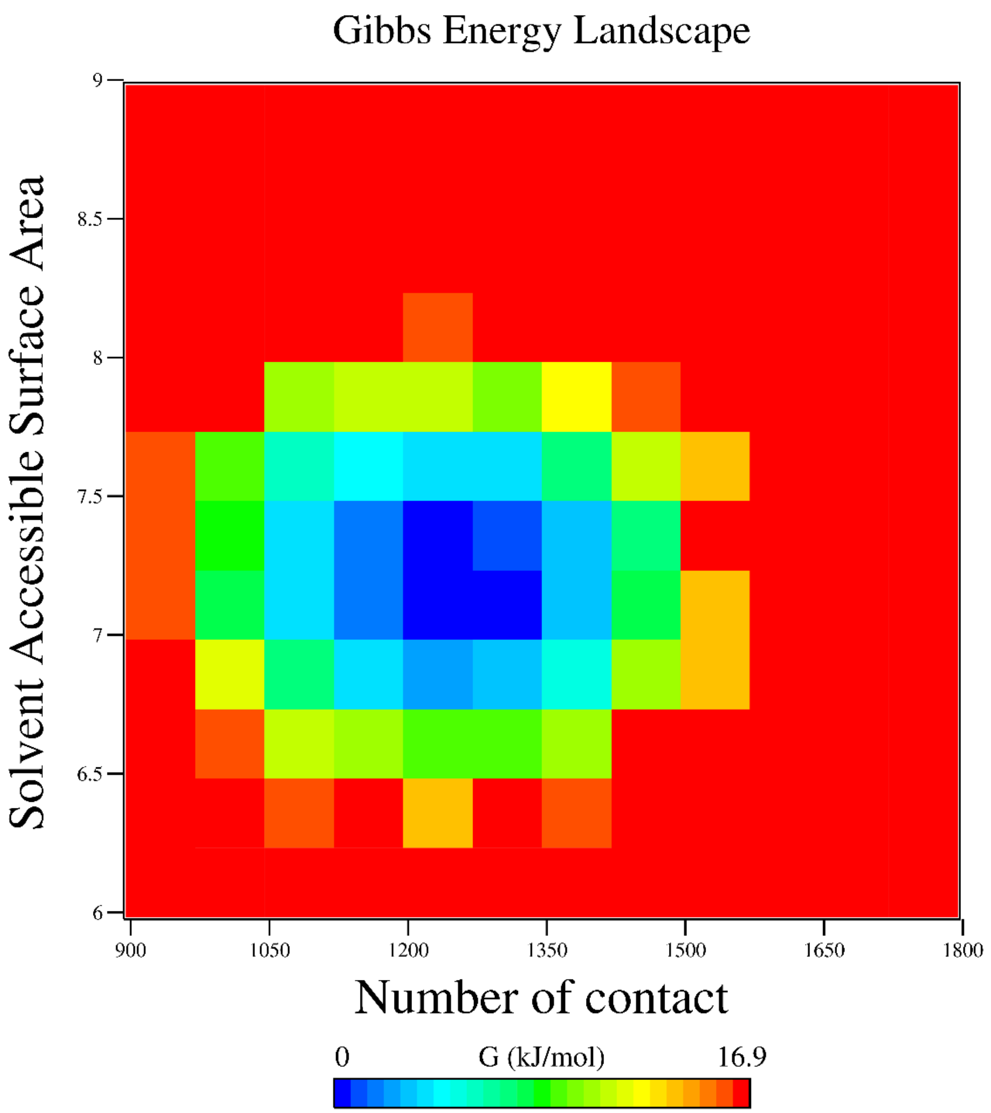

Figure S25: *The free energy landscape (FES) obtained from the last 50 ns of 100 ns MD simulation of compound* ***3i****-EGFR complex*

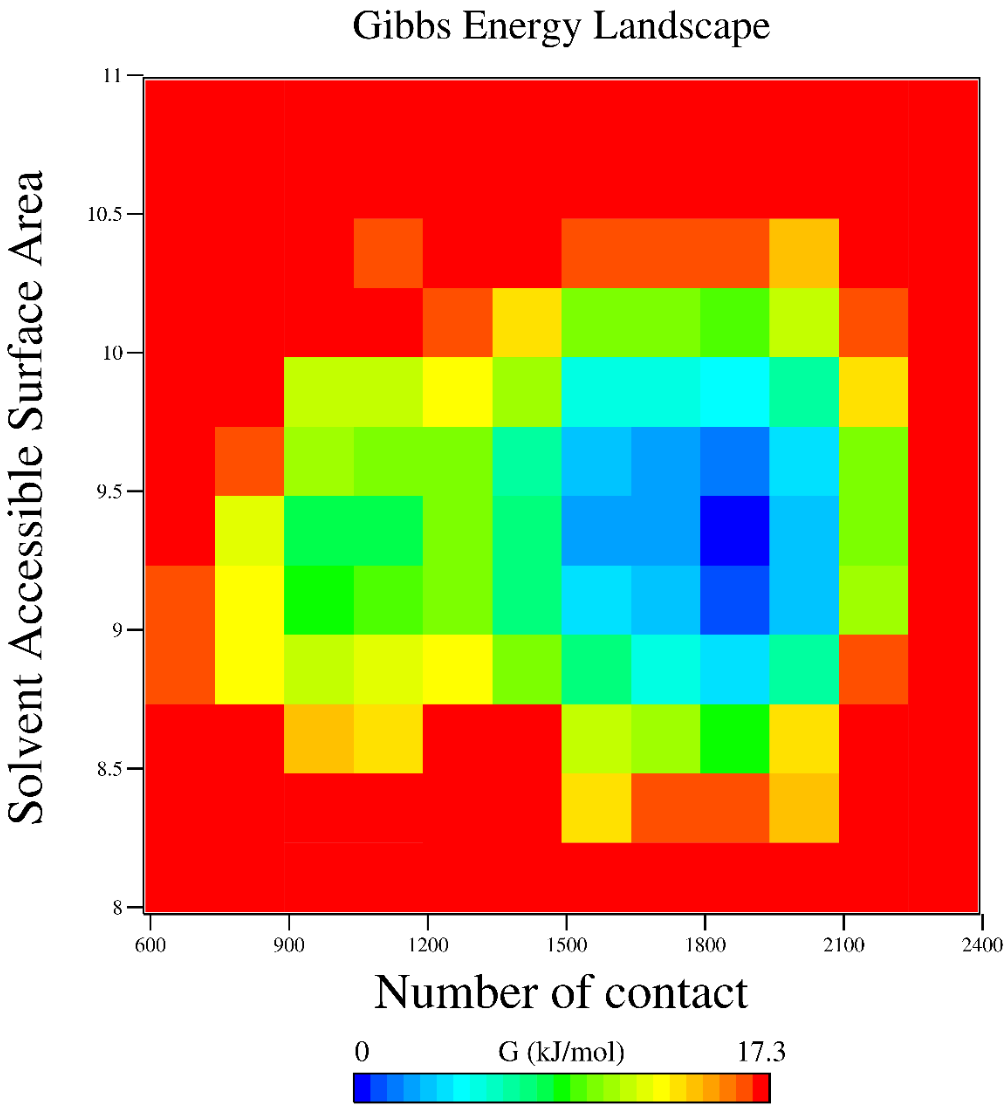

Figure S26: *The free energy landscape (FES) obtained from the last 50 ns of 100 ns MD simulation of compound* ***3k****-EGFR complex*

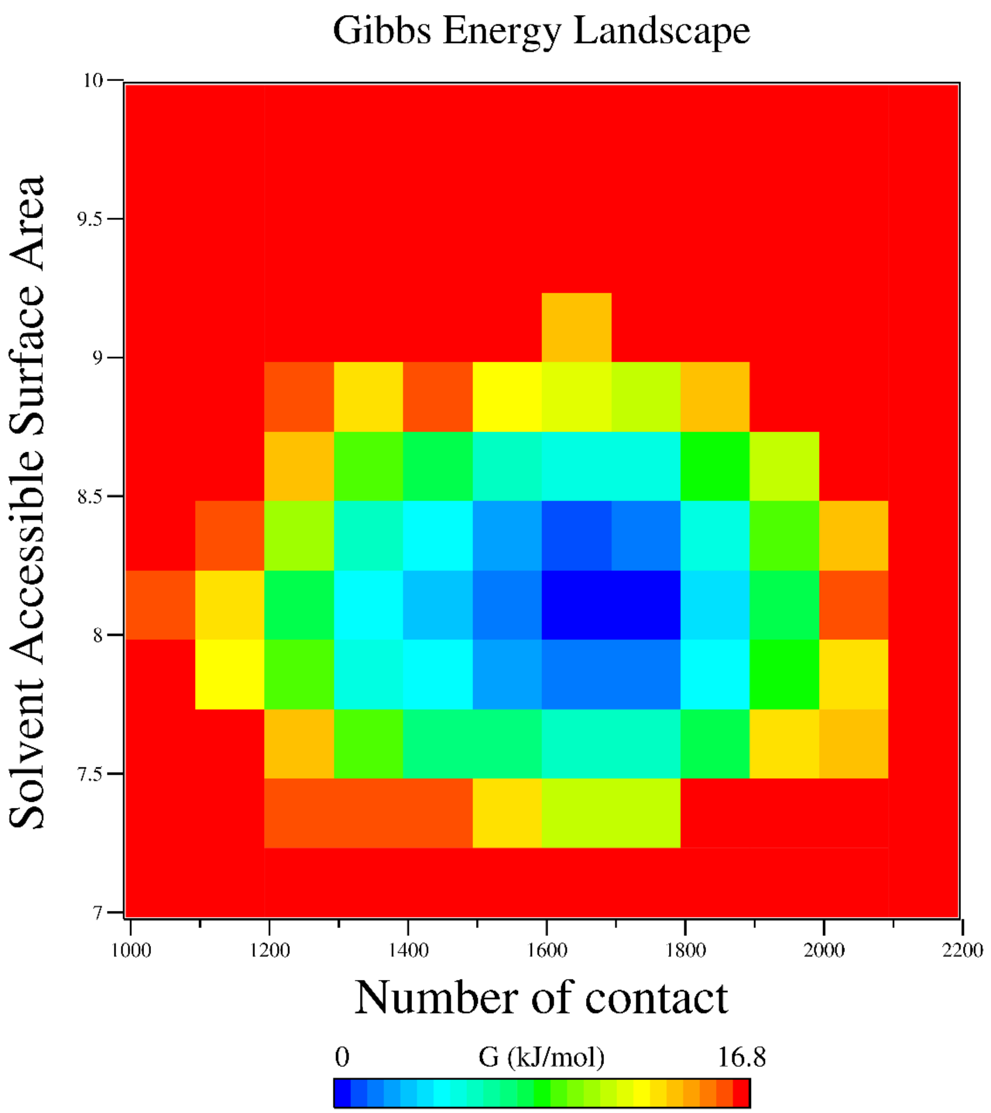

Figure S27: *The free energy landscape (FES) obtained from the last 50 ns of 100 ns MD simulation of compound **4a**-EGFR complex*

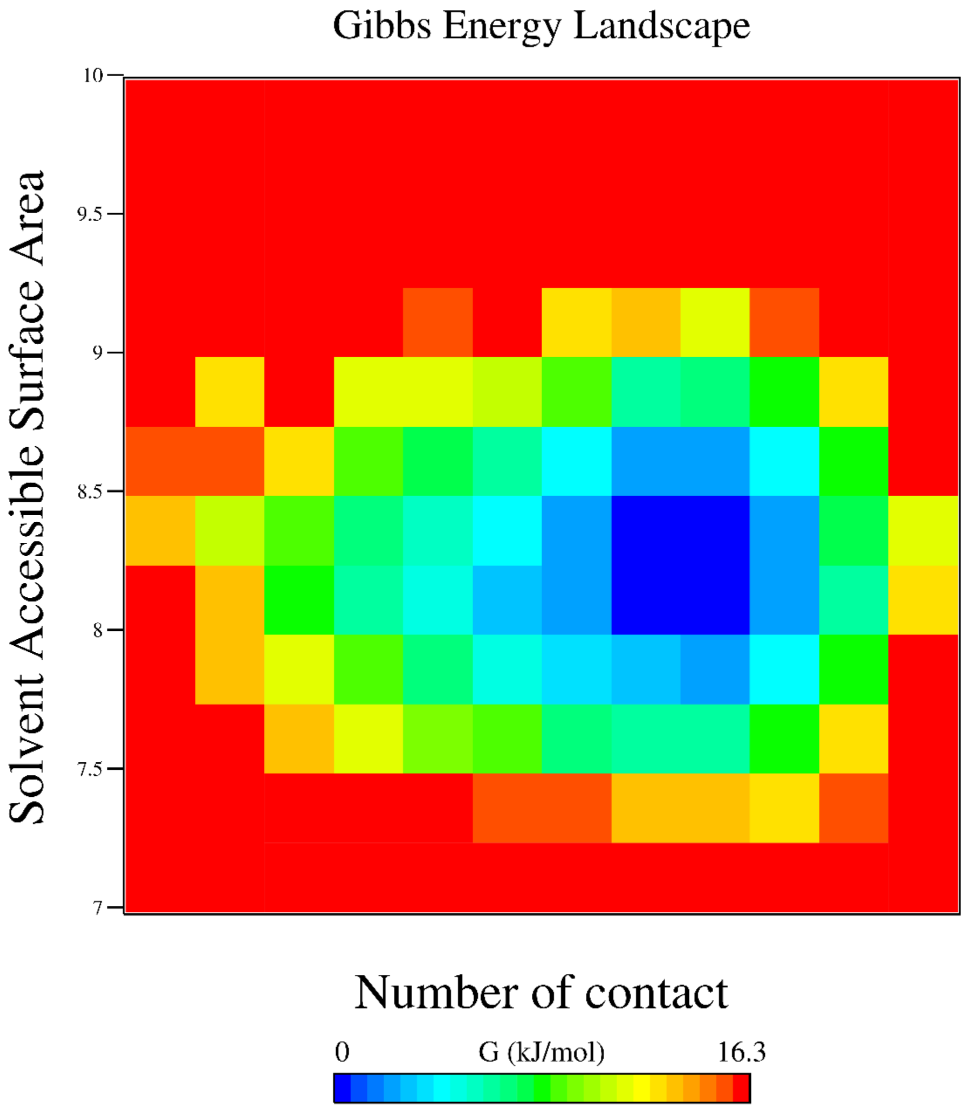

Figure S28: *The free energy landscape (FES) obtained from the last 50 ns of 100 ns MD simulation of compound **4b**-EGFR complex*

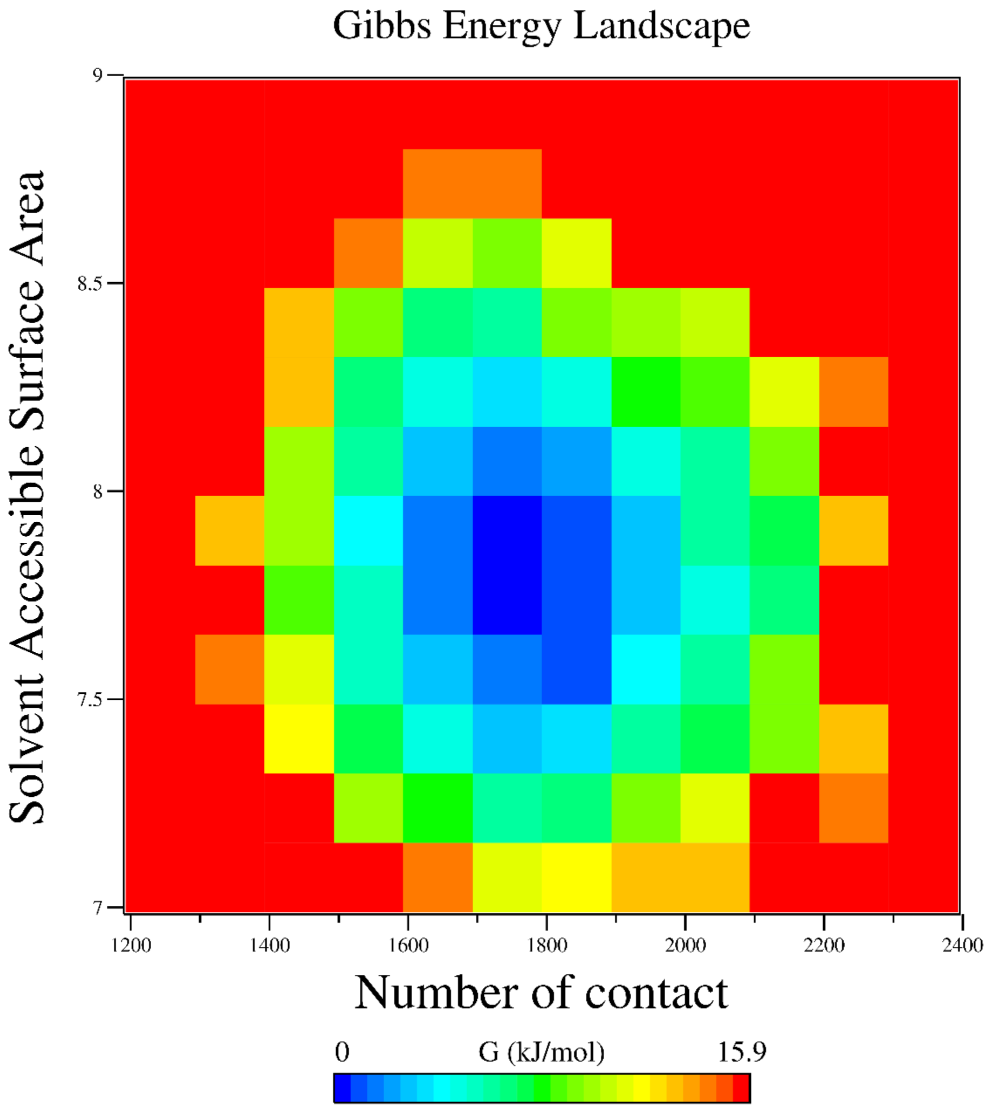

Figure S29: *The free energy landscape (FES) obtained from the last 50 ns of 100 ns MD simulation of compound* ***4c****-EGFR complex*

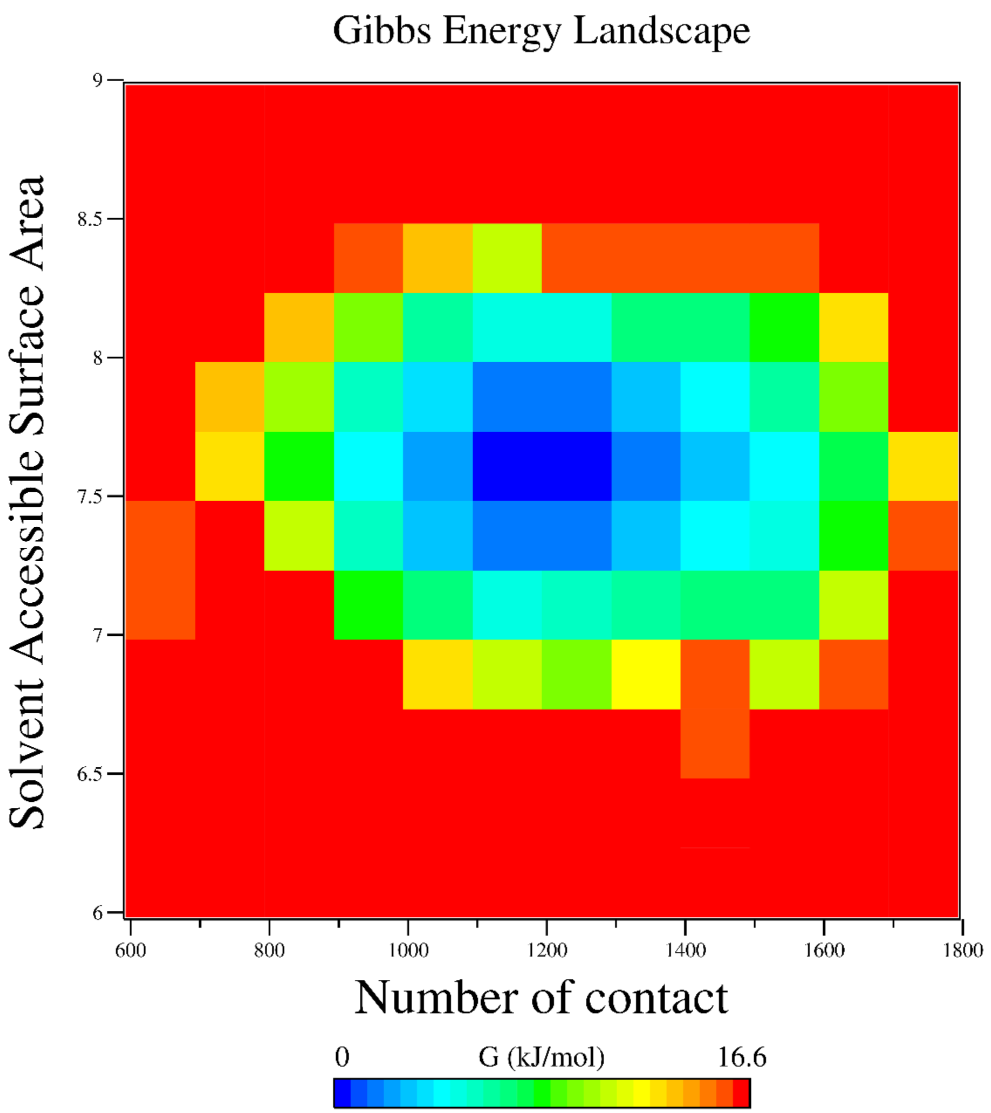

Figure S30: *The free energy landscape (FES) obtained from the last 50 ns of 100 ns MD simulation of compound* ***4f****-EGFR complex*

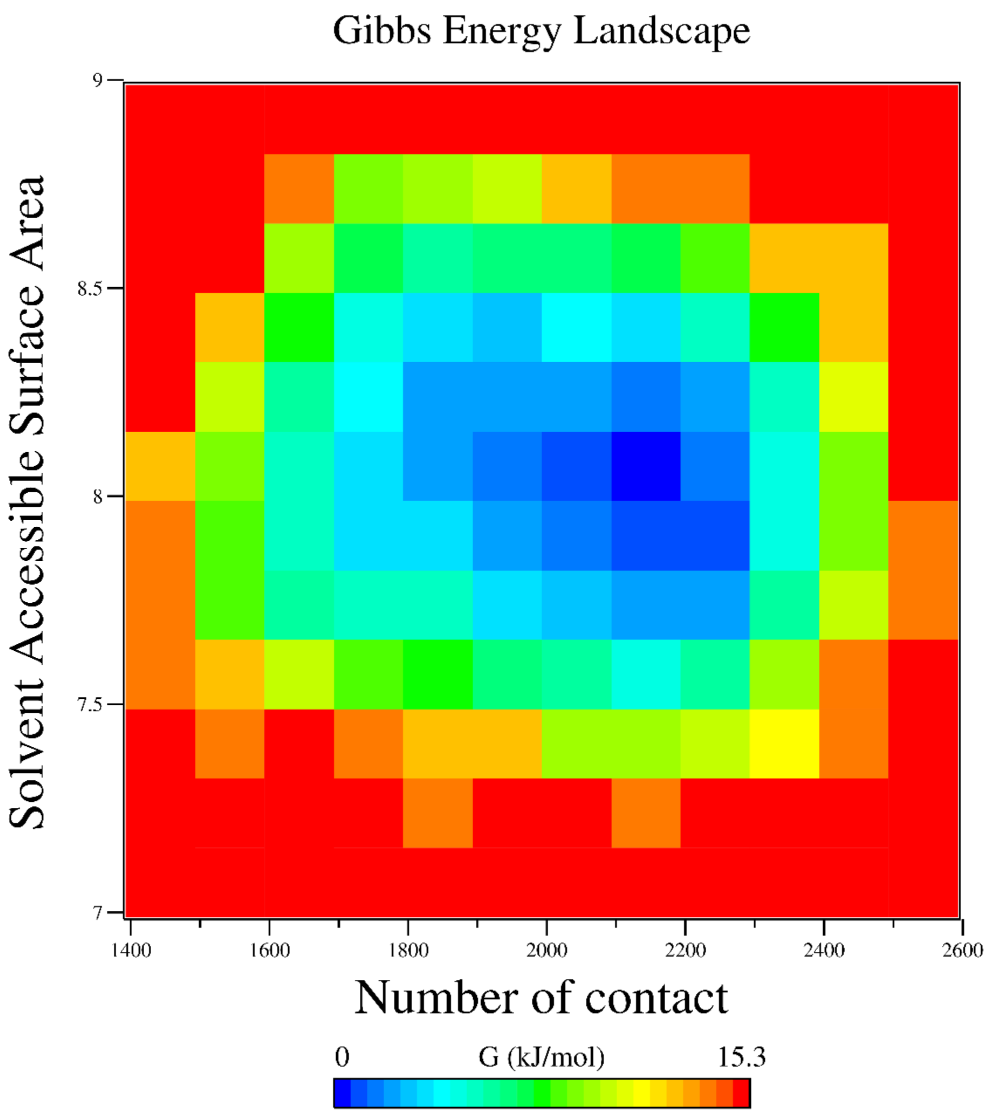

Figure S31: *The free energy landscape (FES) obtained from the last 50 ns of 100 ns MD simulation of compound **4g**-EGFR complex*

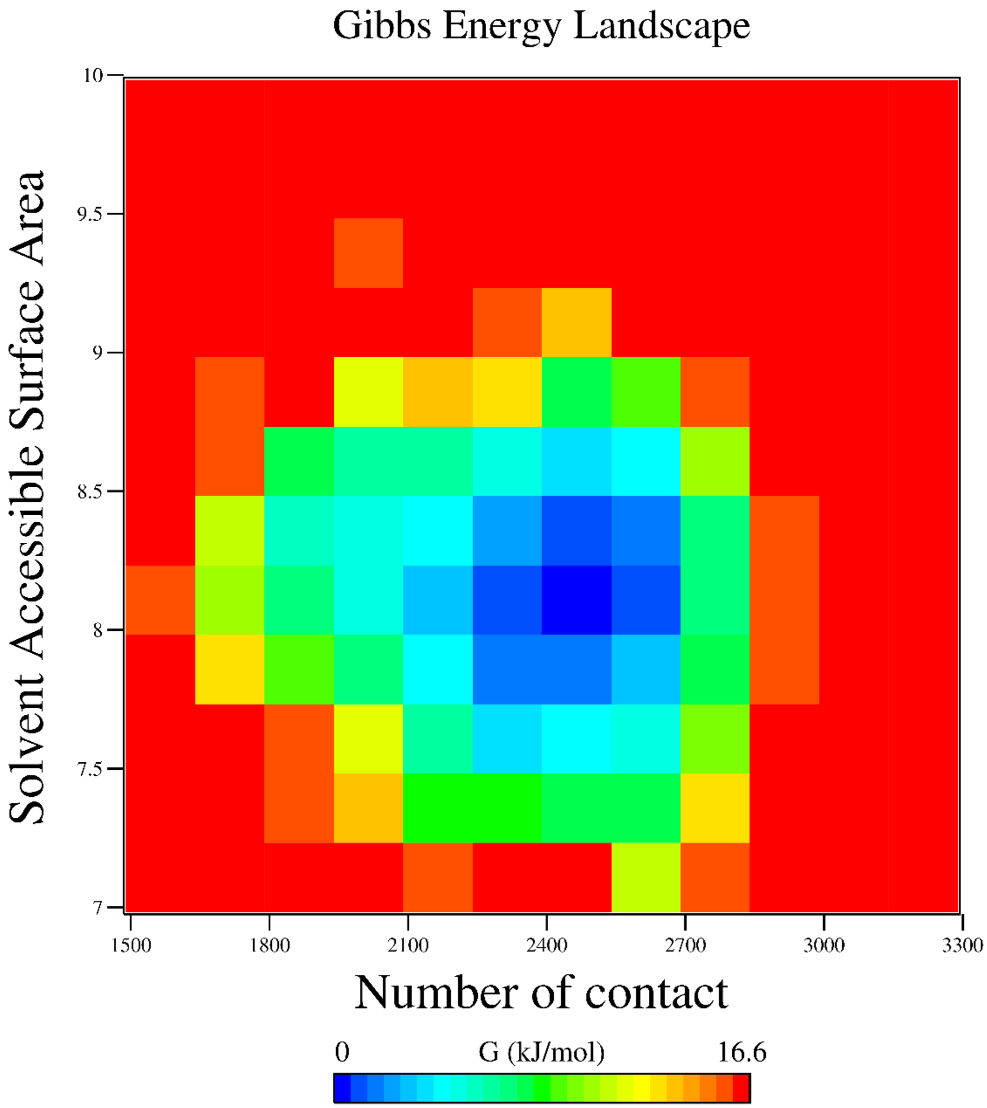

Figure S32: *The free energy landscape (FES) obtained from the last 50 ns of 100 ns MD simulation of compound* ***4h****-EGFR complex*

***Figure S33**: Interactive picture between **compound 1-3a** and EGFR residues*

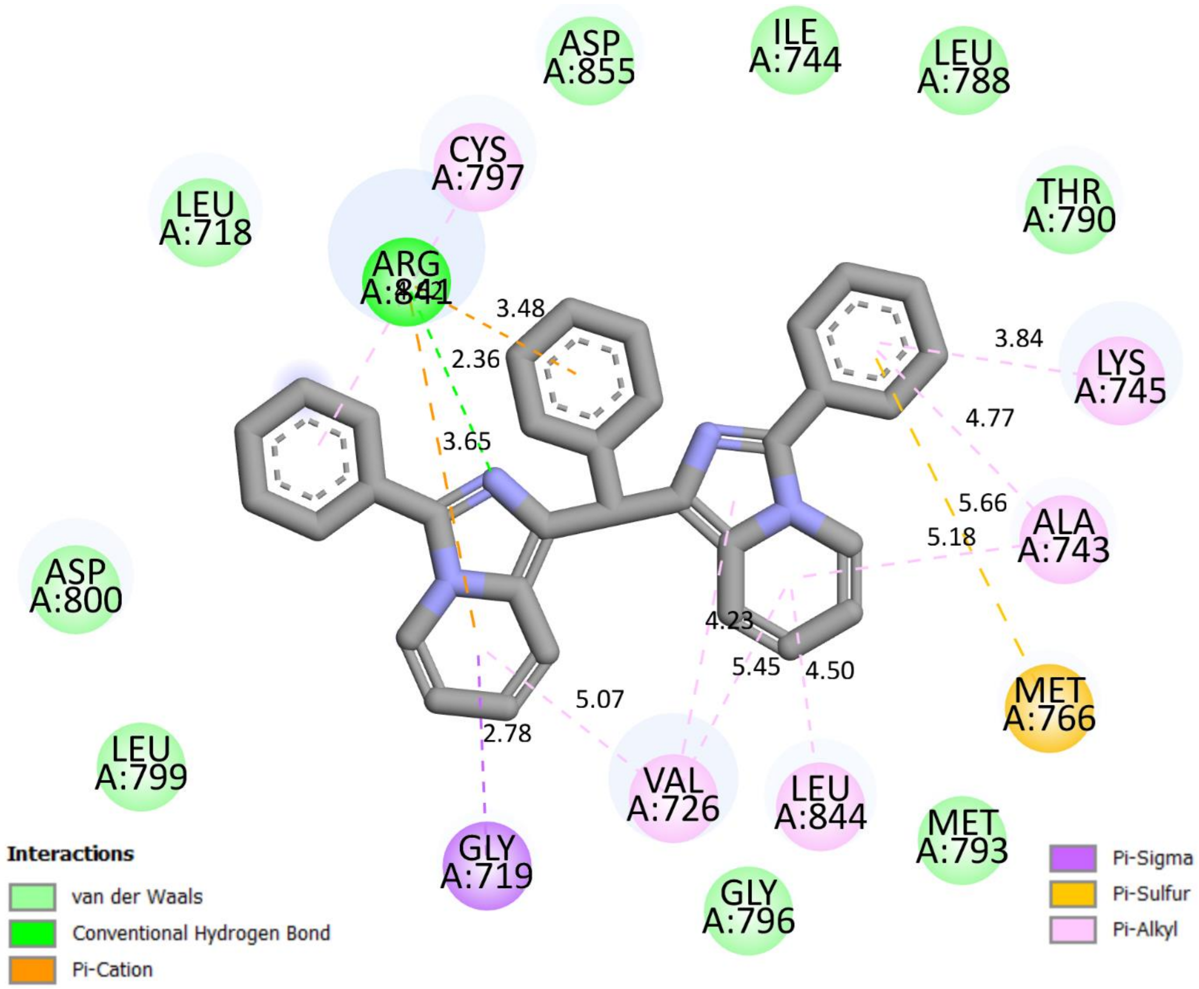

Figure S34: *Interactive picture between* ***compound 2-3b*** *and EGFR*

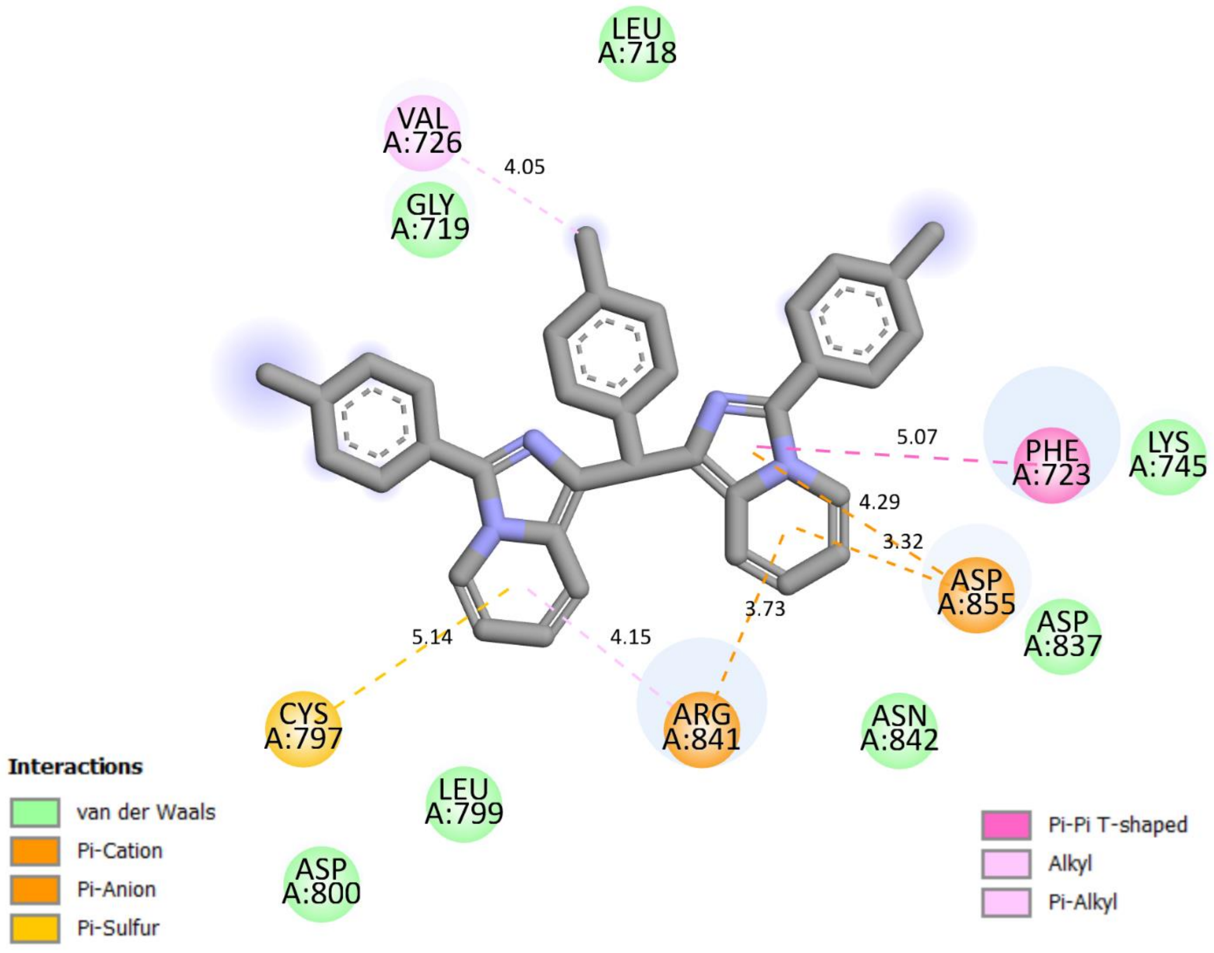

*Figure S35: Interactive picture between **compound 4-3d** and EGFR residues*

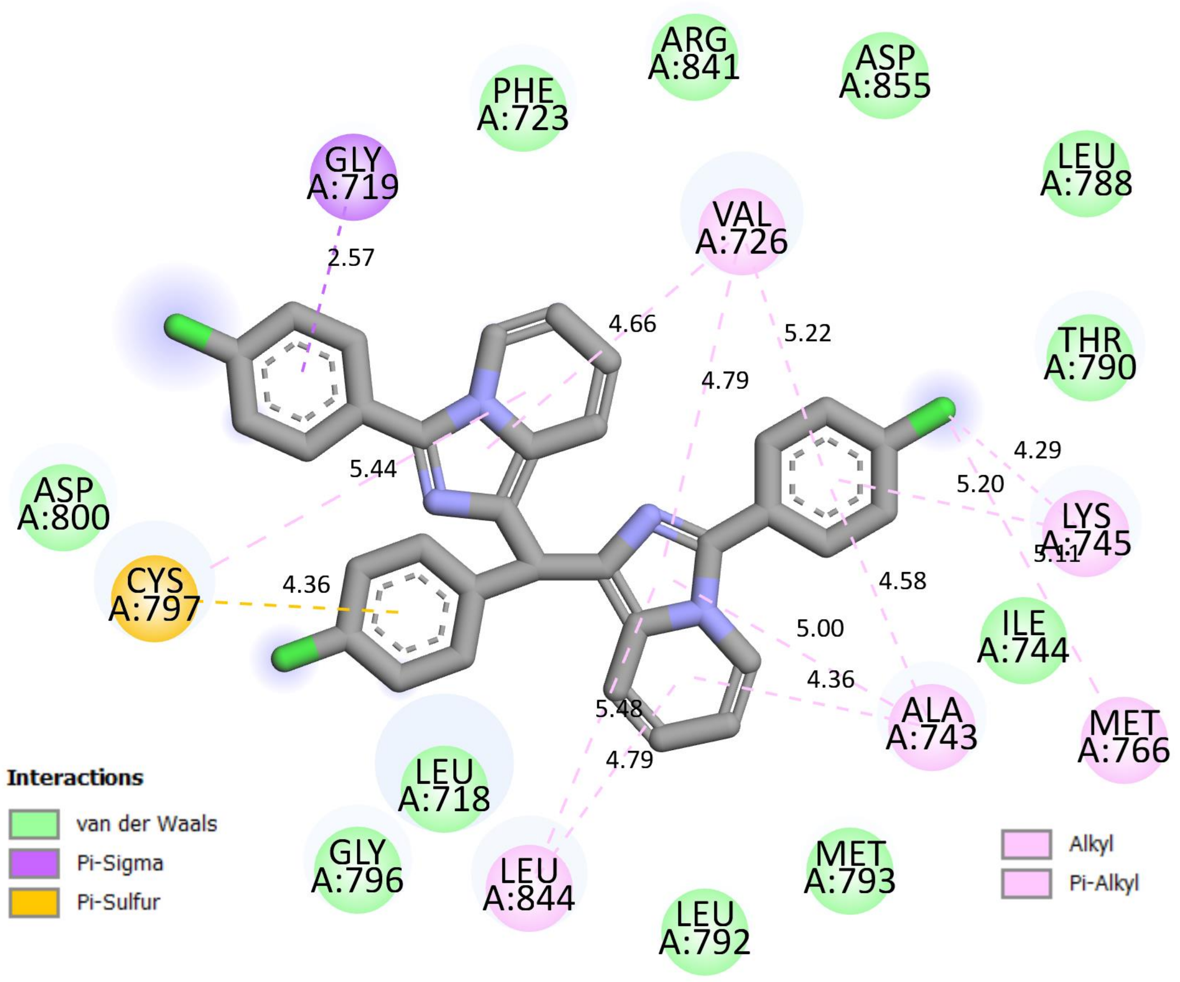

Figure S36: *Interactive picture between* ***compound 5-3e*** *and EGFR residues*

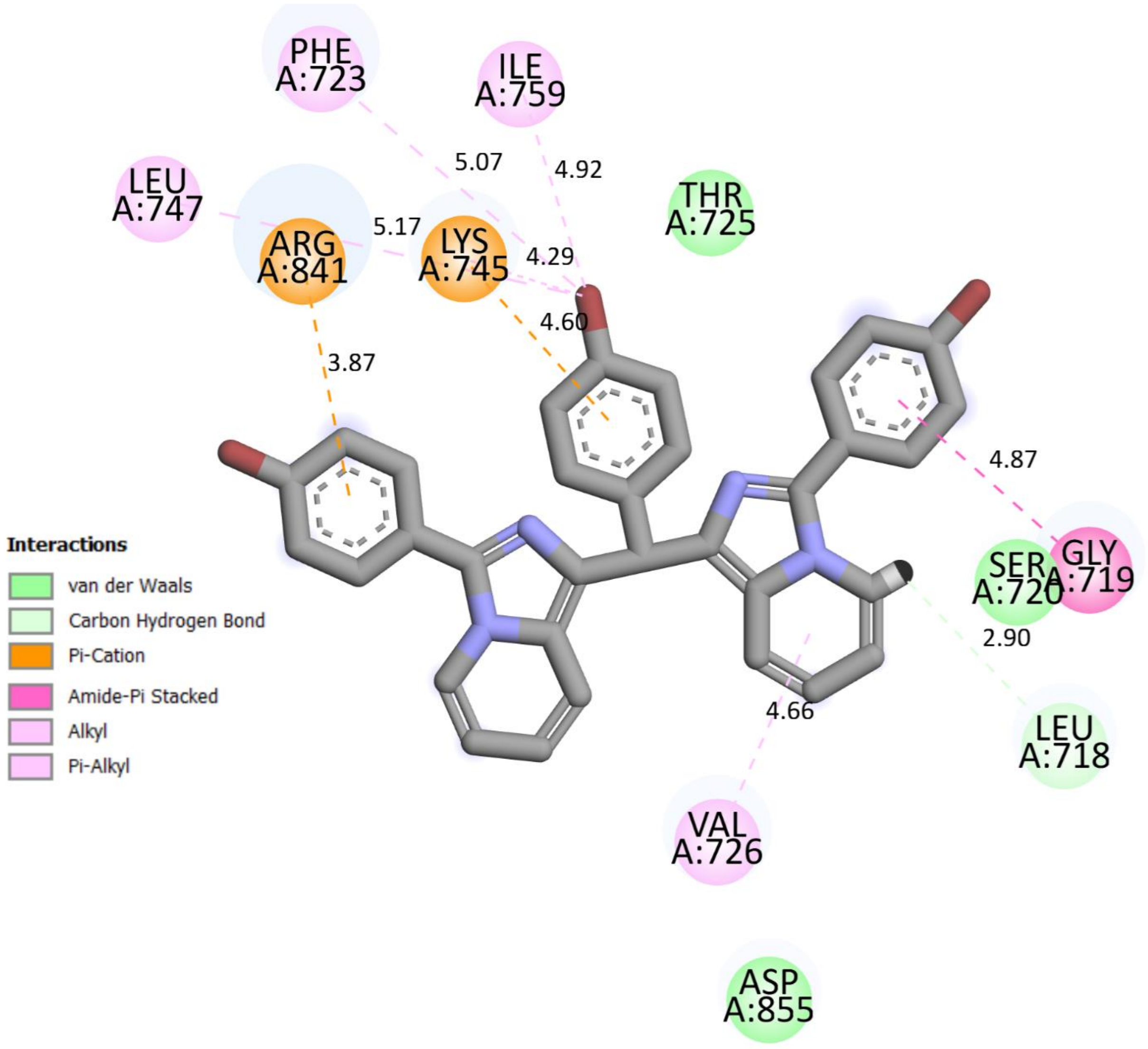

Figure S37: *Interactive picture between* ***compound 6-3f*** *and EGFR residues*

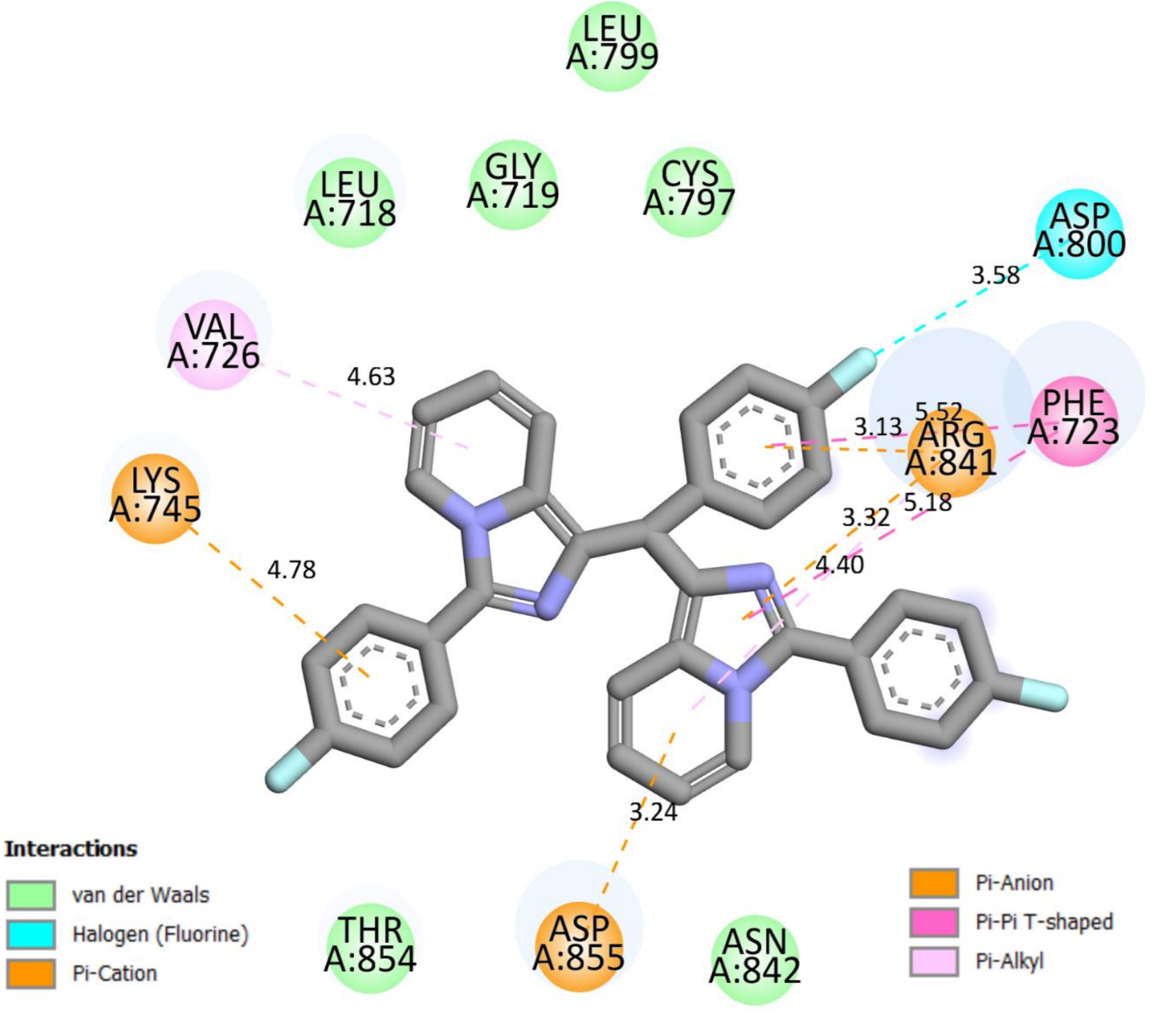

***Figure S38**: Interactive picture between **compound 7-3g** and EGFR residues*

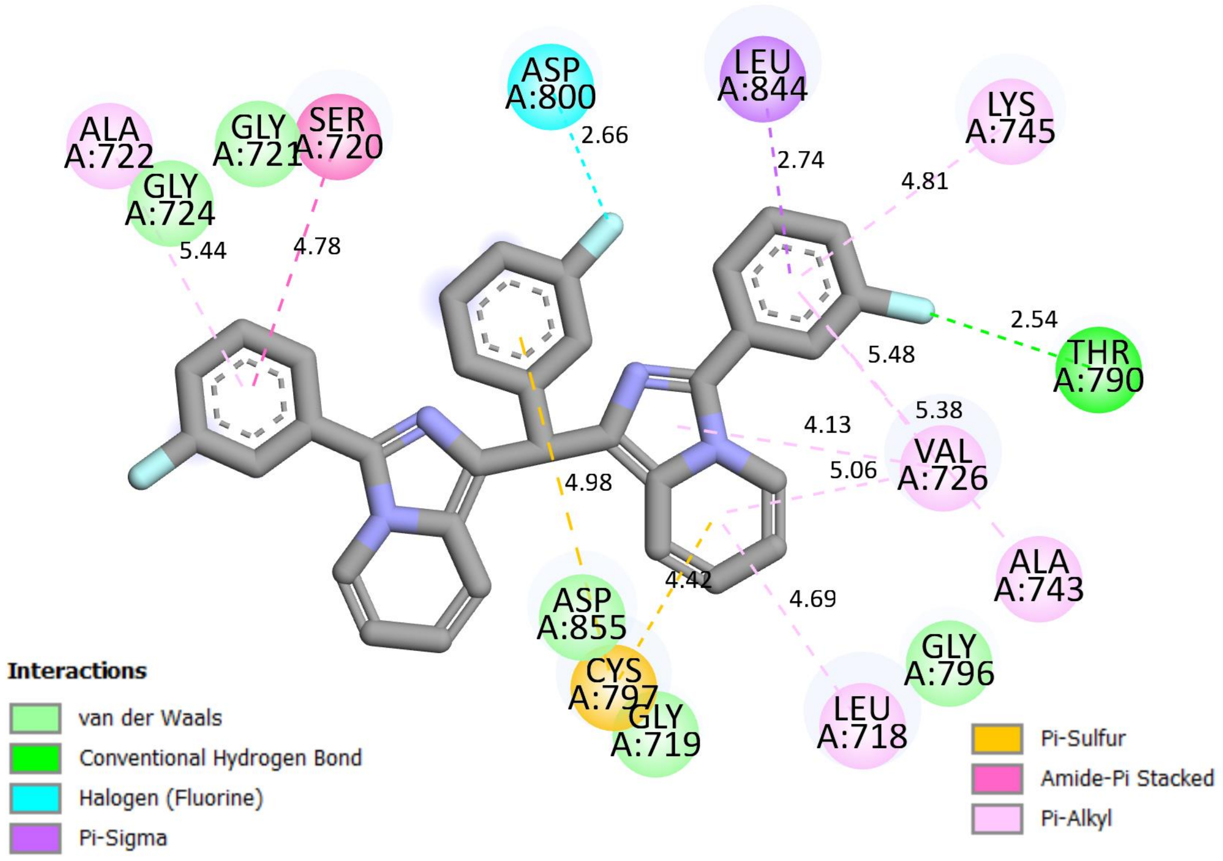

***Figure S39**: Interactive picture between **compound 8-3h** and EGFR residues*

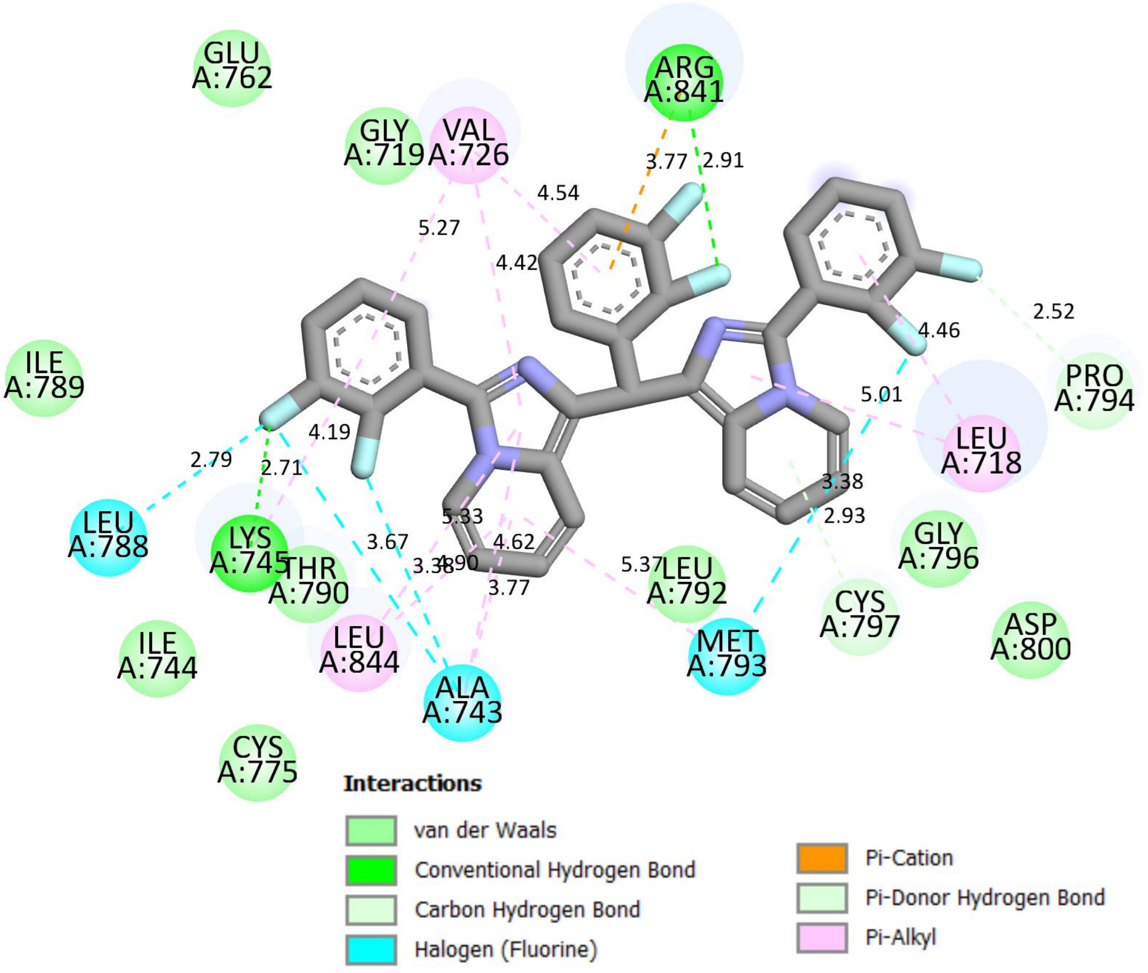

***Figure S40**: Interactive picture between **compound 9-3i** and EGFR residues*

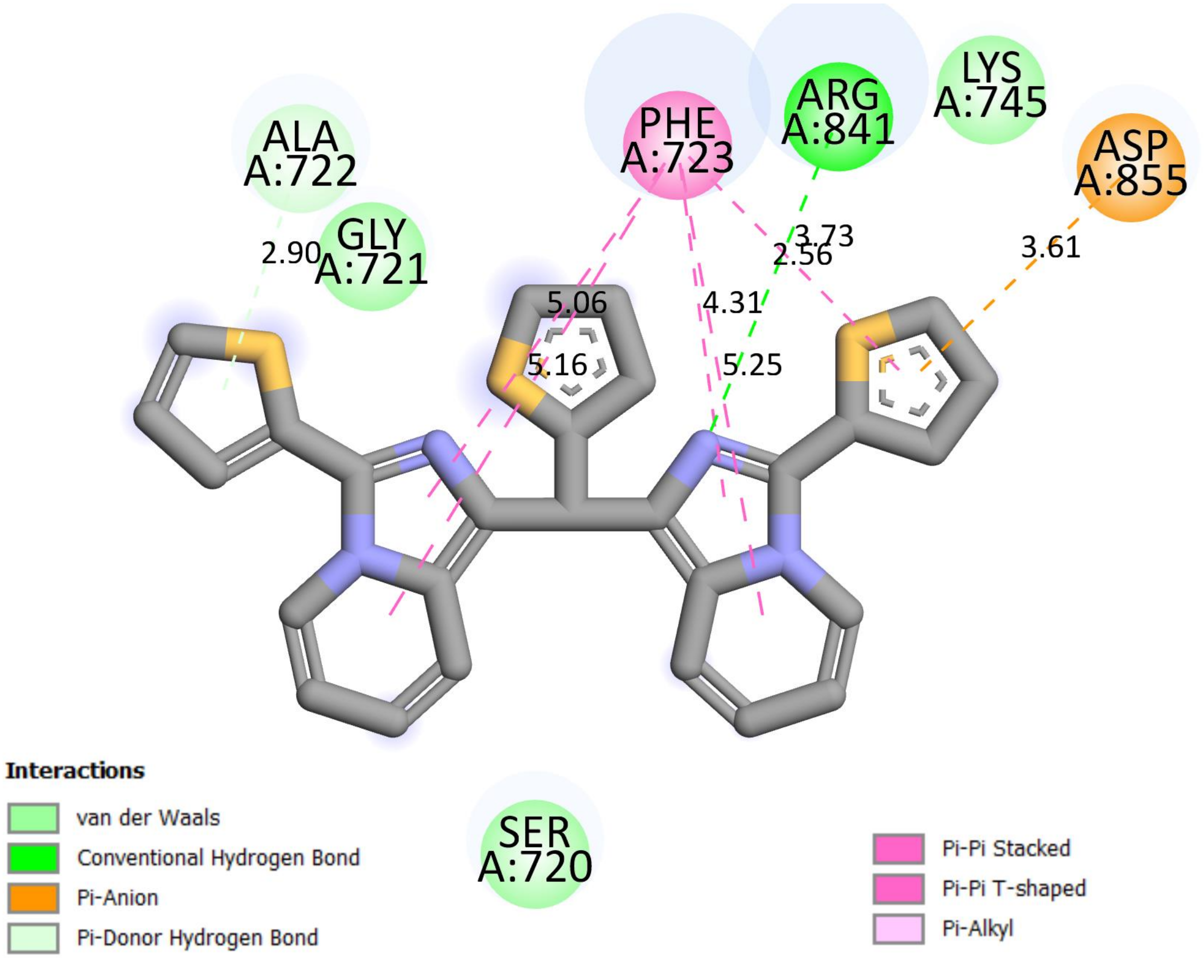
ALA A:722
GLY A:721
PHE A:723
ARG A:841
LYS A:745
ASP A:855
SER A:720
2.90
3.73
2.56
3.61
5.06
5.16
4.31
5.25
Interactions
van der Waals
Conventional Hydrogen Bond
Pi-Anion
Pi-Donor Hydrogen Bond
Pi-Pi Stacked
Pi-Pi T-shaped
Pi-Alkyl

Figure S41*: Interactive picture between* ***compound 11-3k*** *and EGFR*

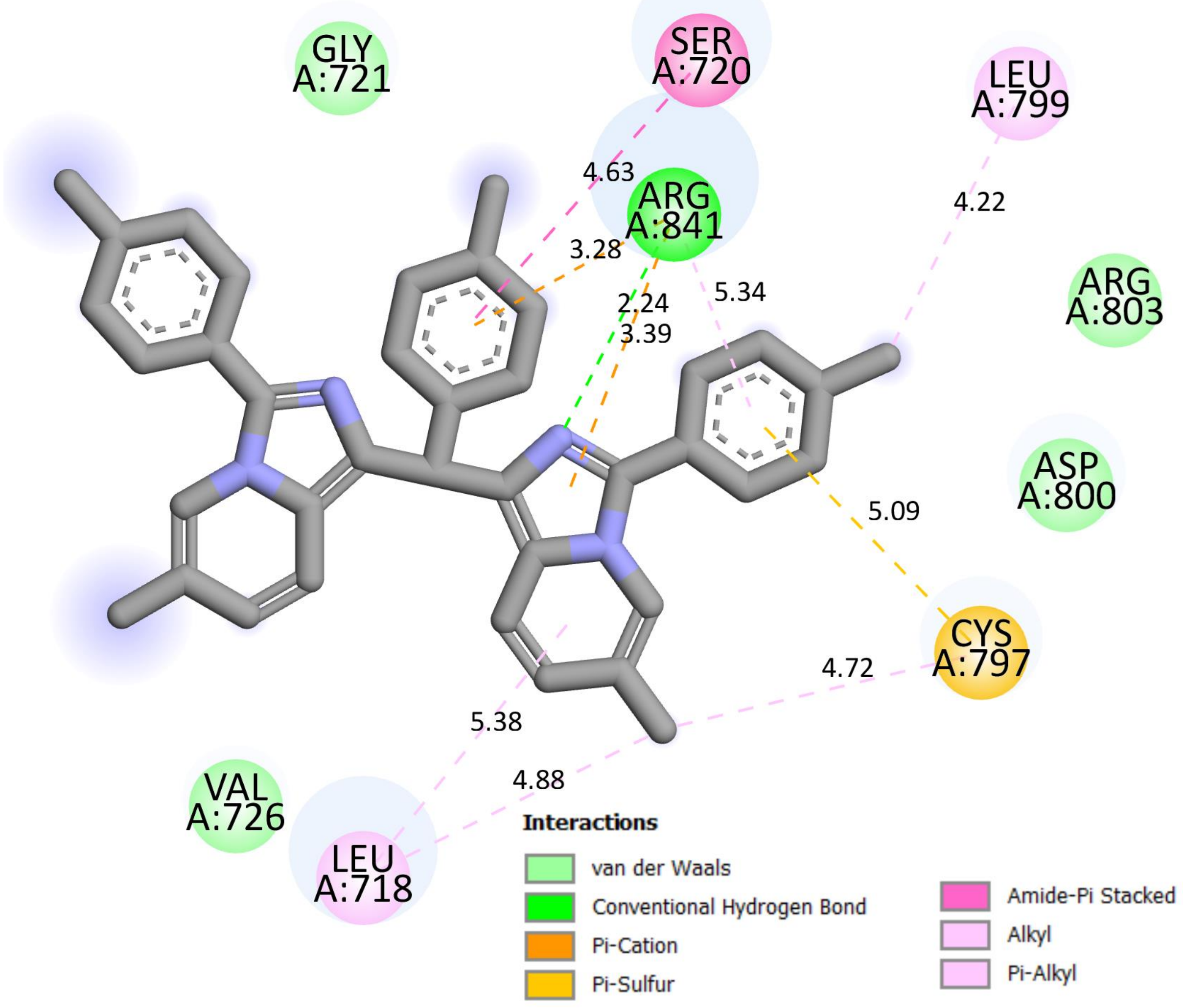

***Figure S42**: Interactive picture between **compound 12-4a** and EGFR residues*

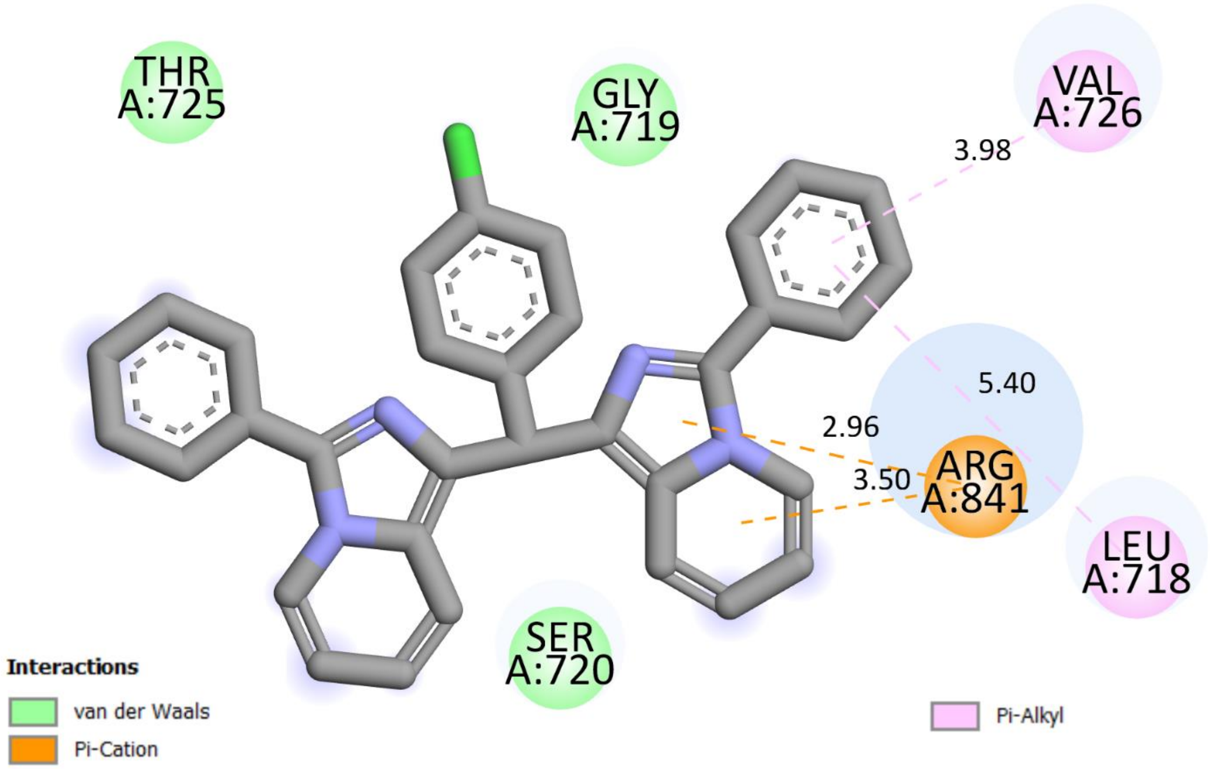

***Figure S43**: Interactive picture between **compound 13-4b** and EGFR*

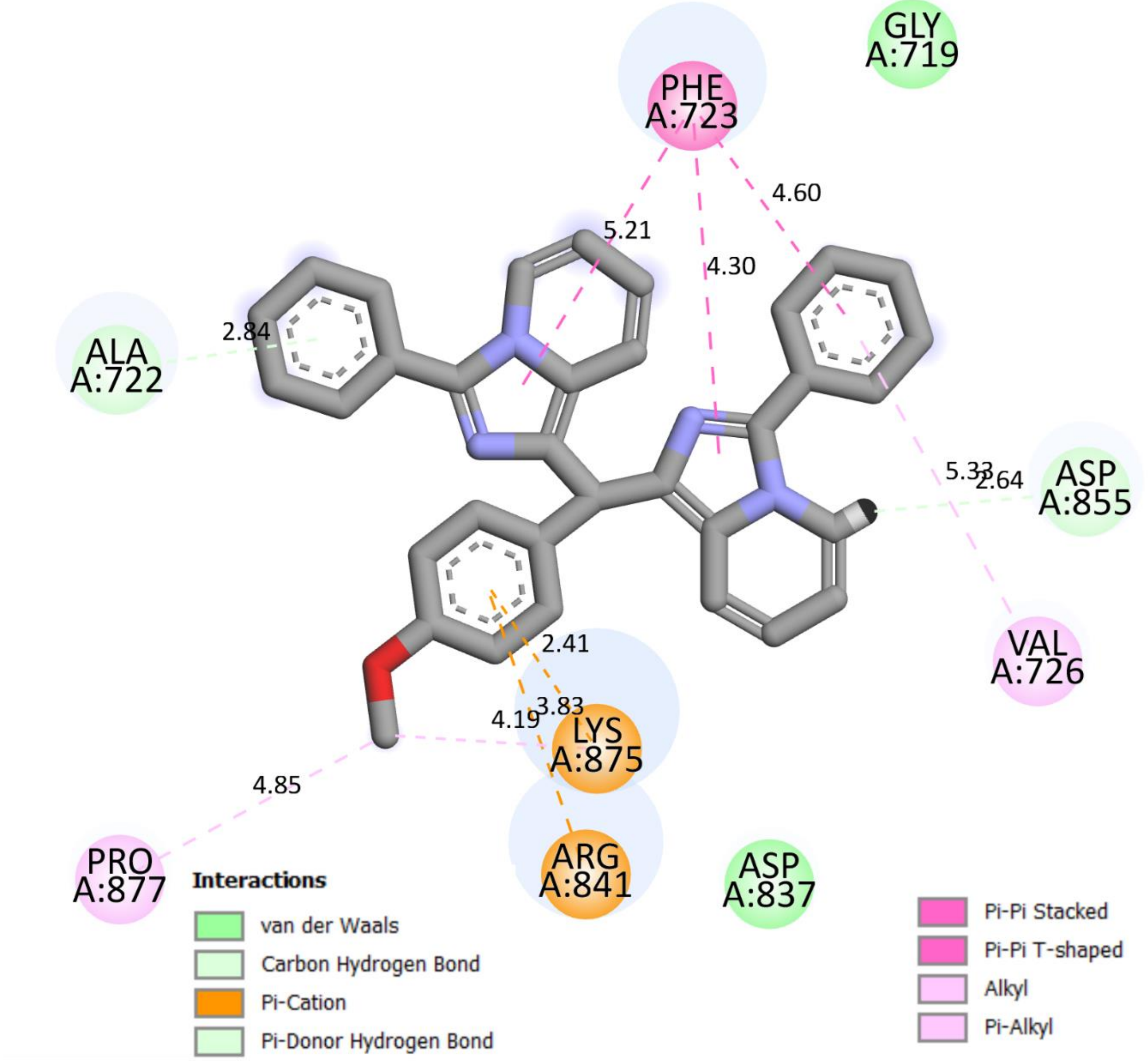

***Figure S44**: Interactive picture between **compound 14-4c** and EGFR residues*

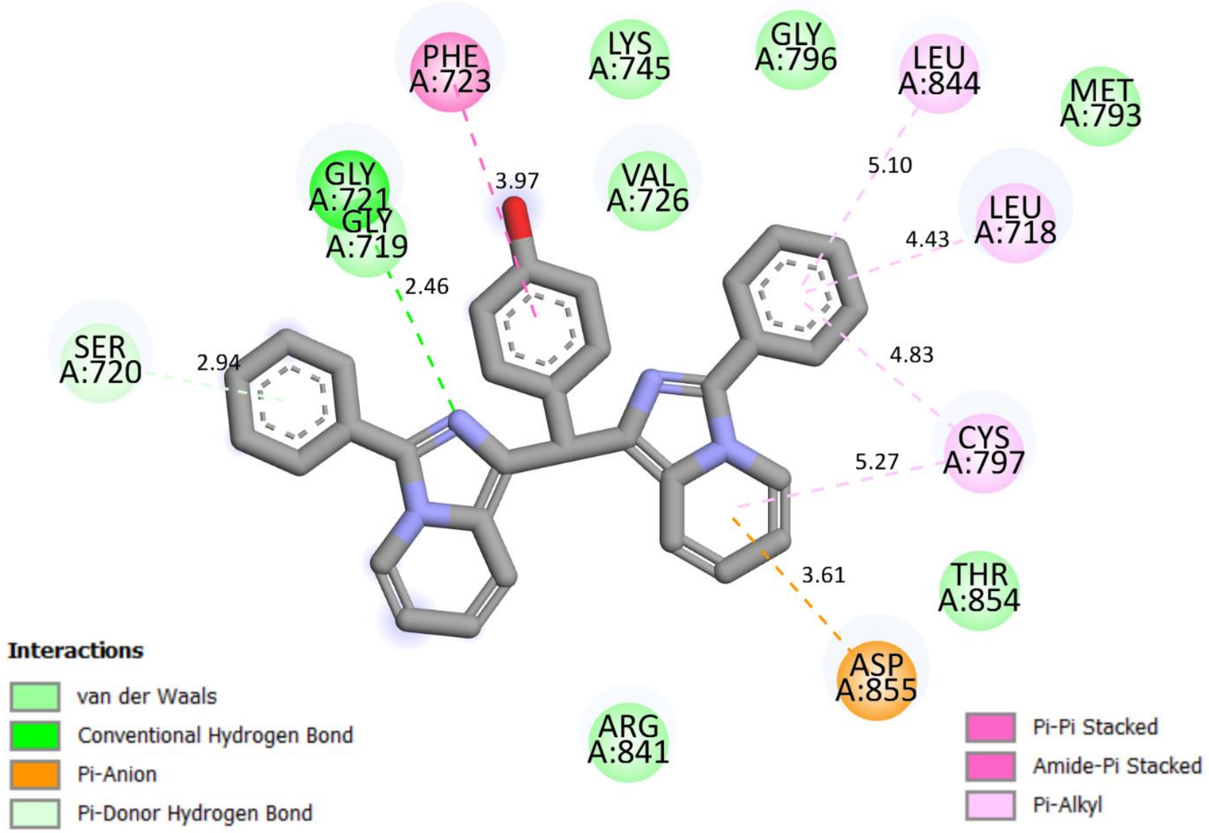

***Figure S45**: Interactive picture between **compound 16-4f** and EGFR residues*

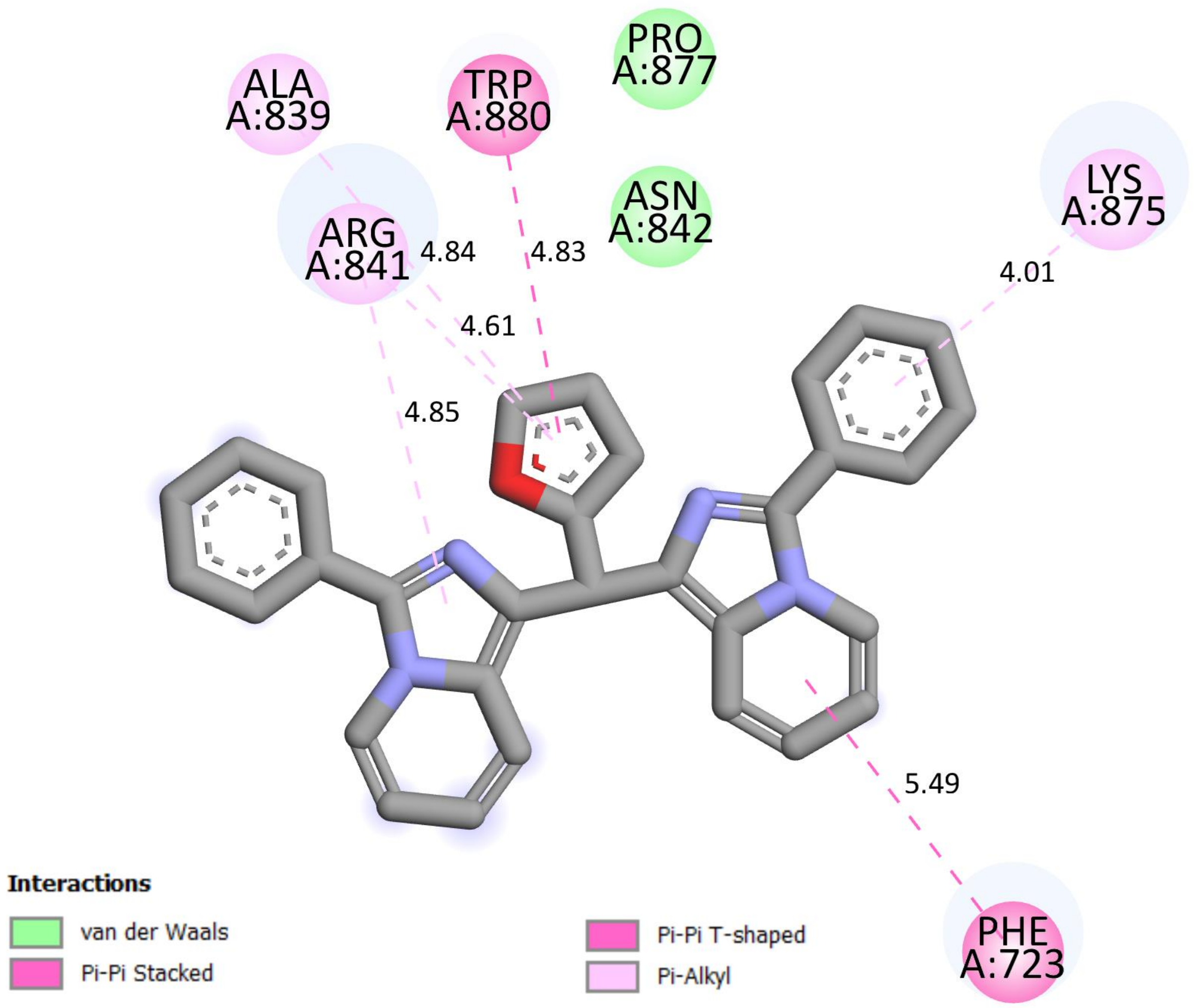

Figure S46: *Interactive picture between* ***compound 17-4g*** *and EGFR residues*

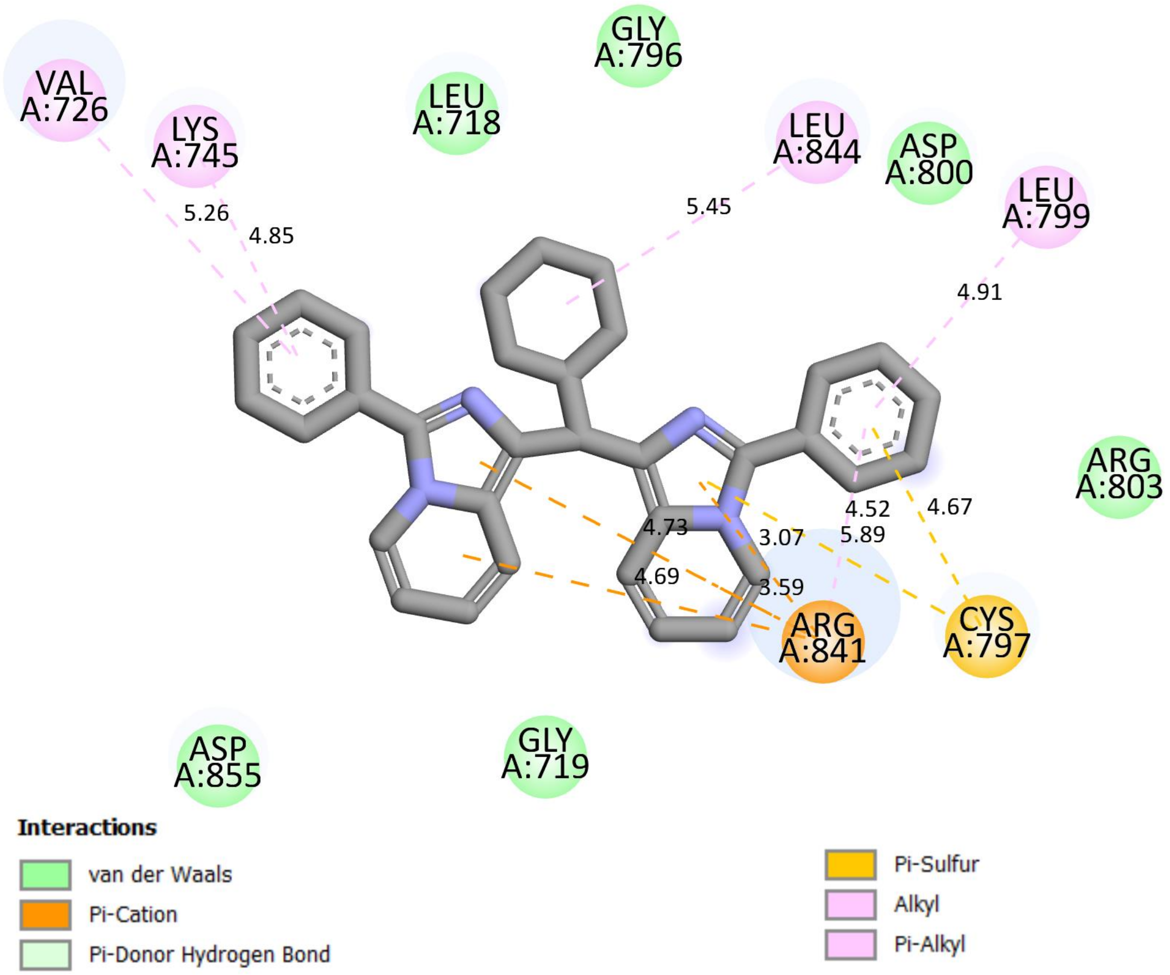

***Figure S47**: Interactive picture between **compound 18-4h** and EGFR residues*

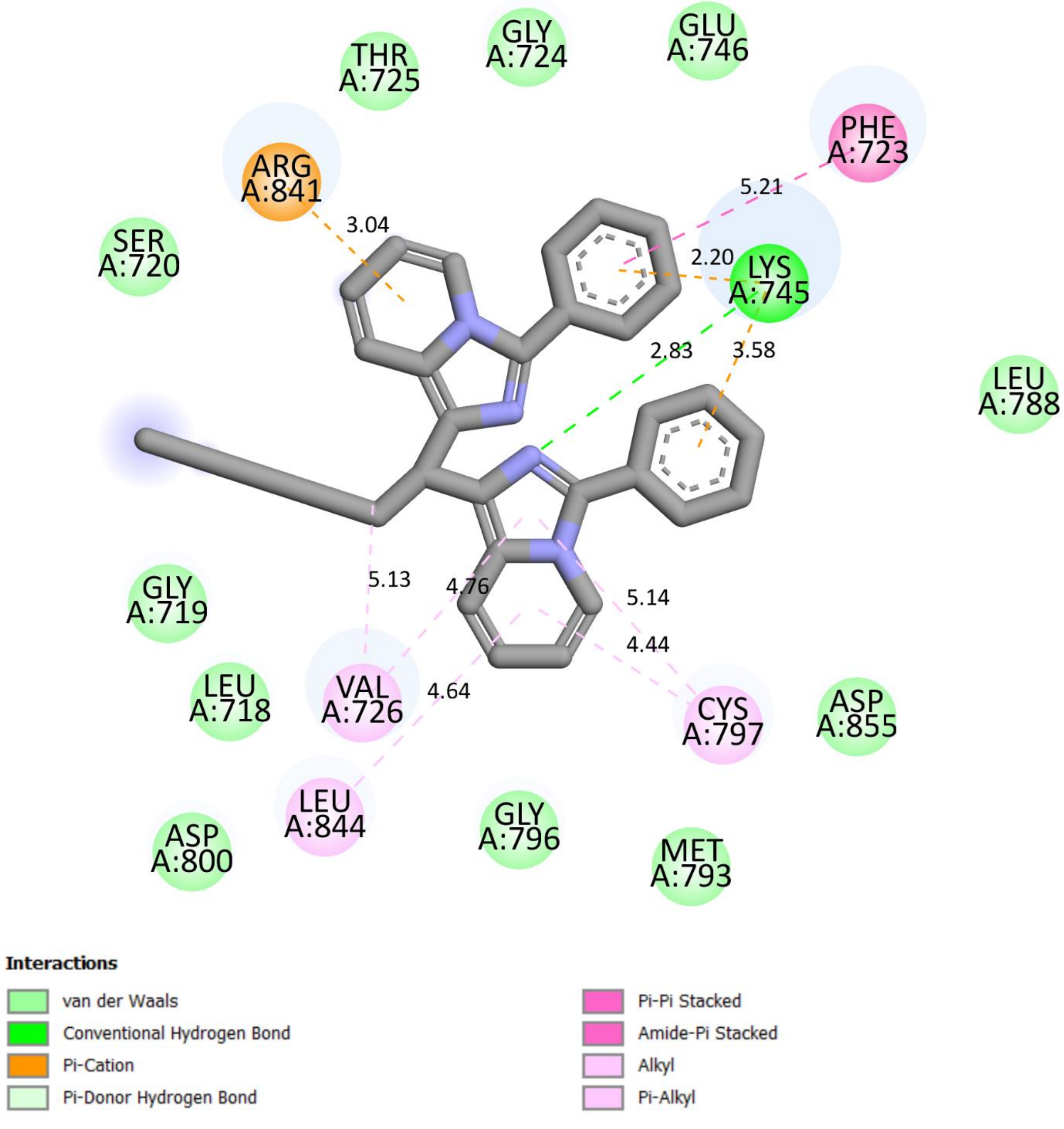

Figure S48: *Interactive picture between* ***1M17*** *and EGFR residues*

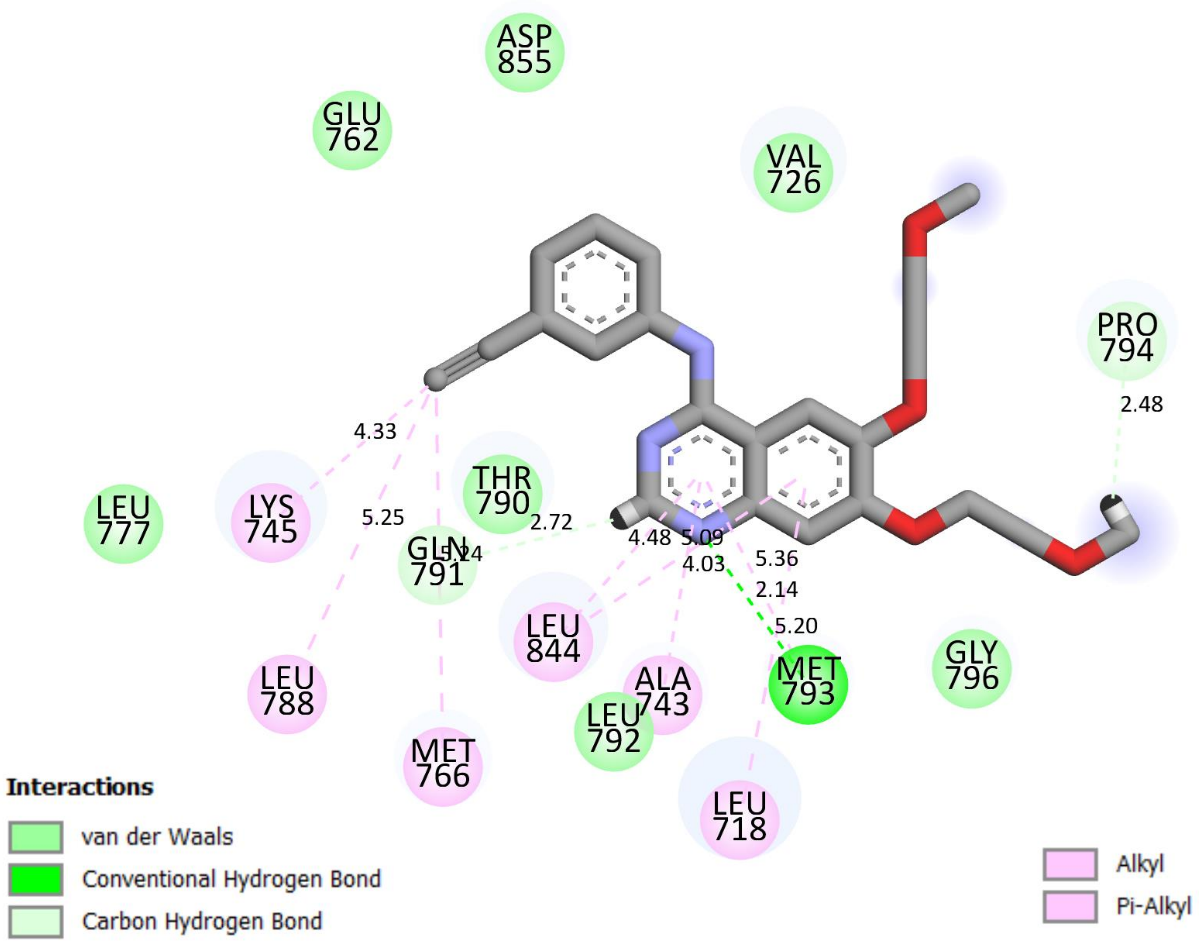

Figure S49: *Interactive picture between* ***4ZAU*** *and EGFR residues*

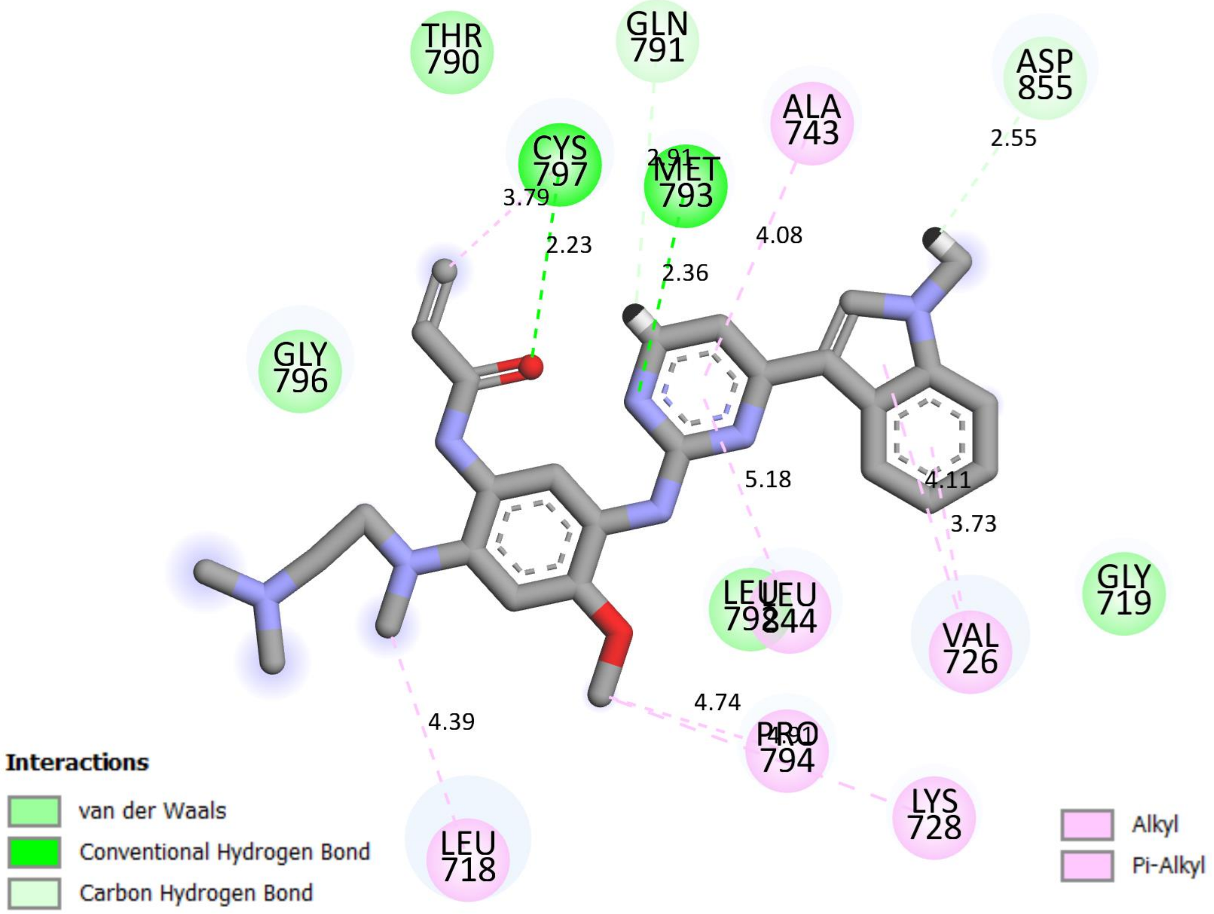

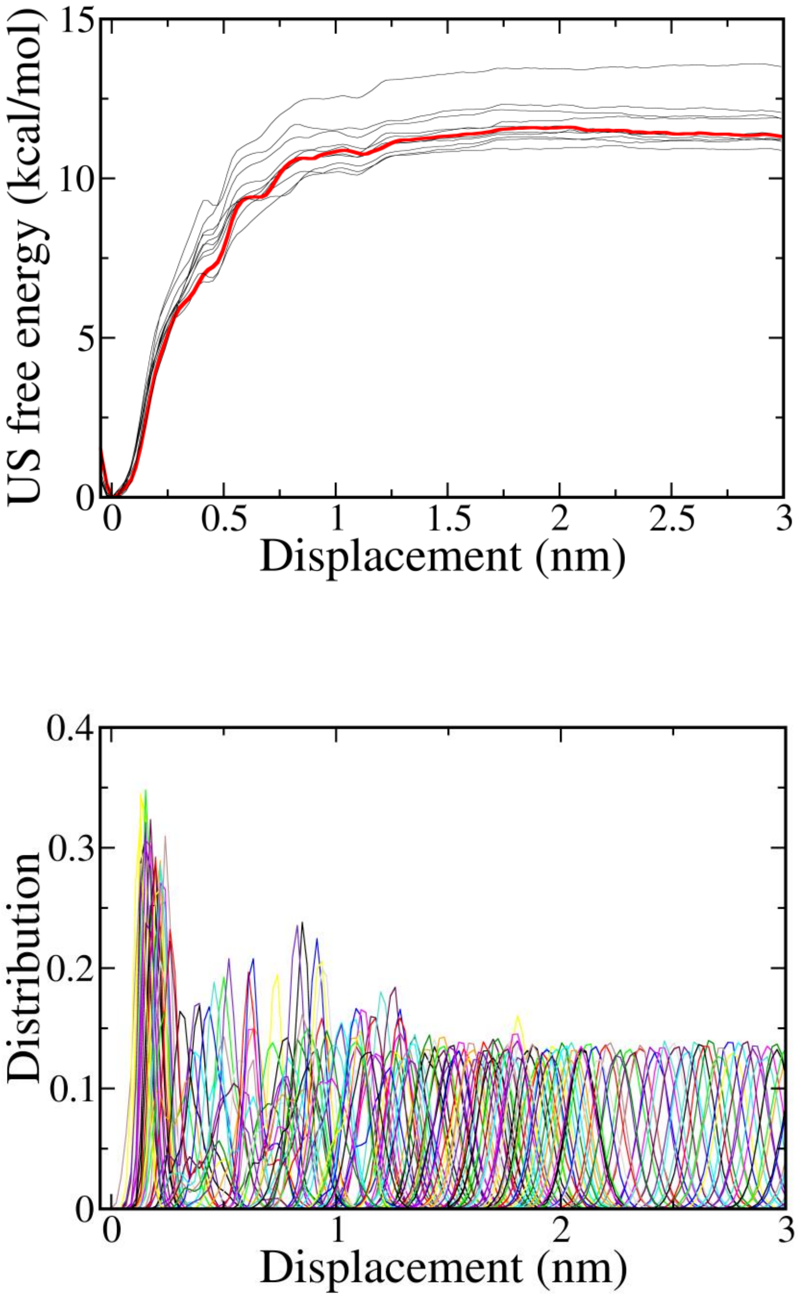

Figure S53: Potential of mean force (upper) and diagram (lower) obtained from umbrella sampling method of **compound 3g** – EGFR complex

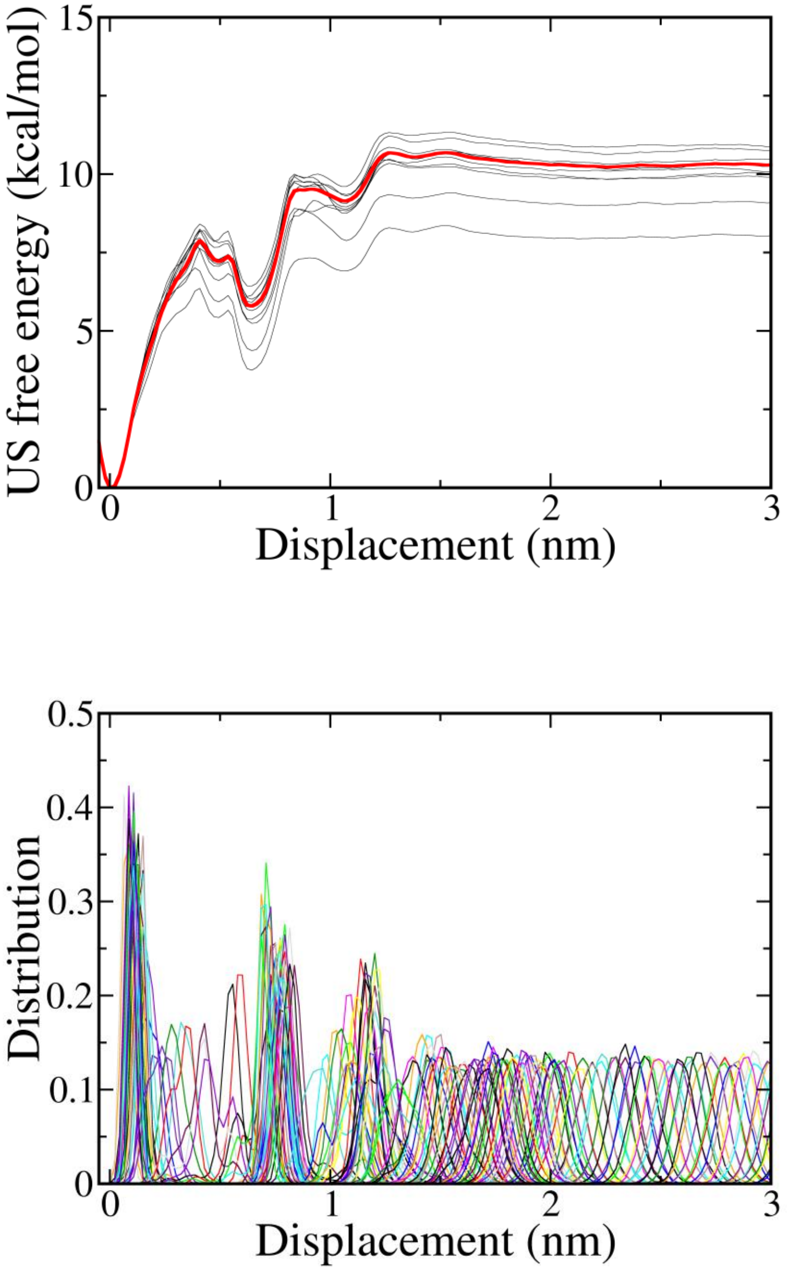

Figure S54: Potential of mean force (upper) and diagram (lower) obtained from umbrella sampling method of **compound 3h** – EGFR complex

Umbrella potential

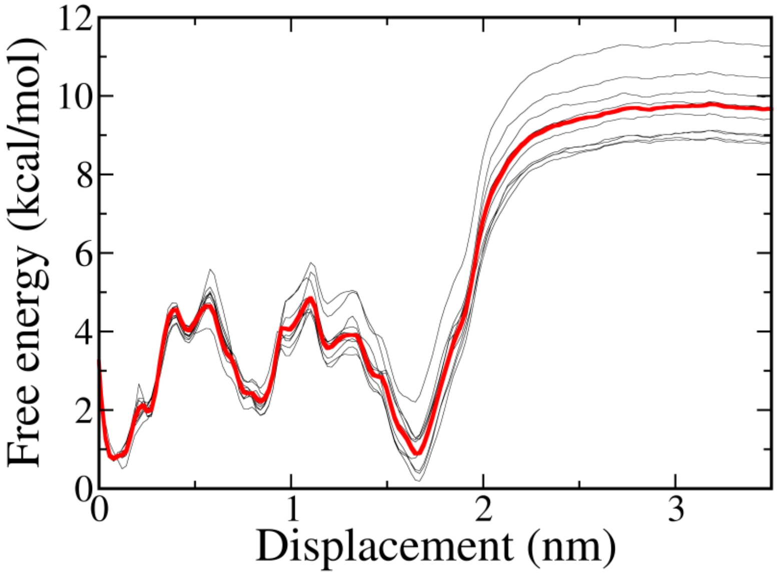

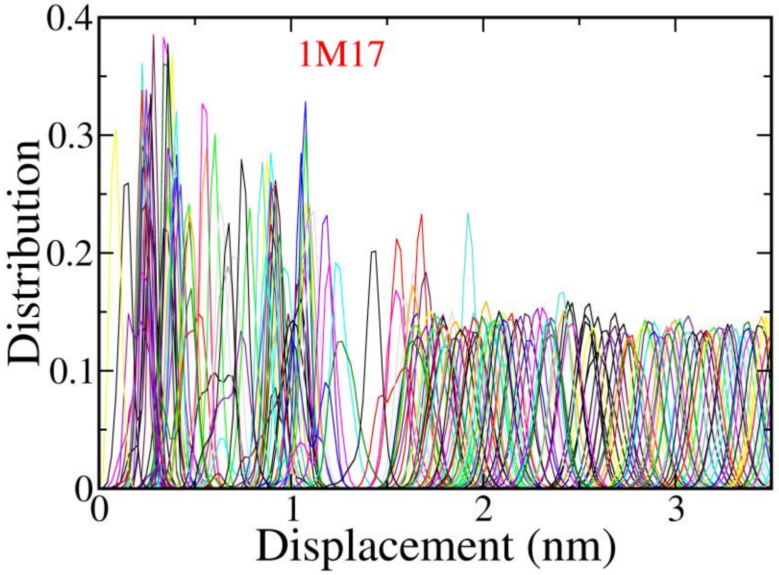

Figure S55: Potential of mean force (upper) and diagram (lower) obtained from umbrella sampling method of **erlotinib –** EGFR complex

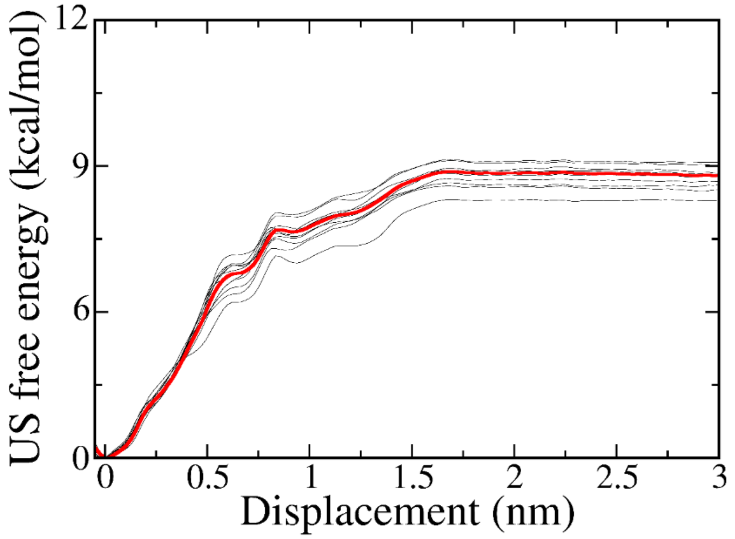

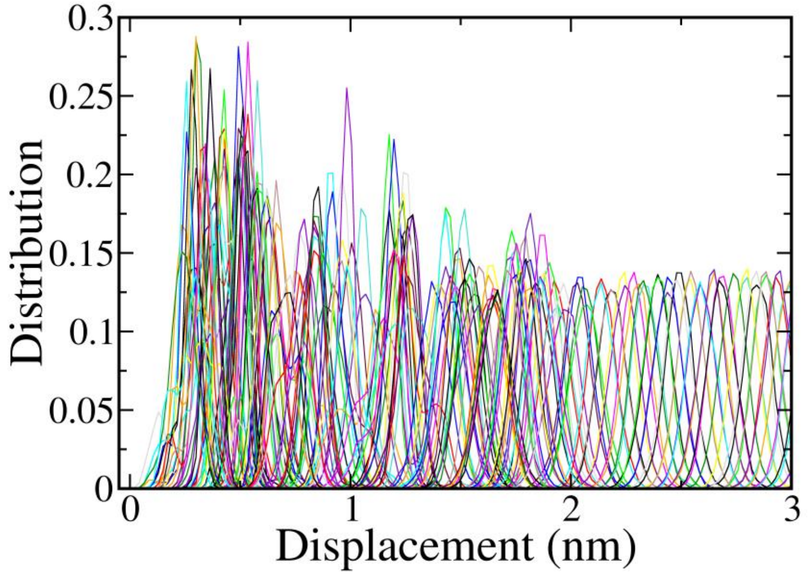

Figure S56: Potential of mean force (upper) and diagram (lower) obtained from umbrella sampling method of **osimertinib** – EGFR complex

Figure 57: *The contact map between 5 sub – groups of* ***compound*** *and 9 key residues of EGFR tyrosine domain.*

DATA are shown in case of ***15 compounds.***

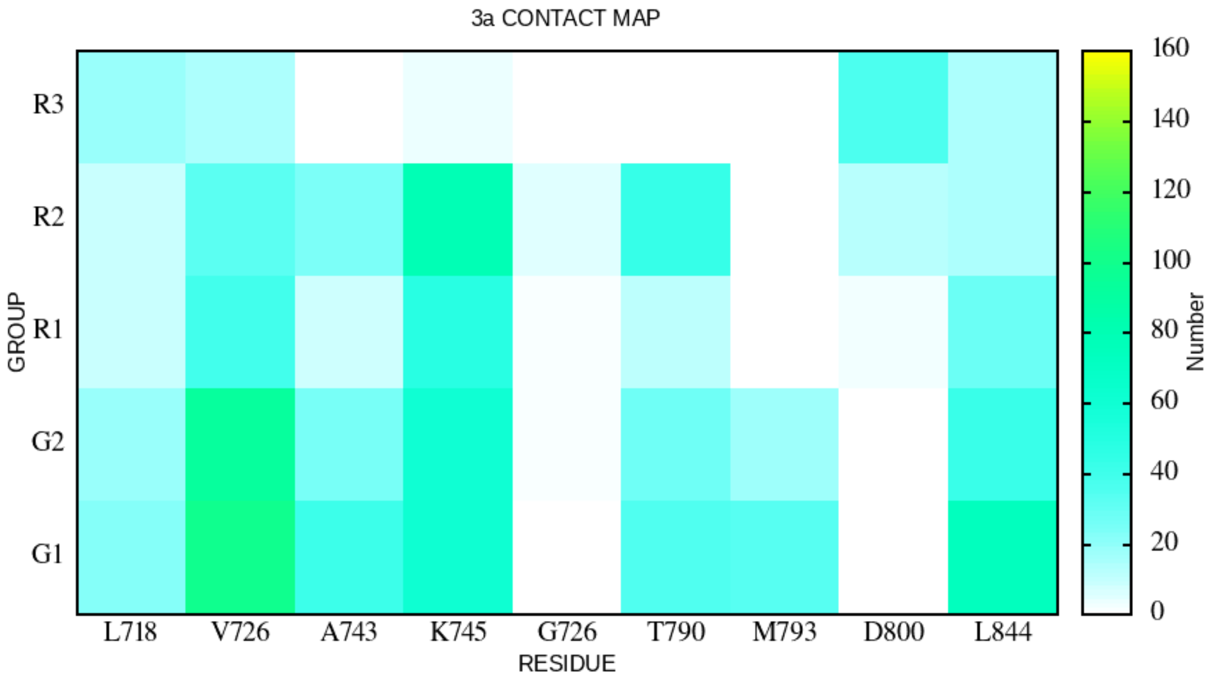

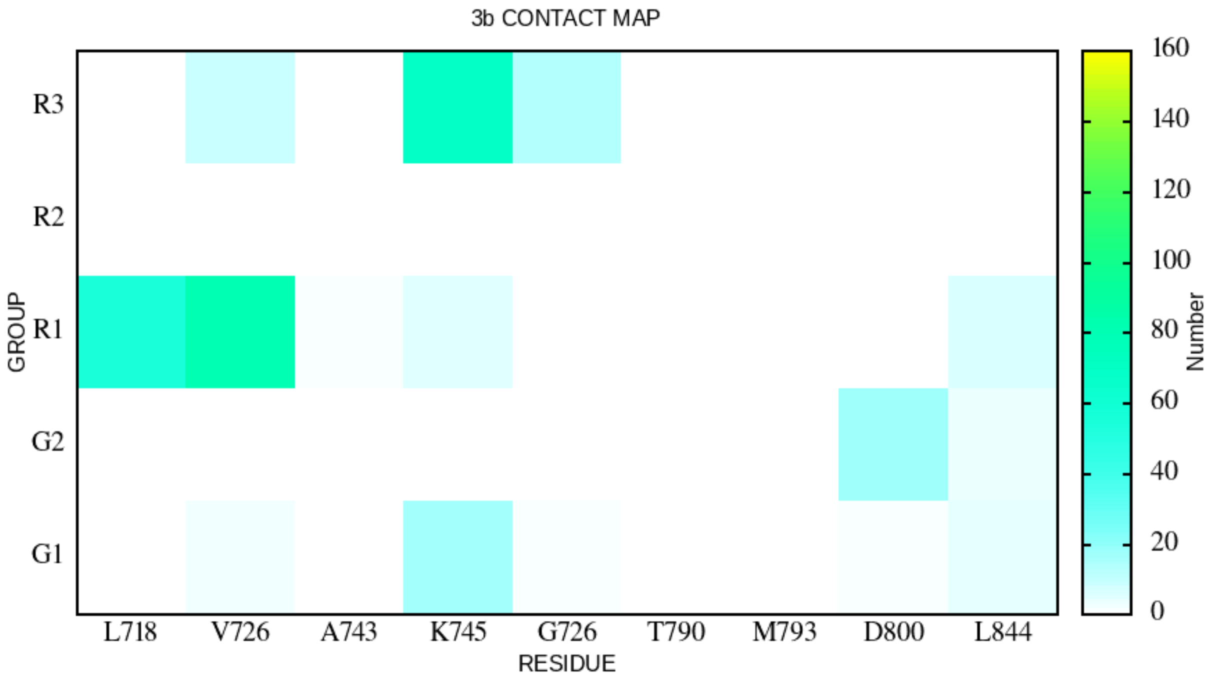
3b CONTACT MAP
R3
R2
R1
G2
G1
GROUP
L718
V726
A743
K745
G726
T790
M793
D800
L844
RESIDUE
160
140
120
100
80
60
40
20
0
Number

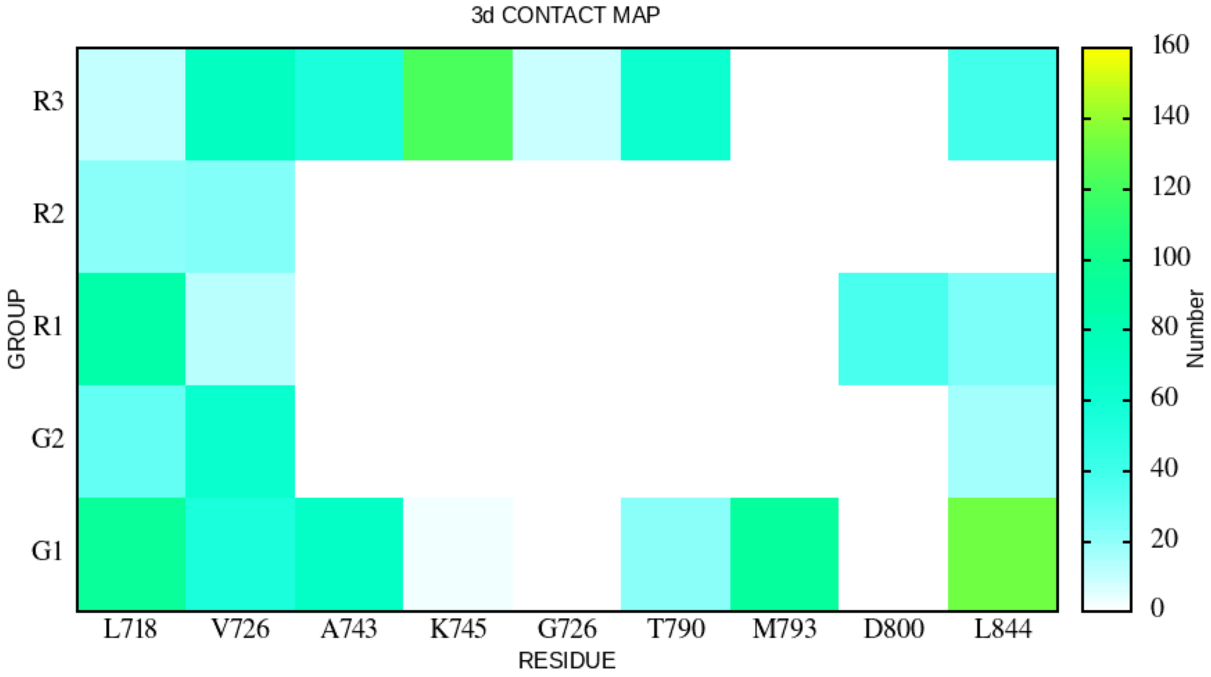
3d CONTACT MAP
R3
R2
R1
G2
G1
GROUP
L718
V726
A743
K745
G726
T790
M793
D800
L844
RESIDUE
160
140
120
100
80
60
40
20
0
Number

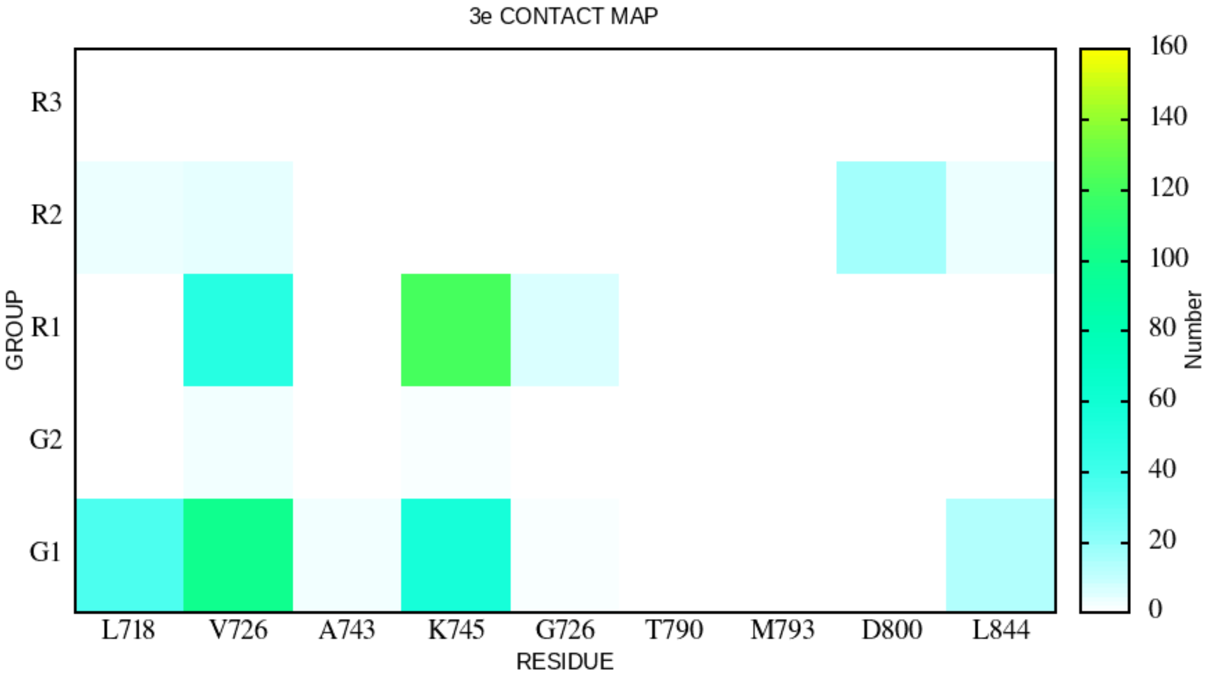
3e CONTACT MAP
R3
R2
R1
G2
G1
GROUP
L718 V726 A743 K745 G726 T790 M793 D800 L844
RESIDUE
160 140 120 100 80 60 40 20 0
Number

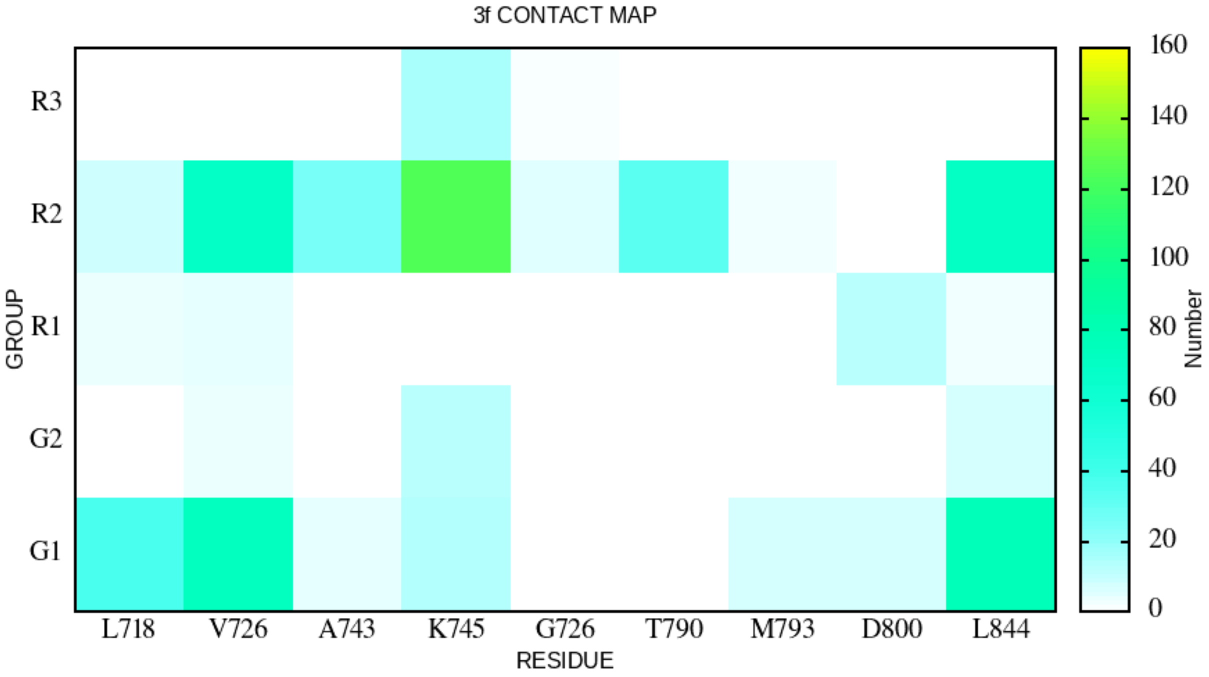
3f CONTACT MAP
R3
R2
R1
G2
G1
GROUP
L718 V726 A743 K745 G726 T790 M793 D800 L844
RESIDUE
160 140 120 100 80 60 40 20 0
Number

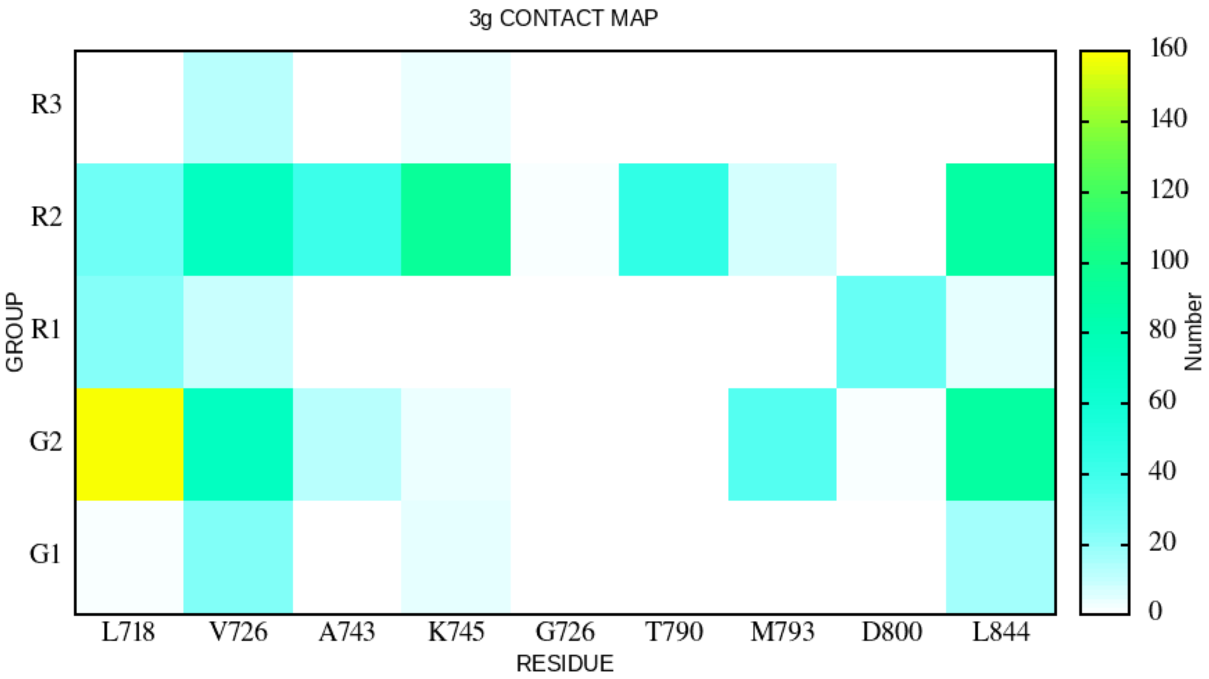
3g CONTACT MAP
R3
R2
R1
G2
G1
GROUP
L718
V726
A743
K745
G726
T790
M793
D800
L844
RESIDUE
160
140
120
100
80
60
40
20
0
Number

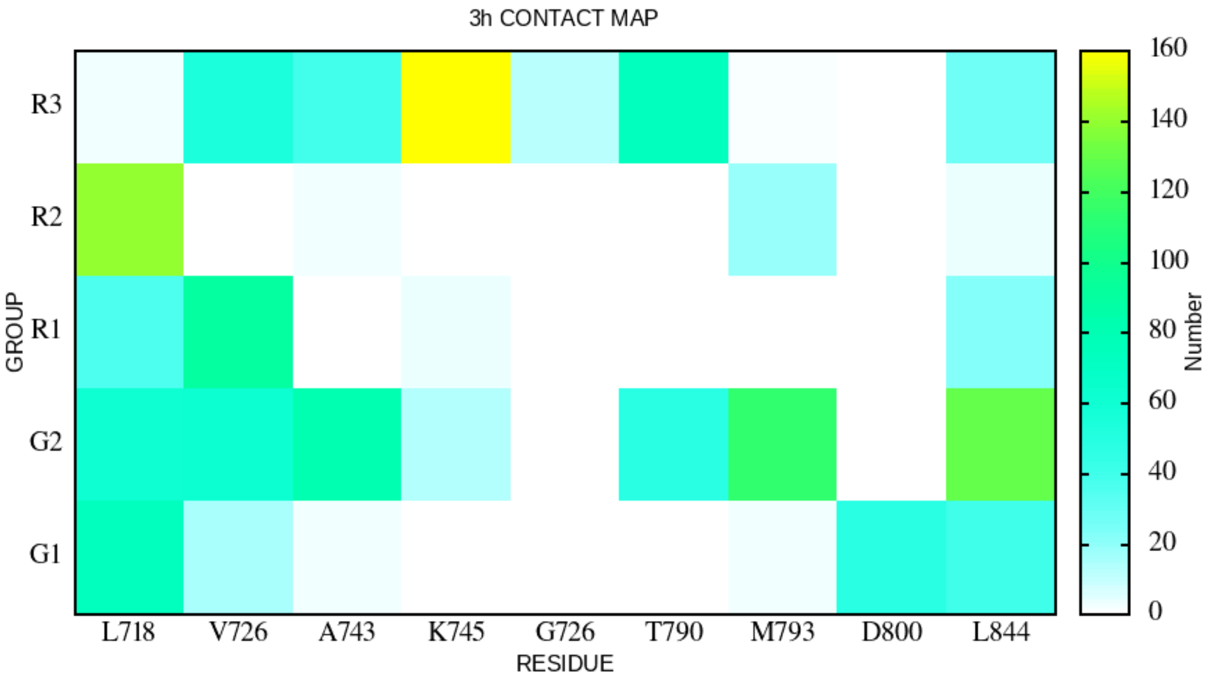
3h CONTACT MAP
R3
R2
R1
G2
G1
GROUP
L718
V726
A743
K745
G726
T790
M793
D800
L844
RESIDUE
160
140
120
100
80
60
40
20
0
Number

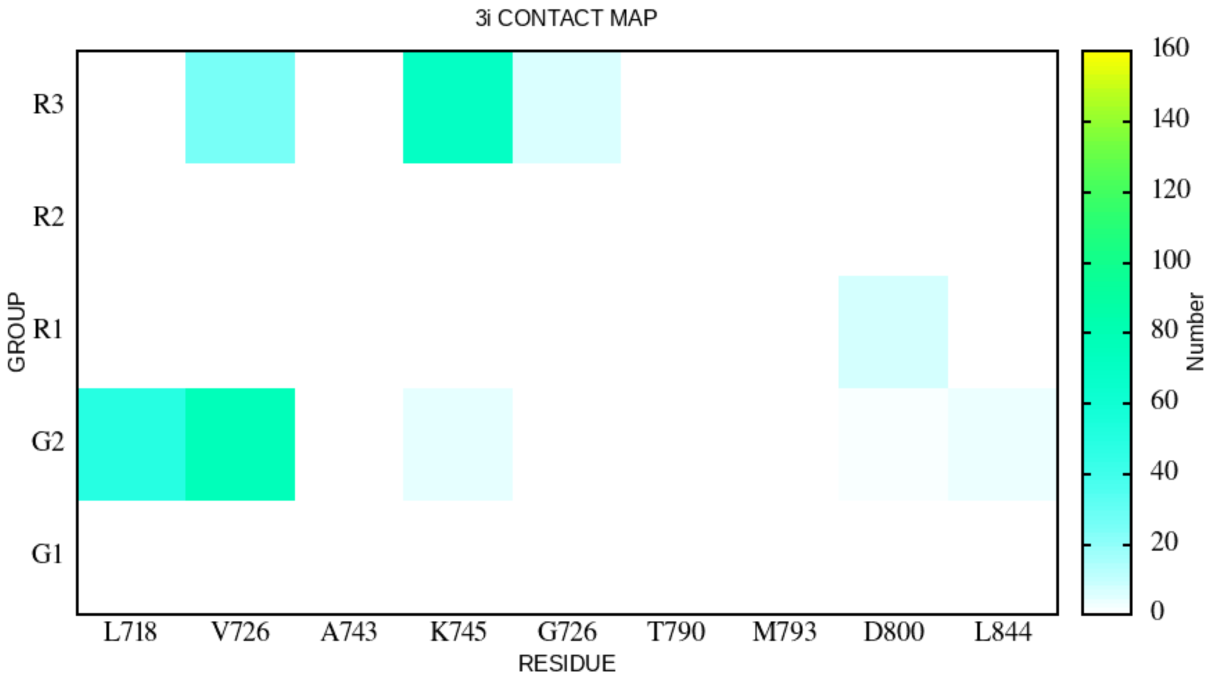
3i CONTACT MAP
R3
R2
R1
G2
G1
GROUP
L718
V726
A743
K745
G726
T790
M793
D800
L844
RESIDUE
160
140
120
100
80
60
40
20
0
Number

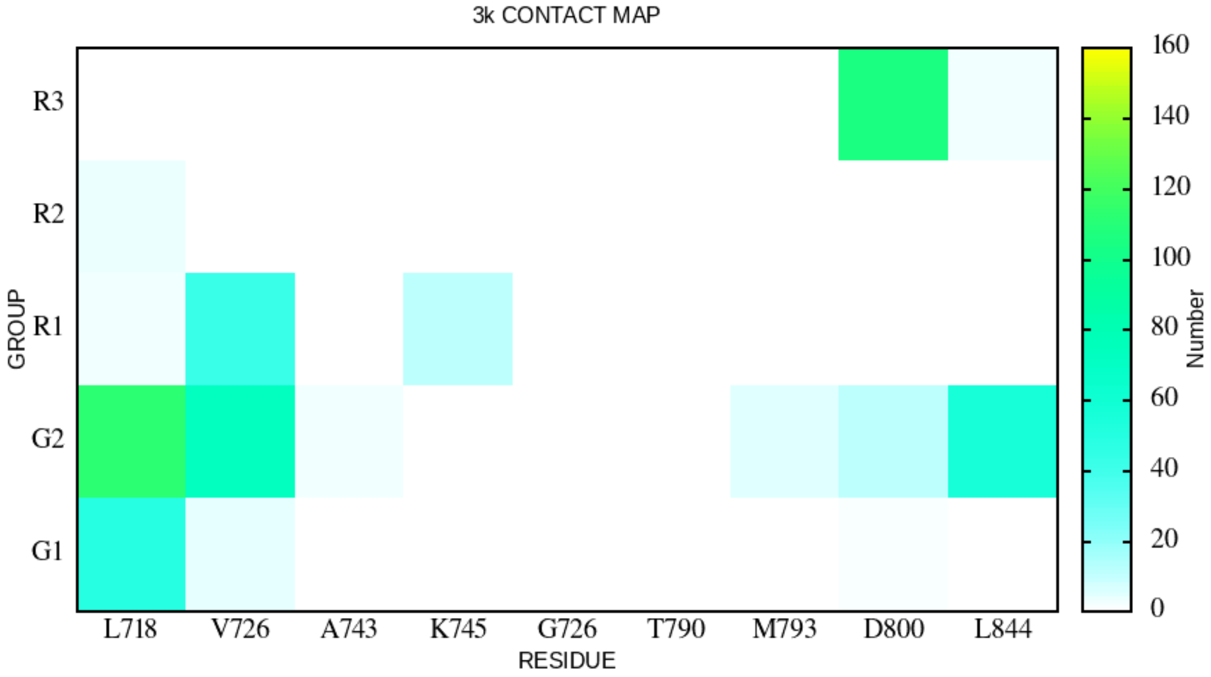
3k CONTACT MAP
R3
R2
R1
G2
G1
GROUP
L718
V726
A743
K745
G726
T790
M793
D800
L844
RESIDUE
160
140
120
100
80
60
40
20
0
Number

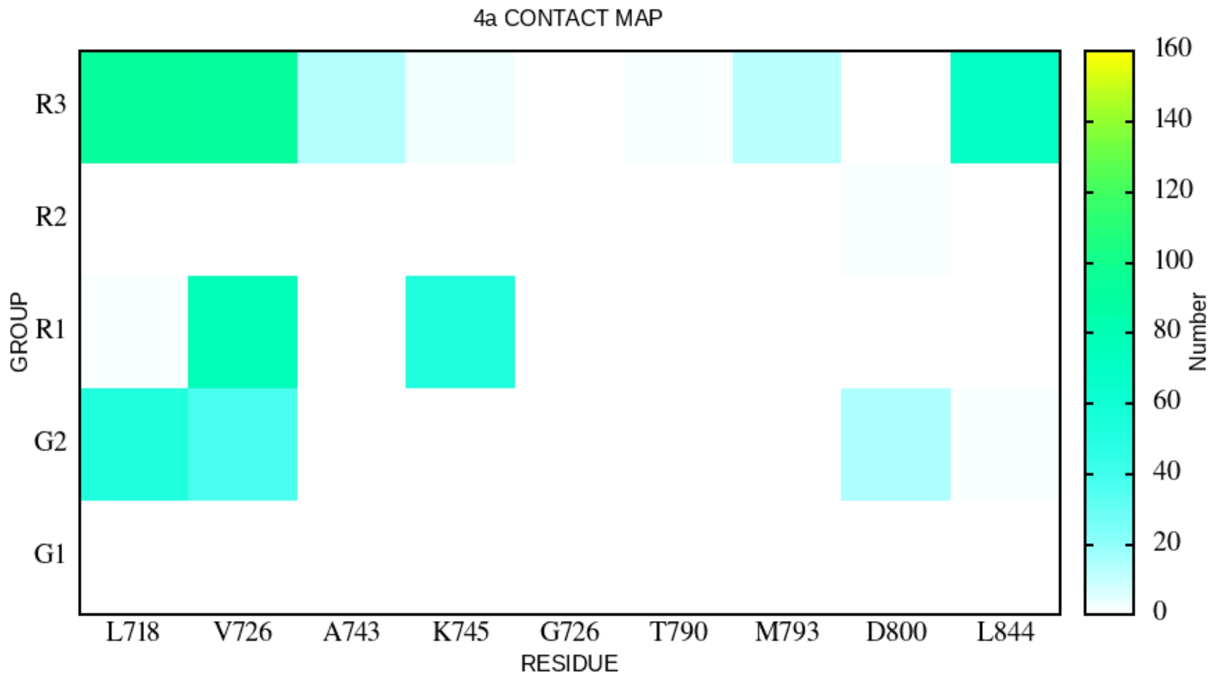
4a CONTACT MAP
R3
R2
R1
G2
G1
GROUP
L718
V726
A743
K745
G726
T790
M793
D800
L844
RESIDUE
160
140
120
100
80
60
40
20
0
Number

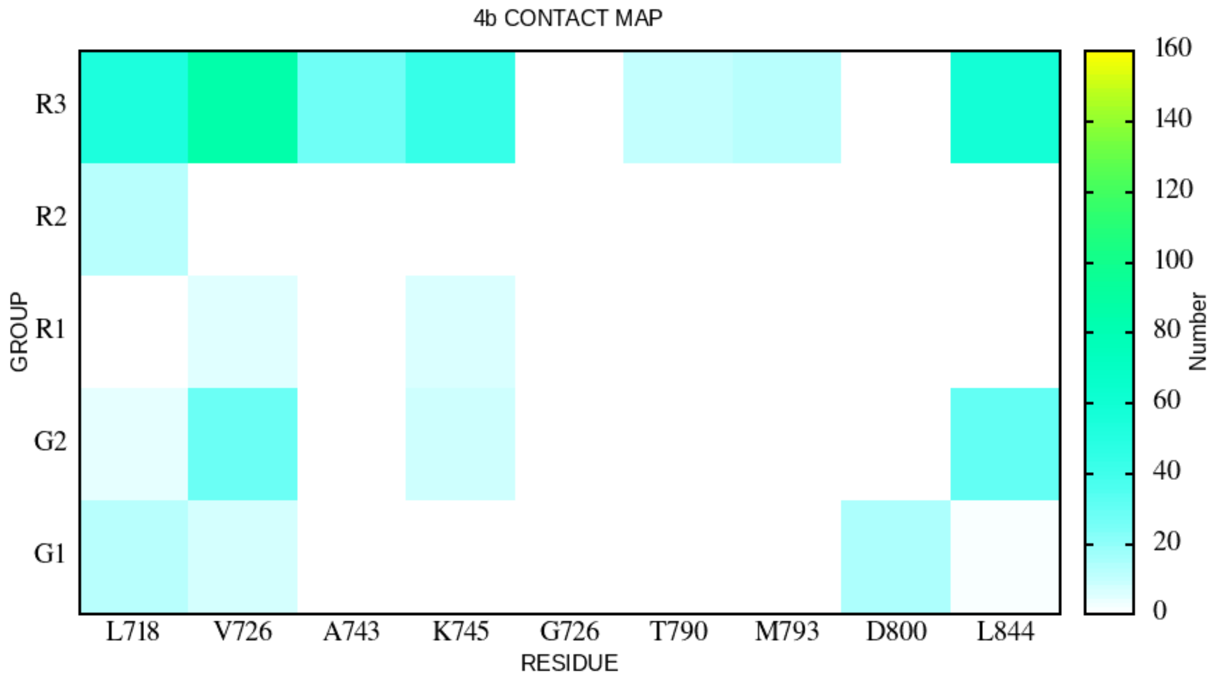
4b CONTACT MAP
R3
R2
R1
G2
G1
GROUP
L718
V726
A743
K745
G726
T790
M793
D800
L844
RESIDUE
160
140
120
100
80
60
40
20
0
Number

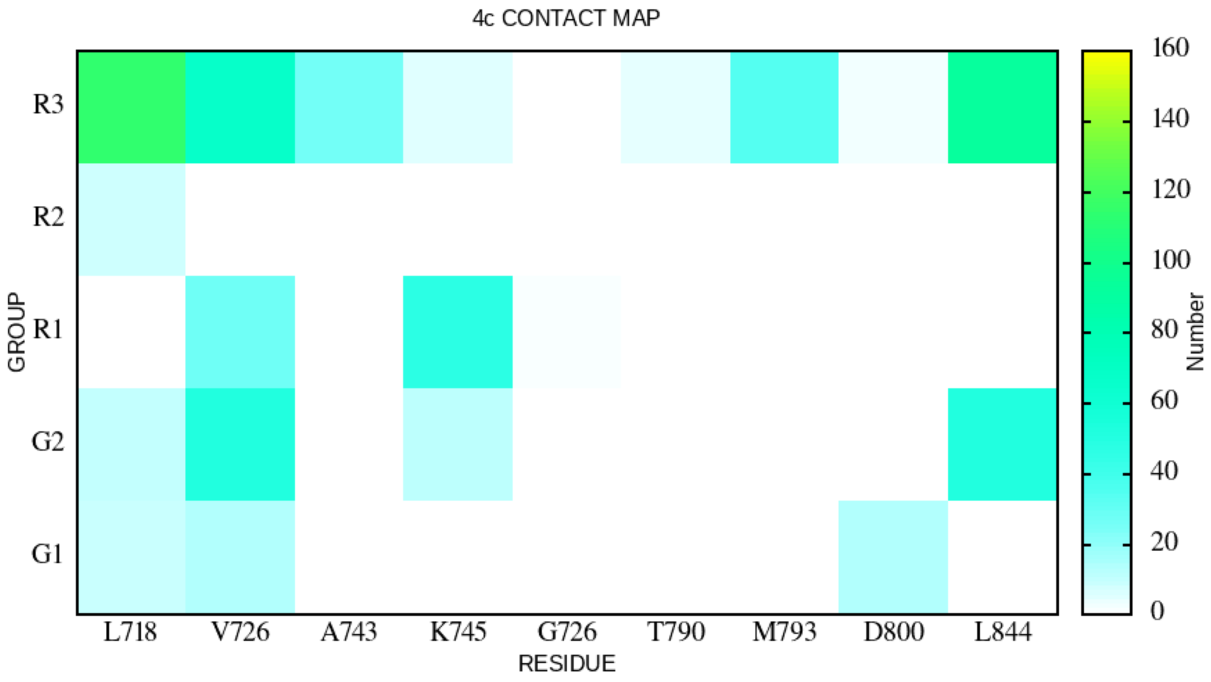
4c CONTACT MAP
R3
R2
R1
G2
G1
GROUP
L718
V726
A743
K745
G726
T790
M793
D800
L844
RESIDUE
160
140
120
100
80
60
40
20
0
Number

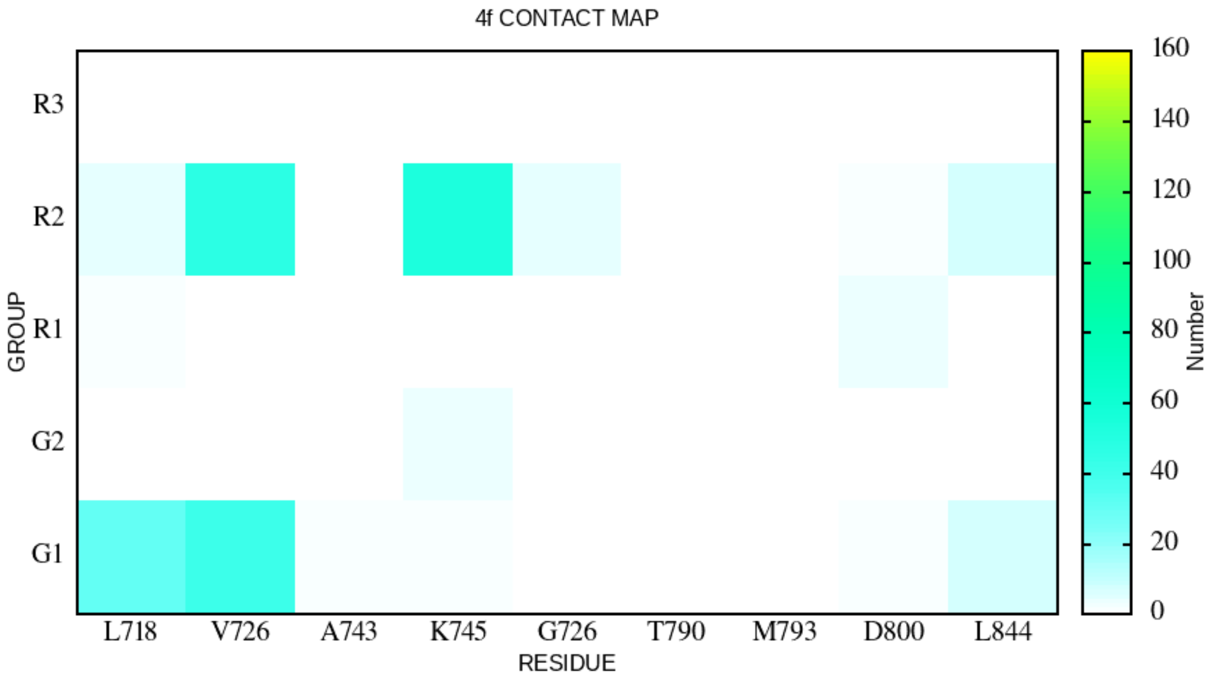
4f CONTACT MAP
R3
R2
R1
G2
G1
GROUP
L718
V726
A743
K745
G726
T790
M793
D800
L844
RESIDUE
160
140
120
100
80
60
40
20
0
Number

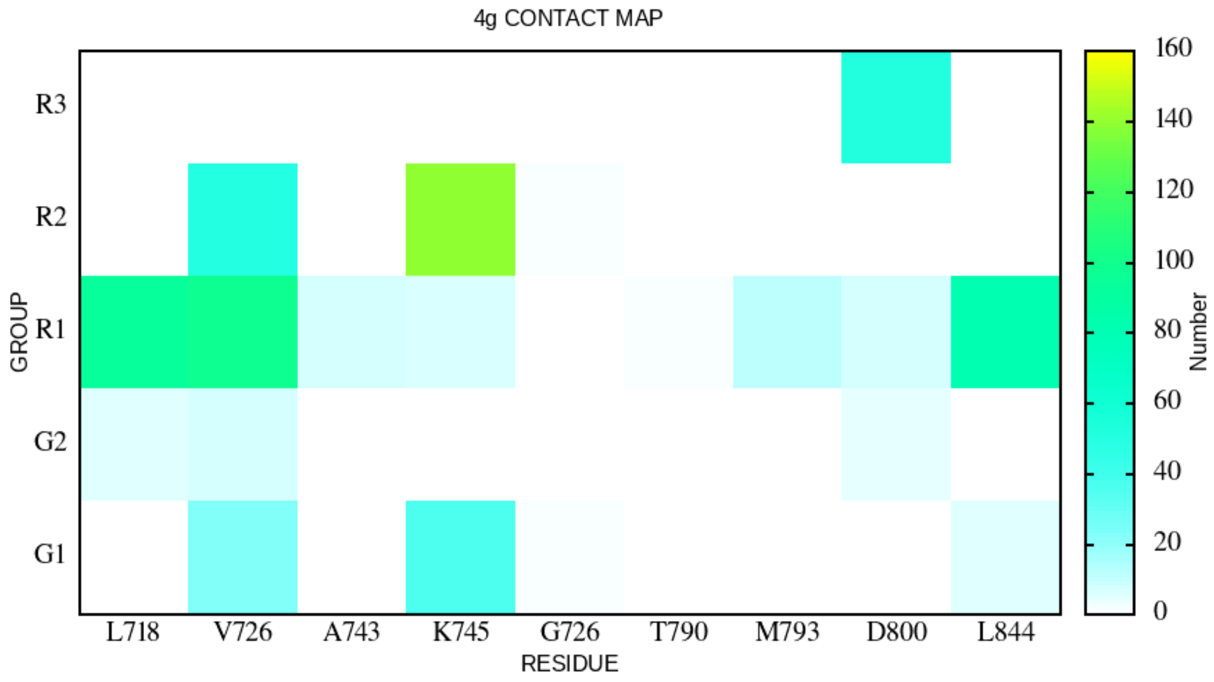
4g CONTACT MAP
R3
R2
R1
G2
G1
GROUP
L718
V726
A743
K745
G726
T790
M793
D800
L844
RESIDUE
160
140
120
100
80
60
40
20
0
Number

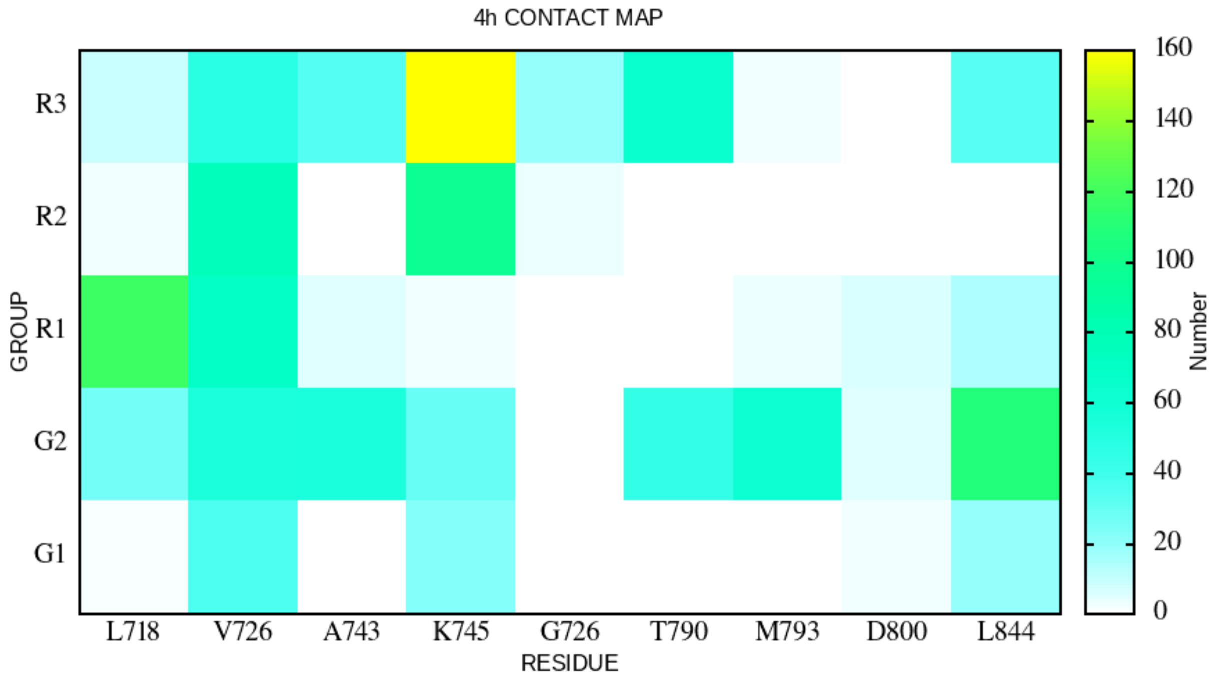
4h CONTACT MAP
R3
R2
R1
G2
G1
GROUP
L718
V726
A743
K745
G726
T790
M793
D800
L844
RESIDUE
160
140
120
100
80
60
40
20
0
Number

Figure 58: *The Coulomb interaction map between 5 sub – groups of* ***compound*** *and 9 key residues of EGFR tyrosine domain.*

DATA are shown in case of ***15 compounds.***

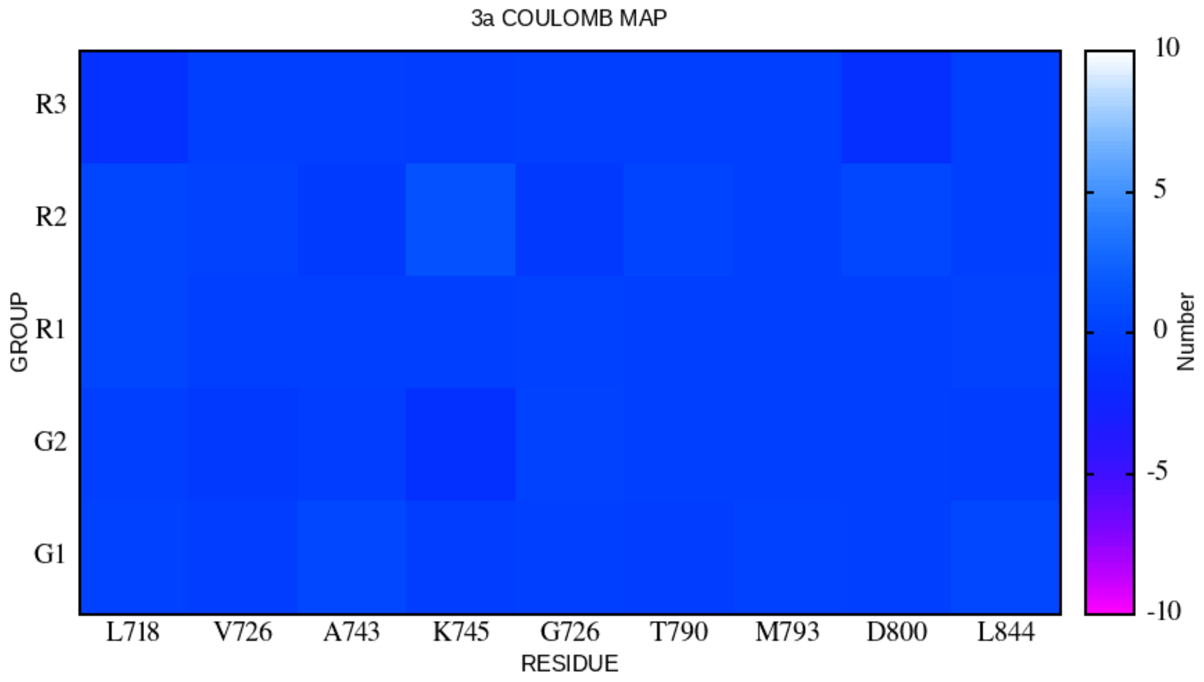

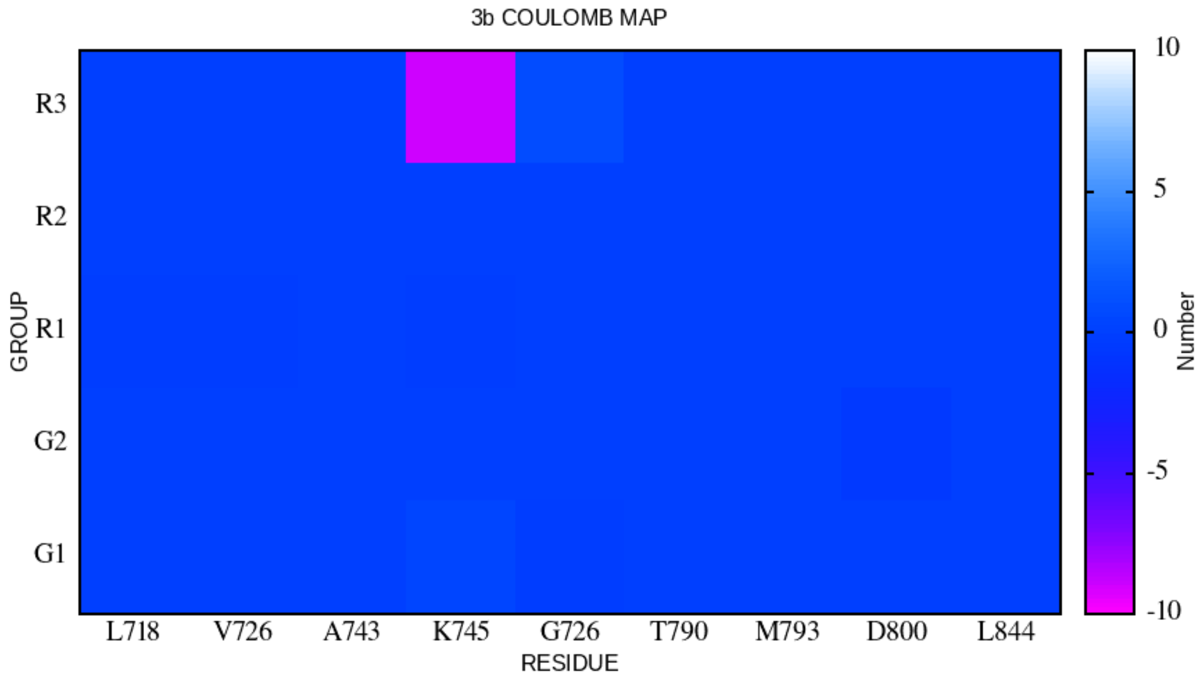

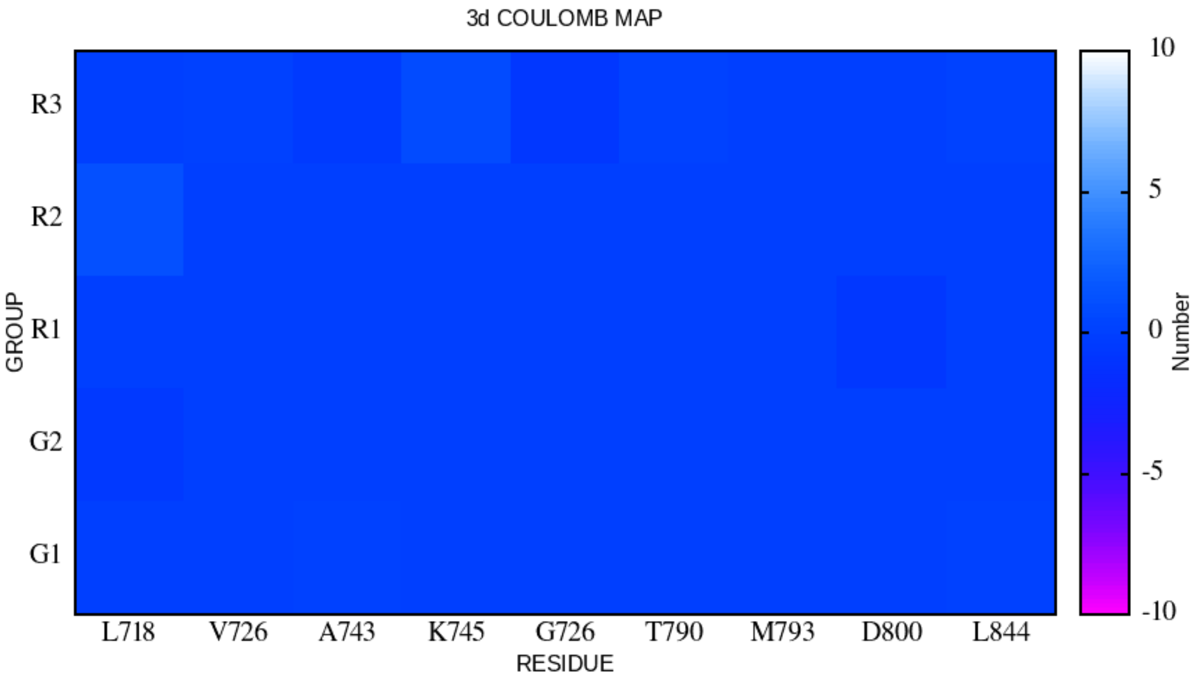

3d COULOMB MAP
R3
R2
R1
G2
G1
GROUP
L718
V726
A743
K745
G726
T790
M793
D800
L844
RESIDUE
10
5
0
-5
-10
Number

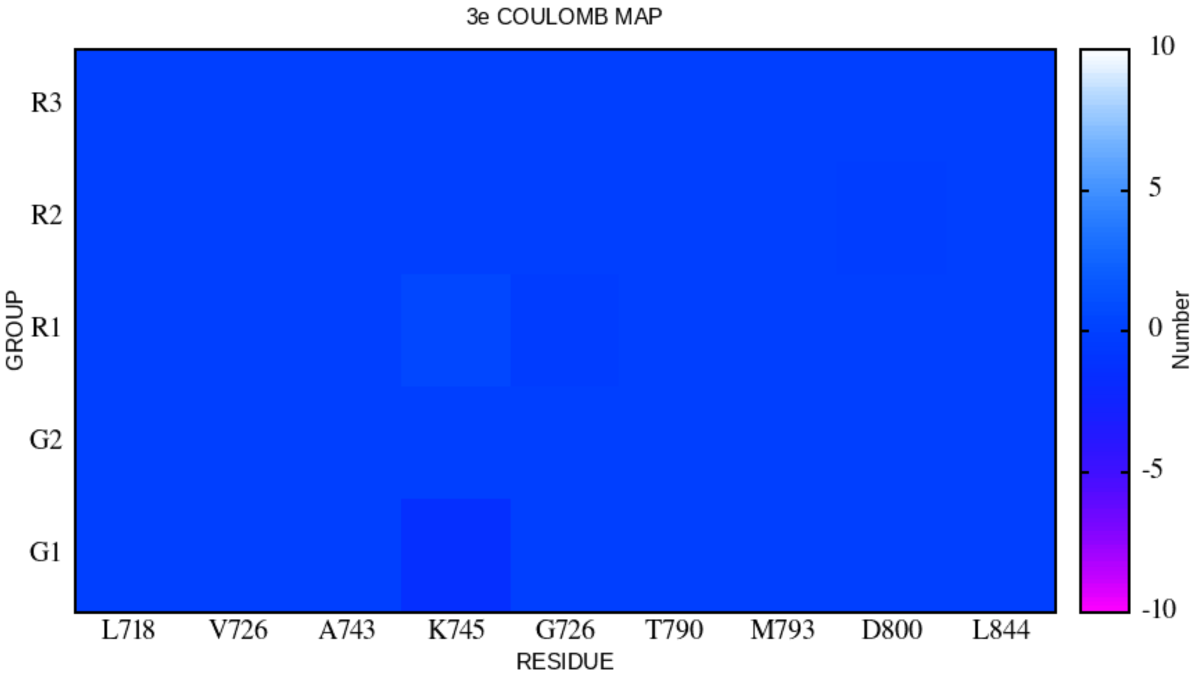

3e COULOMB MAP
R3
R2
R1
G2
G1
GROUP
L718
V726
A743
K745
G726
T790
M793
D800
L844
RESIDUE
10
5
0
-5
-10
Number

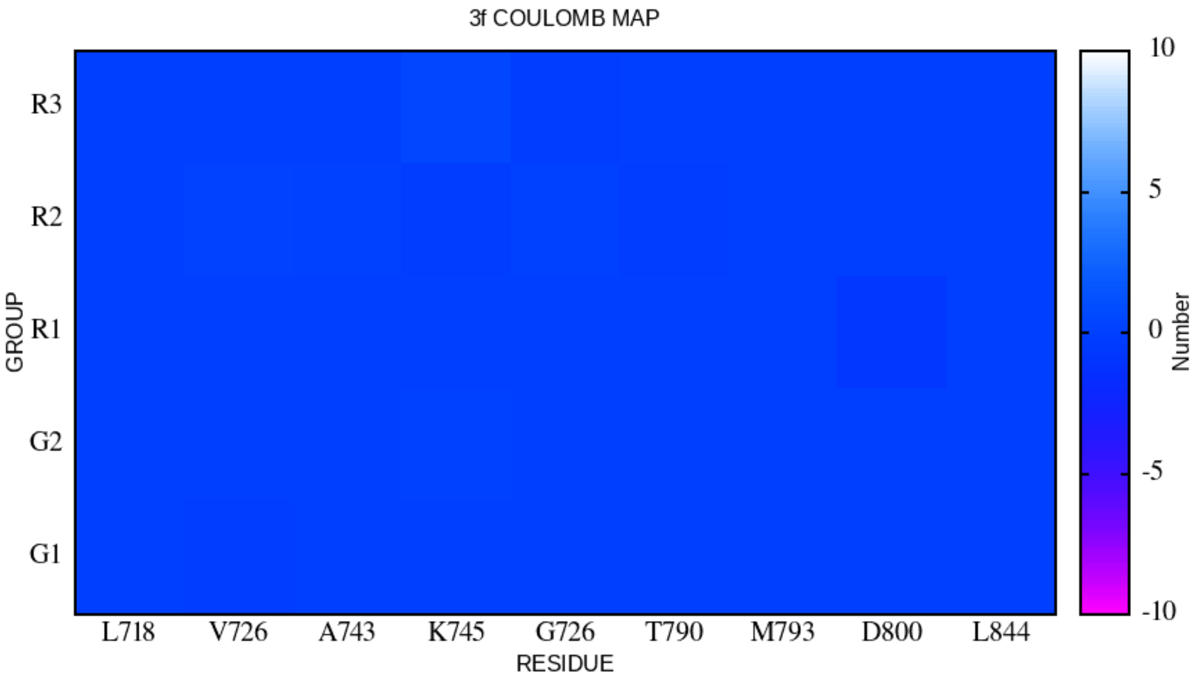
3f COULOMB MAP
R3
R2
R1
G2
G1
GROUP
L718
V726
A743
K745
G726
T790
M793
D800
L844
RESIDUE
10
5
0
-5
-10
Number

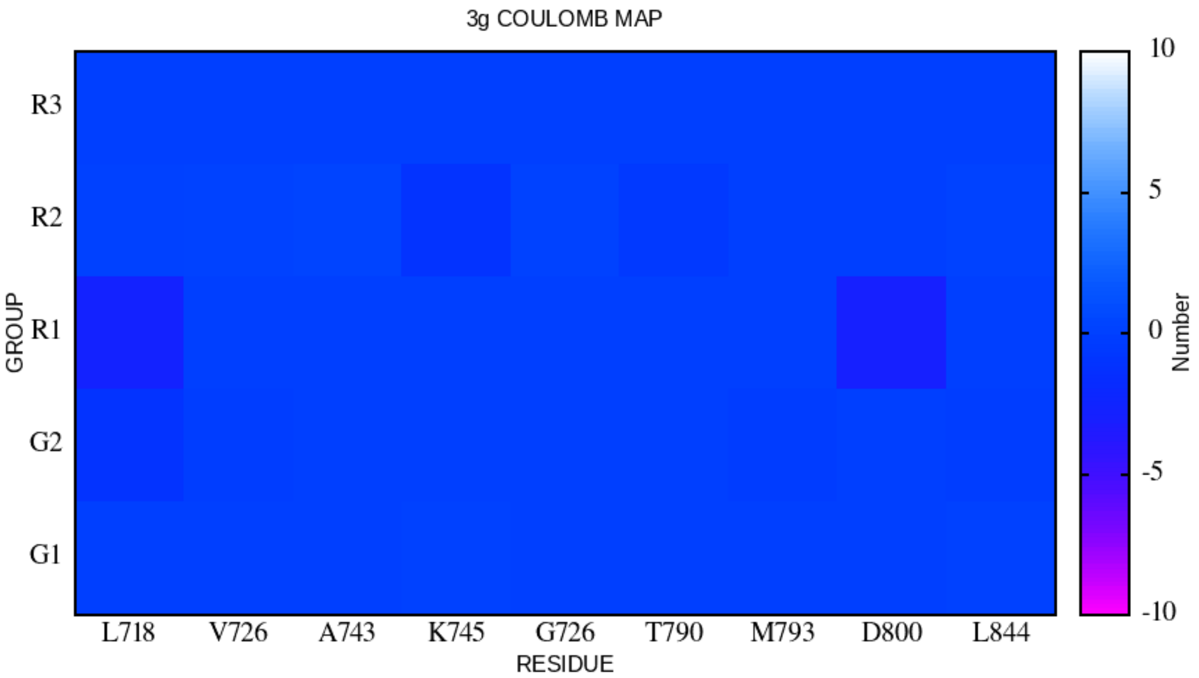
3g COULOMB MAP
R3
R2
R1
G2
G1
GROUP
L718
V726
A743
K745
G726
T790
M793
D800
L844
RESIDUE
10
5
0
-5
-10
Number

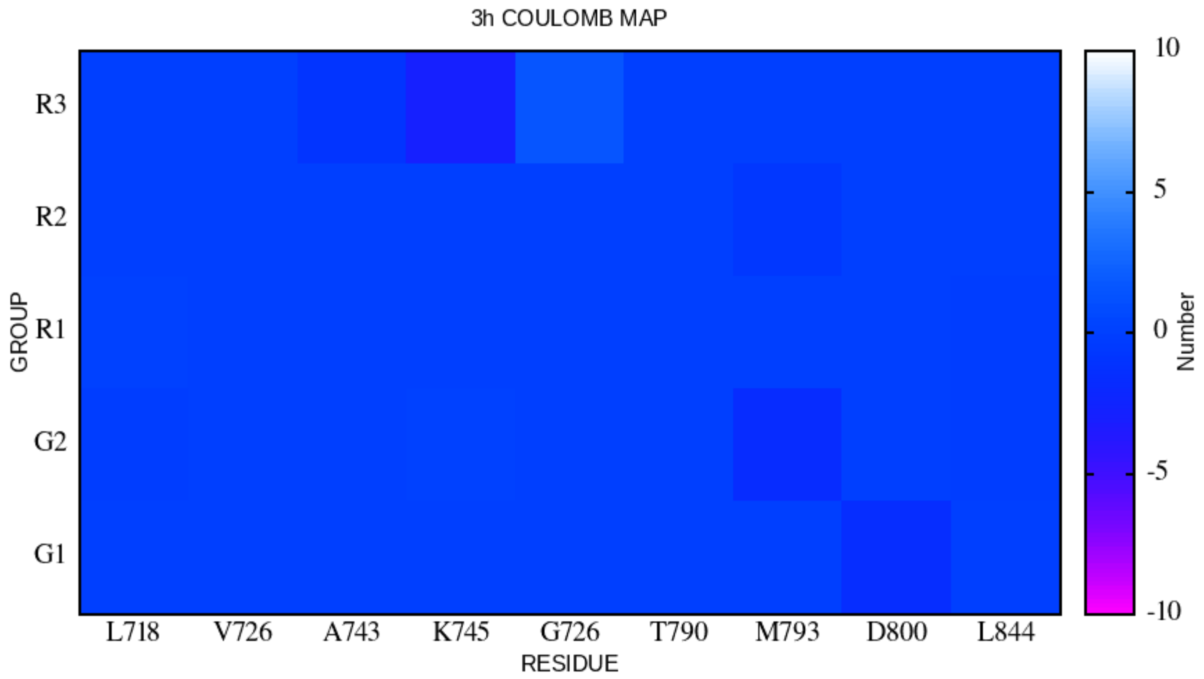
3h COULOMB MAP
R3
R2
GROUP
R1
G2
G1
L718
V726
A743
K745
G726
T790
M793
D800
L844
RESIDUE
10
5
0
-5
-10
Number

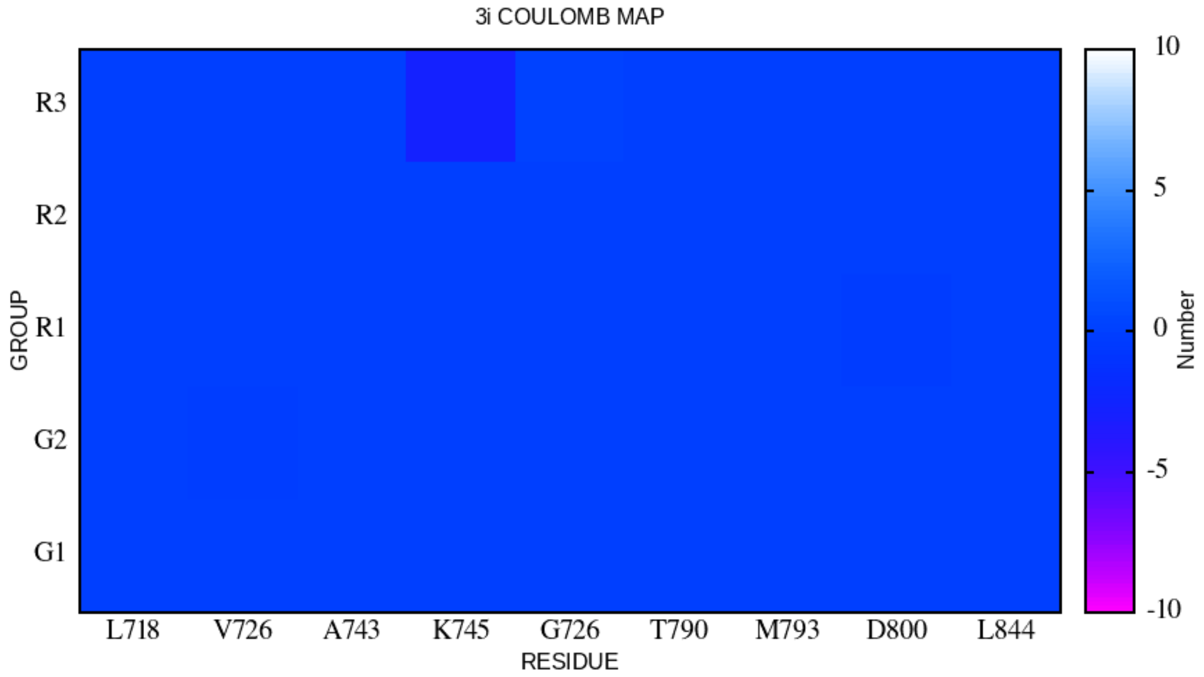
3i COULOMB MAP
R3
R2
GROUP
R1
G2
G1
L718
V726
A743
K745
G726
T790
M793
D800
L844
RESIDUE
10
5
0
-5
-10
Number

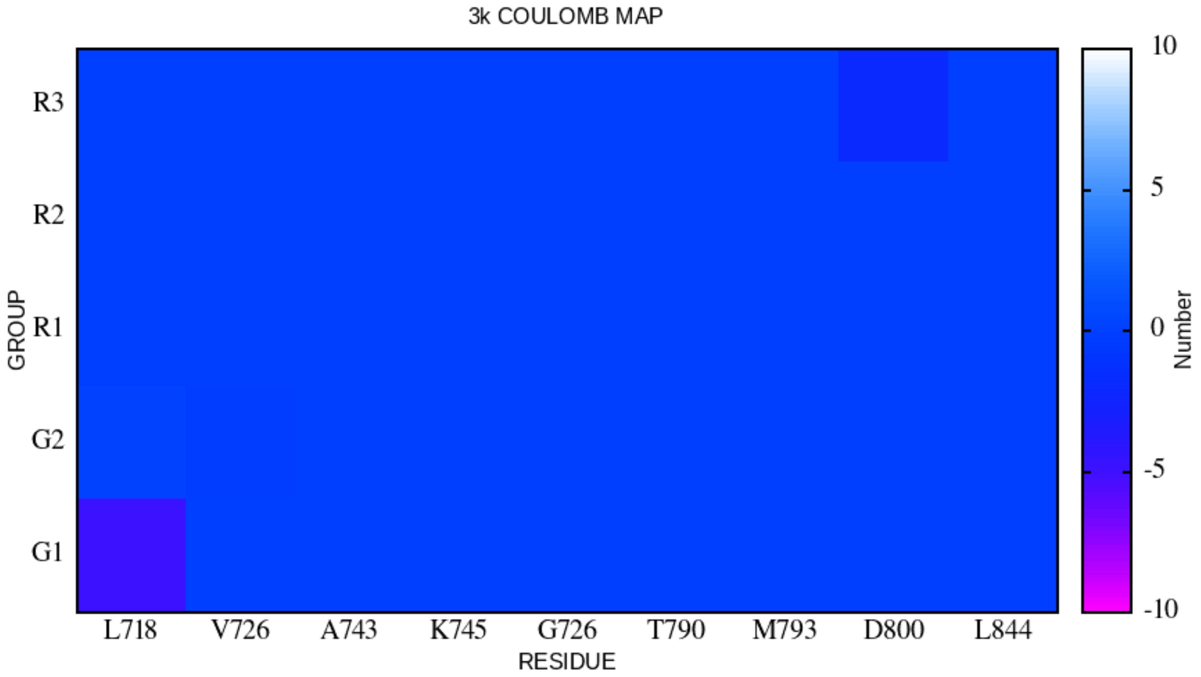
3k COULOMB MAP
R3
R2
R1
G2
G1
GROUP
L718
V726
A743
K745
G726
T790
M793
D800
L844
RESIDUE
10
5
0
-5
-10
Number

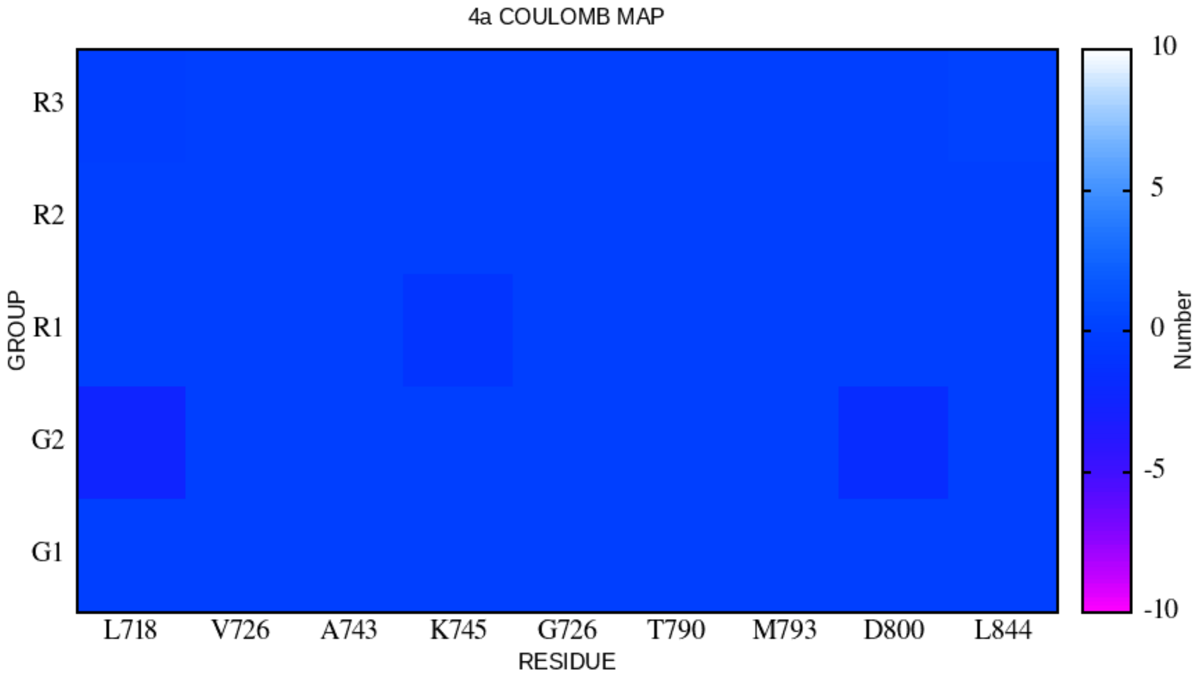
4a COULOMB MAP
R3
R2
R1
G2
G1
GROUP
L718
V726
A743
K745
G726
T790
M793
D800
L844
RESIDUE
10
5
0
-5
-10
Number

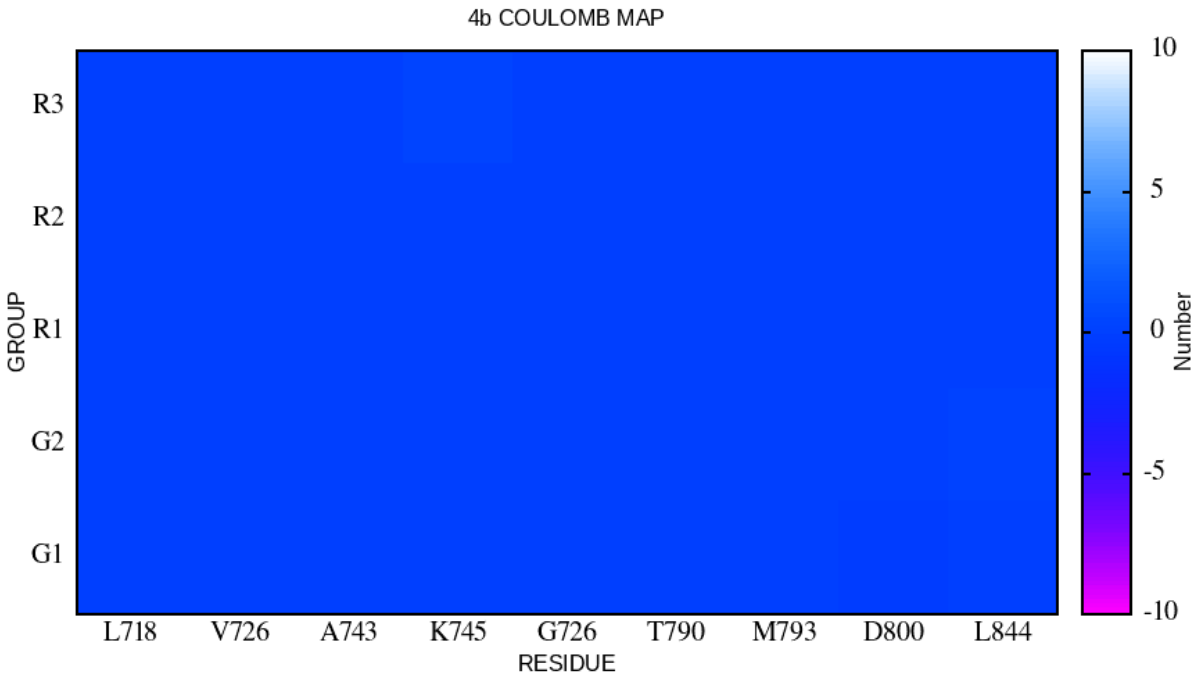
4b COULOMB MAP
R3
R2
R1
G2
G1
GROUP
L718
V726
A743
K745
G726
T790
M793
D800
L844
RESIDUE
10
5
0
-5
-10
Number

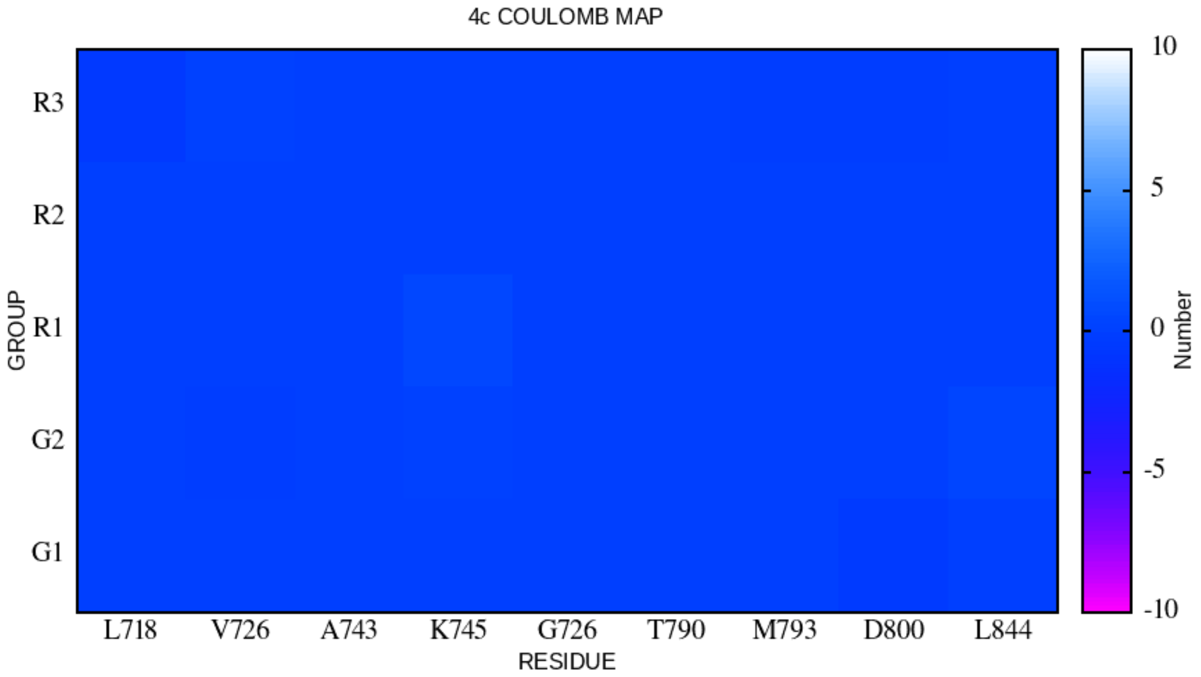
4c COULOMB MAP
R3
R2
R1
G2
G1
GROUP
L718
V726
A743
K745
G726
T790
M793
D800
L844
RESIDUE
10
5
0
-5
-10
Number

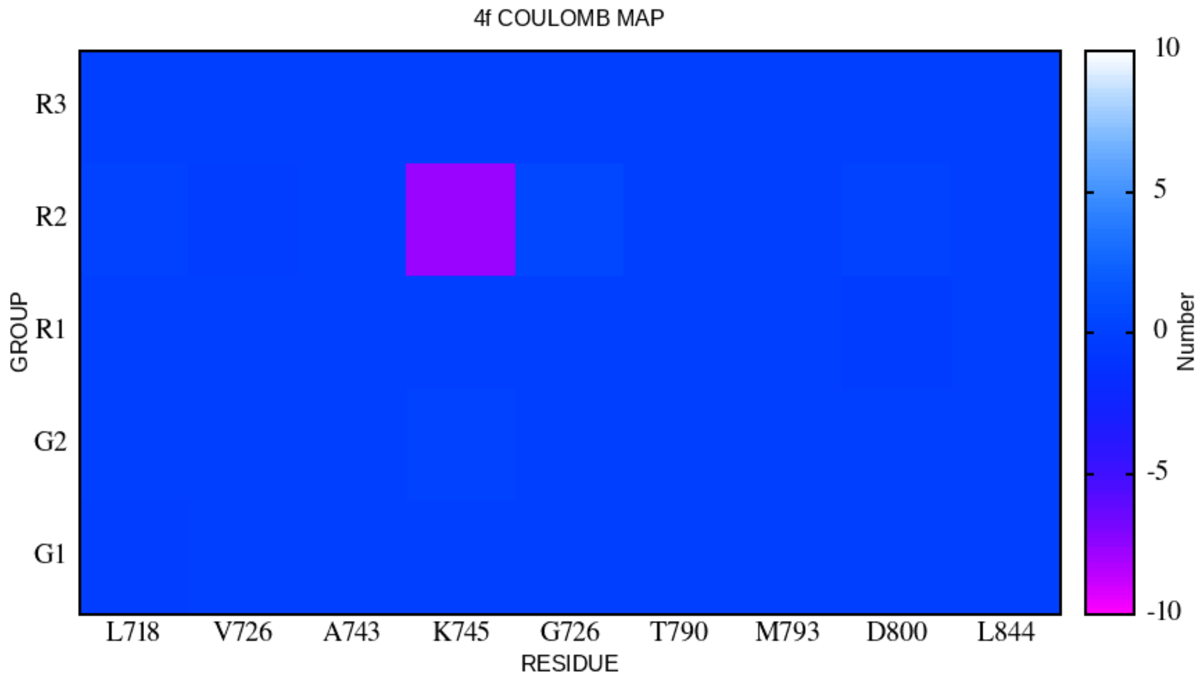
4f COULOMB MAP
R3
R2
R1
G2
G1
GROUP
L718
V726
A743
K745
G726
T790
M793
D800
L844
RESIDUE
10
5
0
-5
-10
Number

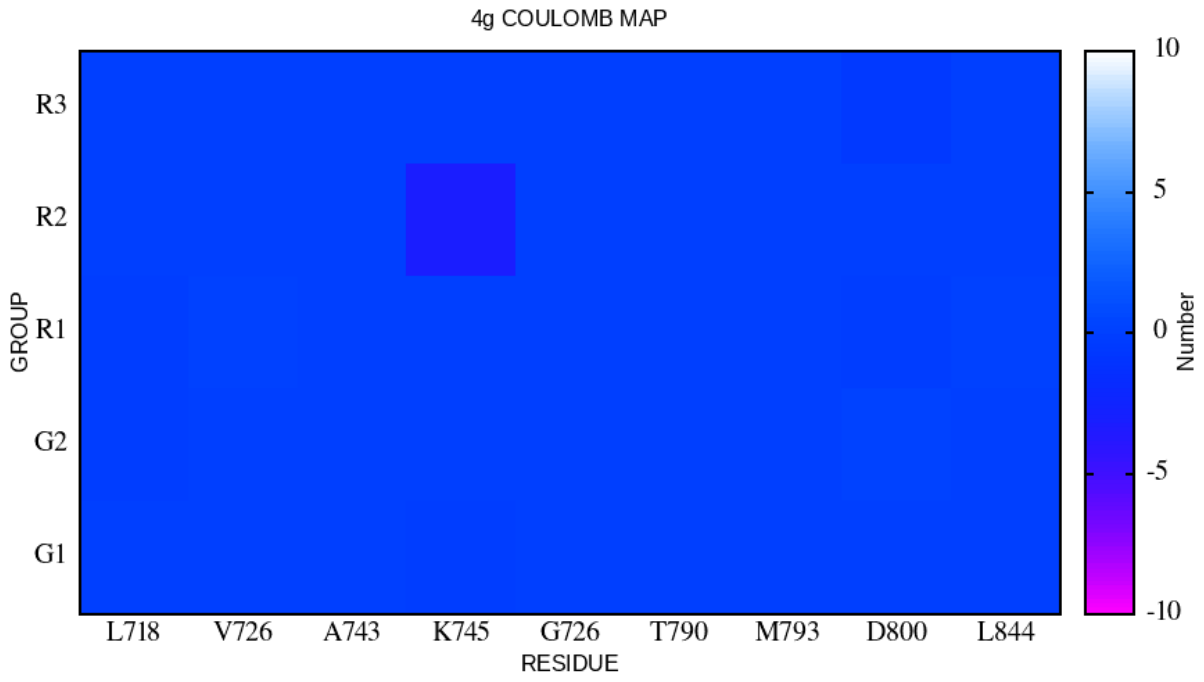
4g COULOMB MAP
R3
R2
R1
G2
G1
GROUP
L718
V726
A743
K745
G726
T790
M793
D800
L844
RESIDUE
10
5
0
-5
-10
Number

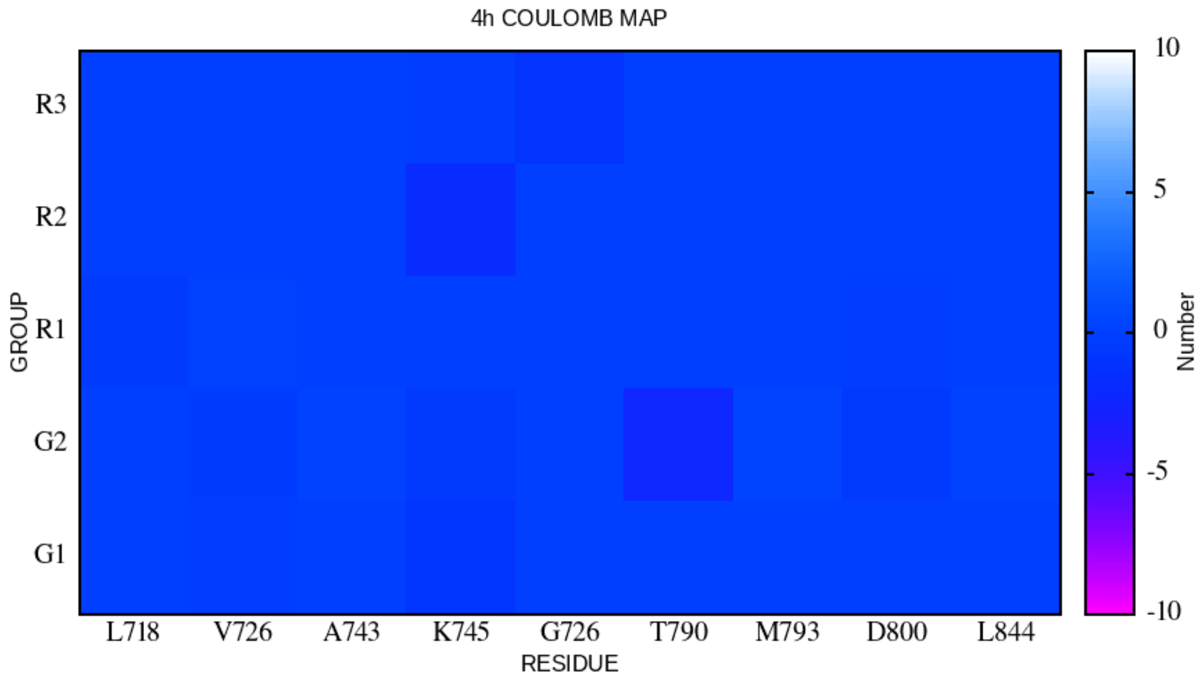

Figure 59: *The Vdw interaction map between 5 sub – groups of* ***compound*** *and 9 key residues of EGFR tyrosine domain.*

DATA are shown in case of ***15 compounds.***

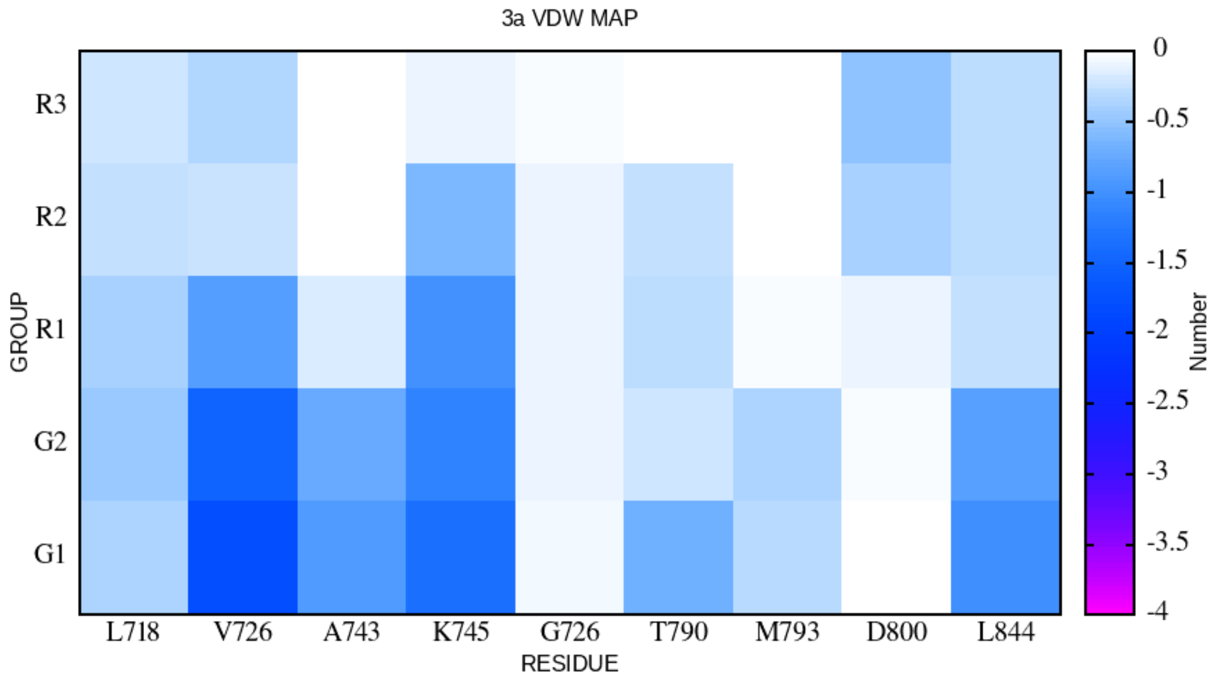
3a VDW MAP
R3
R2
R1
G2
G1
GROUP
L718
V726
A743
K745
G726
T790
M793
D800
L844
RESIDUE
0
-0.5
-1
-1.5
-2
-2.5
-3
-3.5
-4
Number

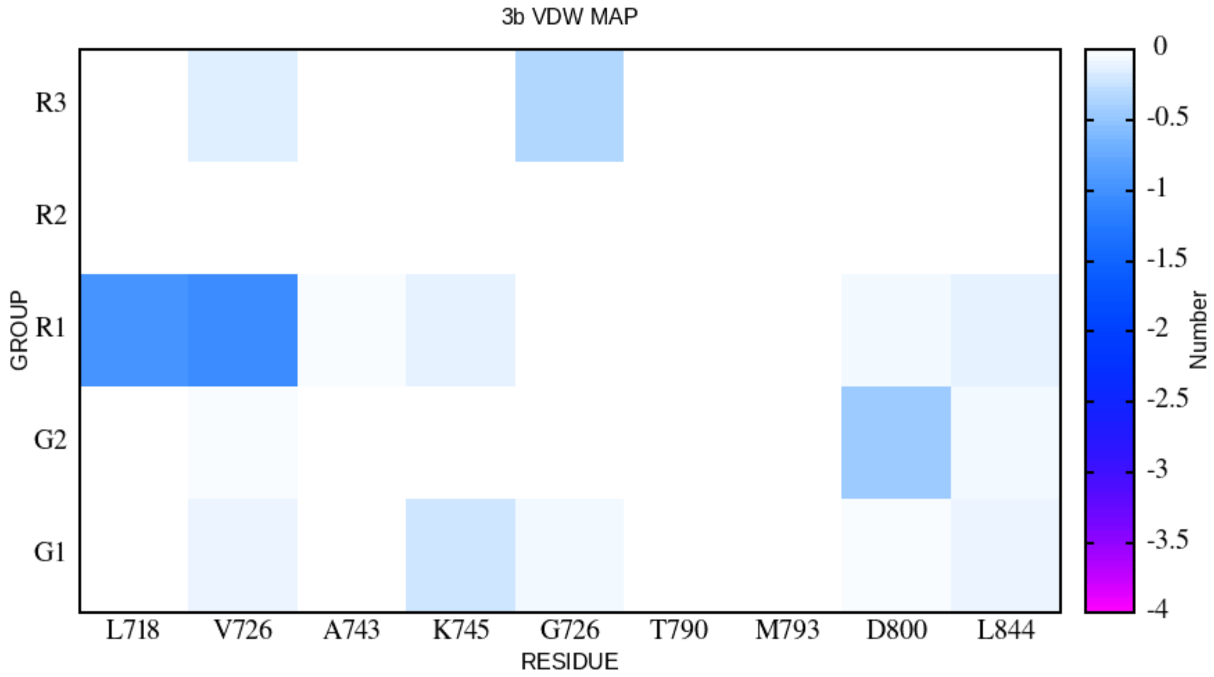
3b VDW MAP
R3
R2
R1
G2
G1
GROUP
L718
V726
A743
K745
G726
T790
M793
D800
L844
RESIDUE
0
-0.5
-1
-1.5
-2
-2.5
-3
-3.5
-4
Number

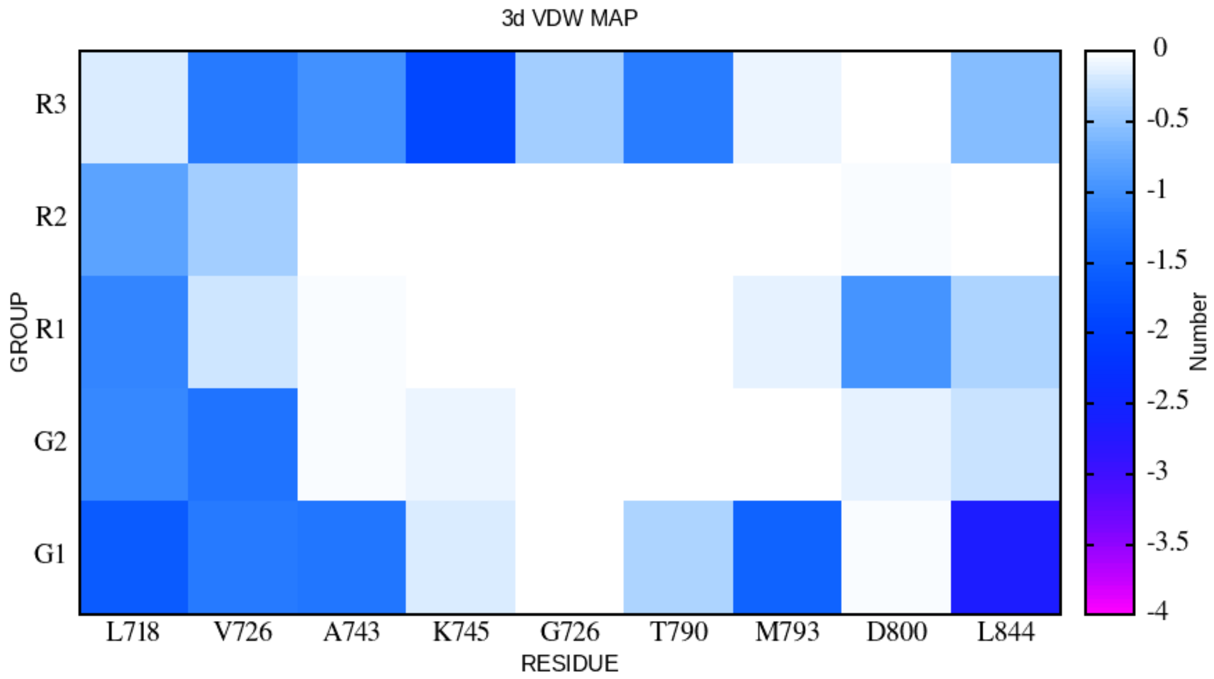
3d VDW MAP
R3
R2
R1
G2
G1
GROUP
L718
V726
A743
K745
G726
T790
M793
D800
L844
RESIDUE
0
-0.5
-1
-1.5
-2
-2.5
-3
-3.5
-4
Number

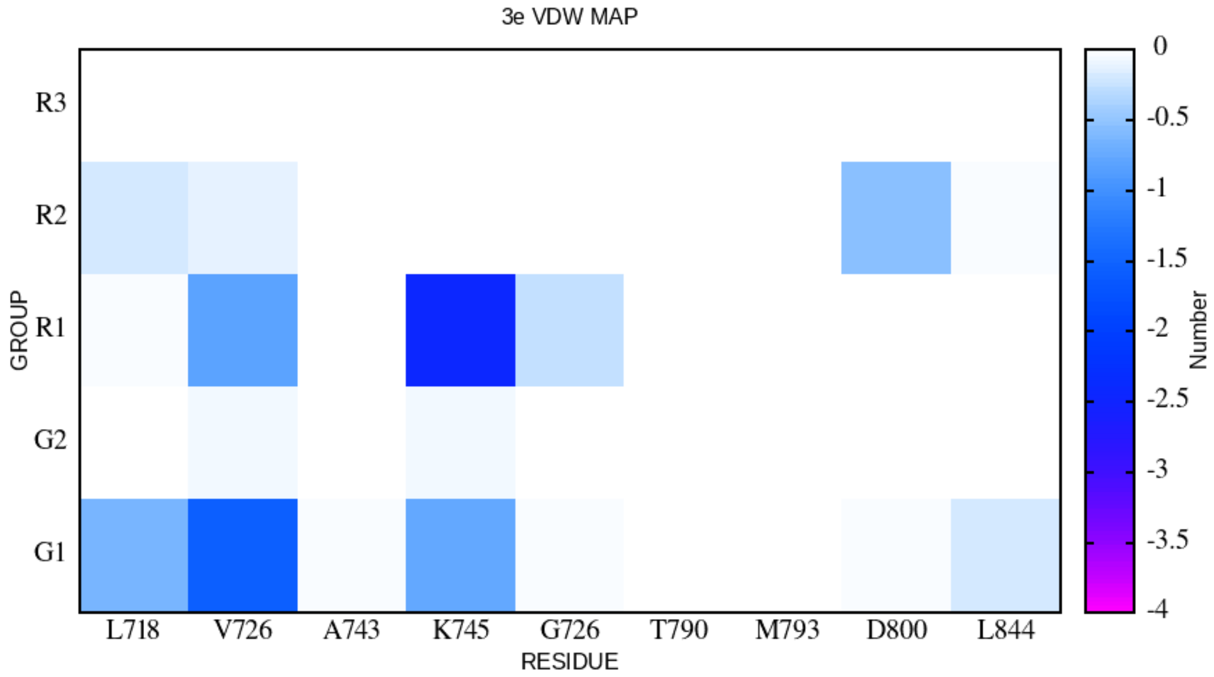
3e VDW MAP
R3
R2
R1
G2
G1
GROUP
L718
V726
A743
K745
G726
T790
M793
D800
L844
RESIDUE
0
-0.5
-1
-1.5
-2
-2.5
-3
-3.5
-4
Number

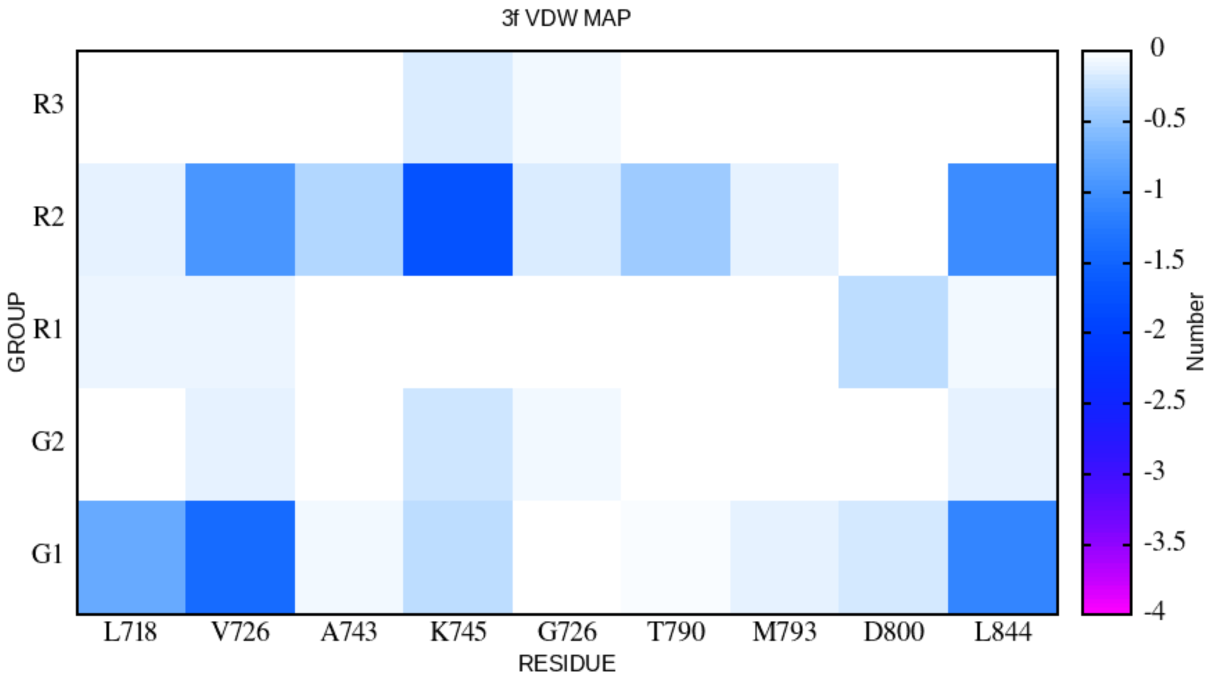
3f VDW MAP
R3
R2
R1
G2
G1
GROUP
L718
V726
A743
K745
G726
T790
M793
D800
L844
RESIDUE
0
-0.5
-1
-1.5
-2
-2.5
-3
-3.5
-4
Number

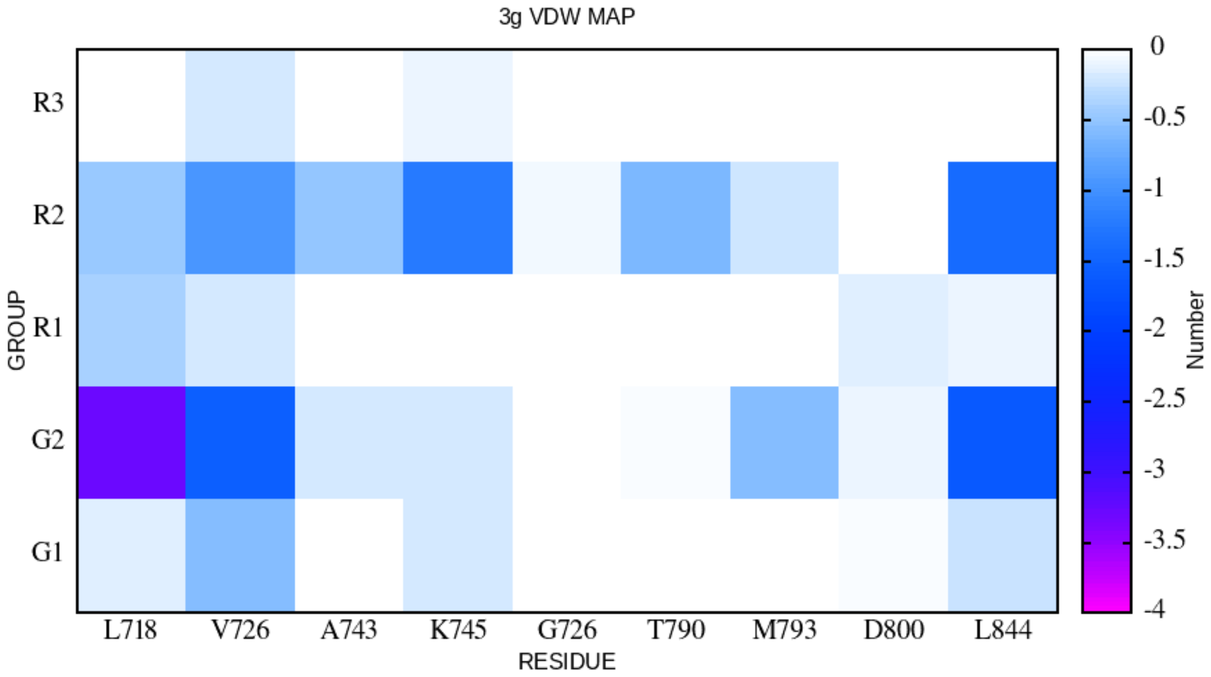
3g VDW MAP
R3
R2
R1
G2
G1
GROUP
L718
V726
A743
K745
G726
T790
M793
D800
L844
RESIDUE
0
-0.5
-1
-1.5
-2
-2.5
-3
-3.5
-4
Number

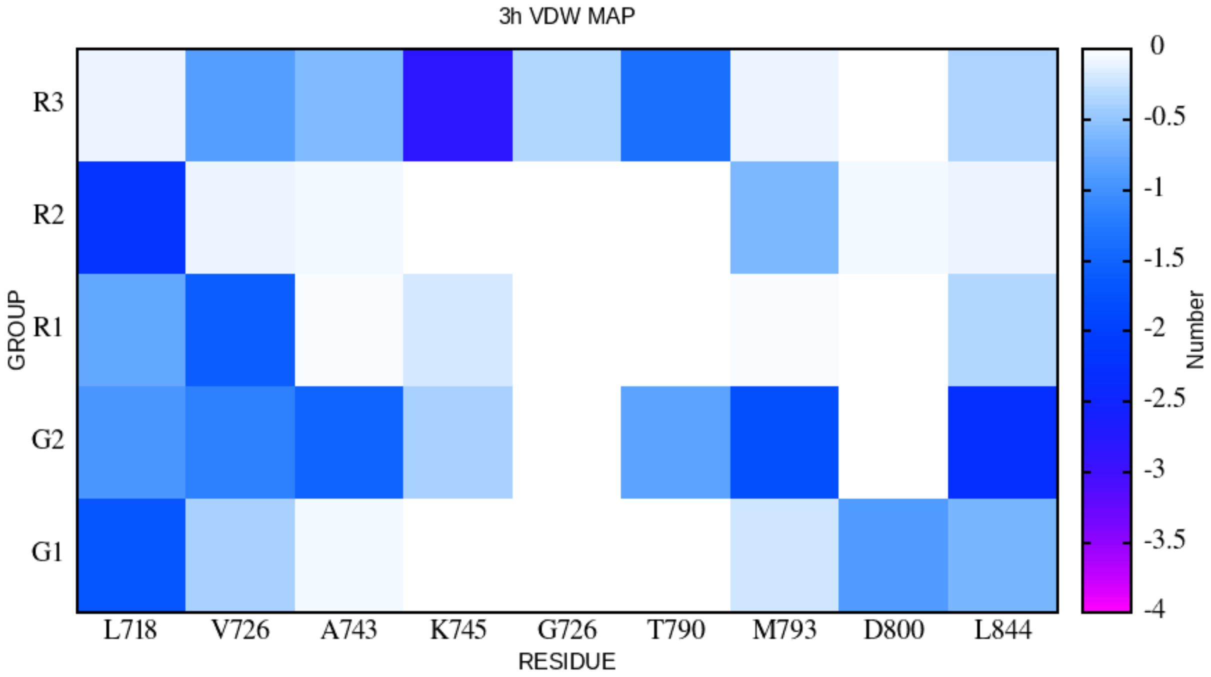
3h VDW MAP
R3
R2
R1
G2
G1
GROUP
L718
V726
A743
K745
G726
T790
M793
D800
L844
RESIDUE
0
-0.5
-1
-1.5
-2
-2.5
-3
-3.5
-4
Number

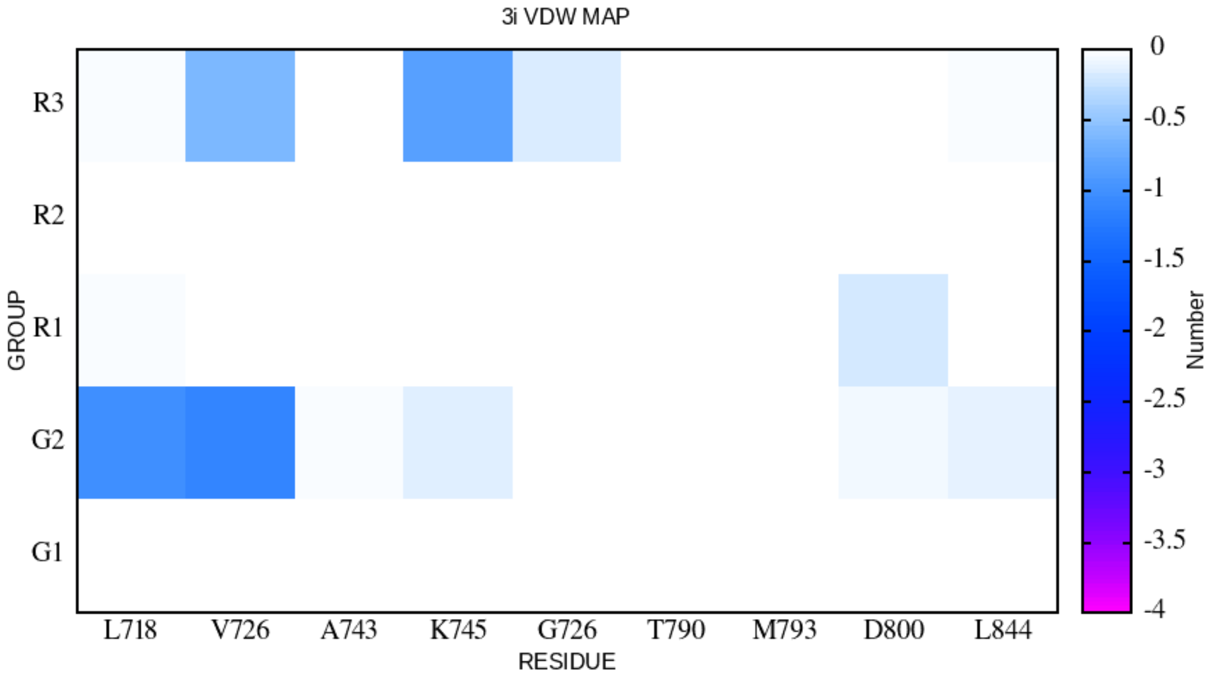
3i VDW MAP
R3
R2
R1
G2
G1
GROUP
L718
V726
A743
K745
G726
T790
M793
D800
L844
RESIDUE
0
-0.5
-1
-1.5
-2
-2.5
-3
-3.5
-4
Number

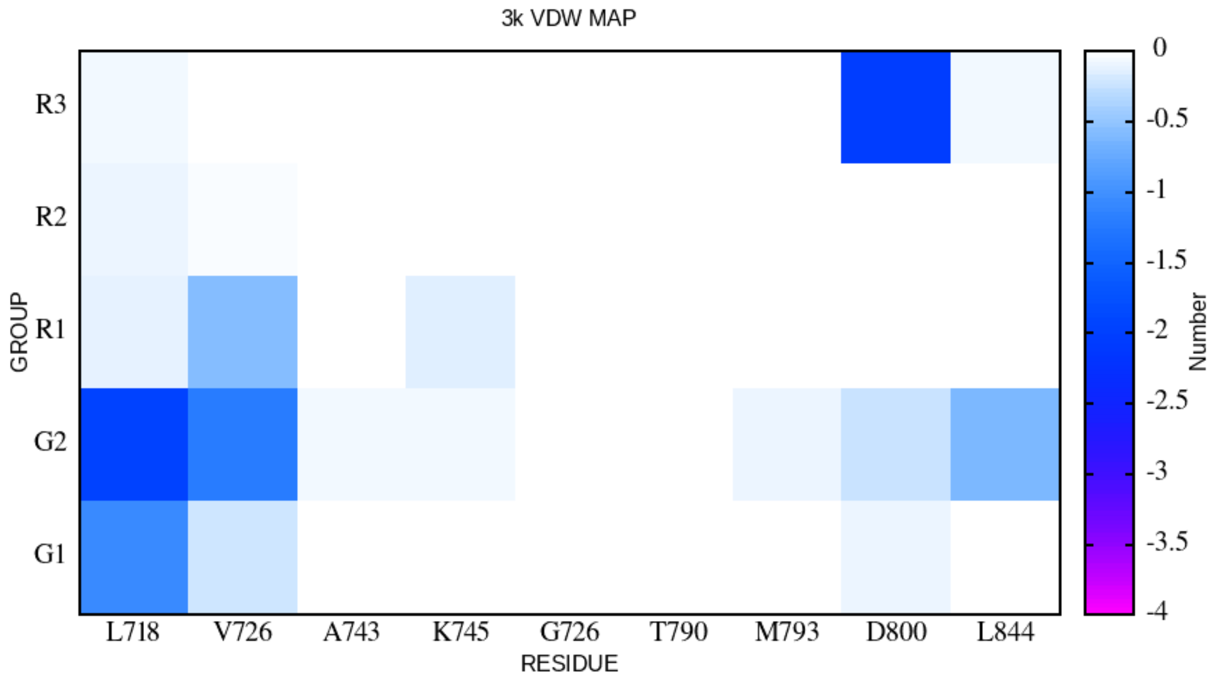
3k VDW MAP
GROUP
R3
R2
R1
G2
G1
L718
V726
A743
K745
G726
T790
M793
D800
L844
RESIDUE
Number
0
-0.5
-1
-1.5
-2
-2.5
-3
-3.5
-4

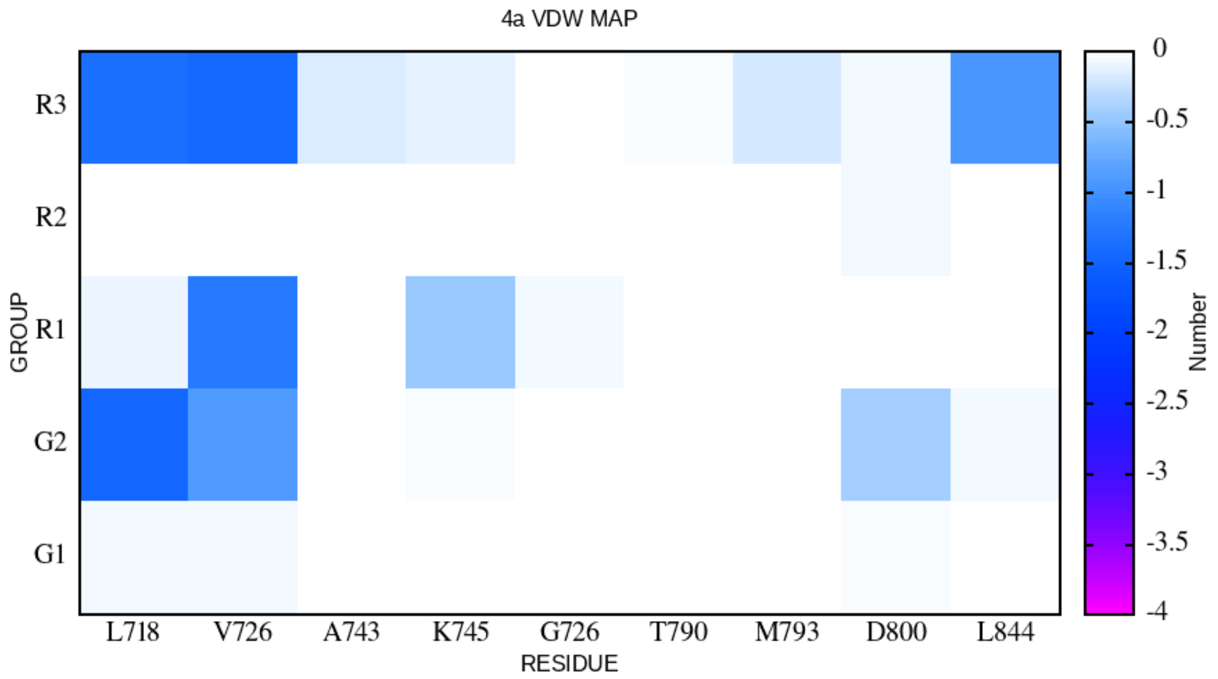
4a VDW MAP
GROUP
R3
R2
R1
G2
G1
L718
V726
A743
K745
G726
T790
M793
D800
L844
RESIDUE
Number
0
-0.5
-1
-1.5
-2
-2.5
-3
-3.5
-4

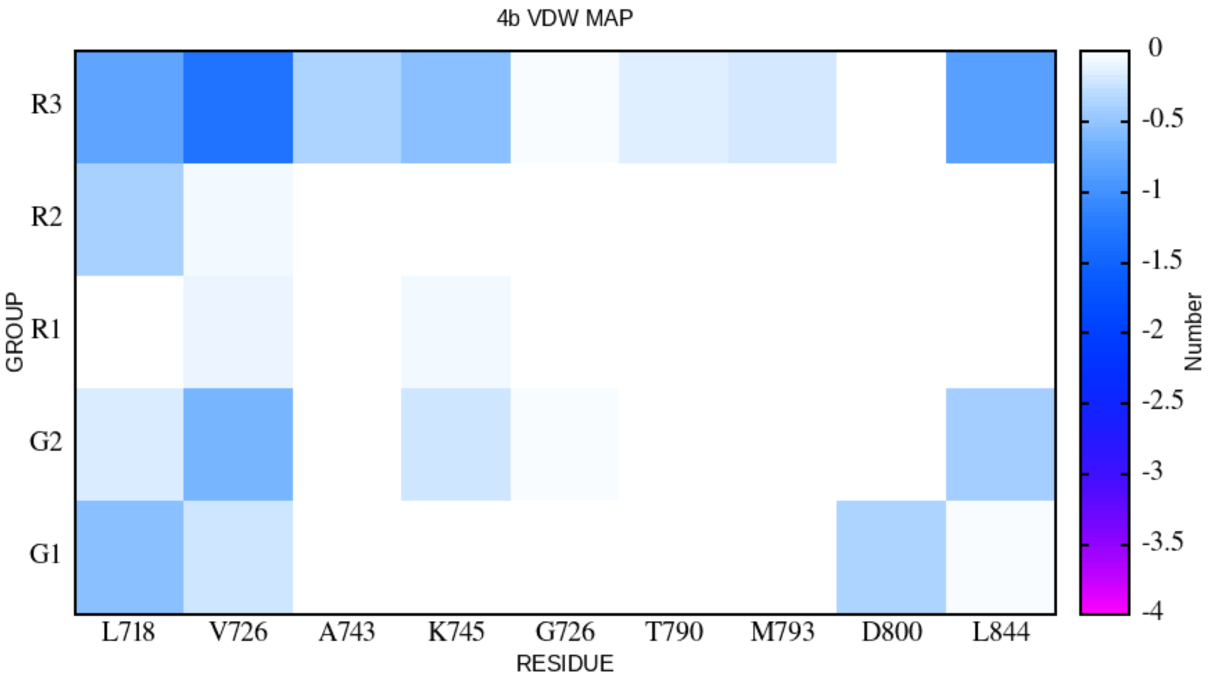
4b VDW MAP
R3
R2
R1
G2
G1
GROUP
L718
V726
A743
K745
G726
T790
M793
D800
L844
RESIDUE
0
-0.5
-1
-1.5
-2
-2.5
-3
-3.5
-4
Number

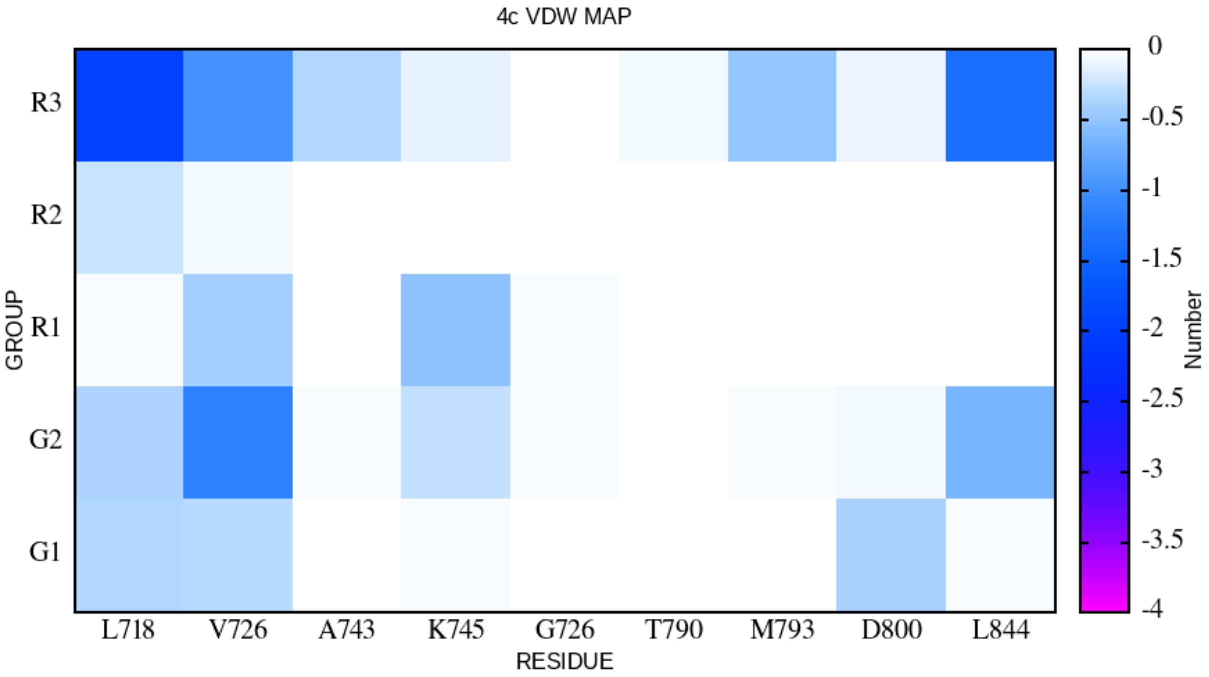
4c VDW MAP
R3
R2
R1
G2
G1
GROUP
L718
V726
A743
K745
G726
T790
M793
D800
L844
RESIDUE
0
-0.5
-1
-1.5
-2
-2.5
-3
-3.5
-4
Number

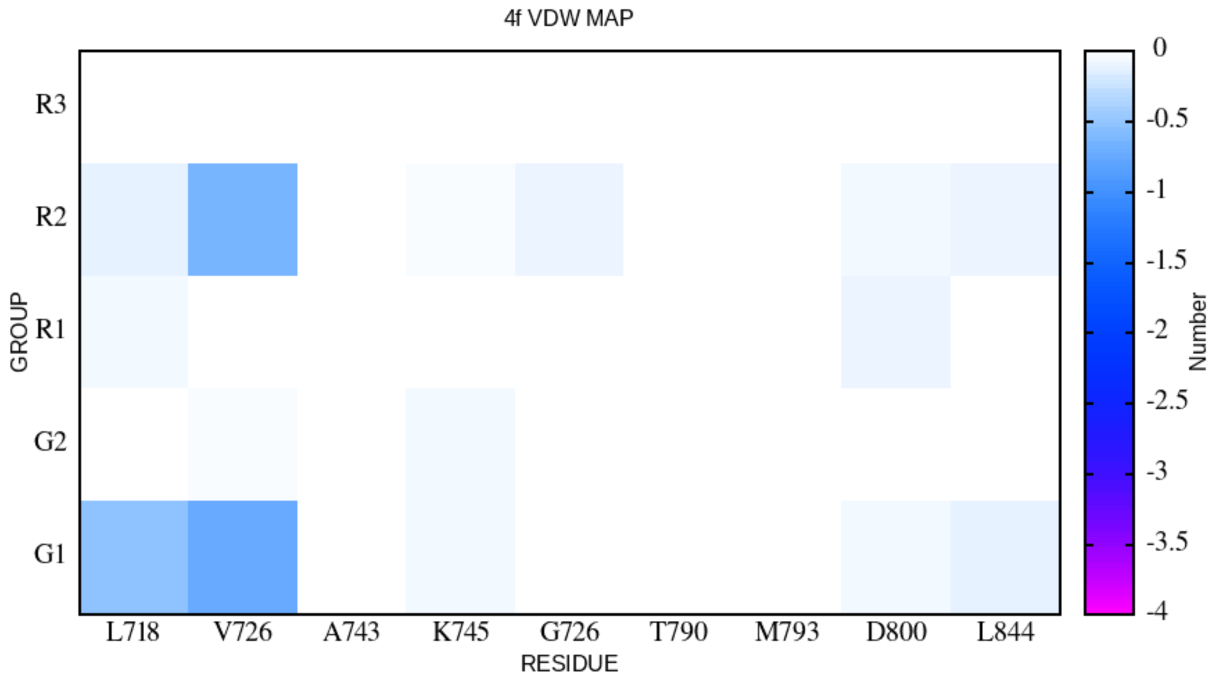
4f VDW MAP
R3
R2
R1
G2
G1
GROUP
L718
V726
A743
K745
G726
T790
M793
D800
L844
RESIDUE
0
-0.5
-1
-1.5
-2
-2.5
-3
-3.5
-4
Number

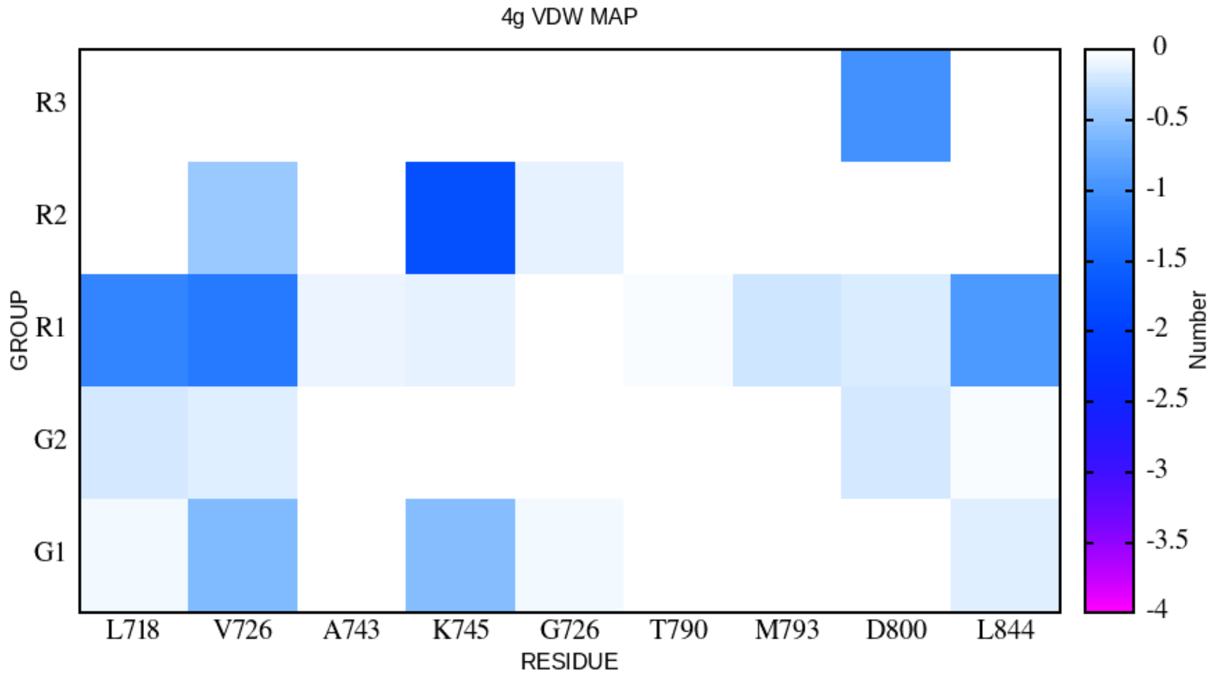
4g VDW MAP
R3
R2
R1
G2
G1
GROUP
L718
V726
A743
K745
G726
T790
M793
D800
L844
RESIDUE
0
-0.5
-1
-1.5
-2
-2.5
-3
-3.5
-4
Number

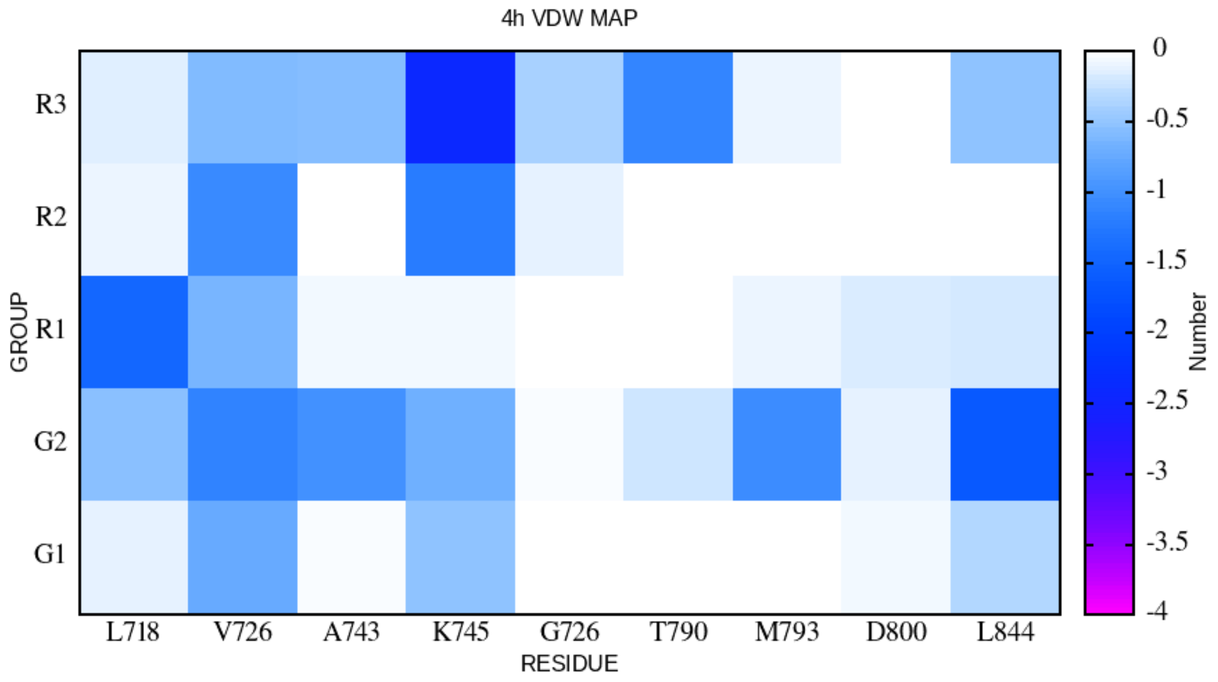
4h VDW MAP
R3
R2
R1
G2
G1
GROUP
L718
V726
A743
K745
G726
T790
M793
D800
L844
RESIDUE
0
-0.5
-1
-1.5
-2
-2.5
-3
-3.5
-4
Number

***Figure S60**: **HOMO and LUMO** of the all compounds is generated by DFT method.*

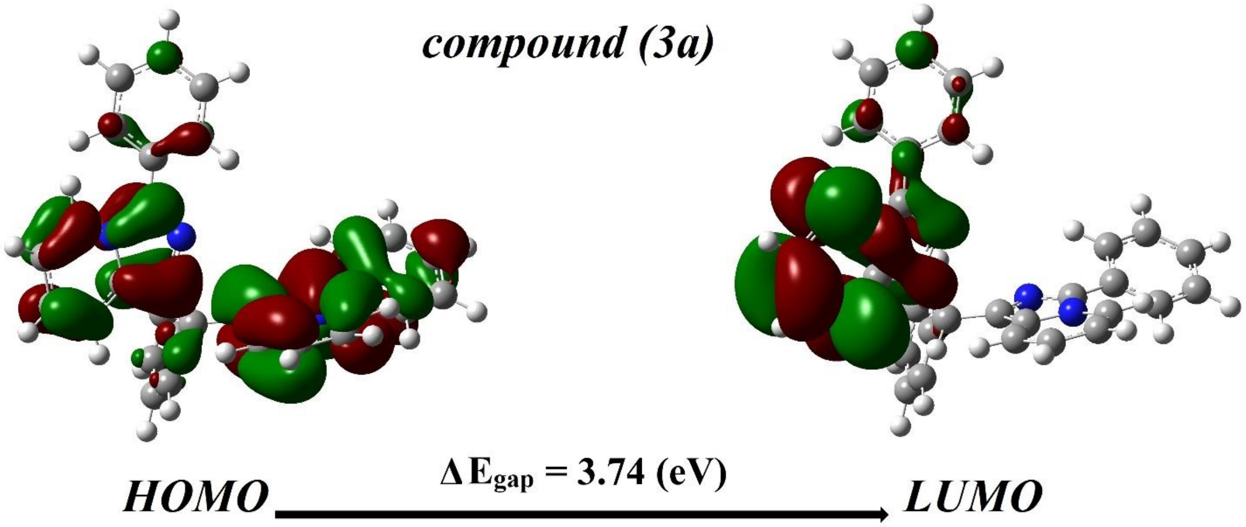

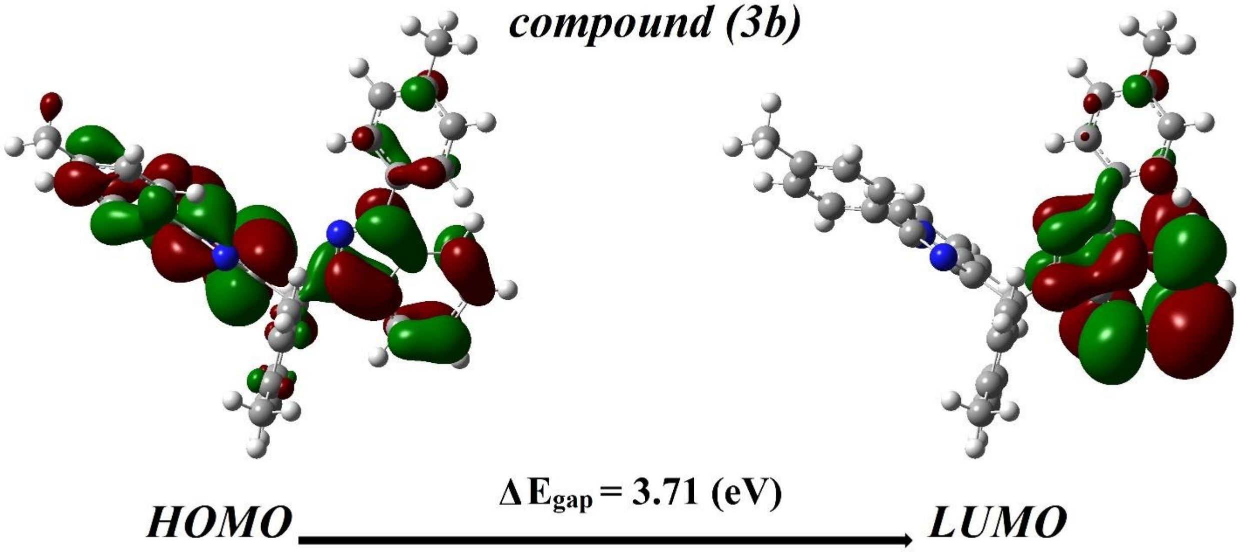

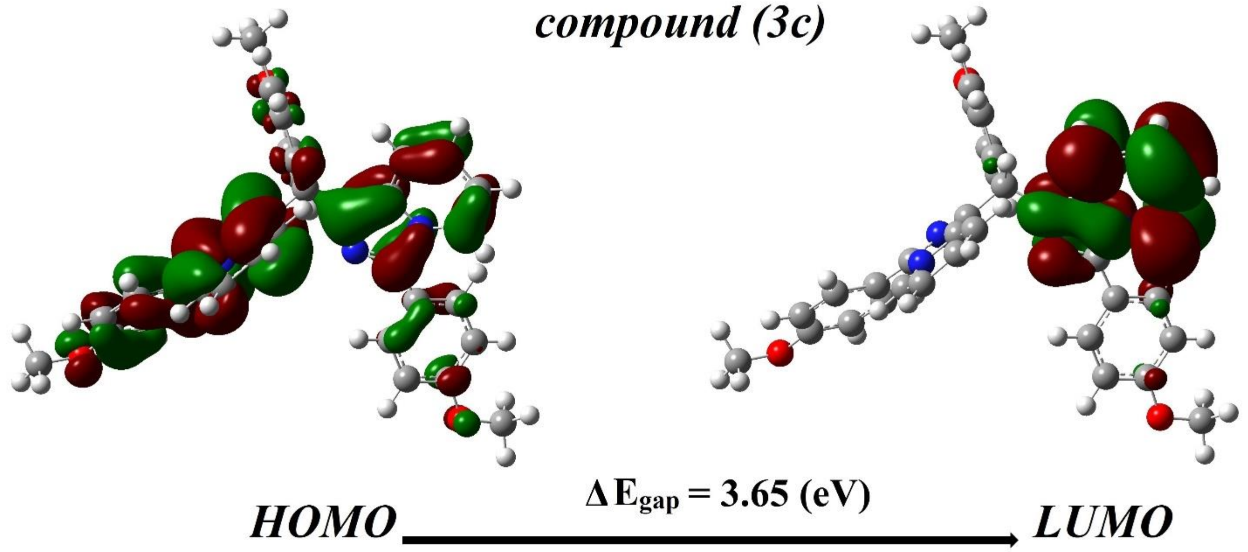
compound (3c)
ΔE$_{gap}$ = 3.65 (eV)
HOMO
LUMO

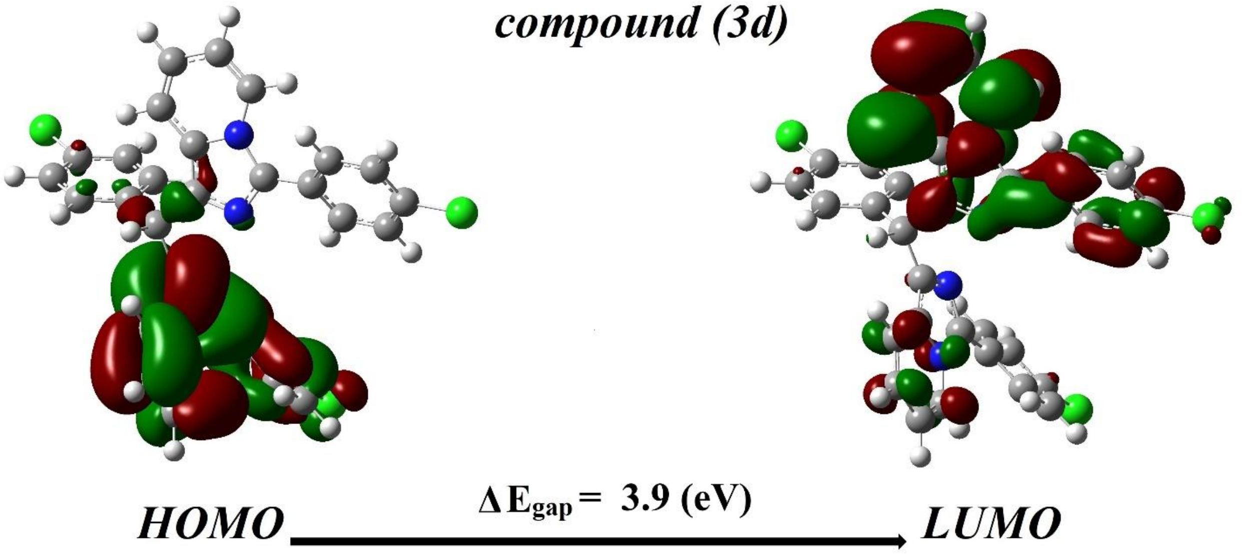
compound (3d)
ΔE$_{gap}$ = 3.9 (eV)
HOMO
LUMO

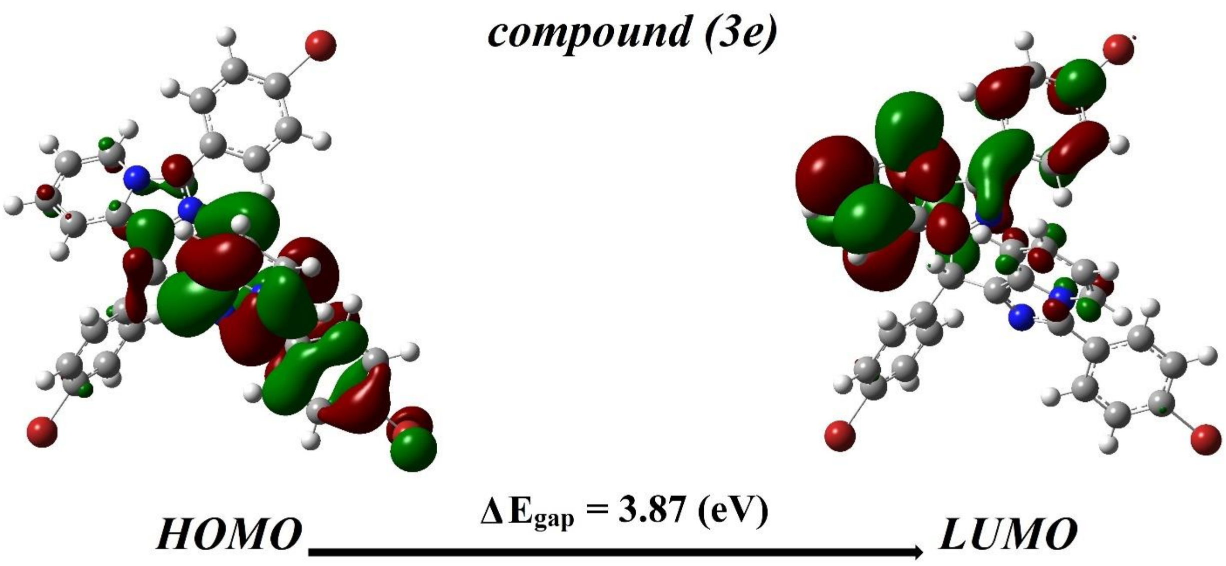

compound (3e)
HOMO
ΔE_{gap} = 3.87 (eV)
LUMO

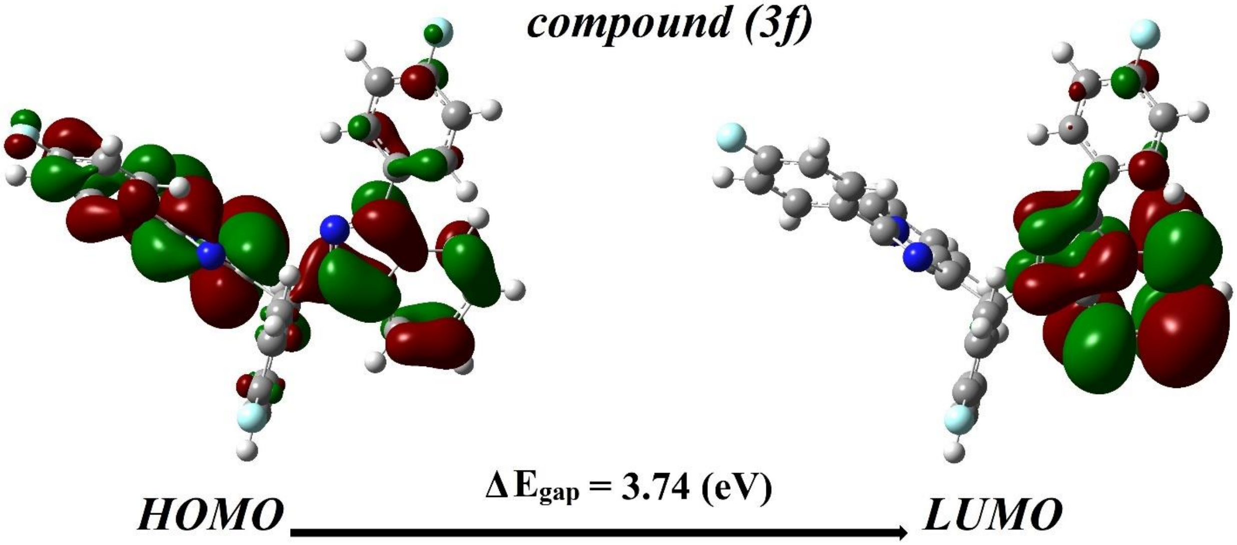

compound (3f)
HOMO
ΔE_{gap} = 3.74 (eV)
LUMO

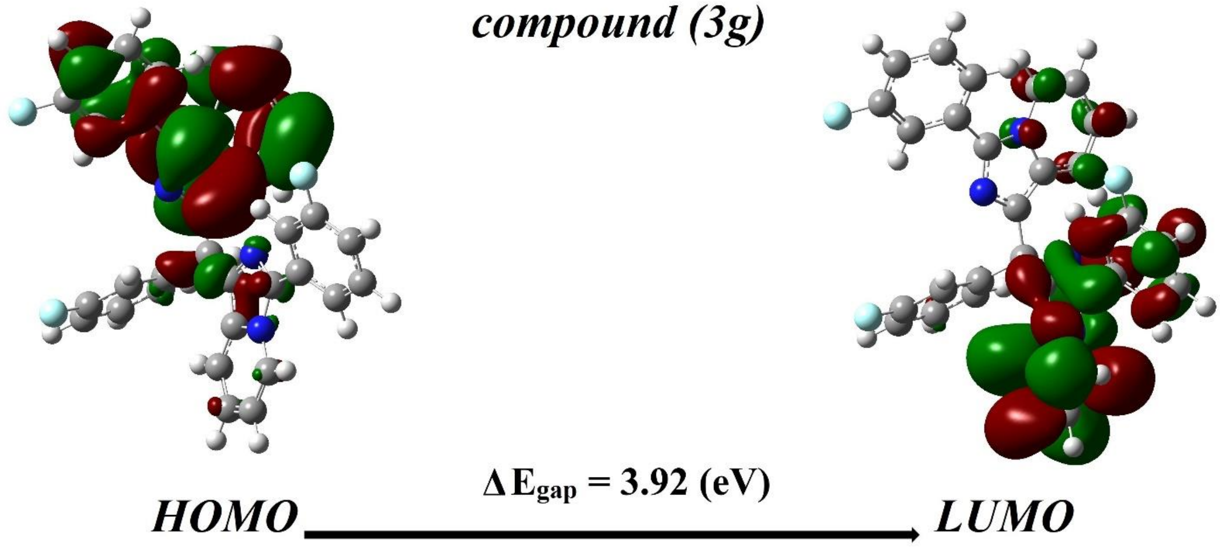
compound (3g)
ΔE$_{gap}$ = 3.92 (eV)
HOMO
LUMO

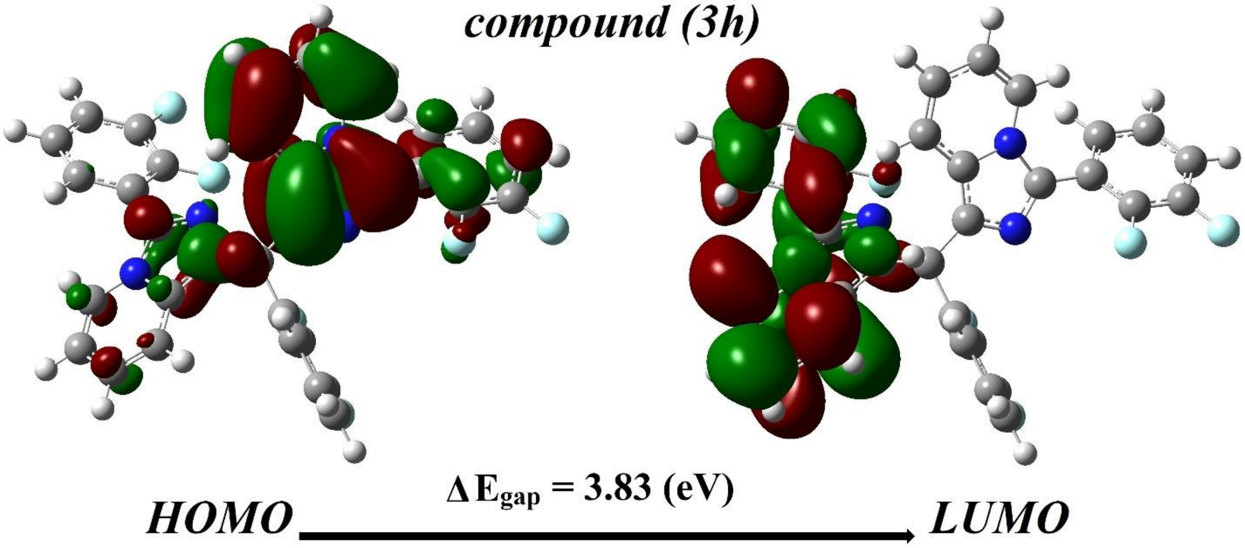
compound (3h)
ΔE$_{gap}$ = 3.83 (eV)
HOMO
LUMO

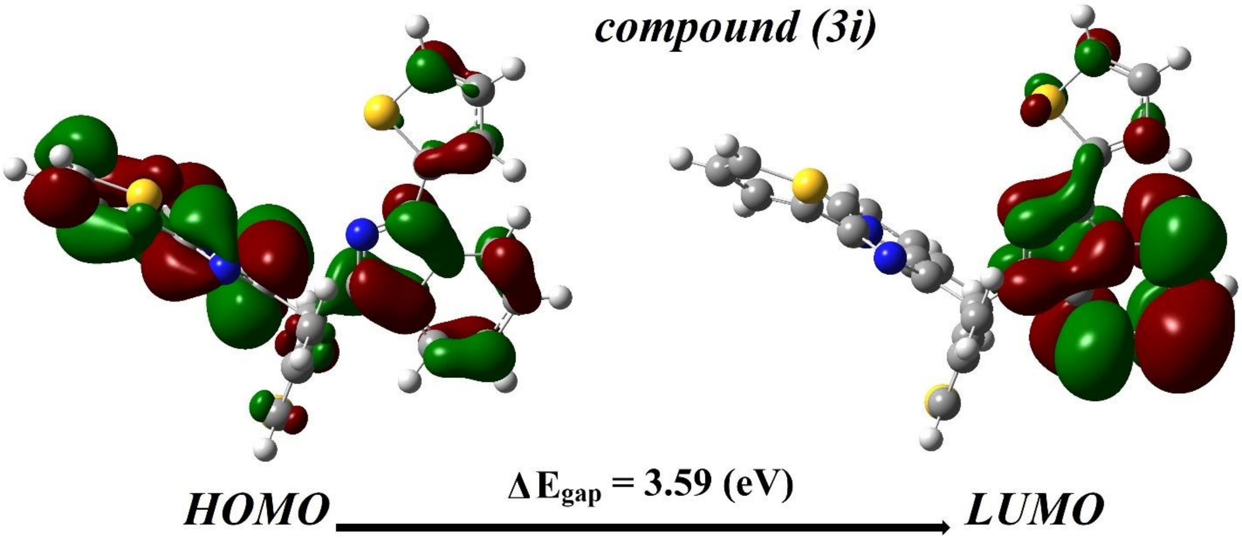
compound (3i)
HOMO
ΔE$_{gap}$ = 3.59 (eV)
LUMO

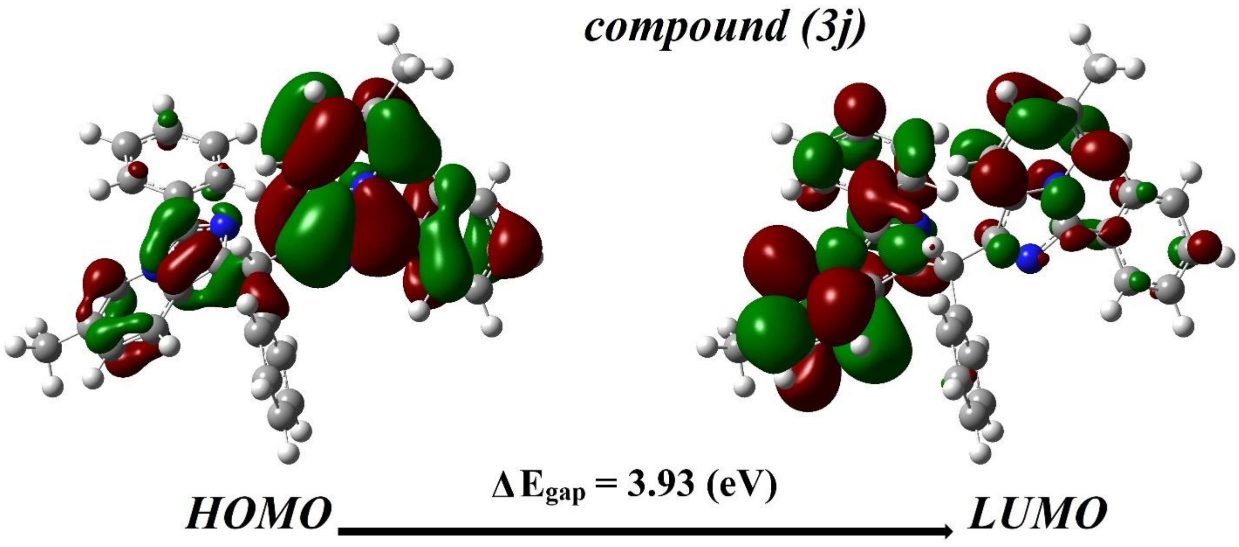
compound (3j)
HOMO
ΔE$_{gap}$ = 3.93 (eV)
LUMO

compound (3k)

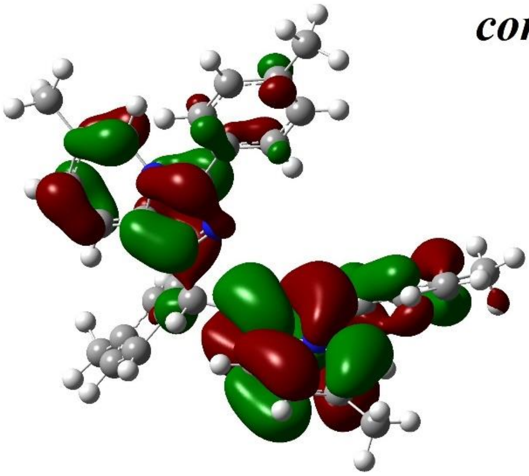

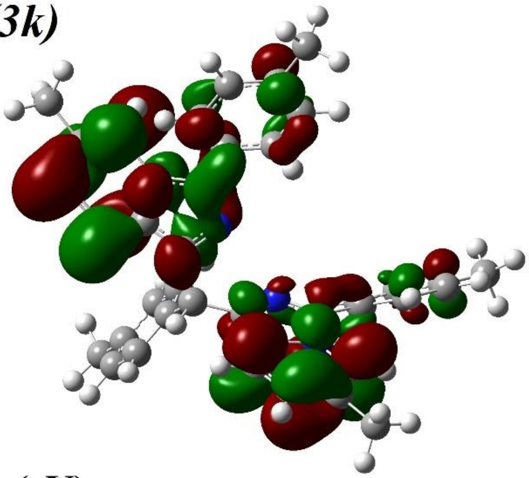

HOMO ——— $\Delta E_{gap} = 3.91$ (eV) ———→ ***LUMO***

compound (4a)

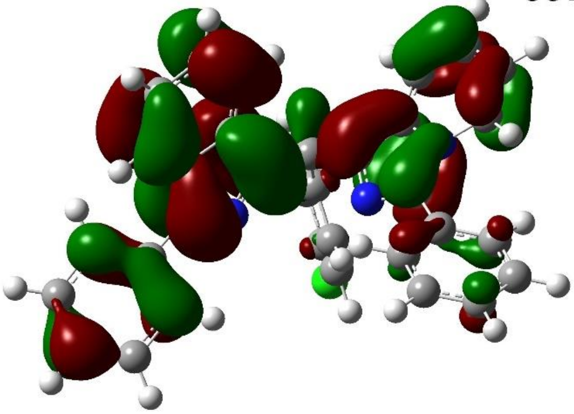

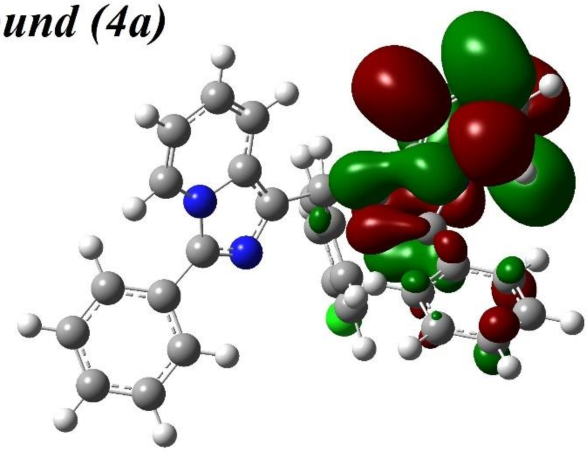

HOMO ——— $\Delta E_{gap} = 3.73$ (eV) ———→ ***LUMO***

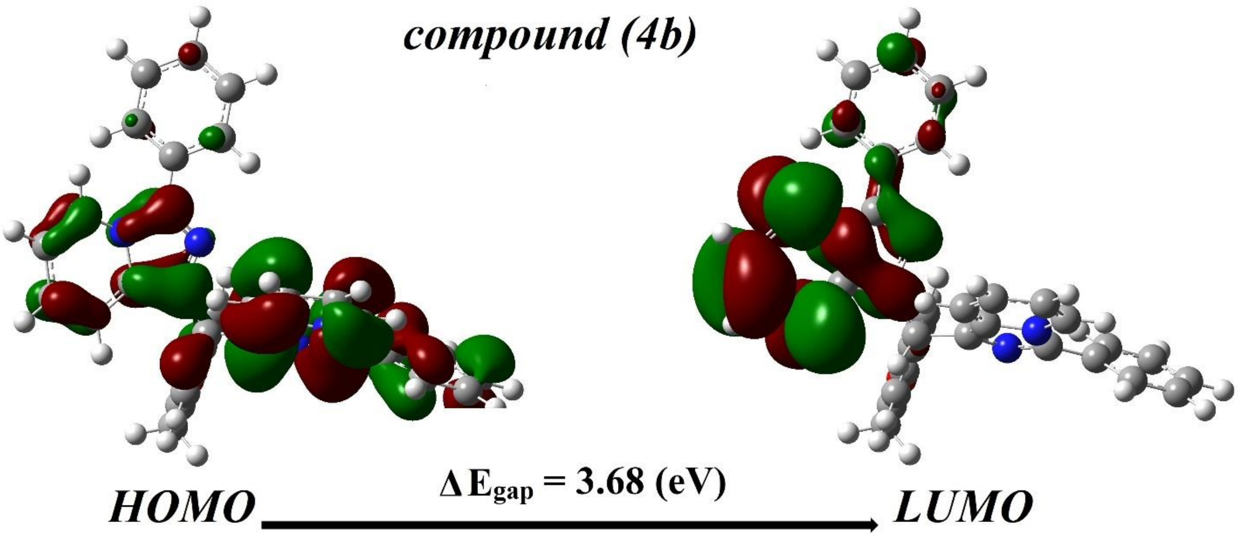
compound (4b)
HOMO
ΔE$_{gap}$ = 3.68 (eV)
LUMO

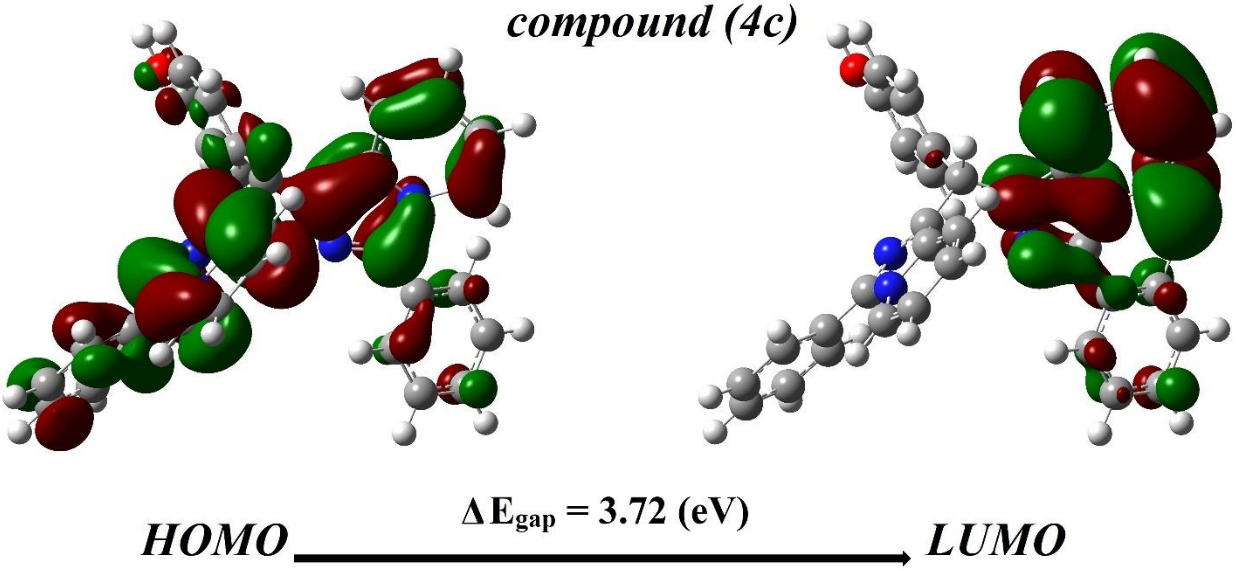
compound (4c)
HOMO
ΔE$_{gap}$ = 3.72 (eV)
LUMO

compound (4d)

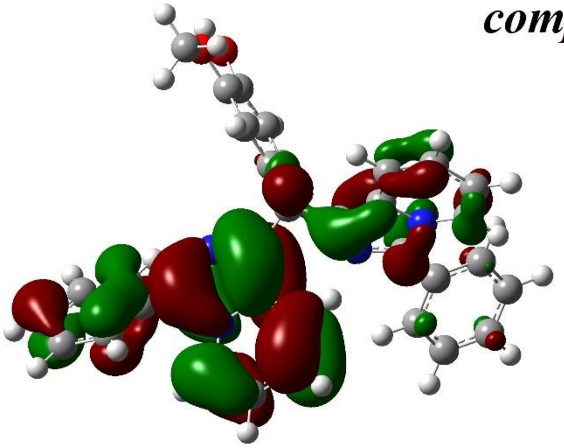

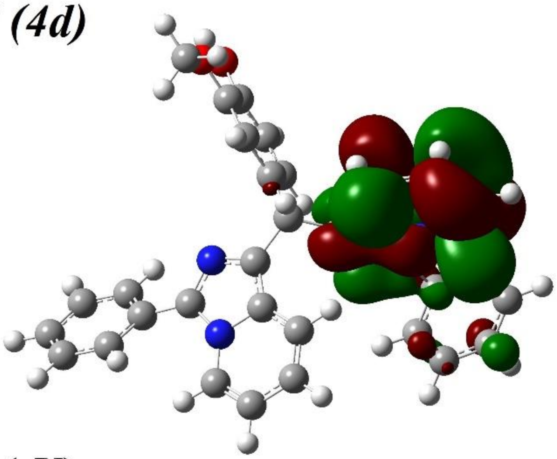

ΔE_{gap} **= 3.68 (eV)**

HOMO → ***LUMO***

compound (4f)

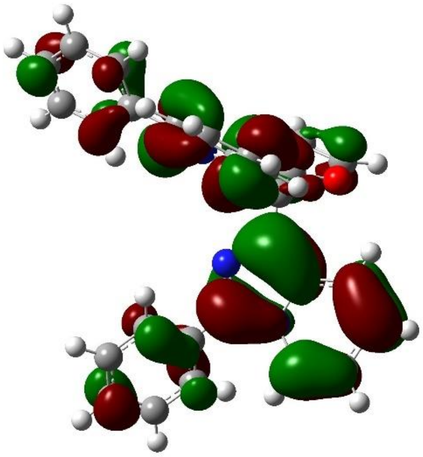

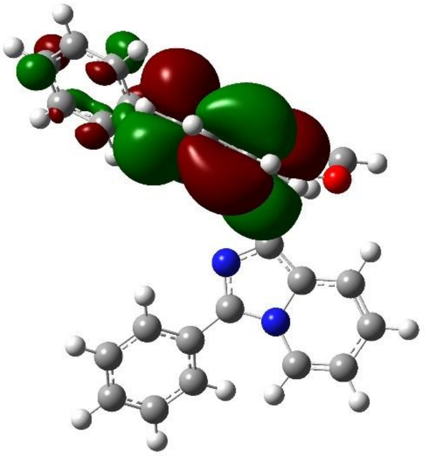

ΔE_{gap} **= 3.84 (eV)**

HOMO → ***LUMO***

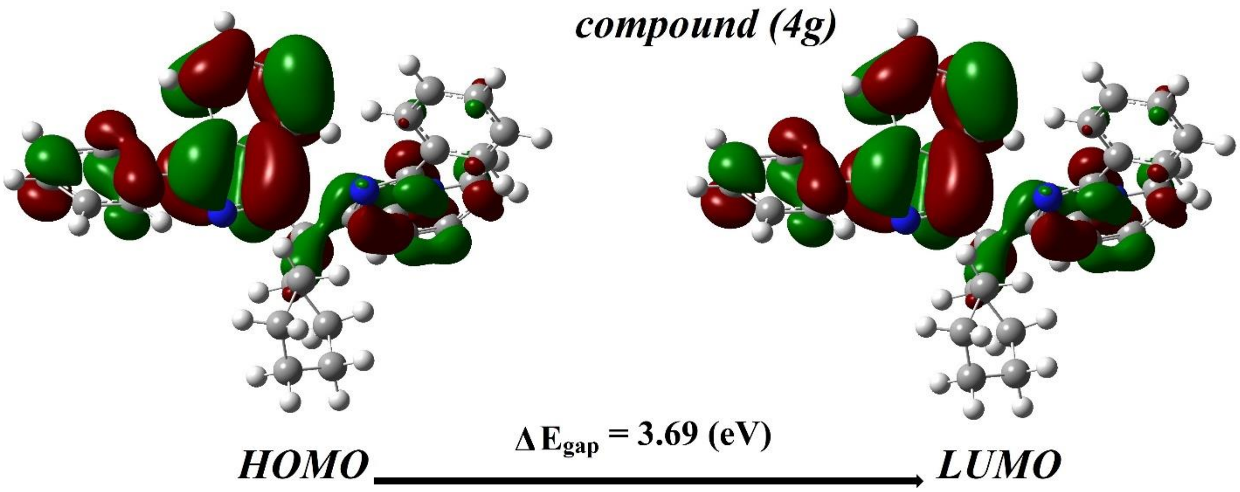
compound (4g)
ΔE_{gap} = 3.69 (eV)
HOMO
LUMO

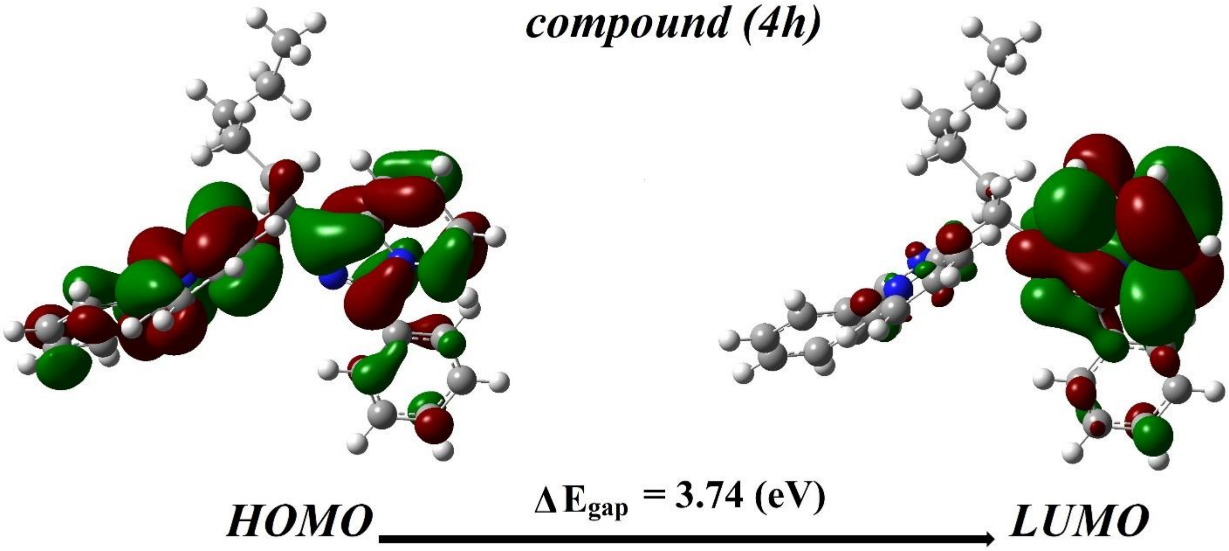
compound (4h)
ΔE_{gap} = 3.74 (eV)
HOMO
LUMO

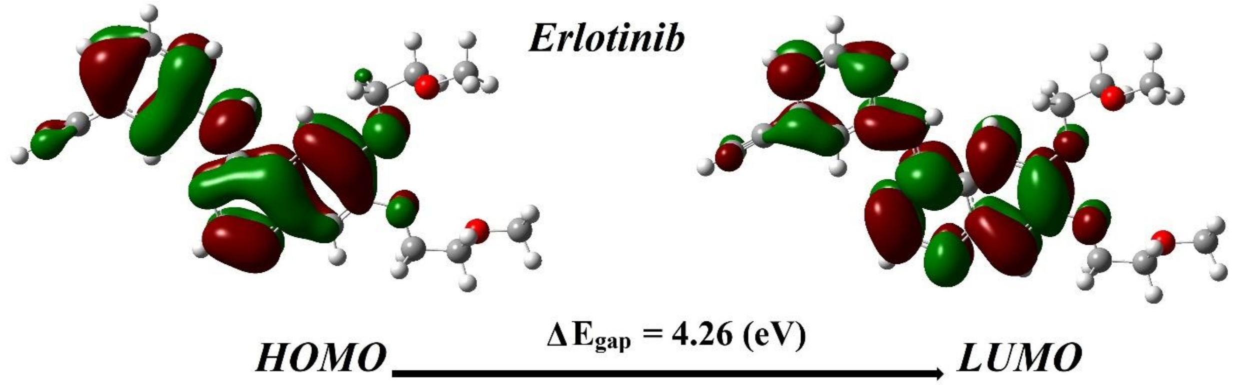
Erlotinib
ΔEgap = 4.26 (eV)
HOMO
LUMO

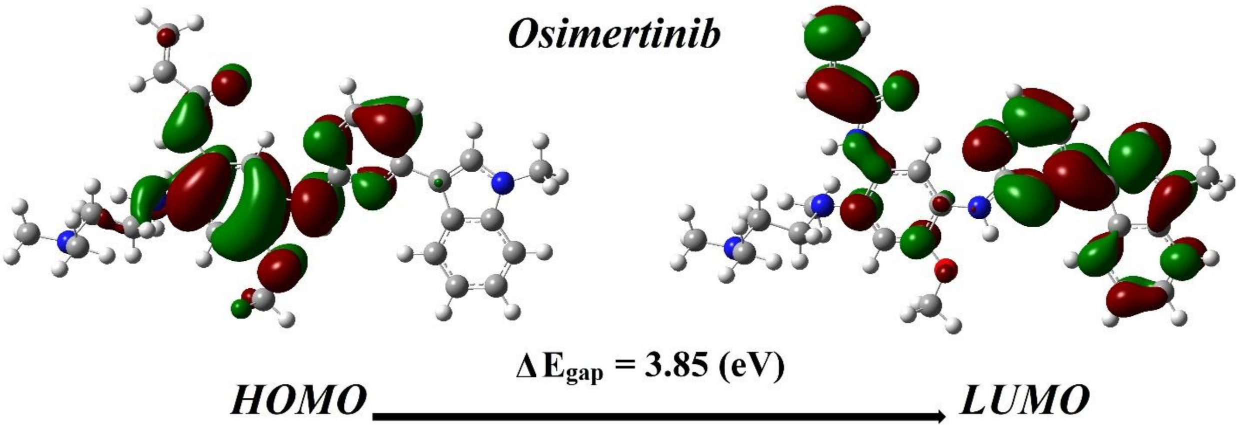
Osimertinib
ΔEgap = 3.85 (eV)
HOMO
LUMO

Reference

1 Ashiru, M. A. *et al.* Identification of EGFR inhibitors as potential agents for cancer therapy: pharmacophore-based modeling, molecular docking, and molecular dynamics investigations. *J Mol Model* **29**, 128, doi:10.1007/s00894-023-05531-6 (2023).

2 Pawara, R., Ahmad, I., Surana, S. & Patel, H. Computational identification of 2,4-disubstituted amino-pyrimidines as L858R/T790M-EGFR double mutant inhibitors using pharmacophore mapping, molecular docking, binding free energy calculation, DFT study and molecular dynamic simulation. *In Silico Pharmacol* **9**, 54, doi:10.1007/s40203-021-00113-x (2021).

3 Kiriwan, D. *et al.* Identification of tripeptides against tyrosine kinase domain of EGFR for lung cancer cell inhibition by in silico and in vitro studies. *Chem Biol Drug Des* **99**, 456-469, doi:10.1111/cbdd.14010 (2022).

4 Yang, Z. *et al.* Computational studies of potent covalent inhibitors on wild type or T790M/L858R mutant epidermal growth factor receptor. *Eur J Pharm Sci* **152**, 105463, doi:10.1016/j.ejps.2020.105463 (2020).

5 Karnik, K. S. *et al.* Development of triple mutant T790M/C797S allosteric EGFR inhibitors: a computational approach. *J Biomol Struct Dyn* **39**, 5376-5398, doi:10.1080/07391102.2020.1786460 (2021).

6 Ahmad, I., Shaikh, M., Surana, S., Ghosh, A. & Patel, H. p38alpha MAP kinase inhibitors to overcome EGFR tertiary C797S point mutation associated with osimertinib in non-small cell lung cancer (NSCLC): emergence of fourth-generation EGFR inhibitor. *J Biomol Struct Dyn* **40**, 3046-3059, doi:10.1080/07391102.2020.1844801 (2022).

7 Maiti, P., Nand, M., Joshi, T., Ramakrishnan, M. A. & Chandra, S. Identification of luteolin -7-glucoside and epicatechin gallate from Vernoniacinerea, as novel EGFR L858R kinase inhibitors against lung cancer: Docking and simulation-based study. *J Biomol Struct Dyn* **39**, 5048-5057, doi:10.1080/07391102.2020.1784791 (2021).

8 Singh, P. K., Chaudhari, D., Jain, S. & Silakari, O. Structure based designing of triazolopyrimidone-based reversible inhibitors for kinases involved in NSCLC. *Bioorg Med Chem Lett* **29**, 1565-1571, doi:10.1016/j.bmcl.2019.05.004 (2019).

9 Tinivella, A. & Rastelli, G. Investigating the Selectivity of Allosteric Inhibitors for Mutant T790M EGFR over Wild Type Using Molecular Dynamics and Binding Free Energy Calculations. *ACS Omega* **3**, 16556-16562, doi:10.1021/acsomega.8b03256 (2018).

10 Uchibori, K. *et al.* Brigatinib combined with anti-EGFR antibody overcomes osimertinib resistance in EGFR-mutated non-small-cell lung cancer. *Nat Commun* **8**, 14768, doi:10.1038/ncomms14768 (2017).
